# Integrative transcriptomic and genomic analysis of odorant binding proteins and chemosensory proteins in aphids

**DOI:** 10.1111/imb.12513

**Published:** 2018-10-05

**Authors:** Q. Wang, J.‐J. Zhou, J.‐T. Liu, G.‐Z. Huang, W.‐Y. Xu, Q. Zhang, J.‐L. Chen, Y.‐J. Zhang, X.‐C. Li, S.‐H. Gu

**Affiliations:** ^1^ State Key Laboratory for Biology of Plant Diseases and Insect Pests, Institute of Plant Protection Chinese Academy of Agricultural Sciences Beijing China; ^2^ Department of Biological Chemistry and Crop Protection Rothamsted Research Harpenden UK; ^3^ College of Plant Protection Shenyang Agricultural University Shenyang China; ^4^ College of Plant Protection Hebei Agricultural University Baoding China; ^5^ Institute of Cotton Research, Hebei Academy of Agriculture and Forestry Sciences Shijiazhuang China; ^6^ Department of Entomology and BIO5 Institute University of Arizona Tucson AZ USA

**Keywords:** *Myzus persicae*, odorant binding protein, chemosensory protein, genomic structure, motif‐pattern, tissue expression profiles

## Abstract

Odorant binding proteins (OBPs) and chemosensory proteins (CSPs) play essential roles in insect chemosensory recognition. Here, we identified nine *OBPs* and nine *CSPs* from the *Myzus persicae* transcriptome and genome. Genomic structure analysis showed that the number and length of the introns are much higher, and this appears to be a unique feature of aphid OBP genes. Three *M. persicae* OBP genes (*OBP3*/*7*/*8*) as well as *CSP1*/*4*/*6*, *CSP2*/*9* and *CSP5*/*8* are tandem arrayed in the genome. Phylogenetic analyses of five different aphid species suggest that aphid OBPs and CSPs are conserved in single copy across all aphids (with occasional losses), indicating that each OBP and CSP class evolved from a single gene in the common ancestor of aphids without subsequent duplication. Motif pattern analysis revealed that aphid OBP and CSP motifs are highly conserved, and this could suggest the conserved functions of aphid OBPs and CSPs. Three *OBPs* (*MperOBP6*/*7*/*10*) are expressed antennae specifically, and five *OBPs* (*MperOBP2*/*4*/*5*/*8*/*9*) are expressed antennae enriched, consistent with their putative olfactory roles. *M. persicae* *CSPs* showed much broader expression profiles in nonsensory organs than *OBPs*. None of the nine *MperCSPs* were found to be antennae specific, but five of them (*MperCSP1*/*2*/*4*/*5*/*6*) showed higher expression levels in the legs than in other tissues. *MperCSP10* mainly expressed in the antennae and legs. The broad and diverse expression patterns of *M. persicae* *CSPs* suggest their multifunctions in olfactory perception, development and other processes.

## Introduction

The green peach aphid *Myzus persicae* (Hemiptera: Aphididae) is a major destructive polyphagous pest. They can use more than 400 plant species from more than 40 families for parthenogenetic reproduction, and use peach, its primary host, for sexual reproduction (Van Emden *et al*., 1969; Weber, 1986; Troncoso *et al*., 2005). Other than its direct feeding damage to plants, *M. persicae* can also transmit more than 100 plant viruses, including both persistent viruses such as potato leaf roll virus (Eskandari *et al*., 1979) and nonpersistent viruses such as cucumber mosaic virus (Bwye *et al*., 1997). In addition, *M. persicae* is a typical host‐alternating aphid species, and exhibits highly colour‐polymorphic association with different host plants. Green *M. persicae* aphids can produce pink offspring when they are fed on poor quality hosts (Williams *et al*., 2000), whereas red *M. persicae* aphids can produce green offspring when they are crowded on poor diets (Ueda and Takada, 1977). The ovipositing female aphids locate their hosts usually by chemical cues and antennal contacts (Read *et al*., 1970). The success of *M. persicae* in nature is due to its extremely high population adaptability to the environment, a wide genetic variability and a broad phenotypic plasticity for which chemical communications play a critical role (Weber, 1986).

Insects use their sensitive and selective olfactory organs, mainly the antennae, to perceive the surrounding world. Aphids, like other insects, rely heavily on chemical signals, including plant volatiles and species‐specific pheromones, to locate correct hosts, find mates and avoid predators or parasitoids (Pickett and Glinwood, 2007). Mature sexual females release sex pheromones from their tibiae of the hind legs to attract conspecific males (Marsh, 1972; Pickett and Glinwood, 2007). The sex pheromones of aphid species usually comprise (4a*S*,7*S*,7a*R*)‐nepetalactone and (1*R*,4a*S*,7*S*,7a*R*)‐nepetalactol, a mixture of two monoterpenoids (Pickett *et al*., 1992; Dewhirst *et al*., 2010). When attacked by predators or parasitoids, most aphid species, including *M. persicae*, emit chemical signals known as alarm pheromones that signal other individuals to escape and defend (Nault *et al*., 1973; Pickett and Griffiths, 1980), or even attack the predator (eg *Ceratovacuna lanigera*) (Arakaki, 1989). The alarm pheromones of most aphids are a mixture of (*E*)‐β‐farnesene (*E*βf), α‐pinene, β‐pinene and β‐limonene (Pickett and Griffiths, 1980). *E*βf, a sesquiterpene hydrocarbon, is the primary component of the alarm pheromones of many aphids, and thus is considered as a repellent to control aphids (Bowers *et al*., 1972). Chemoreception plays important roles in the complex life cycle of aphids. Studying the interaction of aphids with aphids, plants, natural enemies, competitors and mutualists will contribute to exploit novel environment‐friendly strategies for aphid control.

Odorant binding proteins (OBPs) and chemosensory proteins (CSPs) are two families of small water‐soluble proteins; they are highly concentrated (as high as 10 mm) in sensillum lymph of the antennal sensilla and believed to be involved in the initial chemosensory recognition of insects (Vogt and Riddiford, 1981; Calvello *et al*., 2003; Pelosi *et al*., 2006). Both OBPs and CSPs have small molecular weights, approximately 15 kDa for OBPs and 12 kDa for CSPs. The common feature of insect OBPs is that they have six highly conserved cysteines that are paired to form interlocked disulphide bridges (Scaloni *et al*., 1999; Northey *et al*., 2016). Insect CSPs, on other hand, have four conserved cysteines that are linked to form disulphide bridges (Angeli *et al*., 1999). So far, the genomic sequences of four aphid species have been published: the pea aphid *Acyrthosiphon pisum* (The International Aphid Genomics Consortium, 2010), the Russian wheat aphid *Diuraphis noxia* (Nicholson *et al*., 2015), the green peach aphid *M. persicae* (Mathers *et al*., 2017) and the soybean aphid *Aphis glycines* (Wenger *et al*., 2017). Meanwhile, the transcriptomic data of several aphid species are also available (Gu *et al*., 2013; Xue *et al*., 2016). Fifteen *OBPs* and 13 *CSPs* were identified from the *A. pisum* genomic data (Zhou *et al*., 2010). Nine *OBPs* and nine *CSPs* were identified in the cotton aphid *Aphis gossypii* (Gu *et al*., 2013), and 13 *OBPs* and five *CSPs* were identified in the grain aphid *Sitobion avenae* (Xue *et al*., 2016) from their transcriptomic data. For *M. persicae*, only four OBPs and five CSPs were identified from the expressed sequence tags (Xu *et al*., 2009). The functional studies of aphid OBPs and CSPs in recognizing and discriminating the sex pheromones, the alarm pheromone and the host plant volatiles, however, are still limited. The OBP3 of *A. pisum* (Qiao *et al*., 2009), the OBP7 of *S. avenae* (Vandermoten *et al*., 2011; Zhong *et al*., 2012), the OBP3 of *Megoura viciae* and *Nasonovia ribisnigri* (Northey *et al*., 2016) and the OBP3 and OBP7 of *Rhopalosiphum padi* (Fan *et al*., 2017) have high binding affinities with the alarm pheromone *E*βf. However, the *in* *vivo* evidence of OBP3 and OBP7 participating in *E*βf recognition is still not conclusive.

In this study, we identified nine OBP and nine CSP genes from the *M. persicae* transcriptomic and genomic sequence datasets and for the first time demonstrated their genomic structures, clusters and uniquely long introns. We further analysed their motif patterns and phylogenetic relationships with the OBPs and CSPs in five other aphid species and in other hemipterans. We also examined the tissue expression profiles of *M. persicae* OBP and CSP genes to infer their putative functions in aphid chemoreception of semiochemicals. The evolution and differentiation of Hemiptera OBPs and CSPs are also discussed.

## Results

### *Sequencing, *de novo *assembly and functional annotation*

A total of 110 059 204 raw reads were produced from the apterous *M. persicae* transcriptome sequencing project. After trimming adaptor sequences and removing low‐quality sequences, 107 199 360 clean reads were remained. After assembly, 50 352 unigenes were generated, with lengths ranging from 201 bp to 27.17 kb and an average length of 829 bp (N50 = 1745 bp, N90 = 294 bp).

The unigenes were searched with blastx and blastn programs against the sequences in the NCBI GenBank database. The results indicated that 15 881 of the 50 352 unigenes (31.54%) had blastx hits in the nonredundant protein (nr) databases, and 14 329 unigenes (28.46%) had blastn hits in the nonredundant nucleotide sequence (nt) databases. Some unigenes are homologous to more than one species, and most of the annotated unigenes have the best hit with Hemiptera insect genes.

**Figure 1:**
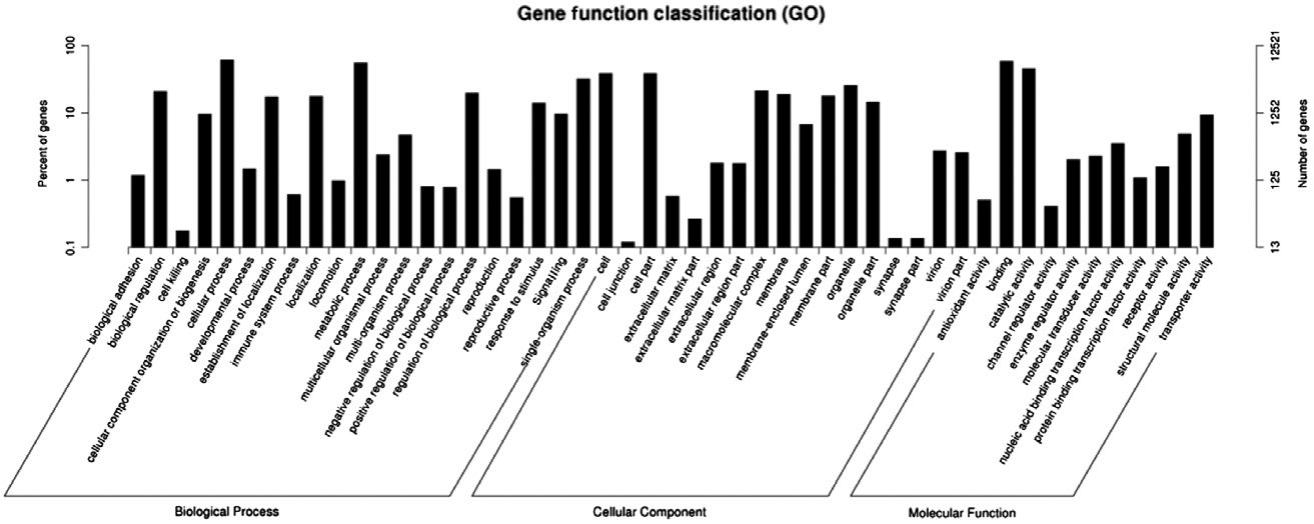
Gene Ontology (GO) classifications of the 50352 *Myzus persicae* unigenes.

The Gene Ontology (GO) category analysis revealed that only 12 521 of the 50 352 unigenes (24.87%) could be annotated into different functional groups (biological process, cellular components and molecular functions). The cellular process (7650 unigenes) and metabolic process (6913 unigenes) GO categories were most abundantly represented within the biological process GO. In the cellular components GO, the transcripts were mainly distributed in the cell (4832 unigenes) and cell part (4935 unigenes). The GO analysis also showed that the unigenes involving in binding (7306 unigenes) and catalytic activity (5696 unigenes) were most abundant in the molecular function ontology (Fig. 1).

### *Identification of OBP and CSP genes in *M. persicae

**Figure 2:**
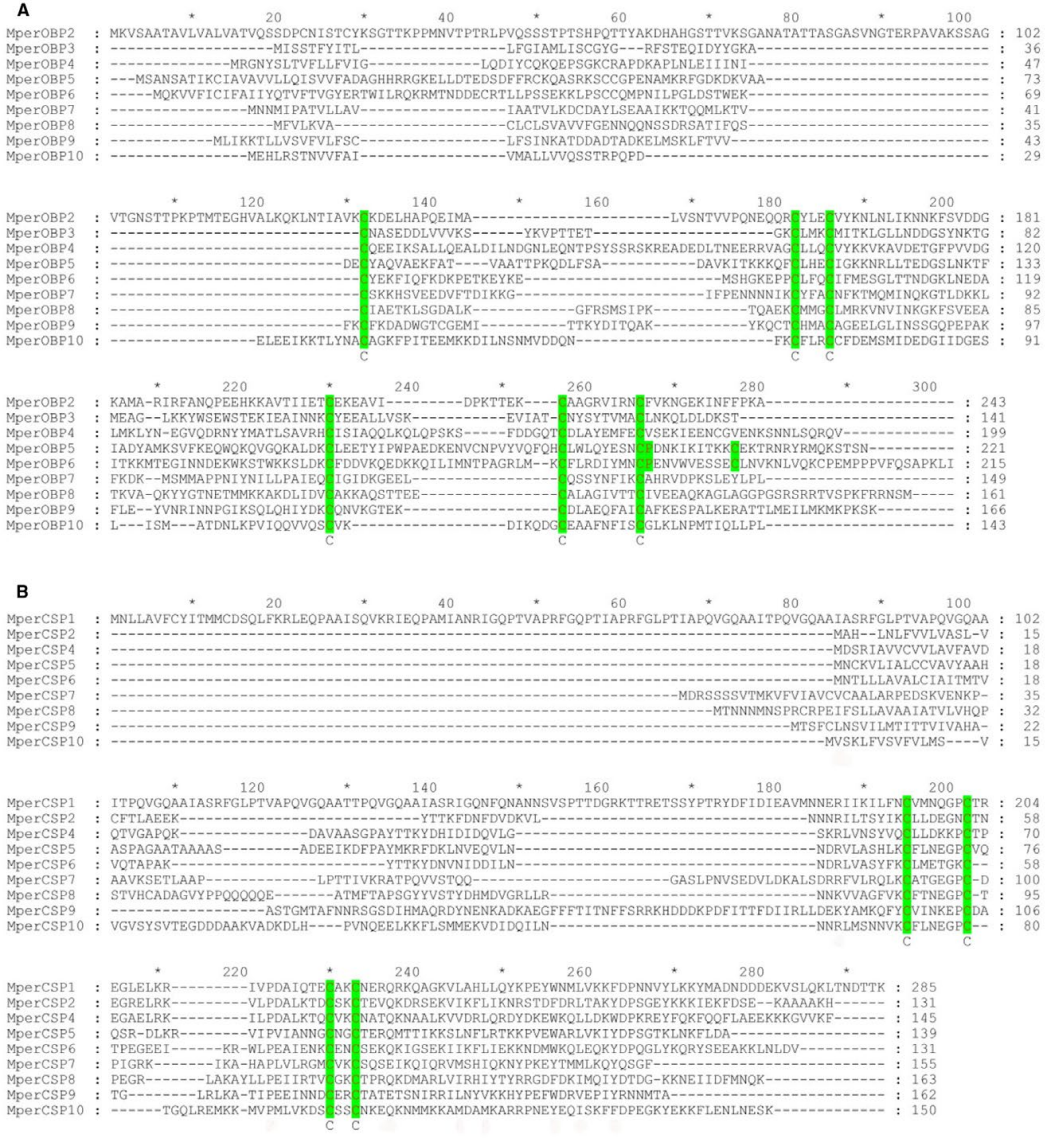
Alignment of the *Mysuz persicae* odorant binding proteins (OBPs) and chemosensory proteins (CSPs). The full‐length amino acid sequences are aligned by ClustalX 2.1 and then edited using GeneDoc. (A) Alignment of the *M. persicae* OBPs. The six conserved cysteine residues are indicated by the green shading. (B) Alignment of the *M. persicae* CSPs. The four conserved cysteine residues are shaded in green. [Colour figure can be viewed at wileyonlinelibrary.com]

A total of nine OBP and nine CSP genes were identified from the transcriptome and genome of *M. persicae* using motifsearch and tblastn program (Table 1). The identification of these OBPs and CSPs was confirmed by searching *M. persicae* genome sequences (https://bipaa.genouest.org/is/aphidbase/myzus_persicae). All *M. persicae* *OBPs* and *CSPs* are full‐lengths with open reading frames (ORFs) ranging from 396 to 858 bp. The nucleotide sequences of *M. persicae* OBP and CSP genes were verified by molecular cloning and sequencing. We name these *M. persicae* OBP genes as *MperOBP2–10* and the CSP genes as *MperCSP1–2* and *MperCSP4–10* following the nomenclatures reported for the OBPs and CSPs of *A. pisum* (Zhou *et al*., 2010), *A. gossypii* (Gu *et al*., 2013) and *S. avenae* (Xue *et al*., 2016). Like in *A. gossypii* (Gu *et al*., 2013), both *OBP1* and *CSP3* identified in *A. pisum* are missing in the *M. persicae* transcriptome and genome dataset. Like AgosCSP1 (Gu *et al*., 2013), MperCSP1 does not have a signal peptide, a signature of secretory protein. The rest of the *M. persicae* OBP and CSP proteins all have a signal peptide at their N‐terminus (Table 1). The nine identified *M. persicae* OBPs can be divided into two distinct subfamilies: MperOBP2/3/4/7/8/9/10 belong to the classical OBP subfamily, which has the typical six conserved cysteines and fit the motif pattern of ‘C_1_‐X_15–39_‐C_2_‐X_3_‐C_3_‐X_21–44_‐C_4_‐X_7–12_‐C_5_‐X_8_‐C_6_’ (Fig. 2A, Zhou *et al*., 2004; Xu *et al*., 2009), MperOBP5 and MperOBP6 belong to the plus‐C OBP subfamily, which has one additional cysteine (C6a) and a conserved proline immediately after the sixth cysteine (Fig. 2A, Zhou *et al*., 2004). All of the MperCSPs have four conserved cysteines and fit the motif pattern of ‘C_1_‐X_6–8_‐C_2_‐X_16–21_‐C_3_‐X_2_‐C_4_’ (Fig. 2B, Zhou *et al*., 2004). The nucleotide sequences of *MperOBPs* and *MperCSPs* have been deposited in GenBank under the accession numbers MG356454 to MG356471.

**Table 1:** List of odorant binding proteins and chemosensory proteins in *Myzus persicae*

Gene name	RPKM	Signal peptide (aa)	Amino acids (aa)	ORF (bp)	Scaffold*	Accession no.	Gene annotation	Homology search with other aphids				
								Species	Protein ID	Score (bits)	*E*‐value	Identity (%)
*MperOBP2*	383	1–19	244	732	105 (340085…345920,−)	MG356454	Odorant‐binding protein 2	*A. cyrthosiphon pisum*	NP 001153528	384	2e‐133	96
*MperOBP3*	135	1–23	142	426	21 (798710…815519,+)	MG356455	Odorant‐binding protein 3	*A. cyrthosiphon pisum*	NP 001153529	281	6e‐96	96
*MperOBP4*	21	1–22	200	600	300 (285472…288740,+)	MG356456	Odorant‐binding protein 4	*S. avenae*	APB03427	400	7e‐141	97
*MperOBP5*	99	1–25	222	666	4 (1732552…1743198,−)	MG356457	Odorant‐binding protein 5	*A. cyrthosiphon pisum*	NP 001153531	450	4e‐160	97
*MperOBP6*	73	1–19	216	648	77 (15902…21431,−)	MG356458	Odorant‐binding protein 6	*S. avenae*	APB03429	394	4e‐138	87
*MperOBP7*	65	1–24	150	450	21 (790748…798189,+)	MG356459	Odorant‐binding protein 7	*A. cyrthosiphon pisum*	NP 001153533	251	1e‐83	87
*MperOBP8*	33	1–18	162	486	21 (816539…821387,+)	MG356460	Odorant‐binding protein 8	*A. cyrthosiphon pisum*	NP 001153534	314	3e‐108	96
*MperOBP9*	19	1–24	167	501	422 (21121…26183,+)	MG356461	Odorant‐binding protein 9	*S. avenae*	APB03432	265	2e‐88	90
*MperOBP10*	8	1–24	144	432	47 (690675…693525,−)	MG356462	Odorant‐binding protein 10	*S. avenae*	APB03433	238	9e‐79	76
*MperCSP1*	16	No SP	286	858	112 (593787…605068,−)	MG356463	Chemosensory protein 1	*A. gossypii*	AGE97641	273	1e‐89	87
*MperCSP2*	330	1–20	132	396	55 (285362…286497,−)	MG356464	Chemosensory protein 2	*A. gossypii*	AGE97642	233	3e‐77	88
*MperCSP4*	286	1–22	146	438	112 (578383…579415,−)	MG356465	Chemosensory protein 4	*A. gossypii*	AGE97643	256	6e‐86	94
*MperCSP5*	609	1–19	140	420	10 (1364996…1366765,−)	MG356466	Chemosensory protein 5	*A. gossypii*	AGE97644	284	1e‐156	92
*MperCSP6*	91	1–21	132	396	112 (615905…617608,−)	MG356467	Chemosensory protein 6	*A. gossypii*	AEG97645	208	3e‐67	87
*MperCSP7*	40	1–24	156	468	910 (34801…42428,−)	MG356468	Chemosensory protein 7	*A. gossypii*	AGE97646	280	4e‐95	89
*MperCSP8*	38	1–37	164	492	10 (1379554…1382877,−)	MG356469	Chemosensory protein 8	*A. gossypii*	AGE97647	240	9e‐79	75
*MperCSP9*	330	1–22	163	489	55 (282954…285123,+)	MG356470	Chemosensory protein 9	*A. gossypii*	AGE97648	212	8e‐68	60
*MperCSP10*	3	1–21	151	453	420 (101975…105884,−)	MG356471	Chemosensory protein 10	*A. gossypii*	AGE97649	177	2e‐54	83

aa, amino acids; ORF, open reading frame; RPKM, reads per kilobase per million; SP, signal peptide.

* AphidBase scaffold ID (start…stop nt, orientation).

### Phylogenetic analyses of Hemiptera OBPs and CSPs

**Figure 3:**
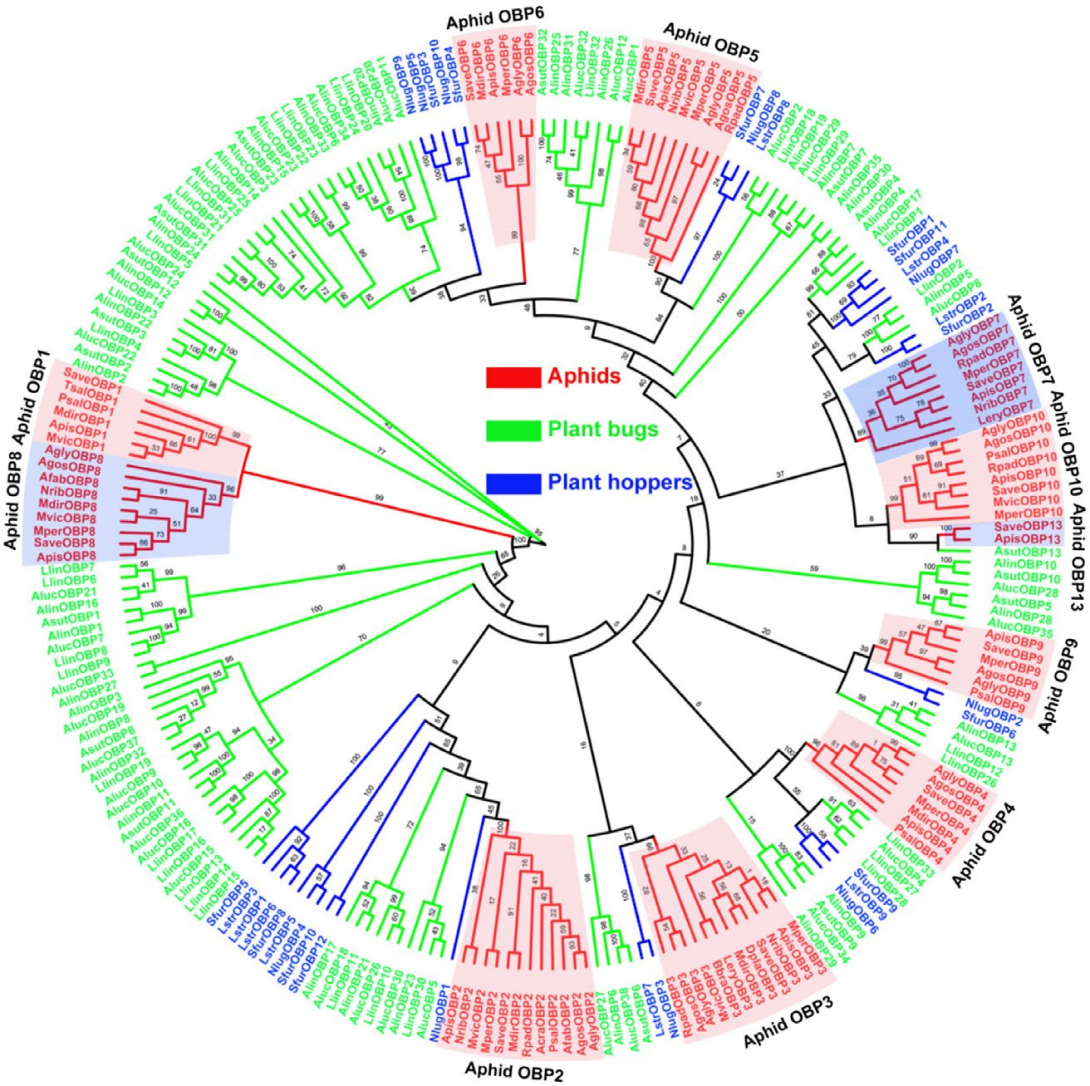
Phylogenetic tree of 237 odorant binding proteins (OBPs) from Hemiptera species. The phylogenetic tree of Hemiptera OBPs was built using 237 OBP sequences from 16 different aphid species (*Myzus persicae*, *Aphis gossypii*, *Acyrthosiphon pisum*, *Aphis glycines*, *Sitobion avenae*, *Pterocomma salicis*, *Aphis fabae*, *Aphis craccivora*, *Tuberolachnus salignus*, *Megoura* *viciae*, *Metopolophium dirhodum*, *Nasonovia* *ribisnigri*, *Rhopalosiphum padi*, *Lipaphis erysimi*, *Drepanosiphum platanoidis* and *Brevicoryne brassicae*), four plant bugs (*Adelphocoris suturalis*, *Apolygus lucorum*, *Adelphocoris lineolatus* and *Lygus lineolaris*) and three plant hoppers (*Nilaparvata lugens*, *Laodelphax striatellus *and *Sogatella furcifera*). The protein names and sequences that were used in phylogenetic analysis are listed in Supporting Information Table S8. [Colour figure can be viewed at wileyonlinelibrary.com]

MperOBPs share relatively low amino acid identities (8–59%) with each other (Supporting Information Table S6), and MperCSPs show relatively high amino acid identities (10–71%) with each other (Supporting Information Table S7). But the amino acid identities of orthologous OBPs and CSPs in the five aphid species of the Aphididae family (*M. persicae*, *A. gossypii*, *A. pisum*, *A. glycines* and *S. avenae*) are much higher (eg 93% among the OBP3 orthologues and 94% among the CSP4 orthologues). The phylogenetic tree of Hemiptera OBPs was built using 237 OBP sequences from 16 different aphid species (*M. persicae*, *A. gossypii*, *A. pisum*, *A. glycines*, *S. avenae*, *Pterocomma salicis*, *Aphis fabae*, *Aphis craccivora*, *Tuberolachnus salignus*, *M. viciae*, *Metopolophium dirhodum*, *N. ribisnigri*, *R. padi*, *Lipaphis erysimi*, *Drepanosiphum platanoidis* and *Brevicoryne brassicae*), four plant bug species (*Adelphocoris suturalis*, *Apolygus lucorum*, *Adelphocoris lineolatus* and *Lygus lineolaris*) and three plant hopper species (*Nilaparvata lugens*, *Laodelphax striatellus* and *Sogatella furcifera*) (Supporting Information Table S8; Fig. 3). Aphid OBPs are clearly separated from OBPs from plant bugs and plant hoppers and clustered together to form multiple homologous subgroups (lineages) named OBP1 to OBP10 subgroups supported by high bootstrap values of 89–100 (Fig. 3), indicating that these subgroups evolved from a single gene in the common ancestor of aphids. Furthermore, OBP1 subgroup and OBP8 subgroup are more closely related and clustered into a branch with an average amino acid identity of 61.54% and a bootstrap value of 99. The OBP1 and OBP8 subgroups contain OBPs from 11 aphid species (*M. persicae*, *A. gossypii*, *A. pisum*, *A. glycines*, *S. avenae*, *M. dirhodum*, *A. fabae*, *N. ribisnigri*, *M. viciae*, *T. salignus* and *P. salicis*), suggesting a more recent evolution into OBP1 subgroup and OBP8 subgroup from a common ancestor before aphid speciation (Zhou *et al*., 2010).

**Figure 4:**
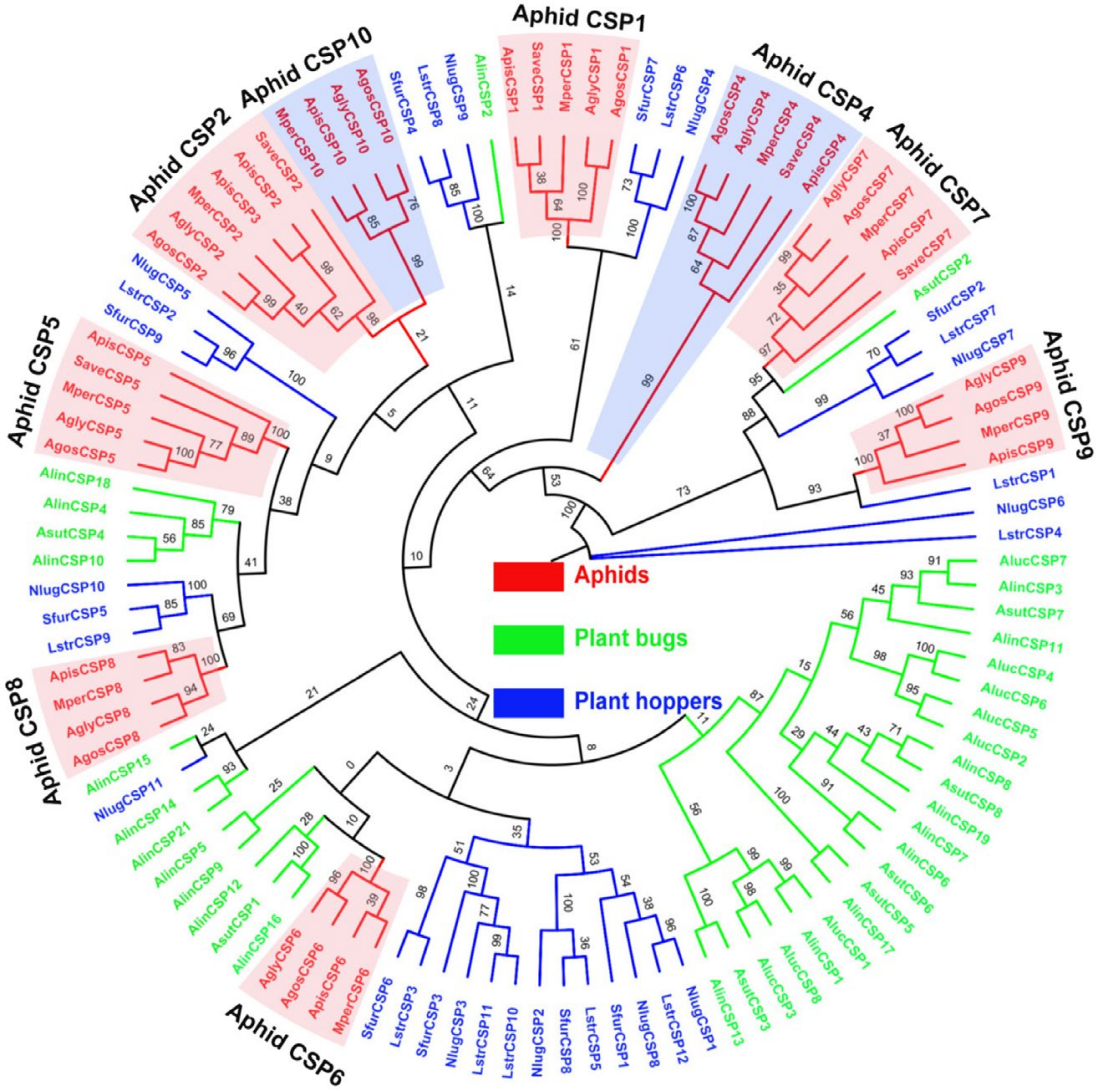
Phylogenetic tree of 110 chemosensory proteins (CSPs) from Hemiptera species. The phylogenetic tree of Hemiptera CSPs was built using 110 CSP sequences from five different aphid species (*Myzus persicae*, *Aphis gossypii*, *Acyrthosiphon pisum*, *Aphis glycines* and *Sitobion avenae*), three plant bugs (*Adelphocoris suturalis*, *Apolygus lucorum *and *Adelphocoris lineolatus*) and three plant hoppers (*Nilaparvata lugens*, *Laodelphax striatellus *and *Sogatella furcifera*). The protein names and sequences that were used in phylogenetic analysis are listed in Supporting Information Table S9. [Colour figure can be viewed at wileyonlinelibrary.com]

The phylogenetic tree of Hemiptera CSPs was built using 110 CSP sequences from five different aphid species (*M. persicae*, *A. gossypii*, *A. pisum*, *A. glycines* and *S. avenae*), three plant bugs (*A. suturalis*, *A. lucorum* and *A. lineolatus*) and three plant hoppers (*N. lugens*, *L. striatellus* and *S. furcifera*) (Fig. 4, Supporting Information Table S9). Like the OBPs shown in Fig. 3, the phylogenetic analysis clusters aphid CSPs into several clear clades of nine homologous subgroups (CSP1, CSP2, CSP4, CSP5, CSP6, CSP7, CSP8, CSP9 and CSP10). Interestingly, some aphid CSP subgroups are clustered with non‐aphid CSP genes in conserved clades. For example, the plant bug AsutCSP2 and the aphid CSP7 subgroup are clustered in the same clade with a bootstrap value as high as 95, and the plant hopper LstrCSP1 and the aphid CSP9 subgroup are clustered in the same branch with a bootstrap value as high as 93 (Fig. 4). This suggests that the AsutCSP2 and aphid CSP7s and the LstrCSP1 and aphid CSP9s evolved from the same ancestor and may play similar roles in these sucking insects.

### *Genomic structure of *M. persicae *OBP and CSP genes*

**Figure 5:**
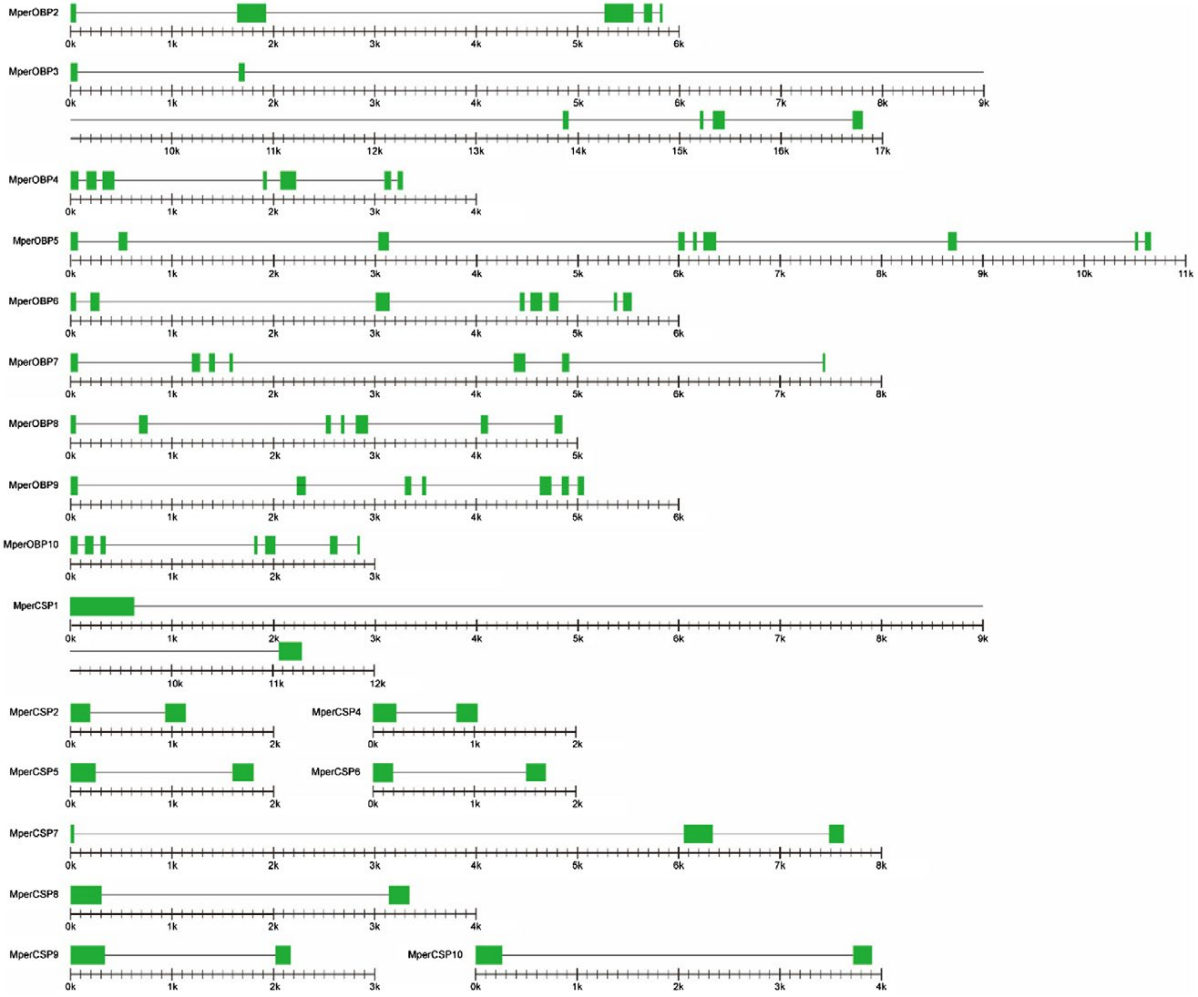
Genomic structure of *Myzus persicae *odorant binding protein (OBP) and chemosensory proteins (CSP) genes. The green rectangles and hairlines between two green rectangles represent the exons and introns, respectively. The length has been shown to scale with a scale bar under each *M. persicae* OBP and CSP gene; every minor mark represents 100 bp. [Colour figure can be viewed at wileyonlinelibrary.com]

The genomic structures and the splice site of the intron–exon junctions of *M. persicae* OBP and CSP genes were analysed based on the *M. persicae* genome annotations and the GT–AG rules (Modrek and Lee, 2002). The results revealed that the sizes of the genomic sequences of MperOBP genes range from 2.85 to 16.81 kb with an average length of 6.92 kb. Five OBP genes (*MperOBP4*/*7*/*8*/*9*/*10*) have six introns and seven exons. The remaining four OBP genes (*MperOBP2*/*3*/*5*/*6*) have 4, 5, 8 and 7 introns and 5, 6, 9 and 8 exons, respectively (Fig. 5Table 5). The intron lengths of each *MperOBP* are variable; for example, the intron lengths of *MperOBP3*, *MperOBP5* and *MperOBP10* are 16.38 kb, 9.98 kb and 2.42 kb, respectively (Table 2). Cross‐species comparison reveals that the numbers of introns and exons of each OBP subgroup are the same among four different aphid species (*M. persicae*, *A. gossypii*, *A. pisum* and *A. glycines*), but the lengths of the introns and exons are different among the four species (Table 2).

**Table 2:** The introns and exons of aphid odorant binding protein (OBP) and chemosensory protein (CSP) genes in *Myzus persicae*, *Aphis gossypii*, *Acyrthosiphon pisum* and *Aphis glycines*

Gene name	Aphid species	Genomic DNA size (bp)	No. of introns	Total length of introns (bp)	Average intron size (bp)	No. of exons	Total length of exons (bp)	Average exon size (bp)	Scaffold	Strand
*OBP2*	*M. persicae*	5 836	4	5 104	1 276	5	732	146	105	−
	*A. gossypii*	3 277	4	2 545	636	5	732	146	x	x
	*A. pisum*	3 885	4	3 153	788	5	732	146	748	+
	*A. glycines*	3 347	4	2 615	654	5	732	146	23	+
*OBP3*	*M. persicae*	16 810	5	16 384	3 277	6	426	71	21	+
	*A. gossypii*	17 385	5	16 959	3 392	6	426	71	x	x
	*A. pisum*	17 976	5	17 550	3 510	6	426	71	195	−
	*A. glycines*	14 751	5	14 325	2 865	6	426	71	455	−
*OBP4*	*M. persicae*	3 269	6	2 669	445	7	600	86	300	+
	*A. gossypii*	4 108	6	3 511	585	7	597	85	x	x
	*A. pisum*	3 250	6	2 668	445	7	582	83	116	+
	*A. glycines*	3 854	6	3 257	543	7	597	85	426	+
*OBP5*	*M. persicae*	10 647	8	9 981	1 248	9	666	74	4	−
	*A. gossypii*	12 286	8	11 611	1 451	9	675	75	x	x
	*A. pisum*	10 371	8	9 705	1 213	9	666	74	838	+
	*A. glycines*	10 483	8	9 808	1 226	9	675	75	449	+
*OBP6*	*M. persicae*	5 530	7	4 882	697	8	648	81	77	−
	*A. gossypii*	5 345	7	4 697	671	8	648	81	x	x
	*A. pisum*	5 349	7	4 701	672	8	648	81	211	−
	*A. glycines*	3 349	7	2 701	386	8	648	81	272	−
*OBP7*	*M. persicae*	7 442	6	6 992	1 165	7	450	64	21	+
	*A. gossypii*	12 546	6	12 099	2 017	7	447	64	x	x
	*A. pisum*	8 526	6	8 058	1 343	7	468	67	195	−
	*A. glycines*	10 771	6	10 306	1 718	7	465	66	455	−
*OBP8*	*M. persicae*	4 849	6	4 363	727	7	486	69	21	+
	*A. gossypii*	7 317	6	6 831	1 139	7	486	69	x	x
	*A. pisum*	6 425	6	5 939	990	7	486	69	195	−
	*A. glycines*	6 285	6	5 799	967	7	486	69	455	−
*OBP9*	*M. persicae*	5 063	6	4 562	760	7	501	72	422	+
	*A. gossypii*	10 033	6	9 532	1 589	7	501	72	x	x
	*A. pisum*	5 803	6	5 302	884	7	501	72	754	−
	*A. glycines*	7 534	6	7 033	1 172	7	501	72	753	−
*OBP10*	*M. persicae*	2 851	6	2 419	403	7	432	62	47	−
	*A. gossypii*	6 429	6	5 985	998	7	444	63	x	x
	*A. pisum*	2 839	6	2 404	401	7	435	62	244	+
	*A. glycines*	3 906	6	3 462	577	7	444	63	Contig_3	+
*CSP1*	*M. persicae*	11 282	1	10 424	10 424	2	858	429	112	−
	*A. gossypii*	6 763	1	6 250	6 250	2	513	257	x	x
	*A. pisum*	9 417	1	8 733	8 733	2	684	342	630	−
	*A. glycines*	6 077	1	5 564	5 564	2	513	257	331	−
*CSP2*	*M. persicae*	1 136	1	740	740	2	396	198	55	−
	*A. gossypii*	1 110	1	705	705	2	405	203	x	x
	*A. pisum*	1 131	1	735	735	2	396	198	447	+
	*A. glycines*	1 118	1	713	713	2	405	203	391	+
*CSP4*	*M. persicae*	1 033	1	595	595	2	438	219	112	−
	*A. gossypii*	1 024	1	586	586	2	438	219	x	x
	*A. pisum*	1 018	1	586	586	2	432	216	630	−
	*A. glycines*	1 017	1	579	579	2	438	219	331	−
*CSP5*	*M. persicae*	1 770	1	1 350	1 350	2	420	210	10	−
	*A. gossypii*	1 770	1	1 350	1 350	2	420	210	x	x
	*A. pisum*	1 792	1	1 378	1 378	2	414	207	152	−
	*A. glycines*	1 771	1	1 351	1 351	2	420	210	192	+
*CSP6*	*M. persicae*	1 704	1	1 308	1 308	2	396	198	112	−
	*A. gossypii*	1 818	1	1 422	1 422	2	396	198	x	x
	*A. pisum*	2 075	1	1 679	1 679	2	396	198	630	−
	*A. glycines*	1 802	1	1 406	1 406	2	396	198	331	−
*CSP7*	*M. persicae*	8 096	2	7 628	3 814	3	468	156	910	−
	*A. gossypii*	7 760	2	7 301	3 651	3	459	153	x	x
	*A. pisum*	7 846	2	7 378	3 689	3	468	156	145	−
	*A. glycines*	7 718	2	7 259	3 630	3	459	153	1910	−
*CSP8*	*M. persicae*	3 324	1	2 832	2 832	2	492	246	10	−
	*A. gossypii*	4 586	1	4 097	4 097	2	489	245	x	x
	*A. pisum*	5 252	1	4 760	4 760	2	492	246	152	−
	*A. glycines*	5 061	1	4 572	4 572	2	489	245	192	+
*CSP9*	*M. persicae*	2 170	1	1 681	1 681	2	489	245	55	+
	*A. gossypii*	2 474	1	1 958	1 958	2	516	258	x	x
	*A. pisum*	2 532	1	2 034	2 034	2	498	249	447	−
	*A. glycines*	2 061	1	1 548	1 548	2	513	257	391	−
*CSP10*	*M. persicae*	3 910	1	3 457	3 457	2	453	227	420	−
	*A. gossypii*	3701	2	3 251	1 626	3	450	150	x	x
	*A. pisum*	4 916	1	4 463	4 463	2	453	227	145	+
	*A. glycines*	3 811	1	3 355	3 355	2	456	228	550	+

‘x’ indicates the genomic location and transcriptional orientation of *A. gossypii* OBPs and CSPs cannot be investigated due to the *A. gossypii* genome not being available.

Eight of the nine *MperCSPs* (*MperCSP1*/*2*/*4*/*5*/*6*/*8*/*9*/*10*) have only one intron, and *MperCSP7* has two introns (Fig. 5Table 5). The lengths of MperCSP genes are much shorter than those of MperOBP genes, ranging from 1.03 to 11.28 kb with an average length of 3.83 kb. All CSPs in the CSP subgroups (*CSP1*/*2*/*4*/*5*/*6*/*8*/*9*) from four different aphid species (*M. persicae*, *A. gossypii*, *A. pisum* and *A. glycines*) have one intron and two exons. The members in the *CSP10* subgroup of *M. persicae*, *A. pisum* and *A. glycines* have only one intron, but *A. gossypii* CSP10 has two introns (Table 2, Zhou *et al*., 2010; Gu *et al*., 2013). These results suggest the formation of the intron–exon junctions in aphid CSP genes occurred at the early stages of aphid evolution before speciation.

**Figure 6:**
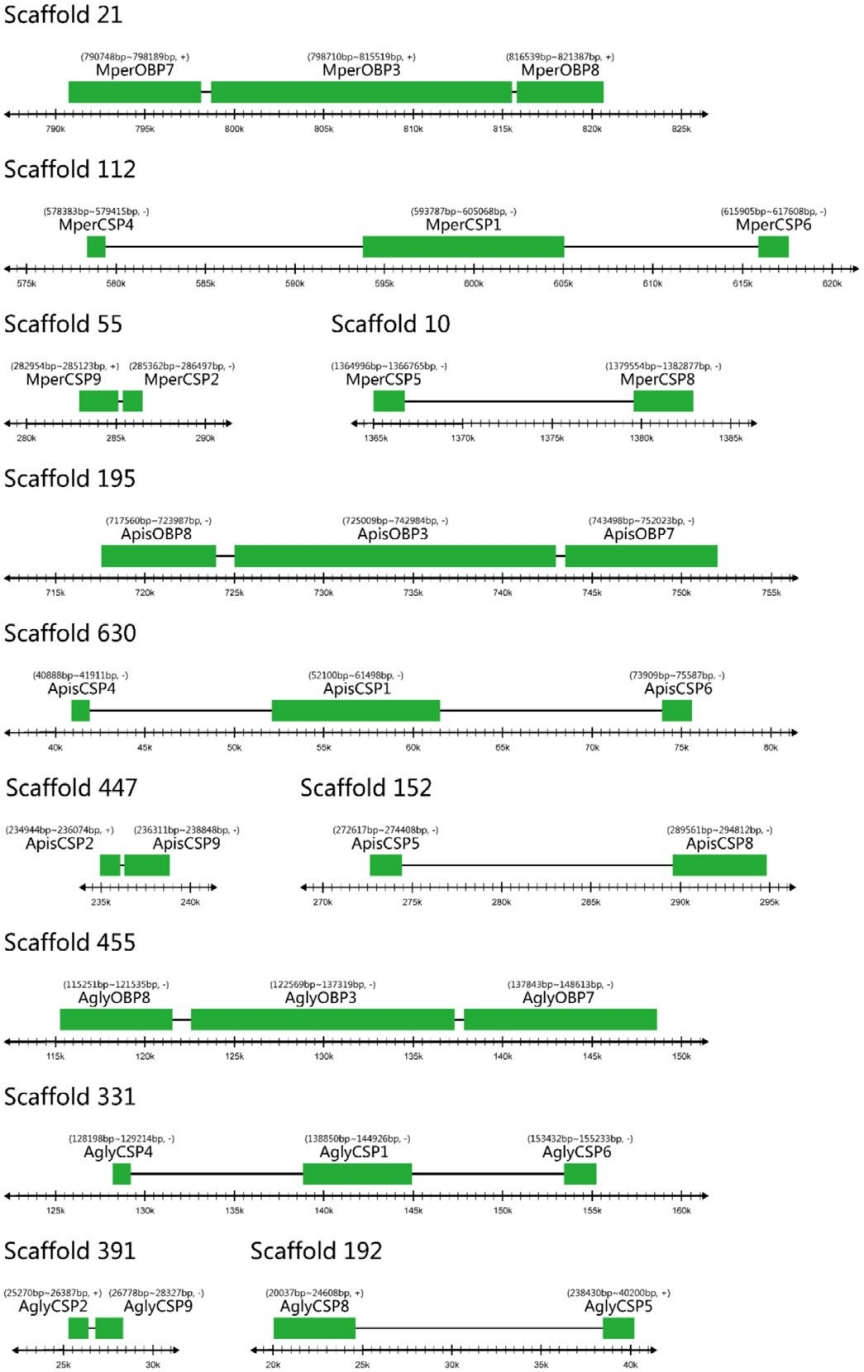
The tandem arrays of odorant binding protein (OBP) and chemosensory protein (CSP) genes in *Myzus persicae*, *Acyrthosiphon pisum *and *Aphis glycines*. The genomic sequences of the *A. pisum*, *M. persicae* and *A. glycines* were downloaded from the AphidBase at the BioInformatics Platform for Agroecosystem Arthropods (https://bipaa.genouest.org/is/). [Colour figure can be viewed at wileyonlinelibrary.com]

We also analysed the genomic clustering of *M. persicae* *OBPs* and *CSPs*. The results indicated that the nine *M. persicae* *OBPs* are distributed among seven scaffolds (scaffolds 4/21/47/77/105/300/422) (Table 1), with *MperOBP3*/*7*/*8* collocated in scaffold 21 (Fig. 6). The *M. persicae* *CSPs* are distributed across five scaffolds (scaffolds 10/55/112/420/910), with *MperCSP1*/*4*/*6* collocated in scaffold 112, *MperCSP2*/*9* located in scaffold 55, and *MperCSP5*/*8* located in scaffold 10 (Table 1Fig. 1). Similar clusters of *OBPs* and *CSPs* were also found in *A. pisum* and *A. glycines* to form the OBP3/7/8 cluster, CSP1/4/6 cluster, CSP2/9 cluster and CSP5/8 cluster (Fig. 6). The phenomenon of gene cluster arrangement can also be found in other insects, such as in *Bombyx mori*, *Drosophila melanogaster* and *Apis mellifera* (Hekmat‐Scafe *et al*., 2002; Xu *et al*., 2003; Forêt and Maleszka, 2006; Gong *et al*., 2007, 2009 ).

### Motif pattern analysis

**Figure 7:**
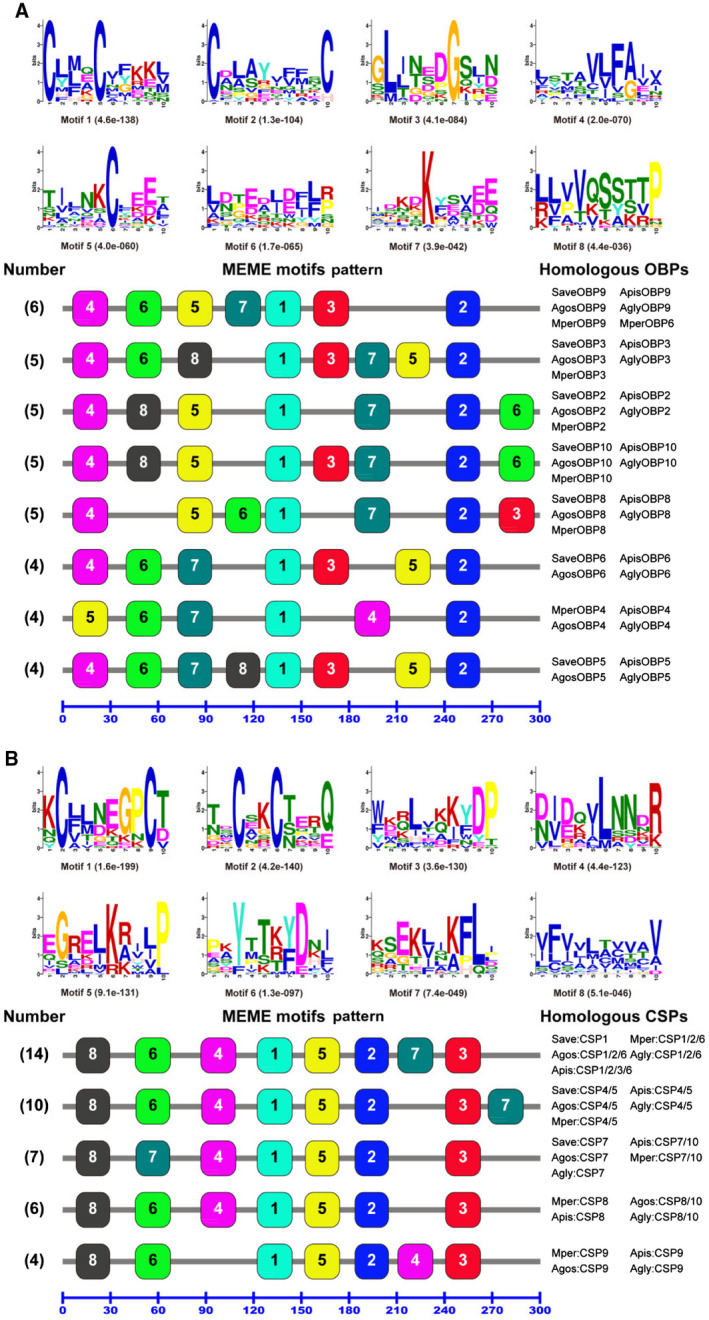
Motif analysis of odorant binding proteins (OBPs) and chemosensory proteins (CSPs) from five aphid species. The parameters used for motif discovery were minimum width = 6, maximum width = 10 and maximum number of motifs to find = 8. The upper parts in (A) and (B) list the eight motifs discovered in the five aphids’ OBPs and CSPs, respectively. All the motifs were discovered using meme (version 4.11.4; Bailey *et al*., 2009) online server (https://meme-suite.org/). The lower parts indicate approximate locations of each motif on the protein sequence. The numbers in the boxes correspond to the numbered motifs in the upper part of the figure, where small numbers indicate high conservation. The numbers on the bottom show the approximate locations of each motif on the protein sequence, starting from the N‐terminus. (A) The eight most common motif patterns which presented in 38 OBPs, with each motif pattern present in more than three OBPs; the remaining seven OBPs had five different motif patterns, with each of them presented in less than four OBPs. (B) All five motif patterns which presented in 41 CSPs, and each motif pattern presented in more than three CSPs. The protein names and sequences of the 45 OBPs and 41 CSPs from five different aphid species are listed in Supporting Information Table S2. [Colour figure can be viewed at wileyonlinelibrary.com]

The conserved motifs are important elements of functional domains. In order to compare the amino acid motif patterns of aphid OBPs and CSPs, a total of 45 OBPs and 41 CSPs with intact ORFs from five aphid species (*M. persicae*, *A. gossypii*, *A. pisum*, *A. glycines* and *S. avenae*) were combined into one set of sequences as a fasta format file and then submitted to the meme server (https://meme-suite.org/) to discover the conserved motifs within each aphid species and subgroups (Fig. 7). The meme program identified eight most conserved motifs (named motifs 1–8) from the 45 OBPs (Fig. 7A) and 41 CSPs (Fig. 7B). Based on the number, position and identity of motifs in the 45 OBPs, 13 motif patterns were identified in these 45 aphid OBP sequences. The most common eight motif patterns (with each motif pattern present in more than three OBPs) are shown as motifs 1/2/3/4/5/6/7/8 and their relative arrangements along each protein sequence are shown in Fig. 7A. Notably, all the paralogous OBPs present in a given aphid species have a unique motif pattern, while the homologous OBPs in each OBP subgroup from different aphid species often share one common motif pattern. The homologous OBP2, OBP3, OBP8, OBP9 and OBP10 subgroups, in the five aphid species *M. persicae*, *A. gossypii*, *A. pisum*, *A. glycines* and *S. avenae*, display 4‐8‐5‐1‐7‐2‐6, 4‐6‐8‐1‐3‐7‐5‐2, 4‐5‐6‐1‐7‐2‐3, 4‐6‐5‐7‐1‐3‐2 and 4‐8‐5‐1‐3‐7‐2‐6 motif patterns, respectively. The OBP5 and OBP6 subgroups, in the four aphid species *A. gossypii*, *A. pisum*, *A. glycines* and *S. avenae*, have motif patterns of 4‐6‐7‐8‐1‐3‐5‐2 and 4‐6‐7‐1‐3‐5‐2, respectively. The OBP4 subgroup in the four aphid species *M. persicae*, *A. gossypii*, *A. pisum* and *A. glycines* has a motif pattern of 5‐6‐7‐1‐4‐2. Furthermore, 38 out of 45 aphid OBPs contain motifs 1, 2, 4, 5, 6 and 7. Motifs 1 and 2 are always located in the middle and at the C‐terminus in all 45 aphid OBPs sequences (Fig. 7A).

The motif patterns of the aphid CSPs are much more conserved than the aphid OBPs, since there are only 5 motif patterns in the 41 aphid CSPs (Fig. 7B). Unlike OBP subgroups, paralogous CSPs in a given aphid species and different CSP subgroups may share a common motif pattern. For example, 14 CSPs in the CSP1, CSP2 and CSP6 subgroups (SaveCSP1, MperCSP1/2/6, AgosCSP1/2/6, AglyCSP1/2/6 and ApisCSP1/2/3/6) have the same motif pattern of 8‐6‐4‐1‐5‐2‐7‐3. Ten CSPs in the CSP4 and CSP5 subgroups (SaveCSP4/5, ApisCSP4/5, AgosCSP4/5, AglyCSP4/5 and MperCSP4/5) exhibit a motif pattern of 8‐6‐4‐1‐5‐2‐3‐7. ApisCSP10 and MperCSP10 in the CSP10 subgroup share the same motif pattern (8‐7‐4‐1‐5‐2‐3) with SaveCSP7, ApisCSP7, AgosCSP7, MperCSP7 and AglyCSP7 in the CSP7 subgroup. Similarly, two CSPs (AgosCSP10 and AglyCSP10) share one motif pattern (8‐6‐4‐1‐5‐2‐3) with four CSPs in the CSP8 subgroup (MperCSP8, AgosCSP8, ApisCSP8 and AglyCSP8). The four CSPs in the CSP9 subgroup (MperCSP9, ApisCSP9, AgosCSP9 and AglyCSP9), however, have a subgroup‐specific motif pattern of 8‐6‐1‐5‐2‐4‐3 (Fig. 7B). Meanwhile, motifs 1, 2, 3, 4, 5 and 8 are located at the same positions in all 41 aphid CSPs, except for the four CSP9 subgroup genes, in which motif 4 is located at the C‐terminus (Fig. 7B).

**Figure 8:**
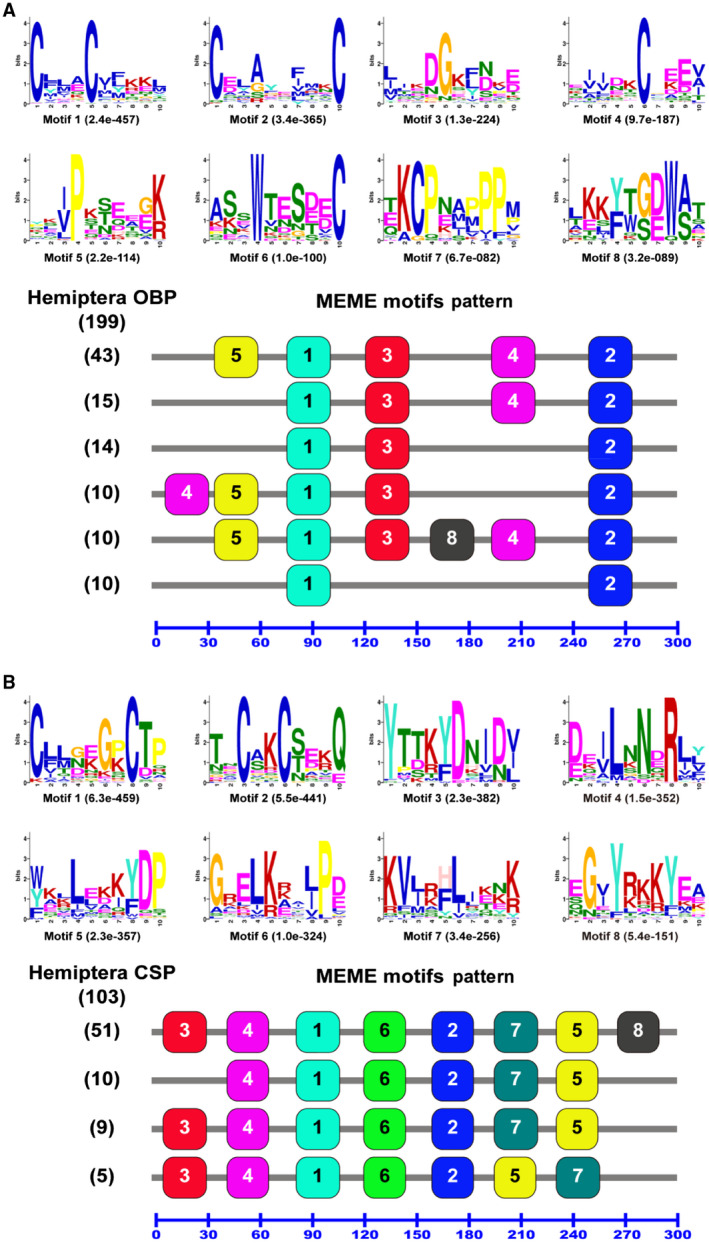
Motif analysis of Hemiptera odorant binding proteins (OBPs) and chemosensory proteins (CSPs). Parameters used for motif discovery were minimum width = 6, maximum width = 10 and maximum number of motif to find = 8. The upper parts in (A) and (B) list the eight motifs discovered in the Hemiptera OBPs and CSPs, respectively. All the motifs were discovered using meme (version 4.11.4; Bailey *et al*., 2009) online server (https://meme-suite.org/). The lower parts indicate approximate locations of each motif on the protein sequence. The numbers in the boxes correspond to the numbered motifs in the upper part of the figure, where small numbers indicate high conservation. The numbers on the bottom show the approximate locations of each motif on the protein sequence, starting from the N‐terminus. (A) The most common six motif patterns which presented in 102 OBPs, with each motif pattern present in more than nine OBPs; the remaining 97 OBPs had 45 different motif patterns, with each of them presented in less than 10 OBPs. (B) The four most common motif patterns which presented in 75 CSPs, with each motif pattern present in more than four CSPs; the remaining 28 CSPs had 13 different motif patterns, with each of them presented in less than five CSPs. The protein names and sequences of the 199 OBPs and 103 CSPs from 18 different Hemiptera species are listed in Supporting Information Table S3. [Colour figure can be viewed at wileyonlinelibrary.com]

When the motif pattern of the 199 OBPs from 18 Hemiptera species were compared, we found a total of 51 different motif patterns (data not shown), but 51.3% of the 199 OBPs (102) are affiliated to the top six common motif patterns (Fig. 8A). These include 43 OBPs from the 5‐1‐3‐4‐2 pattern, 15 OBPs from the 1‐3‐4‐2 pattern, 14 OBPs from the 1‐3‐2 pattern, 10 OBPs from the 4‐5‐1‐3‐2 pattern, 10 OBPs from the 5‐1‐3‐8‐4‐2 pattern and another 10 OBPs from the 1‐2 pattern (Fig. 8A).

Motifs 1 and 2 that contain four highly conserved cysteine residues (C2 and C3 in motif 1, C5 and C6 in motif 2) of the classic OBP subfamily in insects (Zhou *et al*., 2004, 2010 ) are present in all the 102 Hemiptera OBPs (Fig. 8A). Motif 3 with highly conserved glycine (G) and aspartic acid (D) is present in the top five common motif patterns (92 of the 102 OBPs). Motif 4, which contains another characteristically conserved cysteine residue (C4), was only mapped to some OBPs. However, motif 6, containing highly conserved tryptophan (W) and conserved cysteine 4 (C6a), and motif 7, containing highly conserved lysine (K), C6b and proline (P) of the plus‐C OBP subfamily, were not present in the 102 OBPs (Fig. 8A).

Motif analysis of a total of 103 Hemiptera CSPs revealed that 75 out of the 103 CSPs (72.8%) belong to the top four common motif patterns (Fig. 8B). These include 51 CSPs from the 3‐4‐1‐6‐2‐7‐5‐8 pattern, 10 CSPs from the 4‐1‐6‐2‐7‐5 pattern, 9 CSPs from the 3‐4‐1‐6‐2‐7‐5 pattern and 5 CSPs from the 3‐4‐1‐6‐2‐5‐7 pattern (Fig. 8B). Ten CSPs have lost motif 3, and 51 CSPs have motif 8. The motif patterns shown in Figs 7 and 8 further demonstrated that insect CSPs are more conversed than OBPs. It should be noticed that the motif patterns discovered by meme in Figs 7 and 8 are not comparable, because different input sequences were used in each analysis.

### *Expression profiles of *M. persicae *OBP and CSP genes in different tissues*

The general and quantitative expression profiles of *M. persicae* OBP and CSP transcripts in different tissues (antennae, heads, legs and decapitated bodies) were examined using both semi‐quantitative reverse transcription PCR (RT‐PCR) and quantitative real‐time PCR (qRT‐PCR). Because equal amounts of cDNA (150 ng) were used in the RT‐PCR reactions, the intensity of the PCR bands of *MperOBP2*/*3*/*6*/*9*/*10* and *MperCSP2*/*4*/*5*/*9* was higher than the remaining *MperOBPs* and *MperCSPs*, which can reflect their relatively high expression levels in *M. persicae*. Actually, the reads per kilobase per million mapped reads (RPKM) analysis also showed their relative high abundances in the *M. persicae* transcriptome, with the RPKM values of *MperOBP2*/*3* and *MperCSP2*/*4*/*5*/*9* more than 100 (Table 1).

**Figure 9:**
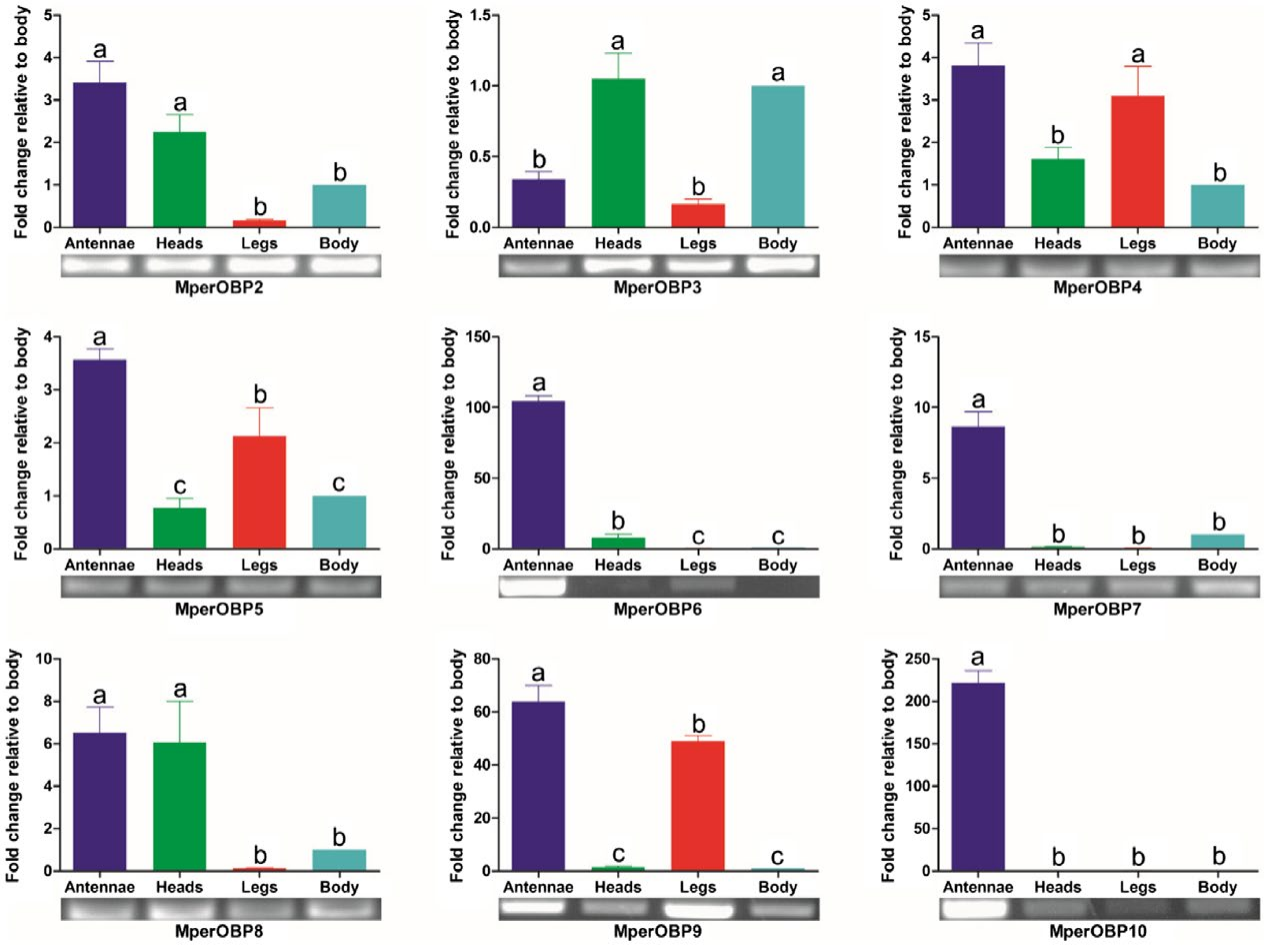
*Myzus persicae* odorant binding protein (OBP) transcript levels in different tissues assessed by reverse transcription PCR and quantitative real‐time PCR. The error bars present the standard error, and the different letters (a, b, c) indicate significant differences (*p* < 0.05). This figure was presented using *GAPDH* as reference gene to normalize the target gene expression and correct sample‐to‐sample variation; similar results were also obtained with *β‐actin* as reference gene. The standard error is represented by the error bar. [Colour figure can be viewed at wileyonlinelibrary.com]

The absolute values of the slope of all lines from template dilution plots (log cDNA dilution vs. Δ*C*_T_) were <0.1 (Supporting Information Figs S2 and S3), and the real PCR amplification efficiencies of target and reference genes were more than 1.90 (calculated using the LinRegPCR program and listed in Supporting Information Table S5). Therefore, the efficiencies of the target and reference genes were similar in our analysis, and the ΔΔ*C*_T_ calculation method can be used for the relative quantification. Both RT‐PCR and qRT‐PCR results showed that three OBP genes (*MperOBP6*/*7*/*10*) were antennae specific, and their expression levels were approximately 101, 8.7 and 222 times higher in the antennae than in the body (*p* < 0.05), respectively (Fig. 9). *MperOBP2*/*4*/*5*/*8*/*9* showed the highest expression in the antennae, and their expression levels were, respectively, 3.5, 3.8, 3.6, 6.5 and 63.9 times greater in the antennae than in the body (*p* < 0.05). *MperOBP2*/*4*/*5*/*8*/*9* were also detectable in other tissues, such as head, leg and body (Fig. 9). *MperOBP3* was the only OBP whose expression level was lower in the antennae than in the head and body.

**Figure 10:**
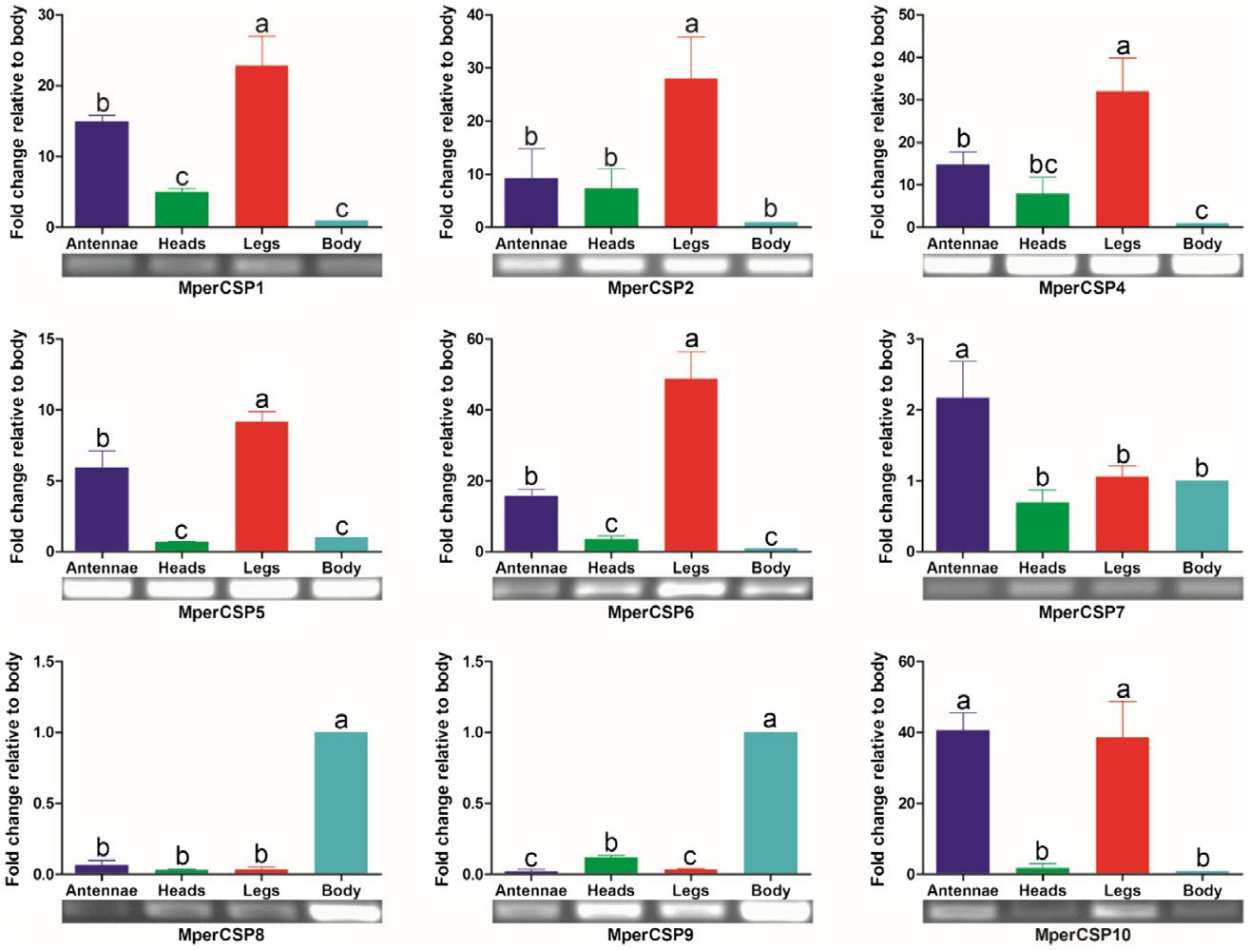
*Myzus persicae* chemosensory protein (CSP) transcript levels in different tissues assessed by reverse transcription PCR and quantitative real‐time PCR. The error bars present the standard error, and the different letters (a, b, c) indicate significant differences (*p* < 0.05). This figure was presented using *GAPDH* as reference gene to normalize the target gene expression and correct sample‐to‐sample variation; similar results were also obtained with *β‐actin* as reference gene. The standard error is represented by the error bar. [Colour figure can be viewed at wileyonlinelibrary.com]

None of the nine *MperCSPs* were found to be antennae specific, and five of them (*MperCSP1*/*2*/*4*/*5*/*6*) expressed at a higher expression level in the legs than the other three tissues (antennae, head and body) (Fig. 10), with the expression levels 20–50 times higher in the legs than in the decapitated bodies (*p* < 0.05). *MperCSP10* expressed 41 and 39 times higher in the antenna and legs, respectively, than in the body (*p* < 0.05). *MperCSP8* and *MperCSP9* mainly expressed in the body. *MperCSP7* exhibited a 2.2 times higher expression in the antennae than in the body (*p* < 0.05) (Fig. 10).

## Discussion

With the increase in insect genome projects and transcriptome sequencing projects, particularly in recent years, very large numbers of both *OBPs* and *CSPs* have been identified in different insect species. These studies have provided an excellent chance to comparatively study insect *OBPs* and *CSPs*. For the first time, we had identified nine *OBPs* and nine *CSPs*, both from the *M. persicae* transcriptomic and genomic data, including four *OBPs* and five *CSPs* reported from the expressed sequence tags in *M. persicae* (Xu *et al*., 2009). The number of *M. persicae* *OBPs* and *CSPs* identified here is less than the number of *OBPs* and *CSPs* identified in *A. pisum* (15 *OBPs*, 13 *CSPs*) (Zhou *et al*., 2010), the same as in *A. gossypii* (nine *OBPs*, nine *CSPs*) (Gu *et al*., 2013), and similar to those of other sucking insects such as the plant hoppers *N. lugens* (10 *OBPs*, 11 *CSPs*) (Zhou *et al*., 2014), *S. furcifera* (12 *OBPs*, nine *CSPs*) (He and He, 2014; Zhou *et al*., 2015) and the plant bug *A. suturalis* (16 *OBPs*, eight *CSPs*) (Cui *et al*., 2017). The high conservation in the sequence identities and genomic structures of the OBP and CSP orthologues among aphid species indicates that aphid OBPs and CSPs are conserved in a single copy across all aphids (with occasional losses), indicating that each OBP and CSP class evolved from a single gene in the common ancestor of aphids without subsequent duplication.

The absence of OBP1 and CSP3 homologous genes in *M. persicae*, as demonstrated in this study by transcriptome sequencing, searching the genome database and molecular cloning using gene‐specific primers of *A. pisum* *OBP1* and *CSP3* (data not shown here), is consistent with the same results reported in the cotton aphid *A. gossypii* (Gu *et al*., 2013) and recently in the *A. glycines* genome (Wenger *et al*., 2017). However, OBP1 homologous genes have been found in six other aphid species (*A. pisum*, *S. avenae*, *M. viciae*, *M. dirhodum*, *T. salignu* and *P. salicis*). The OBP1 orthologous genes are also not found in the plant hoppers and plant bugs in this study. It is possible that OBP1 and CSP3 genes might have been lost after aphid speciation of *M. persicae*, *A. gossypii* and *A. glycines.* Furthermore, there is a high amino acid identity between OBP1 and OBP8 subgroups in aphids, consistent with previous reports (Gu *et al*., 2013; He and He, 2014; Yuan *et al*., 2015; Xue *et al*., 2016).

The phylogenetic analyses clearly divided most of the aphid OBPs and CSPs into species‐specific homology subgroups. However, some aphid OBP and CSP subgroups are clustered with non‐aphid OBP and CSP genes in conserved clades. For example, aphid OBP4 and OBP5 subgroups are clustered with OBPs of plant hopper and plant bug with a bootstrap support of 100 and 84, respectively (Fig. 3), and aphid CSP7 and CSP9 subgroups are clustered with plant hopper and plant bug CSPs with a bootstrap support of 73 (Fig. 4), suggesting these genes may have a common function in these sucking insects.

The motif analysis contributes to the understanding of the functional domains of insect OBPs and CSPs. The members of each OBP subgroup have a common motif pattern, particularly for members of the aphid OBP family and members of the CSP9 subgroup (Fig. 7). Five motif patterns are shared by different CSP subgroups, further supporting previous reports of greater conservation between insect CSPs than between OBPs (Fig. 7). Interestingly, *A. pisum* ApisCSP3 shares the same motif pattern arrangement with members of the aphid CSP1 and CSP6 subgroups as well as ApisCSP2, despite the sequences between these three subgroups (CSP1, CSP2 and CSP6) being very different (Fig. 4). This result is not consistent with a previous report on the motif analysis of Hemiptera OBPs and CSPs (Wang *et al*., 2017). This could be because different sets of OBP and CSP protein sequences were used in these two studies. The comparative sequence analyses with other Hemiptera species confirmed the results from the previous studies that CSPs are more conserved than OBPs and some highly conserved cysteine residues (C2, C3, C5 and C6) in insect OBPs (Fig. 8), suggesting their involvement in protein structure and possible functions.

The intron numbers and lengths of *M. persicae* *OBPs* are nearly equal to other orthologous genes in *A. pisum* and *A. gossypii* (Zhou *et al*., 2010; Gu *et al*., 2013), but much greater than those in aphid CSPs; this appears to be a unique feature of aphid OBP genes in general. Four gene clusters (*OBP3*/*7*/*8*, *CSP1*/*4*/*6*, *CSP2*/*9* and *CSP5*/*8*) are found in three aphid species (Fig. 6). The conserved genomic structure and same transcriptional orientation demonstrate that these aphid OBP and CSP genes were created by gene duplication, possibly through chromosome recombination, supporting the birth‐and‐death evolutionary model (Nei and Rooney, 2005; Zhou *et al*., 2010).

The major alarm pheromone component *E*βf emitted from the cornicles of aphids (Edwards *et al*., 1973) is recognized by large placoid sensillum neurons, which express *OR5* on the sixth antennal segment in *A. pisum* (Zhang *et al*., 2017); *OBP3* and *OBP7* are both expressed in the large placoid sensilla in *M. persicae* (Sun *et al*., 2013) and in the type II trichoid sensilla in *A. pisum* in the antennae (Biasio *et al*., 2015). OBP3 and/or OBP7 proteins in several aphid species (*A. pisum*, *S. avenae*, *M. viciae*, *N. ribisnigri* and *R. padi*) have high binding affinities with *E*βf (Qiao *et al*., 2009; Vandermoten *et al*., 2011; Zhong *et al*., 2012; Fan *et al*., 2017), but the cellular location of all *M. persicae* OBPs in the antennal sensilla and their binding abilities with *E*βf are still unknown. Our data reveal that *MperOBP7* is antennae specific and expressed at a level that is 8.7 times higher in the antennae than in the body (*p* < 0.05) (Fig. 9), consistent with previous reports of the high binding affinity of this OBP to *E*βf (Qiao *et al*., 2009; Vandermoten *et al*., 2011; Zhong *et al*., 2012; Fan *et al*., 2017) and its localization in antennal sensilla (Sun *et al*., 2013; Biasio *et al*., 2015). However, OBP3, which was also reported as the *E*βf binding protein in several aphid species (*A. pisum*, *S. avenae*, *M. viciae*, *N. ribisnigri* and *R. padi*), is neither antennae specific nor antennae enriched, but is highly expressed in the heads and body in *M. persicae* (Fig. 9). This expression pattern is similar to that of *A. pisum* OBP3 (Biasio *et al*., 2015) and of *S. avenae* OBP3 (Xue *et al*., 2016), suggesting that aphid OBP3 proteins may have other functional roles in addition to alarm pheromone sensation. The fact that the *A. pisum* OBP3 was found in the type II trichoid sensilla in the antennae and in the terminal region of the body (Biasio *et al*., 2015) suggests that it may have dual functions of recognizing the *E*βf from the environment and transporting the *E*βf from the cornicles to the external environment.

Insect *CSPs* usually exhibit much broader expression profiles both in chemosensory organs and nonchemosensory organs than *OBPs* do, and play multiple roles in chemoreception, growth and development. For example, the American cockroach (*Periplaneta americana*) CSP gene *P10* has a putative role in leg regeneration (Nomura *et al*., 1992), and the migratory locust (*Locusta migratoria*) antennae‐expressed *CSP* gene *LmigCSP3* can regulate the rapid switch between attraction and repulsion behaviours in this species (Guo *et al*., 2011). In the present study, *M. persicae* *CSPs* also showed much broader expression profiles in nonsensory organs than *M. persicae* *OBPs* do (Fig. 10). None of the nine *MperCSPs* were found to be antennae‐specific. Five of them (*MperCSP1*/*2*/*4*/*5*/*6*) showed a higher expression level in the legs than the other three tissues (antennae, heads and body). *MperCSP10* was mainly expressed in the antennae and legs. The broad and diverse expression patterns of *M. persicae* *CSPs* are consistent with their possible multifunctions in olfactory perception, development and other processes.

The sex pheromones from the Aphididae family are usually released from the tibiae of the hind legs of the mature sexual female aphids (Marsh, 1972; Pickett and Glinwood, 2007), and usually comprise of a mixture of the ubiquitous iridoids (4a*S*,7*S*,7a*R*)‐nepetalactone and (1*R*,4a*S*,7*S*,7a*R*)‐nepetalactol (Pickett *et al*., 1992; Dewhirst *et al*., 2010); however, in order to convey species integrity, the ratio of (4a*S*,7*S*,7a*R*)‐nepetalactone to (1*R*,4a*S*,7*S*,7a*R*)‐nepetalactol is species dependent, and this ratio is 1 : 1.5 for *M. persicae* (Dawson *et al*., 1990). The aphid OBPs and CSPs that can specially recognize the two ubiquitous sex pheromones are still unknown. It is reasonable to assume that the *M. persicae* *OBPs* and *CSPs* which are highly expressed in the antennae and legs may participate in the intersexual communication process. Till now, functional studies of the aphid OBPs and CSPs have narrowly focused on several OBPs (eg OBP3/7); the putative functional roles of other aphid OBPs and all the CSPs in the *M. persicae* and other aphid species still need to be elucidated. Future work towards the functions of these *M. persicae* OBPs and CSPs will enhance our understanding of the ecological context of aphid–aphid and aphid–plant interactions and facilitate design of novel sustainable strategies for aphid control.

## Experimental procedures

### Aphids, sample collection and RNA extraction

*M. persicae* aphids were collected from Chinese cabbage (*Brassica rapa* L. ssp. *pekinensis*) leaves at Langfang Experimental Station of the Chinese Academy of Agricultural Sciences, Hebei Province, China. Apterous adults were reared continuously as a parthenogenetic colony on tobacco (*Nicotiana tabacum* L.) seedlings in chambers, at 18–24 °C, 65–75% relative humidity, and a 16 h : 8 h light : dark photoperiod.

About 2000 apterous aphids were dissected with fine scissors to collect their antennae, heads, legs and decapitated bodies into separate tubes and stored in liquid nitrogen. Total RNAs from these samples were extracted using Trizol reagent (Life Technologies, Carlsbad, CA, USA) following the manufacturer’s protocol. The quantity and integrity of RNA samples were verified using 1.1% agarose gel electrophoresis and a NanoDrop ND‐1000 spectrophotometer (Thermo Scientific, Wilmington, DE, USA).

### cDNA library construction and Illumina sequencing

About 50 mg of apterous aphids was collected and kept in a 1.5 ml centrifuge tube in liquid nitrogen for total RNA extraction using the Trizol reagent. The cDNA libraries were constructed using the NEBNext Ultra RNA Library Prep Kit for Illumina (New England Biolabs, Ipswich, MA, USA) according to the manufacturer’s protocol. Briefly, messenger RNAs (mRNAs) were isolated from 10 µg total RNAs from apterous aphids using oligo (dT) magnetic beads and fragmented into short nucleotides using the fragmentation buffer provided with the kit at 94 °C for 5 min. The cleaved mRNA was transcribed into the first‐strand cDNA using a random hexamer primer and M‐MuLV reverse transcriptase (RNaseH^−^). The second‐strand cDNA was subsequently synthesized using DNA polymerase I, deoxyribonucleotide triphosphate and ribonuclase H. After the end repair, dA‐tailing and adaptor ligation, the products were amplified by PCR and purified using the QIAquick PCR purification kit (Qiagen, Valencia, CA, USA) to create the final sequencing library. The cDNA library was pair‐end sequenced using a PE150 strategy on an Illumina Hiseq2500 platform (Illumina, San Diego, CA, USA).

### Bioinformatics analysis

The raw reads were cleaned by removing adaptor sequences and low‐quality sequences using Q20 with 99% accuracy using trimmomatic software (version 0.32) (Bolger *et al*., 2014). The clean reads were *de novo* assembled into unigenes with the short read assembly program trinity (version 20121005) with default parameters (Grabherr *et al*., 2011). After assembly, homology searches of all unigenes were performed using the blastx and blastn programs against the GenBank nonredundant protein (nr) and nucleotide sequence (nt) databases at the National Center for Biotechnology Information (NCBI). Matches with an *E*‐value less than 1.0 × 10^−5^ were considered to be significant and kept (Anderson and Brass, 1998). Gene names were assigned to each unigene based on the best blastx hit with the highest score value.

The BWA‐MEM alignment algorithm (Li, 2013) and htseq program (version 0.6.1) (Anders *et al*., 2015) were used for aligning the RNA reads and counting the read numbers mapped to each gene, respectively. The abundances of the unigenes in the transcriptome were calculated by the RPKM method (Mortazavi *et al*., 2008), using the formula

$$\text{RPKM(A)}=\frac{1\;000\;000\times C\times 1000}{N\times L}$$

where RPKM(A) is the expression abundance of gene A, *C* is the number of reads that are uniquely mapped to gene A, *N* is the total number of reads that are uniquely mapped to all genes and *L* is the number of bases on gene A. The RPKM method is able to eliminate the influence of different gene lengths and sequencing discrepancies on the calculation of transcript abundance.

### *Annotation of putative OBP and CSP genes in *M. persicae *transcriptome and genome*

The genomic sequences of *M. persicae* clone G006 assembly v2 were downloaded from AphidBase at the BioInformatics Platform for Agroecosystem Arthropods (https://bipaa.genouest.org/is/aphidbase/myzus_persicae/). Two methods were used to identify unigenes encoding putative *OBPs* and *CSPs* in the *M. persicae* transcriptome and genome. First, we ran the OBP motifsearch program on C1‐X_15–39_‐C2‐X_3_‐C3‐X_21–44_‐C4‐X_7–12_‐C5‐X_8_‐C6 (Zhou *et al*., 2008) and the CSP motifsearch program on C_1_‐X_6‐8_‐C_2_‐X_16‐21_‐C_3_‐X_2_‐C_4_ (Zhou *et al*., 2006) to identify the respective putative OBP and CSP genes from the *M. persicae* transcriptomic and genomic databases obtained earlier. Second, a tblastn search with known OBP and CSP sequences from other aphid species as the ‘query’ was performed to identify OBPs and CSPs from both the transcriptomic and genomic datasets. All candidate OBP and CSP genes were manually checked using the blastx program available at the NCBI.

### Verification of OBP and CSP sequences by cloning and sequencing

ORFs of each identified OBP and CSP sequence were found by orf finder graphical analysis at NCBI (https://www.ncbi.nlm.nih.gov/gorf/gorf.html). Then, gene‐specific primers (Supporting Information Table S1) were designed using primer 5.0 software to clone the ORF of each *M. persicae* OBP and CSP gene. The template cDNA was synthesized using the Fast Quant RT Kit (TianGen, Beijing, China). PCR reactions were carried out with 200 ng antennal cDNAs with 0.5 units of *Ex Taq* DNA polymerase (TaKaRa, Dalian, China). The PCR amplification conditions were set as 95 °C for 2 min, followed by 36 cycles of 94 °C for 45 s, 56 °C for 1 min, 72 °C for 1 min and a final extension at 72 °C for 10 min. The PCR products were gel‐purified and subcloned into the pGEM‐T Easy vector (Promega, Madison, WI, USA). The inserts were confirmed by sequencing on the ABI3730XL automated sequencer (Applied Biosystems, Carlsbad, CA, USA) with standard M13 primers.

### *Genomic structure analysis of *M. persicae *OBPs and CSPs*

The genomic DNA sequences of each *M. persicae* OBP and CSP gene were extracted using the blastn program, with the mRNA sequences of *M. persicae* OBPs and CSPs as ‘query’. The mRNA‐to‐genomic DNA alignment of each OBP and CSP gene was analysed using the splign online program (https://www.ncbi.nlm.nih.gov/sutils/splign/splign.cgi).

### Motif analysis

A total of 45 OBPs and 41 CSPs from 5 different aphid species (*M*. *persicae*, *A. gossypii*, *A. pisum*, *A. glycines* and *S. avenae*) (Supporting Information Table S2) were used for motif discovery and pattern analysis. A total of 199 OBPs and 103 CSPs from 18 different Hemiptera species (Supporting Information Table S3) were used for comparing the motif patterns between Hemiptera OBPs and CSPs. All OBP and CSP sequences used in this study have intact ORFs and similar lengths. The motif‐based sequence analysis tool meme (Bailey *et al*., 2009) (version 4.9.1; https://meme-suite.org/), which has been widely used for the discovery of DNA and protein motifs, was used to discover and analyse the motifs in the OBP and CSP sequences. The parameters used for motif discovery were as follows: minimum width = 6, maximum width = 10 and the maximum number of motifs to find = 8. This program analyses any multiple input sequences and predicts motifs and their arrangements along an individual sequence. The outputs are two graphic presentations: one contains the similarity plots for each identified motif and another contains a linear arrangement of each predicted motif on the sequences.

### Sequence and phylogenetic analysis

The putative signal peptides of derived *M. persicae* OBP and CSP proteins were predicted using the SignalIP 4.1 Server (https://www.cbs.dtu.dk/services/SignalP/) (Petersen *et al*., 2011). The percentage identity matrix of each pair of OBPs was calculated using Vector NTI 11.5 (Invitrogen Corporation, Carlsbad, USA). Owing to the wide divergence of the 237 OBPs (12.63% amino acid identity) and 110 CSPs (16.48% amino acid identity) used in this study, sequence alignments were conducted with the prank alignment program (Löytynoja and Goldman, 2010), phylogenetic trees were established based on maximum likelihood by raxml version 8 (Stamatakis, 2014) with LG substitution matrix selected by the prottest 3 program (Darriba *et al*., 2011). Bootstrapping was performed to compute the confidence of the branches using 1000 replicates.

### Expression profiles of M. persicae OBP and CSP genes in different tissues

The cDNAs from aphid antennae, heads, legs and the body parts were synthesized using the PrimeScript RT Reagent with gDNA Eraser (TaKaRa, Dalian, China). An equal amount of cDNA (150 ng) was used as the RT‐PCR and qRT‐PCR templates. Gene‐specific primer pairs for RT‐PCR analyses were designed with Primer 3 (https://frodo.wi.mit.edu/) or Primer Premier 5 (see Supporting Information Table S4). *β‐Actin* (GenBank Acc. XM_022309797) of *M. persicae* was used as the control gene to test the integrity of the cDNA. The PCR cycling profile was: 95 °C for 2 min, followed by 35 cycles of 95 °C for 30 s, 56 °C for 45 s, 72 °C for 1 min and a final extension for 10 min at 72 °C. The PCR products were separated on 1.2% agarose gels and stained with ethidium bromide. Each reaction was done at least six times with three biological replicates. In addition, the PCR products were randomly selected and verified by DNA sequencing.

qRT‐PCR was carried out on an ABI 7500 Real‐Time PCR system (Applied Biosystems, Carlsbad, CA, USA). The *β‐actin* and *GAPDH* (GenBank Acc. XM_022315441) genes are stably expressed in different tissues and developmental stages (Supporting Information Fig. S1), so were used as reference genes for normalizing the target gene expression and correcting the sample‐to‐sample variations. The primers used for qRT‐PCR were designed with beacon designer 7.90 (Supporting Information Table S5). Each qRT‐PCR reaction was conducted in a 25‐µl reaction mixture containing 12.5 µl of SuperReal PreMix Plus (TianGen, Beijing, China), 0.75 µl of each primer (10 µm), 1 µl of sample cDNA (150 ng/µl), 0.5 µl of Rox Reference Dye and 9.5 µl of sterilized water. The qRT‐PCR cycling parameters were 95 °C for 15 min, followed by 40 cycles of 95 °C for 10 s and 60 °C for 32 s. Then, the PCR products were heated to 95 °C for 15 s, cooled to 60 °C for 1 min, heated again to 95 °C for 30 s and cooled to 60 °C for 15 s to measure the melt curves. Negative controls without any template were included in each experiment. To ensure reproducibility, each qRT‐PCR reaction for each sample was performed in three technical replicates and three biological replicates. The comparative $2^{-\Delta \Delta C_{\text{T}}}$ method (Livak and Schmittgen, 2001) was used to calculate the relative expressions between tissues. The comparative analyses of each target gene among various tissues were determined using a one‐way nested analysis of variance, followed by Tukey’s honest significance difference test using the spss statistics 18.0 software (SPSS Inc., Chicago, IL, USA). When applicable, the values were presented as the mean plus/minus the standard error.

An assumption in the ΔΔC_T_ calculation for the comparative $2^{-\Delta \Delta C_{\text{T}}}$ method for quantification is that the amplification efficiencies of the target and reference genes are approximately equal (Livak and Schmittgen, 2001). To confirm this, a pilot experiment was conducted to examine the variation of Δ*C*_T_ (*C*_T, Target_ − *C*_T, β–actin/GAPDH_) with template dilution. Briefly, five serial fivefold dilutions of cDNA from each sample were amplified. For each dilution, amplifications were performed in triplicate using primers for the target gene and *β‐actin* or *GAPDH*. Mean *C*_T_ was calculated for both target gene and *β‐actin* or *GAPDH*, Δ*C*_T_ was calculated, and log cDNA dilution vs. Δ*C*_T_ was plotted. The real PCR amplification efficiencies of target and reference genes were calculated using the linregpcr program (version 11.0) (Ramakers *et al*., 2003).

## Supporting information


**FIGURE S1.** The sTABLE expression of M. persicae β‐Actin and GAPDH in different development stages and tissues measured by RT‐PCR.
**FIGURE S2.** The standard curves of M. persicae OBPs and CSPs with GAPDH as reference gene.
**FIGURE S3.** The standard curves of M. persicae OBPs and CSPs with β‐actin as reference gene.

**TABLE S1.** Gene specific primers used for cloning of M. persicae OBP and CSP genes.
**TABLE S2.** The protein names and sequences of the 45 OBPs and 41 CSPs from M. persicae, A. gossypii, A. pisum, A. glycines and S. avenae used in Fig. 7.
**TABLE S3.** The protein names and sequences of the 199 OBPs and 103 SPs from Hemiptera insects used in Fig. 8.
**TABLE S4.** Primers used in RT‐PCR for determination expression levels of M. persicae OBP and CSP genes.
**TABLE S5.** Primers used in real‐time PCR for determination expression level of M. persicae OBP and CSP genes. The real PCR amplification efficiencies of target and reference genes were calculated using LinRegPCR program (version 11.0) (Ramakers *et al*., 2003).
**TABLE S6.** A percent identity matrix of M. persicae OBPs.
**TABLE S7.** A percent identity matrix of M. persicae CSPs.
**TABLE S8.** The protein names and sequences of the Hemiptera 237 OBPs used in Fig. 3.
**TABLE S9.** The protein names and sequences of the Hemiptera 110 CSPs used in Fig. 4.

## References

[imb12513-bib-0001] Anders S. , Pyl P.T. and Huber W. (2015) HTSeq – a Python framework to work with high‐throughput sequencing data. Bioinformatics, 31, 166–169. 25260700 10.1093/bioinformatics/btu638 PMC4287950

[imb12513-bib-0002] Anderson , I. and Brass , A . (1998) Searching DNA databases for similarities to DNA sequences: when is a match significant? Bioinformatics, 14, 349–356. 9632830 10.1093/bioinformatics/14.4.349

[imb12513-bib-0003] Angeli , S. , Ceron , F. , Scaloni , A. , Monti , M. , Monteforti , G. , Minnocci , A. *et al* (1999) Purification, structural characterization, cloning and immunocytochemical localization of chemoreception proteins from *Schistocerca gregaria* . European Journal of Biochemistry, 262, 745–754. 10411636 10.1046/j.1432-1327.1999.00438.x

[imb12513-bib-0004] Arakaki , N . (1989) Alarm pheromone eliciting attack and escape responses in the sugar cane woolly aphid, *Ceratovacuna lanigera* (Homoptera, Pemphigidae). Journal of Ethology, 7, 83–90.

[imb12513-bib-0005] Bailey , T.L. , Boden , M. , Buske , F.A. , Frith , M. , Grant , C.E. , Clementi , L. *et al* (2009) MEME suite: tools for motif discovery and searching. Nucleic Acids Research, 37, W202–W208. 19458158 10.1093/nar/gkp335 PMC2703892

[imb12513-bib-0006] Biasio , F.D. , Riviello , L. , Bruno , D. , Grimaldi , A. , Congiu , T. , Sun , Y.F. *et al* (2015) Expression pattern analysis of odorant‐binding proteins in the pea aphid *Acyrthosiphon pisum* . Insect Science, 22, 220–234. 24591440 10.1111/1744-7917.12118

[imb12513-bib-0007] Bolger , A.M. , Lohse , M. and Usadel , B. (2014) Trimmomatic: a flexible trimmer for Illumina sequence data. Bioinformatics, 30, 2114–2120. 24695404 10.1093/bioinformatics/btu170 PMC4103590

[imb12513-bib-0008] Bowers , W.S. , Nault , L.R. , Webb , R.E. and Dutky , S.R. (1972) Aphid alarm pheromone: isolation, identification, synthesis. Science, 177, 1121–1122. 17840606 10.1126/science.177.4054.1121

[imb12513-bib-0009] Bwye , A.M. , Proudlove , W. , Berlandier , F.A. and Jones , R.A.C. (1997) Effects of applying insecticides to control aphid vectors and cucumber mosaic virus in narrow‐leafed lupins (*Lupinus angustifolius*). Australian Journal of Experimental Agriculture, 37, 93–102.

[imb12513-bib-0010] Calvello , M. , Guerra , N. , Brandazza , A. , D’Ambrosio , C. , Scaloni , A. , Dani , F.R. *et al* (2003) Soluble proteins of chemical communication in the social wasp *Polistes dominulus* . Cellular and Molecular Life Sciences (CMLS), 60, 1933–1943. 14523553 10.1007/s00018-003-3186-5 PMC11138633

[imb12513-bib-0011] Cui , H.H. , Gu , S.H. , Zhu , X.Q. , Wei , Y. , Liu , H.W. , Khalid , H.D. *et al* (2017) Odorant‐binding and chemosensory proteins identified in the antennal transcriptome of *Adelphocoris suturalis* Jakovlev. Comparative Biochemistry and Physiology Part D: Genomics and Proteomics, 24, 139–145. 10.1016/j.cbd.2016.03.001 27085212

[imb12513-bib-0012] Darriba , D. , Taboada , G.L. , Doallo , R. and Posada , D. (2011) ProtTest 3: fast selection of best‐fit models of protein evolution. Bioinformatics, 27, 1164–1165. 21335321 10.1093/bioinformatics/btr088 PMC5215816

[imb12513-bib-0013] Dawson , G.W. , Griffiths , D.C. , Merritt , L.A. , Mudd , A. , Pickett , J.A. , Wadhams , L.J. *et al* (1990) Aphid semiochemicals **– **a review, and recent advances on the sex pheromone. Journal of Chemical Ecology, 16, 3019–3030. 24263293 10.1007/BF00979609

[imb12513-bib-0014] Dewhirst , S.Y. , Pickett , J.A. and Hardie , J. (2010) Aphid pheromones. Vitamins and Hormones, 83, 551–574. 20831961 10.1016/S0083-6729(10)83022-5

[imb12513-bib-0015] Edwards , L.J. , Siddall , J.B. , Dunham , L.L. , Uden , P. and Kislow , C.J. (1973) *Trans*‐β‐Farnesene, alarm pheromone of the green peach aphid, *Myzus persicae* (Sulzer). Nature, 241, 126–127.4121143

[imb12513-bib-0016] Eskandari , F. , Sylvester , E.S. and Richardson J. (1979) Evidence for lack of propagation of potato leaf roll virus in its aphid vector, *Myzus persicae* . Phytopathology, 69, 45–47.

[imb12513-bib-0017] Fan , J. , Xue , W.X. , Duan , H.X. , Jiang , X. , Zhang , Y. , Yu , W.J. *et al* (2017) Identification of an intraspecific alarm pheromone and two conserved odorant‐binding proteins associated with (*E*)‐β‐farnesene perception in aphid *Rhopalosiphum padi* . Journal of Insect Physiology, 101, 151–160. 28778653 10.1016/j.jinsphys.2017.07.014

[imb12513-bib-0018] Forêt , S. and Maleszka , R. (2006) Function and evolution of a gene family encoding odorant binding‐like proteins in a social insect, the honey bee (*Apis mellifera*). Genome Research, 16, 1404–1413. 17065610 10.1101/gr.5075706 PMC1626642

[imb12513-bib-0019] Gong , D.P. , Zhang , H.J. , Zhao , P. , Lin , Y. , Xia , Q.Y. and Xiang , Z.H. (2007) Identification and expression pattern of the chemosensory protein gene family in the silkworm, *Bombyx mori* . Insect Biochemistry and Molecular Biology, 37, 266–277. 17296501 10.1016/j.ibmb.2006.11.012

[imb12513-bib-0020] Gong , D.P. , Zhang , H.J. , Zhao , P. , Xia , Q.Y. and Xiang , Z.H. (2009) The odorant binding protein gene family from the genome of silkworm, Bombyx mori. BMC Genomics, 10, 332. 19624863 10.1186/1471-2164-10-332 PMC2722677

[imb12513-bib-0021] Grabherr , M.G. , Haas , B.J. , Yassour , M. , Levin , J.Z. , Thompson , D.A. , Amit , I. *et al* (2011) Full‐length transcriptome assembly from RNA‐Seq data without a reference genome. Nature Biotechnology, 29, 644–652. 10.1038/nbt.1883 PMC3571712 21572440

[imb12513-bib-0022] Gu , S.H. , Wu , K.M. , Guo , Y.Y. , Field , L.M. , Pickett , J.A. , Zhang , Y.J. *et al* (2013) Identification and expression profiling of odorant binding proteins and chemosensory proteins between two wingless morphs and a winged morph of the cotton aphid *Aphis gossypii *Glover. PLoS ONE, 8, e73524. 24073197 10.1371/journal.pone.0073524 PMC3779235

[imb12513-bib-0023] Guo , W. , Wang , X.H. , Ma , Z.Y. , Xue , L. , Han , J.Y. , Yu , D. *et al* (2011) *CSP* and *takeout *genes modulate the switch between attraction and repulsion during behavioral phase change in the migratory locust. PLoS Genetics, 7, e1001291. 21304893 10.1371/journal.pgen.1001291 PMC3033386

[imb12513-bib-0024] He , M. and He , P. (2014) Molecular characterization, expression profiling, and binding properties of odorant binding protein genes in the whitebacked planthopper, *Sogatella furcifera* . Comparative Biochemistry and Physiology Part B: Biochemistry and Molecular Biology, 174, 1–8. 10.1016/j.cbpb.2014.04.008 24815350

[imb12513-bib-0025] Hekmat‐Scafe , D.S. , Scafe , C.R. , McKinney , A.J. and Tanouye , M.A. (2002) Genome‐wide analysis of the odorant‐binding protein gene family in *Drosophila melanogaster* . Genome Research, 12, 1357–1369. 12213773 10.1101/gr.239402 PMC186648

[imb12513-bib-0026] Li , H. (2013) Aligning sequence reads, clone sequences and assembly contigs with BWA‐MEM.arXiv 1303.3997v2.

[imb12513-bib-0027] Livak , K.J. and Schmittgen , T.D. (2001) Analysis of relative gene expression data using real‐time quantitative PCR and the 2^−^ ^ΔΔ^ ^Ct^ method. Methods, 25, 402–408. 11846609 10.1006/meth.2001.1262

[imb12513-bib-0028] Löytynoja , A. and Goldman , N. (2010) webPRANK: a phylogeny‐aware multiple sequence aligner with interactive alignment browser. BMC Bioinformatics, 11, 579. 21110866 10.1186/1471-2105-11-579 PMC3009689

[imb12513-bib-0029] Marsh , D. (1972) Sex pheromone in the aphid *Megoura viciae* . Nature New Biology, 238, 31–32. 18663846 10.1038/newbio238031a0

[imb12513-bib-0030] Mathers , T.C. , Chen , Y.Z. , Kaithakottil , G. , Legeai , F. , Mugford , S.T. , Baa‐Puyoulet , P. *et al* (2017) Rapid transcriptional plasticity of duplicated gene clusters enables a clonally reproducing aphid to colonise diverse plant species. Genome Biology, 18, 27. Available at: doi: 10.1101/063610. 28190401 PMC5304397

[imb12513-bib-0031] Modrek , B. and Lee , C. (2002) A genomic view of alternative splicing. Nature Genetics, 30, 13–19. 11753382 10.1038/ng0102-13

[imb12513-bib-0032] Mortazavi , A. , Williams , B.A. , McCue , K. , Schaeffer , L. and Wold , B. (2008) Mapping and quantifying mammalian transcriptomes by RNA‐Seq. Nature Methods, 5, 621–628. 18516045 10.1038/nmeth.1226 PMC13303166

[imb12513-bib-0033] Nault , L.R. , Edwards , L.J. and Styer , W.E. (1973) Aphid alarm pheromones: secretion and reception. Environmental Entomology, 2, 101–105.

[imb12513-bib-0034] Nei , M. and Rooney , A.P. (2005) Concerted and birth‐and‐death evolution of multigene families. Annual Review of Genetics, 39, 121–152. 10.1146/annurev.genet.39.073003.112240 PMC1464479 16285855

[imb12513-bib-0035] Nicholson , S.J. , Nickerson , M.L. , Dean , M. , Song , Y. , Hoyt , P.R. , Rhee , H. *et al.* (2015) The genome of *Diuraphis noxia*, a global aphid pest of small grains. BMC Genomics, 16, 429. 26044338 10.1186/s12864-015-1525-1 PMC4561433

[imb12513-bib-0036] Nomura , A. , Kawasaki , K. , Kubo , T. and Natori , S. (1992) Purification and localization of p10, a novel protein that increases in nymphal regenerating legs of *Periplaneta americana* (American cockroach). The International Journal of Developmental Biology, 36, 391–398. 1445782

[imb12513-bib-0037] Northey , T. , Venthur , H. , De Biasio , F. , Chauviac , F.‐X. , Cole , A. , Ribeiro , K.A.L. *et al* (2016) Crystal structures and binding dynamics of odorant‐binding protein 3 from two aphid species *Megoura viciae* and *Nasonovia ribisnigri* . Scientific Reports, 6, 24739. 27102935 10.1038/srep24739 PMC4840437

[imb12513-bib-0038] Pelosi , P. , Zhou , J.J. , Ban , L.P. and Calvello , M. (2006) Soluble proteins in insect chemical communication. Cellular and Molecular Life Sciences, 63, 1658–1676. 16786224 10.1007/s00018-005-5607-0 PMC11136032

[imb12513-bib-0039] Petersen , T.N. , Brunak , S. , Heijne , G.V. and Nielsen , H. (2011) SignalP 4.0: discriminating signal peptides from transmembrane regions. Nature Methods, 8, 785–786. 21959131 10.1038/nmeth.1701

[imb12513-bib-0040] Pickett , J.A. and Glinwood , R.T. (2007) Chemical ecology In: Van Emden, H.F. and Harrington, R. (Eds.) Aphids as Crop Pests. Wallingford, UK: CAB International Press, pp. 235–260.

[imb12513-bib-0041] Pickett , J.A. and Griffiths , D.C. (1980) Composition of aphid alarm pheromones. Journal of Chemical Ecology, 6, 349–360.

[imb12513-bib-0042] Pickett , J.A. , Wadhams , L.J. , Woodcock , C.M. and Hardie, J. (1992) The chemical ecology of aphids. Annual Review of Entomology, 37, 67–90.

[imb12513-bib-0043] Qiao , H. , Tuccori , E. , He , X. , Gazzano , A. , Field , L. , Zhou , J.‐J. *et al* (2009) Discrimination of alarm pheromone (*E*)‐β‐farnesene by aphid odorant‐binding proteins. Insect Biochemistry and Molecular Biology, 39, 414–419. 19328854 10.1016/j.ibmb.2009.03.004

[imb12513-bib-0044] Ramakers , C. , Ruijter , J.M. , Deprez , R.H.L. and Moorman , A.F.M. (2003) Assumption‐free analysis of quantitative real‐time polymerase chain reaction (PCR) data. Neuroscience Letters, 339, 62–66. 12618301 10.1016/s0304-3940(02)01423-4

[imb12513-bib-0045] Read , D.P. , Feeny , P.P. and Root , R.B. (1970) Habitat selection by the aphid parasite *Diaeretiella rapae* (Hymenoptera: Braconidae) and hyperparasite *Charips brassicae* (Hymenoptera: Cynipidae). The Canadian Entomologist, 102, 1567–1578.

[imb12513-bib-0046] Scaloni , A. , Monti , M. , Angeli , S. and Pelosi , P. (1999) Structural analysis and disulfide‐bridge pairing of two odorant‐binding proteins from *Bombyx mori* . Biochemical and Biophysical Research Communications, 266, 386–391. 10600513 10.1006/bbrc.1999.1791

[imb12513-bib-0047] Stamatakis , A. (2014) RAxML version 8: a tool for phylogenetic analysis and post‐analysis of large phylogenies. Bioinformatics, 30, 1312–1313. 24451623 10.1093/bioinformatics/btu033 PMC3998144

[imb12513-bib-0048] Sun , Y.P. , Zhao , L.J. , Sun , L. , Zhang , S.G. and Ban , L.P. (2013) Immunolocalization of odorant‐binding proteins on antennal chemosensilla of the peach aphid *Myzus persicae *(Sulzer). Chemical Senses, 38, 129–136. 23222972 10.1093/chemse/bjs093

[imb12513-bib-0049] The International Aphid Genomics Consortium . (2010) Genome sequence of the pea aphid *Acyrthosiphon pisum* . PLOS Biology, 8, e1000313. 20186266 10.1371/journal.pbio.1000313 PMC2826372

[imb12513-bib-0050] Troncoso , A.J. , Vargas , R.R. , Tapia , D.H. , Olivares‐Donoso , R. and Niemeyer , H.M. (2005) Host selection by the generalist aphid *Myzus persicae* (Hemiptera: Aphididae) and its subspecies specialized on tobacco, after being reared on the same host. Bulletin of Entomological Research, 95, 23–28. 15705211 10.1079/ber2004334

[imb12513-bib-0051] Ueda , N. and Takada , H. (1977) Differential relative abundance of green–yellow and red forms of *Myzus persicae* (Sulzer) (Homoptera: Aphididae) according to host plant and season. Applied Entomology and Zoology, 12, 124–133.

[imb12513-bib-0052] Van Emden , H.F. , Eastop , V.F. , Hughes , R.D. and Way , M.J. (1969) The ecology of *Myzus persicae* . Annual Review of Entomology, 14, 197–270.

[imb12513-bib-0053] Vandermoten , S. , Francis , F. , Haubruge , E. and Leal , W.S. (2011) Conserved odorant‐binding proteins from aphids and eavesdropping predators. PLoS ONE, 6, e23608. 21912599 10.1371/journal.pone.0023608 PMC3160308

[imb12513-bib-0054] Vogt , R.G. and Riddiford , L.M. (1981) Pheromone binding and inactivation by moth antennae. Nature, 293, 161–163. 18074618 10.1038/293161a0

[imb12513-bib-0055] Wang , R. , Li , F. , Zhang , W. , Zhang , X. , Qu , C. , Tetreau , G. *et al* (2017) Identification and expression profile analysis of odorant binding protein and chemosensory protein genes in *Bemisia tabaci *MED by head transcriptome. PLoS ONE, 12, e0171739. 28166541 10.1371/journal.pone.0171739 PMC5293548

[imb12513-bib-0056] Weber , G. (1986) Ecological genetics of host plant exploitation in the green peach aphid, *Myzus persicae* . Entomologia Experimentalis et Applicata, 40, 161–168.

[imb12513-bib-0057] Wenger, J.A. , Cassone , B.J. , Legeai , F. , Johnston , J.S. , Bansal , R. , Yates , A.D. *et al* (2017) Whole genome sequence of the soybean aphid. Insect Biochemistry and Molecular Biology. Available at: doi: 10.1016/j.ibmb.2017.01.005. 28119199

[imb12513-bib-0058] Williams , I.S. , Dewar , A.M. , Dixon , A.F.G. and Thornhill , W.A. (2000) Alate production by aphids on sugar beet: how likely is the evolution of sugar beet‐specific biotypes? Journal of Applied Ecology, 37, 40–51.

[imb12513-bib-0059] Xu , P.X. , Zwiebel , L.J. and Smith , D.P. (2003) Identification of a distinct family of genes encoding atypical odorant‐binding proteins in the malaria vector mosquito, *Anopheles gambiae* . Insect Molecular Biology, 12, 549–560. 14986916 10.1046/j.1365-2583.2003.00440.x

[imb12513-bib-0060] Xu , Y.L. , He , P. , Zhang , L. , Fang , S.Q. , Dong , S.L. , Zhang , Y.J. *et al* (2009) Large‐scale identification of odorant‐binding proteins and chemosensory proteins from expressed sequence tags in insects. BMC Genomics, 10, 632. 20034407 10.1186/1471-2164-10-632 PMC2808328

[imb12513-bib-0061] Xue , W.X. , Fan , J. , Zhang , Y. , Xu , Q.X. , Han , Z.L. , Sun , J.R. *et al* (2016) Identification and expression analysis of candidate odorant‐binding protein and chemosensory protein genes by antennal transcriptome of *Sitobion avenae* . PLoS ONE, 11, e0161839. 27561107 10.1371/journal.pone.0161839 PMC4999175

[imb12513-bib-0062] Yuan , H.B. , Ding , Y.X. , Gu , S.H. , Sun , L. , Zhu , X.Q. , Liu , H.W. *et al* (2015) Molecular characterization and expression profiling of odorant‐binding proteins in *Apolygus lucorum* . PLoS ONE, 10, e0140562. 26466366 10.1371/journal.pone.0140562 PMC4605488

[imb12513-bib-0063] Zhang , R.B. , Wang , B. , Grossi , G. , Falabella , P. , Liu , Y. , Yan , S.C. *et al* (2017) Molecular basis of alarm pheromone detection in aphids. Current Biology, 27, 55–57. 27916525 10.1016/j.cub.2016.10.013

[imb12513-bib-0064] Zhong , T. , Yin , J. , Deng , S.S. , Li , K.B. and Cao , Y.Z. (2012) Fluorescence competition assay for the assessment of green leaf volatiles and *trans*‐β‐farnesene bound to three odorant‐binding proteins in the wheat aphid *Sitobion avenae* (Fabricius). Journal of Insect Physiology, 58, 771–781. 22306433 10.1016/j.jinsphys.2012.01.011

[imb12513-bib-0065] Zhou , J.J. , Huang , W.S. , Zhang , G.A. , Pickett , J.A. and Field , L.M. (2004) “Plus‐C” odorant‐binding protein genes in two *Drosophila* species and the malaria mosquito *Anopheles gambiae* . Gene, 327, 117–129. 14960367 10.1016/j.gene.2003.11.007

[imb12513-bib-0066] Zhou , J.J. , Kan , Y.C. , Antoniw , J. , Pickett , J.A. and Field , L.M. (2006) Genome and EST analyses and expression of a gene family with putative functions in insect chemoreception. Chemical Senses, 31, 453–465. 16581978 10.1093/chemse/bjj050

[imb12513-bib-0067] Zhou , J.J. , He , X.L. , Pickett , J.A. and Field , L.M. (2008) Identification of odorant‐binding proteins of the yellow fever mosquito *Aedes aegypti*: genome annotation and comparative analyses. Insect Molecular Biology, 17, 147–163. 18353104 10.1111/j.1365-2583.2007.00789.x

[imb12513-bib-0068] Zhou , J.J. , Vieira , F.G. , He , X.L. , Smadja , C. , Liu , R. , Rozas J. *et al* (2010) Genome annotation and comparative analyses of the odorant‐binding proteins and chemosensory proteins in the pea aphid *Acyrthosiphon pisum* . Insect Molecular Biology, 19, 113–122. 10.1111/j.1365-2583.2009.00919.x 20482644

[imb12513-bib-0069] Zhou , S.S. , Sun , Z. , Ma , W.H. , Chen , W. and Wang , M.Q. (2014) *De novo* analysis of the *Nilaparvata lugens* (Stål) antenna transcriptome and expression patterns of olfactory genes. Comparative Biochemistry and Physiology Part D: Genomics and Proteomics, 9, 31–39. 10.1016/j.cbd.2013.12.002 24440828

[imb12513-bib-0070] Zhou , W.W. , Yuan , X. , Qian , P. , Cheng , J.A. , Zhang , C.X. , Gurr , G. *et al* (2015) Identification and expression profiling of putative chemosensory protein genes in two rice planthoppers, *Laodelphax striatellus* (Fallén) and *Sogatella furcifera* (Horváth). Journal of Asia‐Pacific Entomology, 18, 771–778.

