# Supplementary figures and images for: Integrative transcriptomic and genomic analysis of odorant binding proteins and chemosensory proteins in aphids
Source: Insect Mol Biol. 2018 Oct 5;28(1):1–22. doi: 10.1111/imb.12513 (PMC7380018; doi:10.1111/imb.12513)

Figure S1.

*
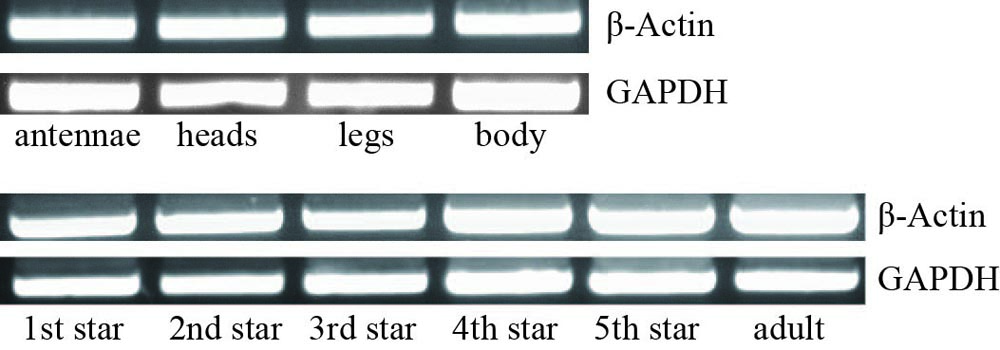
*

Figure S2.

Figure S3.

Supplement: Supplementary file 1 — FIGURE S1. The sTABLE expression of M. persicae β‐Actin and GAPDH in different development stages and tissues measured by RT‐PCR. FIGURE S2. The standard curves of M. persicae OBPs and CSPs with GAPDH as reference gene. FIGURE S3. The standard curves of M. persicae OBPs and CSPs with β‐actin as reference gene. [file IMB-28-1-s001.docx]
