# Supplementary material for: Integrative transcriptomic and genomic analysis of odorant binding proteins and chemosensory proteins in aphids
Source: Insect Mol Biol. 2018 Oct 5;28(1):1–22. doi: 10.1111/imb.12513 (PMC7380018; doi:10.1111/imb.12513)
Supplement: Supplementary file 2 — TABLE S1. Gene specific primers used for cloning of M. persicae OBP and CSP genes. TABLE S2. The protein names and sequences of the 45 OBPs and 41 CSPs from M. persicae, A. gossypii, A. pisum, A. glycines and S. avenae used in Fig. 7. TABLE S3. The protein names and sequences of the 199 OBPs and 103 SPs from Hemiptera insects used in Fig. 8. TABLE S4. Primers used in RT‐PCR for determination expression levels of M. persicae OBP and CSP genes. TABLE S5. Primers used in real‐time PCR for determination expression level of M. persicae OBP and CSP genes. The real PCR amplification efficiencies of target and reference genes were calculated using LinRegPCR program (version 11.0) (Ramakers et al., 2003). TABLE S6. A percent identity matrix of M. persicae OBPs. TABLE S7. A percent identity matrix of M. persicae CSPs. TABLE S8. The protein names and sequences of the Hemiptera 237 OBPs used in Fig. 3. TABLE S9. The protein names and sequences of the Hemiptera 110 CSPs used in Fig. 4. [file IMB-28-1-s002.docx]

**Table S1.**

| **Gene** | **Forward primer (5’-3’)** | **Reverse primer (5’-3’)** |
| --- | --- | --- |
| *OBP2* | ATGAAGGTATCTGCAGCGACC | TTATGCTTTAGGGAAGAAATTTATTTT |
| *OBP3* | ATGATTTCGTCGACGTTTTACATAA | TCAAGTTGACTTGTCGAGATCCA |
| *OBP4* | ATGCGTGGAAATTATTCTTTGAC | TTAAACTTGACGTTGGCTTAAGTTAT |
| *OBP5* | ATGTCCGCTAACTCTGCTACGA | TTAGTTTGATGTCGATTTTTGCAT |
| *OBP6* | ATGCAAAAGGTGGTTTTTATATGTAT | TTAAATTAATTTGGGTGCGGATT |
| *OBP7* | ATGAATAATATGATACCAGCTACAGTTTT | CTATAGTGGTAGATACTCTAAACTTTTTGG |
| *OBP8* | ATGTTCGTTCTTAAAGTGGCGT | TTACATGCTATTGCGTCTGAATTT |
| *OBP9* | ATGTTAATAAAAAAGACATTGTTAGTGTCA | TTATTTCGATTTTGGTTTCATCTTC |
| *OBP10* | ATGGAACATTTACGTAGCACAAAC | TCACAATGGCAACAGTTGAATAGT |
| *CSP1* | ATGAATTTGTTAGCGGTTTTTTGTTA | TTATTTGGTTGTATCGTTGGTGAGT |
| *CSP2* | ATGGCTCATCTTAACTTATTTGTCGT | TTAATGTTTAGCAGCTGCAGCC |
| *CSP4* | ATGGATTCACGAATTGCAGTAGTC | TTAAAATTTGACAACACCTTTTTTCTTC |
| *CSP5* | ATGAACTGCAAGGTCTTGATCG | TTACGCGTCGAGGAATTTGTTC |
| *CSP6* | ATGAACACACTTCTCCTAGCAGTTG | TTAAACATCAAGGTTCAATTTCTTTG |
| *CSP7* | ATGGATCGATCGTCGTCAAGT | TTAGAATCCGCTCTGGTACTGTTT |
| *CSP8* | ATGACGAATAATAATATGAACAGTCCG | CTATTTTTGGTTCATGAAATCGATG |
| *CSP9* | ATGACGTCGTTTTGTCTGAACTCT | TTAAGCTGTCATATTATTTCTATAAATTGGT |
| *CSP10* | ATGGTCTCAAAACTGTTTGTTTCC | TTATTTCGATTCGTTTAAGTTCTCTAAA |

**Table S2.**

>SaveOBP2

MKVSAATAVLVALVATVQSSDPCNISTCYKSGTTKPPMAVTPTRLPVQSSSTPTSHPQTTYAKDHVHGSTTIKSGANATATTASGASVNGTERPIVVKSSAGVIGNSTTPKPTMTEGHVALKQKLNTIAVKCKDELHAPQEIMALVSNTVVPQNEQQRCYLECVYKNLNLIKNNKFSVEDGKAMARIRFANQPEEHKKAVTIIETCEKEAIIDPKITEKCAAGRIIRNCFVKNGEKINFFPKA

>SaveOBP3

MISSTFYITSVFGIALLISCGYGRFTTDQIDYYGKACNASEDDLVVVKSYKVPSTETGKCLMKCMITKLGLLNDDGSYNKTGMEAGLKKYWSEWSTEKIENINNKCYEEALLVSKEVVATCNYSYTVMACLNKQLDLDKST

>SaveOBP4

MRGNYSLMVFLLFAIGLQDIFCQKQEPSGKCRAPDKAPLNLEIIINICQEEIKSALLQEALDILNDGNLEQNTPSHSSRSKREADEDLTNEERRVAGCLLQCVYKKVKAVDETGFPVVDGLMKLYNEGVQDRNYYMATLSAVRHCISIAQQLKQQQPSKSFDDGQTCDLAYEMFECVSEKIEENCGVENKSNNLSQRQV

>SaveOBP5

MSVNSATIKCIAVAVVLLQISIIFADAGHHRRGKELLDTEDSDFFRCKQASRKSCCGPENAMKRFGDKDKVAADECYAQVAEKFATVTATTPKQDLFSAEAVKITKKKQFCLHECIGKKNNLLTEDGSLNKTFIADYAMKSVFKEQWQKQVGQKALDKCLEETYIPWPAEDKENVCNPVYVQFQHCLWLQYESNCPANKIKTTKKCEKTRNRYRMQKSTSN

>SaveOBP6

MQKVVFICIFAIICQTVFTAGYDRTWILRQKRMTNDDECRTLIPSPEKKLPSCCQMPDILPNSNSTWEKCFETFKQFKDKPETKEYKEMAHGKEPPCLFQCIFMQSGLTTSDGKLNEDAITKKMSEGINNDEKWKSTWQNSLNKCFDDVKQEDKKQIPIMNTPAGRLMKCFLRDMYMSCPKNVWVESSECLNMKDLVQKCPEMPPPVFKSPPKLI

>SaveOBP7

MYNMLPKTVLFAIIAATVLKDCDAYLSEAAIKKTQQMLKTVCSKKFSVEEDVFTDIKKGIFPEDNNNIKCYFACNFKTMQLINQKGSIDKKMFKDKMTMMAPPNVLKVLLPVIEQCTGIDKGEELCQSSYNLIKCAHTVDPRSLEYLPL

>SaveOBP8

MFALKVACLCLSVAVVFGENNQQNGPSDRSASIFQSCIAETKLSGDALKGFRSMSIPKTQAEKCMMGCLMRKVNVINKGKFSVEEATKVAQKYYGTNEVMMKKAKDLIDVCAKKAQSTTEECALAGIVTTCIVEEAQKAGLAGGPGSRSRRTVSPKFRRDAM

>SaveOBP9

MIIKKTLLVSVFVLFGCLFSINKAADDADAGDKELMSKLFTVVLKCFKDADWGTCGEMITTKYDITQAKYKQCTCHMACAGEELGMINTSGQPEPAKFLEYVNKINHPSIKSQLQLIYDKCHNVKGSEKCDLAEQFAICAFKESPALKERAATLMEMLVKMKPKSK

>SaveOBP10

MEHLRSTNVVFAIVMALLVVQSSTRPQPDEMEEIKKTLYNACAGKFPITEEIKKNAKNAIISDDPTFKCFLKCCFDEMSLIDEDGILDGDSMKAMAPDHIKPIFEQVIPSCLKNVKQDGCEASFEFISCGMKLNPLTVELLPL

>AgosOBP2

MKVSAATAVLVALVATVQSSDPCNISTCYKSGTTKPPTTVTPTRLPVQSSSTPTSHQQTTYAKDHVHSSTATKSGVNTTATTTSGASVNGTERTTVVKSSSGVAGNVTTPKPTMTDGHVALKQKLNTIAVKCKDELHAPQEIMALVSNTVVPQNEQQRCYLECVYKNLNLIKNNKFSVDDGKAMAKIRFANQPEEHKKAVTIIETCEKEAIIDPKTTEKCAAGRVIRNCFVKNGEKINFFPKA

>AgosOBP3

MISSTFYTSLMFGIAMLISCSFGRFTTEQIDHYGKACNATEDDLVIVKSYKVPTSDTGKCLMKCMISKLGLLNDDGSYNKTGMEAGLKKYWSEWSTDTIESINNKCYEEALLVSKDIIATCNYAYVVMACLNKQLKLDNST

>AgosOBP4

MRGNYSLVVFLLFGFGLLEIYCQKQELSGKCRAPDKAPLNLEIIINICQEEIKSALLQEALDILNDGTLEQNTPSYSRSKRDADEDLSNEERRVAGCLLQCVYKKVKAVDETGFPVVDGLMKLYNEGVQDRNYYMATLSAVRHCISIAQQLKQQQPSKSFDDGQTCDLAYEMFECVSEKIEENCGVENKLNNLSQRQV

>AgosOBP5

MKMSANGATMKCVAVAVVLFQMSVIFAEAGHQRRGKELLDTEDSDFFRCKQASRKSCCGPENAMKRFGDKDKVAADECYAQVAEKFATVTATTPKQDLFSGEAVKITKKKQFCLHECIGKKNKLLTEDGSLNKTFIADYAMKSVFKEQWQKQIGQKALDKCLEETYIPWPAEETENKCNPVYVQFQHCLWLEYESNCPDNKIKLTKKCEKTRNRYRMQKSPSNQ

>AgosOBP6

MQKVVFLCIFAIICQTVFTVGFERTWILRQKRMTNDNECRALFPSPEKKLPTCCQMPNILPGLDNAWEVCFEKFKQFKDKHATKEYKEMVHENEPPCLFQCVFMQSGLTTSDGKVNEDAVIKKMAEGMDNDEKWKSIWRNTFNKCLNDVKQEDKEQIKVMNTPTGRLMKCFLRDLYMNCPKNVWVENSECSNLKDLVEKCPKLPPPVFQSPPKLI

>AgosOBP7

MNMLPATVLLAVVAATILKDSDAYLSEEAIKKTQKMLKNVCSKKHSVEEEVFTDIKKGIFPENNNNIKCYFACNFKTMQMVNQKGILDKKMFKDKMTMLAPPNVLAILLPPIEQCIGNDKDTEICQSSYNFIKCAHRVDPKSLEFLPL

>AgosOBP8

MFAFKVACLCLSVAVVFGENNQQNSNDRSASIFQSCISETKLSGDALKGFRSMSIPKTQAEKCMMGCLMRKVNVINKGKFSVEEATKVAQKYYGTNESMMKKAKDLIDVCAKKAQSTTEECALAGIVTTCIVEEAQKAGLTGGPGSRSKRTVSPKFRHSIV

>AgosOBP9

MIIKKTLLVSGFVLFGCMFSINKAADDADTADKELMSKLITVAFKCFKDADWGTCGEMITTKYDITQAKYKQCTCHMACAGEDLGLINSNGQPEPAKFLEYVKRINNSVIKSQLQHIYDKCQNVKGTEKCDLAEQFAICAFKESPEMKERVTKLIEMLVKMKPKSK

>AgosOBP10

MEHLRGTNVMFAIVMALLVVQSSTRPQPDEPDDIKKTLYNACSEKFPLTEEIKNNVKNSMVIDDQNFKCFLRCCFDEMSLIDEDGIIDGESLAAMAVDKIKPVAEKIVHDCLPAGKQEKQDGCEAAFKFFSCGMKLNPLTIELLPLQ

>MperOBP2

MKVSAATAVLVALVATVQSSDPCNISTCYKSGTTKPPMNVTPTRLPVQSSSTPTSHPQTTYAKDHAHGSTTVKSGANATATTASGASVNGTERPAVAKSSAGVTGNSTTPKPTMTEGHVALKQKLNTIAVKCKDELHAPQEIMALVSNTVVPQNEQQRCYLECVYKNLNLIKNNKFSVDDGKAMARIRFANQPEEHKKAVTIIETCEKEAVIDPKTTEKCAAGRVIRNCFVKNGEKINFFPKA

>MperOBP3

MISSTFYITLLFGIAMLISCGYGRFSTEQIDYYGKACNASEDDLVVVKSYKVPTTETGKCLMKCMITKLGLLNDDGSYNKTGMEAGLKKYWSEWSTEKIEAINNKCYEEALLVSKEVIATCSYTVMACLNKQLDLDKST

>MperOBP4

MRGNYSLTVFLLFVIGLQDIYCQKQEPSGKCRAPDKAPLNLEIIINICQEEIKSALLQEALDILNDGNLEQNTPSYSSRSKREADEDLTNEERRVAGCLLQCVYKKVKAVDETGFPVVDGLMKLYNEGVQDRNYYMATLSAVRHCISIAQQLKQLQPSKSFDDGQTCDLAYEMFECVSEKIEENCGVENKSNNLSQRQV

>MperOBP5

MSANSATIKCIAVAVVLLQISVVFADAGHHRRGKELLDTEDSDFFRCKQASRKSCCGPENAMKRFGDKDKVAADECYAQVAEKFATVAATTPKQDLFSADAVKITKKKQFCLHECIGKKNRLLTEDGSLNKTFIADYAMKSVFKEQWQKQVGQKALDKCLEETYIPWPAEDKENVCNPVYVQFQHCLWLQYESNCPDNKIKITKKCEKTRNRYRMQKSTSN

>MperOBP6

MQKVVFICIFAIIYQTVFTVGYERTWILRQKRMTNDDECRTLLPSSEKKLPSCCQMPNILPGLDSTWEKCYEKFIQFKDKPETKEYKEMSHGKEPPCLFQCIFMESGLTTNDGKLNEDAITKKMTEGINNDEKWKSTWKKSLDKCFDDVKQEDKKQILIMNTPAGRLMKCFLRDIYMNCPENVWVESSECLNVKNLVQKCPEMPPPVFQSAPKLI

>MperOBP7

MNNMIPATVLLAVIAATVLKDCDAYLSEAAIKKTQQMLKTVCSKKHSVEEDVFTDIKKGIFPENNNNIKCYFACNFKTMQMINQKGTLDKKLFKDKMSMMAPPNIYNILLPAIEQCIGIDKGEELCQSSYNFIKCAHRVDPKSLEYLPL

>MperOBP8

MFVLKVACLCLSVAVVFGENNQQNSSDRSATIFQSCIAETKLSGDALKGFRSMSIPKTQAEKCMMGCLMRKVNVINKGKFSVEEATKVAQKYYGTNETMMKKAKDLIDVCAKKAQSTTEECALAGIVTTCIVEEAQKAGLAGGPGSRSRRTVSPKFRRNSM

>MperOBP9

MLIKKTLLVSVFVLFSCLFSINKATDDADTADKELMSKLFTVVFKCFKDADWGTCGEMITTKYDITQAKYKQCTCHMACAGEELGLINSSGQPEPAKFLEYVNRINNPGIKSQLQHIYDKCQNVKGTEKCDLAEQFAICAFKESPALKERATTLMEILMKMKPKSK

>MperOBP10

MEHLRSTNVVFAIVMALLVVQSSTRPQPDELEEIKKTLYNACAGKFPITEEMKKDILNSNMVDDQNFKCFLRCCFDEMSMIDEDGIIDGESLISMATDNLKPVIQQVVQSCVKDIKQDGCEAAFNFISCGLKLNPMTIQLLPL

>ApisOBP2

MKVSAATAVLVALVATVQSSDPCNISTCYKSGTTKPPMSVTPTRLPVQSSSTPTSHPQTTYAKDHSHGSTTTKSGANATATTASGASVNGTERPAVVKSSAGVTGNLTTPKPTMTEGHVALKQKLNTIAVKCKDELHAPQEIMALVSNTVVPQNEQQRCYLECVYKNLNLIKNNKFSVEDGKAMAKIRFANQPDEHKKAVTIIETCEKEAVIDPKTTEKCAAGRVIRNCFVKNGEKINFFPKA

>ApisOBP3

MISSTFYLTSLFGIAMLISCGYGRFTTEQIDYYGKACNASEDDLVVVKSYKVPSSETGKCLMKCMITKLGLLNDDGSYNKTGMEAGLKKYWSEWSTEKIESINNKCYEEALLVSKEVIATCNYSYTVMACLNKQLDLDKST

>ApisOBP4

MRGNYSLMVFLLLAIGSQDIYCQKQELSGKCKAPDKAPLNLEIIINICQEEIKSALLQEALDILNEGNLEQNTPSYSSRSKREADEDLTNEERRVAGCLLQCVYKKVKAVDETGFPVVDGLMKLYNEGVQDRNYYMATLSAVRHCISIAQQLKQQQPSKSFDDGQTCDLAYEMFECVSEKIEENCGVENKSNNLSQRQV

>ApisOBP5

MSVNSLTIKCIAAAVVLLQISVIFADAGHHRRGKELLDTEDSDFFRCKQASRKSCCGPENAMKRFGDKDKVAADECYAQVAEKFATVTATTPKQDLFSADAVKITKKKQFCLHECIGKKNHLLTEDGSLNKTFIADYAMKSVFKEQWQKPVGLKALEKCLEETYIPWPAEDKENVCNPVYVQFQHCLWLQYESNCPANKIKITKKCEKTRNRYRMQKLTSN

>ApisOBP6

MQKVVFVCIFAIICQTVFTVGYDRTWILRQKRMTNDDECRTLIPGPEKKLPSCCQMPNILPNMDSTWEKCFETFKQFKDKSETKQYKEMAHGKEPPCLFQCIFMQSGLTTSDGKLNKDAITKKMSEGINNDEKWKSTWQNSLNKCFDDVKQEDKKQIPIMNTPAGRLMKCFLRDMYMSCPKNVWVESSECLNVKDLVQKCPEMPPPVFKSPPQLI

>ApisOBP7

MVAQKRMYNMLPTTVLFAVIAATVLKDCDAYLSETAIKKTQQMLKSVCSKKHSVNEDVFLDIKKGIFPEDNNNIKCYFACNFKTMQLINQKGSIDKKMFRDKMSMMAPPNVFNILSPVIEQCTGIDDGKELCQSSYNVIKCAHRVNPKSLEYLPL

>ApisOBP8

MFALKVAYLCLSVAVVFGENNQQNSNDRSATIFQSCISETKLSGDALKGFRSMSIPKTQAEKCMMGCLMRKVNVINKGKFSVEEATKVAQKYYGTNETMMKKAKDLIDVCAKKAQSTTEECALAGIVTTCIVEEAQKAGLSGGPGSRSRRTVSPKFRRNVM

>ApisOBP9

MIIKKTLLVSVFIIFGCLFSINKAADDADAADKELISKLFTVVFKCFKDADWGACGEMITTKYDITQAKYKQCTCHMACAGEELGMINSSGQPEPAKFLEYVKRINNPDIKSQLQLVYDKCQNVKGSEKCDLAEQFAICAFKESPALKERVATLMELLVKMKPKSK

>ApisOBP10

MEHLRKTNVVFGVVIVLLVIQKSSTRPQPDELEEIKKTLYNACAGKFPITEEVKNNAKNSIFLDDQNFKCFLKCCLDEMSLIDDDGIIDGDSLKAMASDKIKPILEQVVPNCLKDVKQDGCEAAFDFLSCGIKLNPLTVELLPL

>AglyOBP2

MKVSAATAVLVALVATVQSSDPCNISTCYKSGTTKPPTTVTPTRLPVQSSSTPTSHQQTTYAKDHVHSSTATKSGVNTTATTTSGASVNGTERTTVVKSSSGVAGNVTTPKPTMTDGHLALKQKLNTIAVKCKDELHAPQEIMALVSNTVVPQNEQQRCYLECVYKNLNLIKNNKFSVDDGKAMAKIRFANQPEEHKKAVTIIETCEKEAIIDPKTTEKCAAGRVIRNCFVKNGEKINFFPKA

>AglyOBP3

MISSTFYTSLMFGIVMLISCSFGRFTTEQIDHYGKACNATEDDLVVVKSYKVPTSDTGKCLMKCMISKLGLLNDDGSYNKTGMEAGLKKYWSEWSTDTIESINNKCYEEALLVSKDIIATCNYAYVVMACLNKQLDLDKST

>AglyOBP4

MRGNYSLVVFLLFGFGLLEIYCQKQETSGKCRAPDKAPLNLEIIINICQEEIKSALLQEALDILNDGTLEQNTPSYSRSKRDADEDLSNEERRVAGCLLQCVYKKVKAVDETGFPVVDGLMKLYNEGVQDRNYYMATLSAVRHCISIAQQLKQQQPSKSFDDGQTCDLAYEMFECVSEKIEENCGVENKSNNLSQRQV

>AglyOBP5

MKMSTNGATMKCVAIAVVLFQMSVIFAEAGHQRRGKELLDTEDSDFFRCKQASRKSCCGPENAMKRFGDKDKVAADECYAQVAEKFATVTATTPKQDLFSGEAVKITKKKQFCLHECIGKKNKLLTEDGSLNKTFIADYAMKSVFKEQWQKQIGQKALDKCLEETYIPWPAEETENKCNPVYVQFQHCLWLEYESNCPDNKIKLTKKCEKTRNRYRMQKSPSNQ

>AglyOBP6

MQKVVFLCIFAIICQTVFTVGFERTWILRQKRVTNDDECRTLIPSSEKKLPTCCQMPNILPGLDNAWEVCFEKFKQFKDKHATKEYKEMAHGNEPPCLFQCVFMQSGLTTSDGKVNEDAVIKKMAEGMDNDEKWKSIWRNTFNKCLNDVKQEDKEQIKMTNTPTGRLMKCFLRDLYMNRPKNVWVESSECSNLKDLVEKCPKMPPPVFKSPPKLI

>AglyOBP7

MVARKRMYMLPATVLLAVVAATILKDSDAYLSEEAIKKTQKMLKNVCSKKHSVEEEVFTDIKKGIFPENNNNIKCYFACNFRTMQMVNQKGILDKKMFKDKMTMLAPPNVLAILLPPIEQCIGNDKDTEICRSSYNFIKCAHRVDPKSLEFLPL

>AglyOBP8

MFAFKVACLCLSVAVVFGENNQQNSNDRSASIFQSCISETKLSGDALKGFRSMSIPKTQAEKCMMGCLMRKVNVINNGKFSVEEATKVAQKYYGTNETMMKKAKDLIDVCAKKAQSTTEECALAGIVTTCIVEEAQKAGLTGGPGSRSKRTVSPKFRHSIV

>AglyOBP9

MIIKKTLLVSGFVLFGCMFSINKAADDADAKDKELMSKLITVAFKCFKDADWGTCGEMITTKYDITQAKYKQCTCHMACAGEDLGLINSNGQPEPAKFLEYVKRINNSVIKSQLQHIYDKCQNVKGTEKCDLAEQFAICAFKESPEMKERVTKLIEMLVKMKPKSK

>AglyOBP10

MEHLRGTNVVFAIVMALLVVQSSTRPQPDELDDIKKTLYNACSEKFPLTEEIKNNVKNSIVIDDQNFKCFLRCCFDEMSLIDEDGIIDGESLAAMAVDKIKPVAEKIVHDCLPAGKQEKQDGCEASFKFFSCGIKLNPLTIELLPLQ

>SaveCSP4

MDSRIAVVCVVLAVFAVDQTVGAPQKDALAAGSPTTYTNKYDHIDIDQVLASKRLVNSYVQCLLDKKPCTPEGAELRKILPDALKTQCAKCSATQKNAALKVVDRLQKDYDKEWKQLLDKWDPKREQFQKFQQFLTEEKKKGVVKF

>SaveCSP5

MNCKVLIALCCVAVYAAQANPAGAATATAADDEIKDFPAYMKRFDKLNVEQVLNNDRVLASHLKCFLNEGPCVQQSRDLKRVIPVIANNGCNGCTERQMTTIKKSLNFLRTKKPVEWARLVKIYDPSGTKLNKFLDA

>SaveCSP7

MARSSSTSVTMKVFVMAVCVCAALARPEEAKMENKPTAVKSETLAAPLPTTIVKRATPQVVSIQKDASLPNVSEDVLDKALSDRRFVQRQLKCATGEGPCDPIGRKIKAHAPLVLRGMCVKCSQSEIKQIQRVMSHIQKNYPKEYTKMLKQYQSGF

>SaveCSP1

MNLLAIFCYITMMCDSQFRRLEQPTAIPQVKRIEQPATIATRIGQATIAPRFGQPTVAPRFGQPTIAPRFGQATAAPQTGEAAIGPRIGQTFQNVNDSVSPTTDGRKTTRETSSYPTRYDFIDIEAVMNNDRIIKILFNCVMNQGPCTREGLELKRIVPDAIQTECAKCNERQRKQAGKVLAHLLQYKPEYWNMLVKKFDPNNIYLRKYMADNDDDEKLSLQKLSNNTTK

>ApisCSP1

MNLLAIFCYITMMCDSQFRRLEQMTAMPQVKQPATIATRIGQATIAPRFGQPTIAPRFGQATVAPQVGQAAVTPQIGQAAIGSRIGQSFQSVNGSVTPTTDGRKTTRETASYPTRYDFIDIEAVMNNDRIIKILFNCVMNQGPCTREGLELKRIVPDAIQTECAKCNERQRKQAGKVLAHLLQYKPEYWNMLVKKFDPNNIYLRKYMADNDDDEKLSLQKLTNNTTK

>ApisCSP2

MAHLNLFVVLVASLVCFTLAEEKYTTKFDNFDVEKVLNNDRILTSYIKCLLDQGNCTNEGRELKRVLPDALKTDCSKCTDVQKDRSERVIKFLIKNRSAEFDKLTAKYDPSGEYKKKIEKFDAERAAAAKH

>ApisCSP3

MVHLNLFVVVVASLVCFTLAQEKYSTKYENFDEDKVLNNDSLLTSYINCLLDEGNCTEEGQALKRILPDALKTNCGKCTDAQKLKIEKIMKFLIKNRSIDFDRLTAKYDPSGEYKKKLEKFSA

>ApisCSP4

MDSRIALVCVVLAVFAVDQTVGAPQKDAASGPVYTTKYDNIDIDQILASKRLVNNYVQCLLDKKPCTPEGAELRKILPDALKTQCSKCNPGQKNAALKVVDRLQKDYDKEWKLLLDKWDPKREQFQKFQQFLVEEKKKGVVKF

>ApisCSP5

MNCKILIALCCVAVYAAQANPAGVATATAADEEIKDLPAYMKRFEKLNVEQVLNNDRVLASHLKCFLNEGPCVQQSRDLKRVIPVIANNSCNGCTERQITTIKKSLNFLRTKKPVEWARLVKIYDPSGVKLNKFLDA

>ApisCSP6

MNKLFLAVAFCIVTMMTVVQTAPAKYTTKYDNVNIDDILNNDRLVNSYFKCLMETGKCTPEGEEIKRWLPEAIENKCEDCSEKQKLGSEKIIKFLIEKKNDMWKQLEEKYDSKGLYRQRYSEDAKKLDIHI

>ApisCSP7

MARSSSSVTMKVFVIAVCVCAALARPEEAKMENKPAVVKSETLAAPLPTTIVKRATPYVVSTQQDSSLPNVSEDVLDKALSDRRFVQRQLKCATGEGPCDPIGRKIKAHAPLVMRGMCVKCSQSEIKQIQRVMSHIQKNYPKEYTKMLKQYQSGF

>ApisCSP8

MTNNNMNCPRSRPEIFSLLAVTTIAAVLVHQPAKVYCADGTIYPSQQQQQQTMMFTAPSGYYLSTYDNLDVGHLLRNKKVVSGFVKCFVNEGPCTPDGKLVKAYLLPEIIRTVCGKCTPRQKDMSRAVLRHLYTYRRADFDKIMQIYDTDNKKNEIINFMNQK

>ApisCSP9

MSSFCLNSVILMTVITVVVARVAFAESTTSNDRPGSDIRLVKKDVDYNEDDADDREEGFFFRISHFFGFTSYDDDKPDFITTFDLIRLLDEKYAMKQFYCVINEEPCDAVGLRLKATIPEEINRDCERCTATETSNIRRILNYVKKHYPKFWERVEPIYRNNTTA

>ApisCSP10

MVSKRFISVFMFMAVVGVSFSVPEDDDATKVVNKEVDHHSVIQEEIKKFLSMMEKINIDQILNNDRLMSNNVKCFLNEGSCTAQLREMKKMLPVLIKDSCSSCTKEQRNMIKKAMDAIKARRPNEYERVTKFFDPEKKYEKKLSEKLNES

>AgosCSP1

MNILTIFCYVTVMCDTQVKPAVSAQRLQSVNQNVTPTNDGRKTIRETSSYPTRYDYIDIEAVMNNERIIKILFNCVMSRGPCTREGLELKRIVPDAIQTECAKCNERQRKQAGKVLAHLLQYKPEYWKMLVQKFDPNNVYLRKYMADNDDDEKLSLQKLSNDTTKKKRNI

>AgosCSP2

MAHLNLFVVLIASLIYFTSAAEEKYTTKFDNFDVDKVLNNNRILTSYIKCLLDEGNCTNEGRELKRVLPDALKTDCSKCTDVQKDRSEKVIKFLIKNRSTDFDRLTAKYDPTGEYKKNLEKFEKERASAKPLKA

>AgosCSP4

MDSRIAVVCVVLAAFAVDQTVGAPQKDAVAASGPAYTTKYDHIDVDQVLASKRLVNSYVQCLLDKKPCTPEGAELRKILPDALKTQCAKCNATQKNAALKVVDRLQKDYDAEWKQLLDKWDPKREHFQKFQQFLAEEKKKGFTKF

>AgosCSP5

MHCKVLIALCCVAVYAVQASPAGTATAAAVSADDEIKDFPAYMKRFDKLNVEQVLNNDRVLASHLKCFLNEGPCVQQSRDLKRVIPVIANNGCNGCTERQMTTIKKSLNFLRTKKPTEWARLVKIYDPSGTKLNKFLDA

>AgosCSP6

MIKLILAIAFCVSITMTVVQTAPAKYTTKYDNVNIDEILNNDRLVASYFKCLMETGKCTPEGEEIKRWLPEAIENKCEDCSEKQKLGSEKIIKFLFEKKNDMWKQLEAKYDPQGTYRQRYAEEAKKLNINV

>AgosCSP7

MSRSSSSVTMKVFVIAICVCAALARPEDVKVENKPAVIKSETLAAPLPTNIVKRATDTIQLDSSLPNVSEDVLDKALSDRRFVQRQLKCATGEGPCDPIGRKIKAHAPLVLRGMCVKCSQSEIKQIQRVMSHIQKNYPKEYTKMLKQYQSGF

>AgosCSP8

MNNIIMNNSRGRYGIFSLLAVTIAAIMLVHQPATVRCADGGIITPQQQQQQTMMFTAPTGYYVSTYDHIDVGRLLRNNKVVSGYVKCFVNEGPCTPDGKLVKAYLLPEIIRTVCGKCTPRQKDMARMVLKHIYTYRQADFEKIMQIYDTDGKRNEILAFMNH

>AgosCSP9

MSAFCLNSFILMTMITVIVTHATFTRSTKFDDRTGIDIHLVKRDTDDVNDDENSVESDEGFFYRFTHFFQDSSDKEDDDDDEKKPDFITTFDIFKLLDEEYAMQQFYCVINEDPCDEVGMRLKATIPEEINRNCERCTSTERNNIRRILNYVKKHYPQFWKRVEPIYKKKI

>AgosCSP10

MINTRPRKLVRCIRGVSISVAKGDDAVNAENKDDDSHLVNREEIQRYMSMMEKINIDQMLNNTRLMSNNVKCFLNEGPCTAHLREMKKMVPMLVKDSCSSCTKEQKIMMKKAMDAVKARRPNDYEKLSKFFDPEGKYEKKFLENLNESK

>MperCSP1

MNLLAVFCYITMMCDSQLFKRLEQPAAISQVKRIEQPAMIANRIGQPTVAPRFGQPTIAPRFGLPTIAPQVGQAAITPQVGQAAIASRFGLPTVAPQVGQAAITPQVGQAAIASRFGLPTVAPQVGQAATTPQVGQAAIASRIGQNFQNANNSVSPTTDGRKTTRETSSYPTRYDFIDIEAVMNNERIIKILFNCVMNQGPCTREGLELKRIVPDAIQTECAKCNERQRKQAGKVLAHLLQYKPEYWNMLVKKFDPNNVYLKKYMADNDDDEKVSLQKLTNDTTK

>MperCSP2

MAHLNLFVVLVASLVCFTLAEEKYTTKFDNFDVDKVLNNNRILTSYIKCLLDEGNCTNEGRELRKVLPDALKTDCSKCTEVQKDRSEKVIKFLIKNRSTDFDRLTAKYDPSGEYKKKIEKFDSEKAAAAKH

>MperCSP4

MDSRIAVVCVVLAVFAVDQTVGAPQKDAVAASGPAYTTKYDHIDIDQVLGSKRLVNSYVQCLLDKKPCTPEGAELRKILPDALKTQCVKCNATQKNAALKVVDRLQRDYDKEWKQLLDKWDPKREYFQKFQQFLAEEKKKGVVKF

>MperCSP5

MNCKVLIALCCVAVYAAHASPAGAATAAAASADEEIKDFPAYMKRFDKLNVEQVLNNDRVLASHLKCFLNEGPCVQQSRDLKRVIPVIANNGCNGCTERQMTTIKKSLNFLRTKKPVEWARLVKIYDPSGTKLNKFLDA

>MperCSP6

MNTLLLAVALCIAITMTVVQTAPAKYTTKYDNVNIDDILNNDRLVASYFKCLMETGKCTPEGEEIKRWLPEAIENKCENCSEKQKIGSEKIIKFLIEKKNDMWKQLEQKYDPQGLYKQRYSEEAKKLNLDV

>MperCSP7

MDRSSSSVTMKVFVIAVCVCAALARPEDSKVENKPAAVKSETLAAPLPTTIVKRATPQVVSTQQGASLPNVSEDVLDKALSDRRFVLRQLKCATGEGPCDPIGRKIKAHAPLVLRGMCVKCSQSEIKQIQRVMSHIQKNYPKEYTMMLKQYQSGF

>MperCSP8

MTNNNMNSPRCRPEIFSLLAVAAIATVLVHQPSTVHCADAGVYPPQQQQQEATMFTAPSGYYVSTYDHMDVGRLLRNNKVVAGFVKCFTNEGPCTPEGRLAKAYLLPEIIRTVCGKCTPRQKDMARLVIRHIYTYRRGDFDKIMQIYDTDGKKNEIIDFMNQK

>MperCSP9

MTSFCLNSVILMTITTVIVAHAASTGMTAFNNRSGSDIHMAQRDYNENKADKAEGFFFTITNFFSRRKHDDDKPDFITTFDIIRLLDEKYAMKQFYCVINKEPCDATGLRLKATIPEEINNDCERCTATETSNIRRILNYVKKHYPEFWDRVEPIYRNNMTA

>MperCSP10

MVSKLFVSVFVLMSVVGVSYSVTEGDDDAAKVADKDLHPVNQEELKKFLSMMEKVDIDQILNNNRLMSNNVKCFLNEGPCTGQLREMKKMVPMLVKDSCSSCNKEQKNMMKKAMDAMKARRPNEYEQISKFFDPEGKYEKKFLENLNESK

>AglyCSP1

MNILTIFCYVTVMCDTQVKPAVSAQRLQSVNQNVTPTNDGRKTIRETSSYPTRYDYIDIEAVMNNERIIKILFNCVMSRGPCTREGLELKRIVPDAIQTECAKCNERQRKQAGKVLAHLLQYKPEYWKMLVQKFDPNNVYLRKYMADNDDDEKLSLQKLSNDTTKKKRNI

>AglyCSP2

MAHLNLFVVLIASLIYFTSAAEEKYTTKFDNFDVDKVLNNNRILTSYIKCLLDEGNCTNEGRELKRVLPDALKTDCSKCTDVQKDRSEKVIKFLIKNRSTDFDRLTAKYDPTGEYKKNLEKFETERATAKPLKA

>AglyCSP4

MDSRIAVVCVVLAAFAVDQTVGAPQKDAVAASGPAYTTKYDHIDVDQVLASKRLVNSYVQCLLDKKPCTPEGAELRKILPDALKTQCAKCNTTQKNAALKVVDRLQKDYDAEWKQLLDKWDPKREHFQKFQQFLAEEKKKGFTKF

>AglyCSP5

MHCKVLIALCCVAVYAVQASPAGTATAAAVSADDEIKDFPAYMKRFDKLNVEQVLNNDRVLASHLKCFLNEGPCVQQSRDLKRVIPVIANNGCNGCTERQMTTIKKSLNFLRTKKPTEWARLVKIYDPSGTKLNKFLDA

>AglyCSP6

MIKLILAIAFCVTITMTVVQTAPAKYTTKYDNVNIDEILNNDRLVASYFKCLMETGKCTPEGEEIKRWLPEAVENKCEDCSEKQKLGSEKIIKFLFEKKNDMWKQLEAKYDPQGIYRQRYAEEAKKLNINV

>AglyCSP7

MSRSSSSVTMKVFVIAICVCAALARPEDVKVENKPAVIKSETLAVPLPTNIVKRATDTIQLDSSLPNVSEDVLDKALSDRRFVQRQLKCATGEGPCDPIGRKIKAHAPLVLRGMCVKCSQSEIKQIQRVMSHIQKNYPKEYTKMLKQYQSGF

>AglyCSP8

MNNIIMNNSRGRYEIFSLLAVTIAAIMLVHQPATVRCADDGIITPQQQQQQTMMFTAPTGYYVSTYDHIDVGRLLRNNKVVSGYVKCFVNEGPCTPDGKLVKAYLLPEIIRTVCGKCTPRQKDMARMVLKHIYTYRQADFEKIMQIYDTDGKRNEILAFMNH

>AglyCSP9

MSAFCLNSFILMTMITVIVTHATFIRSIKFDDRTGIDIHLVKRDTDDVKDDENSVESDEGFFYKITHFFQHHDKEDDDDDEEKPDFITTFDILKLLDEEYAMEQFYCVINEDPCDEVGMRLKATIPEEINRNCERCTSTERNNIRRILNYVKKHYPQFWKRVEPIYKKKI

>AglyCSP10

MNSKIFISVFMFITIVSVSISVAERDDAVKAENKDDDSHPINREEIQRYMSMMEKINIDQMLNNTRLMSNNVKCFLNEGPCTAHLREMKKMVPMLVKDSCSSCTKEQKIMMKKAMDAVKARRPNDYEKLSKFFDPEGKYEKKFLENLNESK

**Table S3.**

>SaveOBP1

MLNLKVMMFLCLSVTVVYCEIEENRLNNNTAIEICILETNIPKDEFQAMVTMPNNPDVDILTTRAQKCMLGCVMRKNHIINDGYVSTDVLYRYVMNFYGAVPNTKRKLLSRTVSKVIDICTKKDNLPTEECVLADLIMTCVRSEALKRGLQR

>SaveOBP2

MKVSAATAVLVALVATVQSSDPCNISTCYKSGTTKPPMAVTPTRLPVQSSSTPTSHPQTTYAKDHVHGSTTIKSGANATATTASGASVNGTERPIVVKSSAGVIGNSTTPKPTMTEGHVALKQKLNTIAVKCKDELHAPQEIMALVSNTVVPQNEQQRCYLECVYKNLNLIKNNKFSVEDGKAMARIRFANQPEEHKKAVTIIETCEKEAIIDPKITEKCAAGRIIRNCFVKNGEKINFFPKA

>SaveOBP3

MISSTFYITSVFGIALLISCGYGRFTTDQIDYYGKACNASEDDLVVVKSYKVPSTETGKCLMKCMITKLGLLNDDGSYNKTGMEAGLKKYWSEWSTEKIENINNKCYEEALLVSKEVVATCNYSYTVMACLNKQLDLDKST

>SaveOBP4

MRGNYSLMVFLLFAIGLQDIFCQKQEPSGKCRAPDKAPLNLEIIINICQEEIKSALLQEALDILNDGNLEQNTPSHSSRSKREADEDLTNEERRVAGCLLQCVYKKVKAVDETGFPVVDGLMKLYNEGVQDRNYYMATLSAVRHCISIAQQLKQQQPSKSFDDGQTCDLAYEMFECVSEKIEENCGVENKSNNLSQRQV

>SaveOBP5

MSVNSATIKCIAVAVVLLQISIIFADAGHHRRGKELLDTEDSDFFRCKQASRKSCCGPENAMKRFGDKDKVAADECYAQVAEKFATVTATTPKQDLFSAEAVKITKKKQFCLHECIGKKNNLLTEDGSLNKTFIADYAMKSVFKEQWQKQVGQKALDKCLEETYIPWPAEDKENVCNPVYVQFQHCLWLQYESNCPANKIKTTKKCEKTRNRYRMQKSTSN

>SaveOBP6

MQKVVFICIFAIICQTVFTAGYDRTWILRQKRMTNDDECRTLIPSPEKKLPSCCQMPDILPNSNSTWEKCFETFKQFKDKPETKEYKEMAHGKEPPCLFQCIFMQSGLTTSDGKLNEDAITKKMSEGINNDEKWKSTWQNSLNKCFDDVKQEDKKQIPIMNTPAGRLMKCFLRDMYMSCPKNVWVESSECLNMKDLVQKCPEMPPPVFKSPPKLI

>SaveOBP7

MYNMLPKTVLFAIIAATVLKDCDAYLSEAAIKKTQQMLKTVCSKKFSVEEDVFTDIKKGIFPEDNNNIKCYFACNFKTMQLINQKGSIDKKMFKDKMTMMAPPNVLKVLLPVIEQCTGIDKGEELCQSSYNLIKCAHTVDPRSLEYLPL

>SaveOBP8

MFALKVACLCLSVAVVFGENNQQNGPSDRSASIFQSCIAETKLSGDALKGFRSMSIPKTQAEKCMMGCLMRKVNVINKGKFSVEEATKVAQKYYGTNEVMMKKAKDLIDVCAKKAQSTTEECALAGIVTTCIVEEAQKAGLAGGPGSRSRRTVSPKFRRDAM

>SaveOBP9

MIIKKTLLVSVFVLFGCLFSINKAADDADAGDKELMSKLFTVVLKCFKDADWGTCGEMITTKYDITQAKYKQCTCHMACAGEELGMINTSGQPEPAKFLEYVNKINHPSIKSQLQLIYDKCHNVKGSEKCDLAEQFAICAFKESPALKERAATLMEMLVKMKPKSK

>SaveOBP10

MEHLRSTNVVFAIVMALLVVQSSTRPQPDEMEEIKKTLYNACAGKFPITEEIKKNAKNAIISDDPTFKCFLKCCFDEMSLIDEDGILDGDSMKAMAPDHIKPIFEQVIPSCLKNVKQDGCEASFEFISCGMKLNPLTVELLPL

>SaveOBP13

MDACTVHCVFNQLEMLNSNSRPDKYSIVNIMTNQIKDVELKEFIQDSIDECFDTLELDSNNNKCEFSKNFAVCMENKAQRNCDDWDENLSANKINSAGLQDGTNQQDKRKGY

>AgosOBP2

MKVSAATAVLVALVATVQSSDPCNISTCYKSGTTKPPTTVTPTRLPVQSSSTPTSHQQTTYAKDHVHSSTATKSGVNTTATTTSGASVNGTERTTVVKSSSGVAGNVTTPKPTMTDGHVALKQKLNTIAVKCKDELHAPQEIMALVSNTVVPQNEQQRCYLECVYKNLNLIKNNKFSVDDGKAMAKIRFANQPEEHKKAVTIIETCEKEAIIDPKTTEKCAAGRVIRNCFVKNGEKINFFPKA

>AgosOBP3

MISSTFYTSLMFGIAMLISCSFGRFTTEQIDHYGKACNATEDDLVIVKSYKVPTSDTGKCLMKCMISKLGLLNDDGSYNKTGMEAGLKKYWSEWSTDTIESINNKCYEEALLVSKDIIATCNYAYVVMACLNKQLKLDNST

>AgosOBP4

MRGNYSLVVFLLFGFGLLEIYCQKQELSGKCRAPDKAPLNLEIIINICQEEIKSALLQEALDILNDGTLEQNTPSYSRSKRDADEDLSNEERRVAGCLLQCVYKKVKAVDETGFPVVDGLMKLYNEGVQDRNYYMATLSAVRHCISIAQQLKQQQPSKSFDDGQTCDLAYEMFECVSEKIEENCGVENKLNNLSQRQV

>AgosOBP5

MKMSANGATMKCVAVAVVLFQMSVIFAEAGHQRRGKELLDTEDSDFFRCKQASRKSCCGPENAMKRFGDKDKVAADECYAQVAEKFATVTATTPKQDLFSGEAVKITKKKQFCLHECIGKKNKLLTEDGSLNKTFIADYAMKSVFKEQWQKQIGQKALDKCLEETYIPWPAEETENKCNPVYVQFQHCLWLEYESNCPDNKIKLTKKCEKTRNRYRMQKSPSNQ

>AgosOBP6

MQKVVFLCIFAIICQTVFTVGFERTWILRQKRMTNDNECRALFPSPEKKLPTCCQMPNILPGLDNAWEVCFEKFKQFKDKHATKEYKEMVHENEPPCLFQCVFMQSGLTTSDGKVNEDAVIKKMAEGMDNDEKWKSIWRNTFNKCLNDVKQEDKEQIKVMNTPTGRLMKCFLRDLYMNCPKNVWVENSECSNLKDLVEKCPKLPPPVFQSPPKLI

>AgosOBP7

MNMLPATVLLAVVAATILKDSDAYLSEEAIKKTQKMLKNVCSKKHSVEEEVFTDIKKGIFPENNNNIKCYFACNFKTMQMVNQKGILDKKMFKDKMTMLAPPNVLAILLPPIEQCIGNDKDTEICQSSYNFIKCAHRVDPKSLEFLPL

>AgosOBP8

MFAFKVACLCLSVAVVFGENNQQNSNDRSASIFQSCISETKLSGDALKGFRSMSIPKTQAEKCMMGCLMRKVNVINKGKFSVEEATKVAQKYYGTNESMMKKAKDLIDVCAKKAQSTTEECALAGIVTTCIVEEAQKAGLTGGPGSRSKRTVSPKFRHSIV

>AgosOBP9

MIIKKTLLVSGFVLFGCMFSINKAADDADTADKELMSKLITVAFKCFKDADWGTCGEMITTKYDITQAKYKQCTCHMACAGEDLGLINSNGQPEPAKFLEYVKRINNSVIKSQLQHIYDKCQNVKGTEKCDLAEQFAICAFKESPEMKERVTKLIEMLVKMKPKSK

>AgosOBP10

MEHLRGTNVMFAIVMALLVVQSSTRPQPDEPDDIKKTLYNACSEKFPLTEEIKNNVKNSMVIDDQNFKCFLRCCFDEMSLIDEDGIIDGESLAAMAVDKIKPVAEKIVHDCLPAGKQEKQDGCEAAFKFFSCGMKLNPLTIELLPLQ

>MperOBP2

MKVSAATAVLVALVATVQSSDPCNISTCYKSGTTKPPMNVTPTRLPVQSSSTPTSHPQTTYAKDHAHGSTTVKSGANATATTASGASVNGTERPAVAKSSAGVTGNSTTPKPTMTEGHVALKQKLNTIAVKCKDELHAPQEIMALVSNTVVPQNEQQRCYLECVYKNLNLIKNNKFSVDDGKAMARIRFANQPEEHKKAVTIIETCEKEAVIDPKTTEKCAAGRVIRNCFVKNGEKINFFPKA

>MperOBP3

MISSTFYITLLFGIAMLISCGYGRFSTEQIDYYGKACNASEDDLVVVKSYKVPTTETGKCLMKCMITKLGLLNDDGSYNKTGMEAGLKKYWSEWSTEKIEAINNKCYEEALLVSKEVIATCNYSYTVMACLNKQLDLDKST

>MperOBP4

MRGNYSLTVFLLFVIGLQDIYCQKQEPSGKCRAPDKAPLNLEIIINICQEEIKSALLQEALDILNDGNLEQNTPSYSSRSKREADEDLTNEERRVAGCLLQCVYKKVKAVDETGFPVVDGLMKLYNEGVQDRNYYMATLSAVRHCISIAQQLKQLQPSKSFDDGQTCDLAYEMFECVSEKIEENCGVENKSNNLSQRQV

>MperOBP5

MSANSATIKCIAVAVVLLQISVVFADAGHHRRGKELLDTEDSDFFRCKQASRKSCCGPENAMKRFGDKDKVAADECYAQVAEKFATVAATTPKQDLFSADAVKITKKKQFCLHECIGKKNRLLTEDGSLNKTFIADYAMKSVFKEQWQKQVGQKALDKCLEETYIPWPAEDKENVCNPVYVQFQHCLWLQYESNCPDNKIKITKKCEKTRNRYRMQKSTSN

>MperOBP6

MQKVVFICIFAIIYQTVFTVGYERTWILRQKRMTNDDECRTLLPSSEKKLPSCCQMPNILPGLDSTWEKCYEKFIQFKDKPETKEYKEMSHGKEPPCLFQCIFMESGLTTNDGKLNEDAITKKMTEGINNDEKWKSTWKKSLDKCFDDVKQEDKKQILIMNTPAGRLMKCFLRDIYMNCPENVWVESSECLNVKNLVQKCPEMPPPVFQSAPKLI

>MperOBP7

MNNMIPATVLLAVIAATVLKDCDAYLSEAAIKKTQQMLKTVCSKKHSVEEDVFTDIKKGIFPENNNNIKCYFACNFKTMQMINQKGTLDKKLFKDKMSMMAPPNIYNILLPAIEQCIGIDKGEELCQSSYNFIKCAHRVDPKSLEYLPL

>MperOBP8

MFVLKVACLCLSVAVVFGENNQQNSSDRSATIFQSCIAETKLSGDALKGFRSMSIPKTQAEKCMMGCLMRKVNVINKGKFSVEEATKVAQKYYGTNETMMKKAKDLIDVCAKKAQSTTEECALAGIVTTCIVEEAQKAGLAGGPGSRSRRTVSPKFRRNSM

>MperOBP9

MLIKKTLLVSVFVLFSCLFSINKATDDADTADKELMSKLFTVVFKCFKDADWGTCGEMITTKYDITQAKYKQCTCHMACAGEELGLINSSGQPEPAKFLEYVNRINNPGIKSQLQHIYDKCQNVKGTEKCDLAEQFAICAFKESPALKERATTLMEILMKMKPKSK

>MperOBP10

MEHLRSTNVVFAIVMALLVVQSSTRPQPDELEEIKKTLYNACAGKFPITEEMKKDILNSNMVDDQNFKCFLRCCFDEMSMIDEDGIIDGESLISMATDNLKPVIQQVVQSCVKDIKQDGCEAAFNFISCGLKLNPMTIQLLPL

>ApisOBP1

MLNLKVMMFLCLSVIVVYCESDQVPINSSAAVESCLLETNMTRDEFEDMLTSPNARELTILKSHAHKCMFGCVMRKNHIVNDGVVSKEVLSKYVLNFYGRPDYKRRLIIKDVEHIVDVCAKKVADESETDECELAATLVTCIVLEANKAGLVDDPARQI

>ApisOBP2

MKVSAATAVLVALVATVQSSDPCNISTCYKSGTTKPPMSVTPTRLPVQSSSTPTSHPQTTYAKDHSHGSTTTKSGANATATTASGASVNGTERPAVVKSSAGVTGNLTTPKPTMTEGHVALKQKLNTIAVKCKDELHAPQEIMALVSNTVVPQNEQQRCYLECVYKNLNLIKNNKFSVEDGKAMAKIRFANQPDEHKKAVTIIETCEKEAVIDPKTTEKCAAGRVIRNCFVKNGEKINFFPKA

>ApisOBP3

MISSTFYLTSLFGIAMLISCGYGRFTTEQIDYYGKACNASEDDLVVVKSYKVPSSETGKCLMKCMITKLGLLNDDGSYNKTGMEAGLKKYWSEWSTEKIESINNKCYEEALLVSKEVIATCNYSYTVMACLNKQLDLDKST

>ApisOBP4

MRGNYSLMVFLLLAIGSQDIYCQKQELSGKCKAPDKAPLNLEIIINICQEEIKSALLQEALDILNEGNLEQNTPSYSSRSKREADEDLTNEERRVAGCLLQCVYKKVKAVDETGFPVVDGLMKLYNEGVQDRNYYMATLSAVRHCISIAQQLKQQQPSKSFDDGQTCDLAYEMFECVSEKIEENCGVENKSNNLSQRQV

>ApisOBP5

MSVNSLTIKCIAAAVVLLQISVIFADAGHHRRGKELLDTEDSDFFRCKQASRKSCCGPENAMKRFGDKDKVAADECYAQVAEKFATVTATTPKQDLFSADAVKITKKKQFCLHECIGKKNHLLTEDGSLNKTFIADYAMKSVFKEQWQKPVGLKALEKCLEETYIPWPAEDKENVCNPVYVQFQHCLWLQYESNCPANKIKITKKCEKTRNRYRMQKLTSN

>ApisOBP6

MQKVVFVCIFAIICQTVFTVGYDRTWILRQKRMTNDDECRTLIPGPEKKLPSCCQMPNILPNMDSTWEKCFETFKQFKDKSETKQYKEMAHGKEPPCLFQCIFMQSGLTTSDGKLNKDAITKKMSEGINNDEKWKSTWQNSLNKCFDDVKQEDKKQIPIMNTPAGRLMKCFLRDMYMSCPKNVWVESSECLNVKDLVQKCPEMPPPVFKSPPQLI

>ApisOBP7

MVAQKRMYNMLPTTVLFAVIAATVLKDCDAYLSETAIKKTQQMLKSVCSKKHSVNEDVFLDIKKGIFPEDNNNIKCYFACNFKTMQLINQKGSIDKKMFRDKMSMMAPPNVFNILSPVIEQCTGIDDGKELCQSSYNVIKCAHRVNPKSLEYLPL

>ApisOBP8

MFALKVAYLCLSVAVVFGENNQQNSNDRSATIFQSCISETKLSGDALKGFRSMSIPKTQAEKCMMGCLMRKVNVINKGKFSVEEATKVAQKYYGTNETMMKKAKDLIDVCAKKAQSTTEECALAGIVTTCIVEEAQKAGLSGGPGSRSRRTVSPKFRRNVM

>ApisOBP9

MIIKKTLLVSVFIIFGCLFSINKAADDADAADKELISKLFTVVFKCFKDADWGACGEMITTKYDITQAKYKQCTCHMACAGEELGMINSSGQPEPAKFLEYVKRINNPDIKSQLQLVYDKCQNVKGSEKCDLAEQFAICAFKESPALKERVATLMELLVKMKPKSK

>ApisOBP10

MEHLRKTNVVFGVVIVLLVIQKSSTRPQPDELEEIKKTLYNACAGKFPITEEVKNNAKNSIFLDDQNFKCFLKCCLDEMSLIDDDGIIDGDSLKAMASDKIKPILEQVVPNCLKDVKQDGCEAAFDFLSCGIKLNPLTVELLPL

>ApisOBP13

MENGVTDFTHVSHGNTGSRSKNHRQGSYPSADYDSGQSNSNYNRQSLPTRRYRRDDSNEKSKRQKAAVTGSNNRLLGNNRFRNMTKTGGNQPGKGTYLDKMDACTIHCVFNQLEMLNSNSRPDKYSIVNIMTNQIKDVELKEFIQDSIDECFDTLELDSHNNKCEFSKNFAVCMENKAQRNCDDWDENLSANKINSAGLQDGTNQQDKRKGY

>AlucOBP1

MCSKYFVMLIGLTVYTSAEVINEECKDRNQSSTEYETFYNCCDLESSFNETKSKEKEEAREFCENEFEKANNVSEDEAEPSPSSVRQDCYVDCILKKLGAMSEDYKMDKEKVTKWFMEGTHKDFEEVGKQAMEKCYDKTYSKKHCASRVMGLLWCYSEELVMNCPAKYWDQSEKCTAAKAYMKKCSTNPWRSED

>AlucOBP2

MRSTGSECFEEIDAKLGNKTSLESDMDPYNCEKVKRMKKRHYCMHECKAKKLGVATEEGNLEFPKVKELLLSRVNETWQKDILGQAADTCATSKFDQTWKDDTEEYKCNPQALQFKHCVWKQVEMKCPEEHQNTGRHCKKLRSKISSETSKDIAKETSV

>AlucOBP3

MFSSATLVCLFAVALTQGQLDEDPECRPPHPPGKDDKCCTIPELIVGENMQAMMKQCFEESGMERRPPGPPGSGTPPTPEEIEAHRSAHECVDECFFKAAKFMNSDGEFDLEAMKTAAASVFTGDWAPLGSETIDKCFASAKSQVSASAKCTSGAHRAKKCILRNFIINCPPSAWNDSTDCAALKARLTKCSNAMPPFPHHKH

>AlucOBP4

MEVAACLVLLAALAALTAAVEEGRPLCKAPTTAPRKLEKVINQCQEEIKYALLQEAPSVLGETVGLKTALTRNRSKRETFTGEERRIAGCLLQCVYRKMKALDETGFPTATGLVKIYSEGVEDRNYYLATIQGVQRCLSRELQSRNTNPSIVKAEGYSCDVAYDMFNCVSEQIEQLCGTSP

>AlucOBP5

MNSIIVLCLVASAVTLSQGNPTTPNPSTSHVSSSAGITVSGVSKSPEEIKLKIKEQVATLTGACKTQTKLTGEQAKIVASQAIPKTEAEKCFLECIYQGLQLTKDGKFNEPAARAWAQKRFGNAPEDLQKANTMIDICVKEVVVKDENEKCALGRLIRECFVKNGAKINFFPKP

>AlucOBP6

MYDRFKLFALLALVVSCKSAPPEEPAECKLPESDSAELVKCCKLNVVLDEMADSVGECMKLVKGKPEKGPPVPEGFDCMDTCVFSKLGFAANNKLDAEKLTKKFSELFKGDWSALSDSTLKKCLPMAEGAKGSCASGADVFKFCIVRELYMNCPASSWTKSDLCKANVERLEKCPHSMPFLPGTGIKKN

>AlucOBP7

MNPLILILLVVFAAATRGEEQANALVAKAFNKCFGEFPLGDDEMKEVKDKSTVPSSHNAKCLMACMLKEGRILRGGKYELENAILMADVLNKNDHAATDKAKQLIETCAAQVGTDASADECEFAYKMALCASDEAKKLGVRPPDF

>AlucOBP8

MVLKMKQILVVFVALQVLISTTEAVMTQAQMKQAMKTVRNMCIPKSGVDKEALAKMVEGEFDESDQKLKCYLGCVLGMMQAVKNNKINLTMVKNQISKMLAPEQGQRILAAFEGCATVTGDDNCDLAFKFAKCIYDTDKELLFQAFIVP

>AlucOBP9

MKSFVGLIFAVALVEFASAITKEYHDRAVAAKDACLKKHPSIKESDVQEFLKKHKLPETDDGKCMIACYMEEMNLMADGKINVEEAKKTNSDKYDGEPDNKELADKLIDHCSSQVSPDGMSKCEYAYQISKCGLEYGMKNGLTPPKMYEEQRR

>AlucOBP10

MTYHVFFRKFDLPRISRRVRQCYYHSVPRSLSGSSRRMLEETSQHHPKRRSRVSEKHKLPETDDGECMIACYMEEKNLMADGKINVKEANQTNSDKYDGEPDNKQLAEKLIDHCSSQVSPDGMSKCEYAYQFSKCGLEYGMKNGLTPPKMYEEQRR

>AlucOBP11

MGSQYERTLVGVRYLPIMKRVKFILVLSLLSRCSSAPTDDMAACMQITNEDSASMATCCDYVIPFSNKTMTTCDKKETSGEMSKEFECVQDCLFSSDNVLGADKKFDPVAWRKHATNTISGDWKGVIANSGSNCEGFKKVLAQSMEKKCPTSESDVSFNCMTLQWYMNCPKSAWTSSESCEASKKKLMSCFGPIFENTS

>AlucOBP12

MTCSHFIALLSVVALSLSSGEINEECKDIENLKTQLENFYGCCDFESMIERVVRTEEEVETDRFCREERKKINSTDGKVPLASEGHDCFMECVLKRMGAMGQDFKFIREKLDDFFLRGYPEEVKQAGKLAFDKCLSKNFSKKYCASGINGLMMCLPEELVMNCPANIWSSHESCPIAKEAIKKCPSYRVMIEQE

>AlucOBP13

MKHSSCVVPVALTIFVVAIVSGFKELDDVLPKPKQDECRKESNFQAELPSDINQNITQELKCFAACSLVKLGLMNEKDGTINMAQLEDLIAKHTGGKDAADMFKHTVVEPCMKEVNKTTDYCEYSFQLVKCGMSKVKPPSTGTEG

>AlucOBP14

MALNAKAVLLLGVCGLVYVSAYQEVLKATLKDCKGGKEITQEEVDEFMKPLIPKNEEERCLMACVFRAYNVIVDGHFDPKLAYGVAKNILHENPEKLKHIKETLDYCGHEIPTKMDNECDLAGEVMSCRNKYNIDHGYDQDP

>AlucOBP15

MMRPTAYYLFASYAALLVCVHFASVSAITPELDKRAKAAVAKCADVPRTDEAKKEDCHAGCFMSAMGYMTNGEINVKNMEEANKQKWDDQEIIKKGIQVDTTCAKQVGDTKGKSECTIGYEFSTCKKELVKKVGLPPPTPLKE

>AlucOBP16

MKRLVFVLFTLCSLQWVSGITDELKQKAQAARLTCKQQVGLSDKEFNDWVKGIALPTTDGGTCCEVCACWMRELGYLTGGRVNLENMKAVNAQKWNNLAYVELGNKIDALCSDRVLQTGRKECEIAVDFRKCKTELIQQFGGPPKPGST

>AlucOBP17

MRILVLFTAALTCVLAGELPEEMREMAQGLHDSCVGETGVDNGLIAPCAKGSFADDPKLKCYFKCVFGNLGVISDDGELDAEAFASILPDNMQALLPTIRGCGSTTGADPCDLAMNFNKCLQKADPVNFMVI

>AlucOBP18

MHAAIVLIGSALLVAYVSGAPSANVKEIVQNVSKKCAAETKASPDQAKIVLSKNIPKDDAERCFLQCVYTGVGVIKDGKFSEEGGKKLVALRFHDAKEKELANKLIATCAKEIKAKDGEKCSLGRAVRECFVNHGKQVNFFPSA

>AlucOBP19

MNSRFGIVFASLALLHITNAGNIKEGYVAKIAEIKDKCLKEHNVDHSVVEDLLKKSIKPEVKAAQCMVACFFEENGMMKDGKIVSEMVKSNNAHQYEDPADVEKANEASDMCDGEVSTDGKDKCLLAADYALCWVKRTEEAGLPQIDFANSS

>AlucOBP20

MYTFKTFFVLTLASYVIAAPPADEPAECKPMKEKEEEISKCCKLEPVTVKEQAAFVDCMKLVKDTDKKGPPKPEGFECLDDCILSKTGSLGSDKKIDPAKINAAAKTTYTGDWAEPGAKMVEKCLAQVAENKDKTVCSTSGADVYTKCIFRESYINCPEKSWTNSDACKANKERVIKCPKTLPYNAEQHKAETR

>AlucOBP21

MKFFVVSAALVLLVAAAVKANEKKANEKVTEIFNKCKETWPVTDEEIEQVKQKQSIPDSKNVKCILACMLKEAKILRDGEYNKDNAELMADVLYKDEPEHAEKSKQIIEMCSSELGTKTEGDDCEYAYKMSVCASKHAKELGVKTPEF

>AlucOBP22

MSLKIHFFVFAAIGAACVCAYQDQLKQTIKDCQGGKEVTDEELEEFTKPLIPKNEEERCIMACVMRTYNIINNGHYDPKIAFGIIKGILKDHPEKLDRIKEVMDHCGEDVPQHMDNECDLAGEIMQCEVKYQKAMGLN

>AlucOBP23

MYVFTVALSFALLNIVFTHPGHFDEDPECRPPHSHRHEEKECCKTPNLFSKNKDEMHELVHKCFEEAGIKKPHHGHHGPPPPGDEPPPPPPPPFHSKNNTKFECVEQCFLKNLELIDEEGDLKIDDFKALVGEKYTGDWASVGSAALEKCLEKTKTEEKESSKCKAGSKHVLLCIARESFINCPASDWTESEVCSDAKERVVKCPDIPPPMNH

>AlucOBP24

MSTKLRSVGMILAIAITHVCAYQEQLKETIKQCQDGREVTDDEVEEFTKPLVPKNQEERCLVACVFKEYKVIIDGHFDPVNALNVAKMVYKDYPEKWKRIKDVIDHCGEDIPTHNDNECDLAGDIMNCEVKYLNSMPKGVSLELLAGSIAATAEP

>AlucOBP25

MFTSTIFAVFLFSVALTQGQMDDDPECRPPPPPNKEGSCCTVPRLLDNADKPEVIKKCHDEAGMKRPSGPPGSGTPPTAEEMAAHKSAHECADECIFKSSNLLKSDGELDQDAIKATTTKMFTGDWSTIASTAVEKCLATAKSEVGASAKCKSGAHQMVKCFARTMFLNCPASSWTESTECAAAKTRLTKCPNAMPPPPHHSRH

>AlucOBP26

MNPTVAIIFTLLVAYVKANTKELSPSEALKQKVKVQCQQEVKATPEQLKIYDNFKDVPKDDVENCLMECMYTKTGGIGADGKYSVEGFKKLVDMKYKGEENTKARKIAADCEAKAAPKEGEKCSMGRAIRECLAAATKENEFFTI

>AlucOBP27

MARKFIKSCYTLVALLVFVGSIHVEAKELTEEQRTQLFEDLKQCKNSTDLSDDEFETIIAKKELPTSEAGKCFTKCLMEKLDIIEDAEGGKKKISVITMQASLEENMEKEDDIAKGKDIIQKCGDTVEPEDSCAYAYNISKCIYDRMKEAGISQ

>AlucOBP28

MIIEIICVLTVGISPHFIEGQELPPPGGVGNKTAVFKESFIRTAKYCSSIHETSTVAVLAILMSEESDDQNGKCFLNCMLQRYQLMSKQGAYNKDKFKPFLDYIPESRFLQSIKGNLKTCITERDPAPCEKAYKFIKCFYTRARNKDEFGKIQRK

>AlucOBP29

MNRPLLLLTAVLAVGSGQQEDCKTAPAGWPRRPPQCCDLPFPLEGMKKEFGSCIRQIGNRQSSAVPTAQAVRDARLCIEECVYKGLGFMEEHNLNKDQILQQLTKGVADKKDWTKPMEDAVKSCHETITKRETPQEGTCKDSAHEFTHCVMRQLFLSCPASEWNNNDECNLVKSRMQACPNIPPPPPPPPQGFRGQGPPPPQ

>AlucOBP30

MNAHIVLCLVASVFALSQGTPTTPTPATSRRVTVAPEDLEQAKSLRKFCTAKTGFTGITTTETSKGKDQARTATTPRPKTQLEKCYLECLYTGLQLTKDGKFNEPGARALANKRYKNAPEELRKVNSIIDFCITEVVVRDIEEMCALGRLIKECFSKYGAKNFPEL

>AlucOBP31

MFTSATFTVFLFAVTLTRGQIDEDPECRPSGPPGKEPECCTIPMKLFGDEVQEAVVKNCFDEAGMKRPSGPHGGGSPPTAEEMAAHISAHECADECVFKSGNFIKSDGGLDEDAIKAVIAKLFTGDWAPIATAAVNKCLASAKSGVSASAKCKSGAYQLSKCFQRELFLGCPASLWTESTDCSAIKARITKCPNAKVPIGHHHKH

>AlucOBP32

MSGRHSLILVLLAAVTSAEVLTDGDCPKTMPKEMKPLYKCCVVEMDSNKTISDDQKAAVDSCVNTSKSDSDANKHDCMIECIFIKLGYMGEDKTINVDYVLKEMNSLLPEDFHEQTSKSLATCMGKKFSSTECPSEIDGVMACFSTMVLMNCPAKHWTDDEECKATRKFFQKCGDSIGYRYD

>AlucOBP33

MHPWKTTCLIGMTAALMVVTAFAGLPFQNEMAVMQCKVKFDVTAEDIQLLKDSKLPSSHSGKCMMACILKKMKVMTKRGQFDLRNVQKWLRNKYQGDQANLAKGNYVAEACANTLPTLGIQDECEMAAEIMTCVRTKSKLVKKTLNGELPKEVSP

>AlucOBP34

MEHWKWRLALLIFGMVTCVPQLEGAQKSKQPSKAKTKESQVVAARPKDARAAACVTQIGPDEEEEASFYRKEIPETDKGKCLLACYLESKGVLSGGKFSSSGAAKIAARAYPNNAAKTGNVKHILSHCGTIAARETEQCQLAYRLAECTTTLADKFKL

>AlucOBP35

MINVVFVLLIGTGIVSGGFMEALIECKQQHHVSKEEAMTGESEEVKCFSECVLKKSGMMSDNNEFDEEKIQAEGARMIKNDEQKNREFEGAAKACIEKVNGENPSEKCAKGHALFKCMKEAMPMSKMRG

>AlucOBP36

MKTFVGLIFAVALVEFASAVSKEYHDKAIAAKNTCAKLHNVDDETIMKFWKAHQLPEKEPETCIIICYMKEMKLVVDGKVDADAWKASNKEKWDDEKHVAAADEIVDKCSAEVPPTENECEWGLALTKCALKHGKEAGIPPPDMEHPKRR

>AlucOBP37

MDTHFGLLIASLAILHTANAVINKDYLEKVVTAKDKCLKEFNVDDSVVEDFIVRYNKPQSESGKCMVACYMEERGMMKDGKTITEQVMLDNQEKWIAATHVNMGKEVIDTCDKEVPNEKNDKCDLAVDYMMCLVKRGDEAGLPKMDVAQLKH

>AlucOBP38

MGFKFVKYRSYFFVLVIHIILCIQIKAKELTDEQKEQIFAEIKNCMESTKLTDEEFESIMAKKELPTSKEGKCFTKCLMEKMEYLEEGGKINVIAVQAGLEENMEKESEITKAKEIIQQCADTVPPEDSCEYAYGISQCMYTKMKEAGISGGP

>LlinOBP1

MRILVLFTAALTCVMAGELPEEMREMAQGLHDGCVEETGVDNGLIGPCAKGNFADDQKLKCYFKCVFGNLGVISDEGELDAEAFGSILPDNMQELLPTIRGCAGTTGADPCELAMNFNKCLQKVDPVNFMVI

>LlinOBP2

MVLKMSLLLVVFVASQVLISTTEAYMSQAQMKQAMKTVRNMCIPKSGVAKEALAKMVEGEFDDSDQKLKCYLGCVLGMMQAVKNNKINLTMVRNQISKMLAPEQGQRILTAFEGCATVTGDDN

CDLAFKFAKCIYDTDKELLFQAFIVP

>LlinOBP3

MAVNAKAVLFLALCGLVYVSAYQEVLKATLQDCKGGKEITQEEVDEFVKPLIPKNEEERCLMACVFRAYNVIVDGHFDPKLAYGVAKNILHENPEKLKHIKETLDYCGHEIPTKMDNECDLAGEVMACRNKYNKDHGYDQDP

>LlinOBP4

MSIKIHFFVFAAIGLACVCAYQEQLKQTIKDCQGGKEVTDDELEEFTKPLIPKNEEERCIMACVMRTYNIINNGHYDPKIAFGILKGILKDHPEKLNKIKEVMDHCGEDVPQHMDNECDLAGEIMQCEVKYQKAMGLA

>LlinOBP5

MTTKLRSVGMIVAVTIAYVCAYQEQLKATIQKCQDGREVTDDEVEEFTKPLIPKNEEERCLVACVFKEYKVIIDGHFDPVNALNVAKMVYKEYPEKWERIRDVIDHCGEDIPTHNDNECDLAGDIMKCEVKYLNSMPKITSLELLAGSIAATEEP

>LlinOBP6

MKFVLSAAVVLLVAAAVKANEKKANEKVTEIFNKCKETWPVTDEEIEQVKQKQSIPESKNVKCILACMLKEAKILRDGEYNKENAELMADVLYKDEPEHAXKSKQIIEMCSAELGTKTEGDDCEYAYKMSVCASKHAKELGVKTPEF

>LlinOBP8

MNPLIPVLLVVCAAATRGDEQTNAMVAKAFNKCHGEFPIGDDEMKGVREKSTVPDSHNAKCLMACMLKEGKILRDGKYEKENAIVMADVLNKDDPAAADKAKQLVETCATQVGSDASADECEFAYKMAVCAAGEAKKLGVRPPDF

>LlinOBP9

MNPLIPVLLVVCAAATRGDEQTNAMVAKAFNKCHGEFPIGDDEMKGVREKSTVPDSHNAKCLMACMLKEGKILRDGKYEKENAIVMADVLNKDDPAAADKAKQLVETCATQVGSDASADECEFAYKMAVCAAAWSSSTRFLKTHPFLLQLCTHRSTWHTFKYEYLPGNTSSRDLTHHINIVCVT

>LlinOBP10

MTPIVAILFALLAAHVKANTKELSPVEVYKHKIHEECIKETKATPEQAKIVFNYKDVPKDDGEKCFMECVYKKSGGIDANGKYSIEGFNKLVDMKYKGEENAGAKMIVKDCSSKVAPKEGEKCSVGRTIRECLSAASKENEFFTI

>LlinOBP11

MHAAFVLIGSALLVAFVSGAPSANVKEIVQNVSKKCVAETKASPEQAKIAVSQHIPKDDVERCYLQCVYTGVGVIKDGKFSEEGGKKLVALRFHDAKEKELANKLIATCAKEIKAKDGEKCSLGRAVRECFVNHGKQVNFFPSA

>LlinOBP12

MVAECPAYSWVCQSTLSYYLHLQQTLQYIDRDPTLAMNQSSCILTLALTIFVMVVVSGFKELDSVLPQAKQEECRKESNFQGELSGDVSQNVTQELKCFAACSLVKLGLMNEKDGTINTTQLDELIAKHTAGKDAADMFKHSVVEPCLKEVNKTADYCEYSFQLVTCGMNKVKPPTTG

>LlinOBP13

MMKIAFVISVLVVLATVSAITPELDKKAKEAVAKCADVPGINEAKKEDCYAACFMTEMGYMTDGKINVENMEEANKQKWDDQQMINKGIEIDKTCAKQVGDTKGKSECAIGYDFGVCKTRLVKANCILRSILQTQLVPSPWWAHLPPLTPLKDSSTFNASFLKGVYREDMEQRNYNKTGLQPPTPLKQ

>LlinOBP14

MKIAFVVSVLVVLATVSAITPELDKKAKEAVAKCADVPEINEAKKEDCYAACFMTEMGYMTDGKINVENMEEANKQKWDDQQMINKGIEIDKTCAKQVGDTKGKSECAIGYDFGVCKTRLVKANCILRSILQXQLVPSPWWAHLPPLTPLKDSSTFNASFLKGVYRGDMEQRNYNKGKNKTGLQPPTPLKQ

>LlinOBP15

MMKIAFVTSVLVVLATVSAITPELDKKAKEAVAKCADVPGINEAKKEDCYAACFMTEMGYMTDGKINVENMEEANRQKWDDQQMINKGIEIDKTCAKQVGDTKGKSECAIGYDFGVCKTRLVKATGLQPPTPLKQ

>LlinOBP16

MKRLVFVLVTLYLLQSASGITDELRKKATEARLKCKQQVGLSDKEYQDWVKGISLPITNGGSCCEVCACWMRELGYMTDGHLNLNNMKNVNTQKWSEKANVEKANQIDTLCTARVVQDGRKECEIALDYRKCKTEMIKQNGGPPKPGST

>LlinOBP17

MKRLVFVLVTLYLLQSASGITDELRKKATETRLKCKQQVGLSDKEYQDWVKGISLPTTNGGSCCEVCACWMRELGYMTDGHLNLNNMKKRQDKYKHS

>LlinOBP18

MRSTGSECFEEVDAKLGNKTSWESDMDPYNCEKVKRMKKRHYCLHECKAKKLGVANEEGVLDFPKVKDLLLSRVNETWQKDILGQAADTCANSKFDQTWKDDTEEYKCNPQAIQFKHCVWKQVEMKCPEEHQNTGRHCKKLRSKISSETSKDSTAKETSV

>LlinOBP19

MKSFVGLIFAVALVEFASAVTKEYHDRAVAAKDKCAKEHNIKESEIQEFVKKHKLPETEDGKCMIACYMEEMKLITDGKVNVDEWKKSNKEKWDEEAHVAMADEIVDKCNEQVSPDGLAKCEYGFKLTECGLKHRLEKGLPAPNMDDVKRR

>LlinOBP20

MKLVKDDKARPPKPEGYECIDDCIMAKNGFLGTDKKIDAAKVNAAAKTSYTGEWAEPGAKMVEKCLAQVSANKEKGECTSGADIFSICMFRESFINCPEKSWTSSETCKANKERLIKCPKSIPFLNKSAK

>LlinOBP21

MLTAYMIVATLSVFFFAVALTQGQMDEDPDCRPPHPPGKEAQCCPLPDFVGVVDNFHDVMHKCSDEAGLRKPSGPPGSGTPPTAEEMAAHMSAHECADECLFKNTKYLQSNGELDKDAIKASVTKIFTGDWAALASSAADKCLASAKSEVGASAKCKSGARQMVKCFTRAMFLNCPASSWTESTECAAAKARITKCPNAMVPMIPPYPQPISANSTILDSLFACITDGEYMEFQSISSGRLG

>LlinOBP23

MYAFTAALSFFLLNIAFAHPGHFDEDPECRQPHHHRHEENDCCKVPSLFSNNKDEMHELVHKCFEEAGIKKHGPHHEHHGPPPLEDGPIPPPPPPPFSPKNDSKFDCVEQCFLKNLDLVDDEGDLKVDDLKALVTEKFSGDWASVGSSAIEKCLEKAKTEENEPSKCKAGSKRVLHCLAREFFMNCPASDWTESEVCLAAKDRVSKCPHSLPPMHH

>LlinOBP24

MADSVGECMKLIKVKPEKGPPVPEGFDCMDTCVFSKLGFIGADNKLDPEKLAKKFSELFKGDWSALSESTLKKCLPMADVGKGVCSSGADVFKFCLIRELYMNCPASSWTKSDLCKANVERLEKCPNSLPFMNGSGLKNKSSR

>LlinOBP25

MFTTATSTIIFLFAVALTRGQMDEDPECRPPHPPGKAGDCCVQPKLFDEGDMPDVIKKCHEEAGVKRPSGPPGSGTPPTAEEMAAHKSAHECAAECIFKNNNFIKSDGELDXDAIKATVTKMFTGDWATLASTTIDKCLASAKSEVDASPKCKSGADQVVRCFGRSLFIGCPASAWTESTECAAEKARLTKCPNAMPPPPHHKH

>LlinOBP26

MNQSSCILTLALTIVAMAVVSGFKELDSVLPQAKQEECRKESNFQGELSGDLSQNVTQELKCFAACSLVKLGLMNEKDGTINTTQLDELIAKHTEGKDAADMFKHSVVEPCLKEVNKTADYCEYSFQLVTCGMNKVKPPTTG

>LlinOBP28

MEVAACLVLLAALAALTSAVDEKRPLCKAPTSAPRKLEKVINQCQEEIKYALLQEALSVLGETVSLRTALTRNRSKRETFTGEERRIAGCLLQCVYRKMKALDETGFPTATGLVKIYSEGVEDRNYYLATIQGVQQCLSRELQNRNKNPSIVKAEGYSCDVAYDVFNCVSEEIEQLCGTSP

>LlinOBP29

MNRPLLLLTAVLAVGSGQQEDCKTAPAGWPKRPPQCCDLPFPLEGMKREFGSCIRQIGNRQSSAVPTAQAVRDARLCIEECVYKGLGFMEEHNLNKDQLLEQLKKGVAGKKDWEKPMEDAVKSCHETITKRETPQEGACQDSAHEFTHCVMRQLFLSCPASEWNNNDECNLVKNRMQACPNIPPPPPPPPQGFRGQGPPQPQ

>LlinOBP30

MNSFTVLCLVASVVALTQGNPTTPNPTSSSHAASVSGGSTVSGVSKSPEEVKQKIKEQVEALTGACKSQTKITGEQAKIVATQAIPKTEAEKCFLECIYTGLQLTKDGKFNEPAARALAQKRFGNAPEDLTKANSMIDTCVKEVVVKDLNEKCALGRLIRECFVKNGAKINFFPKP

>LlinOBP31

MLTAYMIVATLSVFFFAVALTQGQMDEDPDCRPPHPPGKEAQCCPLPDFVGVVDNFHDVMHKCSDEAGLRKPSGPPGSGTPPTAEEMAAHMSAHECADECLFKNTKYLQSNGELDKDAIKASVTKIFTGDWAALASSAADKCLASAKSEVGASAKCKSGARQMVKCFTRAMFLNCPASSWTESTECAAAKARITKCPNAMVPMIPPKH

>LlinOBP32

MEVNAERNVTDEQRXAVRLCSRYTEVESGLAEAGYDCLAECFFIKLGLMGEDKTLNKENILEEVRIQFHEDXVEPARKALETCMEKKYNTKCPSGIDGTMQCFTVQLMLNCPXQNWTDGEECKETRTFMEKCGETLNYYD

>AlinOBP1

MNSLIPVLLVVCAAATRADEQTNAMVAKAFNKCREEFPISDDEIGGVREKTTIPESHNAKCLMACMLREGKMLRDGKYEKENALIMADVLNKDDPASADKAKQLVETCAGKVGTDAGGDECEFAYKMAVCAAEEAKKLGVRPPDF

>AlinOBP2

MSLKIQFFVFAAICAACVCAYQEQLKQTIRDCQDGKEVTDDELEEFTKPLIPRNREEKCIMACVMRTYNIISNGHYDPKIAFGILKGILKDHPEKLNKIKEVMDHCGEDVPSHMDDECDLAGEIMQCEVKYQKAMGMA

>AlinOBP3

MDIRFGFIIACLAILSVANAISKEYSARMIAAKEKCQKEFNVTDSVVEDFMKRNIKPESKSGKCMVHCIMEEMGMIDDHKINTEQVKLGNKEKWDDPALVELANQVADTCDQEVFTEGRCKCLVAVEYMMCLATHGDEVGLPHVDFEDSQDS

>AlinOBP4

MRIFVIFTAALTCVMAGELPEEMKEMAQGLHDSCVEETGVDNGLIAPCAKGNFADDAKLRCYFKCVFGNLGVISDEGELDAEAFGSILPDSMQELLPTIKSCGGTTGSDPCDLAMNFNKCLQKADPVNFLVI

>AlinOBP5

MVLKMNLLLVVLVMSQVFFSVTEAAMSQAQMKQAMKTVRNMCIPKSGVDKEALAKMVNGEFDESDQKLKCYLGCVLGMMQAVKNNKINLTMVRNQITKMLAPERGQRILAAFESCATVTGDDNCGLAFRFAKCIYDTDKEAFIVP

>AlinOBP6

MGFKFVKYRSYFFVLVIHIILCIQIKAKELTDEQKEQIFAEIKNCMESTKLTDEEFESIMAKKELPTSKEGKCFTKCLMEKMEYLEEGGKINVIAVQAGMEENMEKESEITKAKEIIQQCADSVPPEDSCEYAYGISQCMYNKMKEAGISGS

>AlinOBP7

MNRPLLLLTAVLTVGSGQQEDCKTAPAGWPRRPPQCCDLPFPLEGMKKEFGSCIRQIGNRQSSAVPTAQAVRDARLCIEECVYKGLGFMDEHKLNKDQLLEQLKKGIADKKDWTKPMEGAVKRCHETITKRETPQEAACQDSAHEFTHCAMRELFLNCPASEWNNNDECNLVKSRMQACPNIPPPPPPPPQGFRGQGPPPQ

>AlinOBP8

MDTHFGLLIASLAILHTANAVINKDYLEKVVTAKDKCLKEFNVDDSVVEDFIVKYNKPQSESGKCMVACFMEERGMMKDGKTITEQVMLDNQEKWIAATHVNMGKEVIDTCDKEVPNEENDKCDLAVDYMMCLVKRGDEAGLPKMDVAQLKH

>AlinOBP9

MMELWKWRLALIIFGLVSCIQQTEGSQRTKQQPKSKTKESVVGATRPRDAKATECVNKVNANEEESASFFRKEIPETEAGKCLLACYLEGKGLIVGGKISSSGAARVAARAYPNNRVKTGNVKHILSHCGTIAGRESNNCEMAYKLADCTTTLSDKFRL

>AlinOBP10

MFFNSVFLLVVCVSSYVTKGQELPPPGDVKNKTVVFKNSFLRSAKYCSSIYETSTLAIMALLMSEKSDDQNGKCFLNCMLQRYRLMSQDGSYNKDKFKPFLEYIPDSKFLQSIRGNLKNCISEKDPDPCEKASKFIKCFYTRARNKGEIGASKEVIPADGF

>AlinOBP11

MKTFVGLIFAVALVEFASAISKEYHDKAIEAKNTCAKLHNVDDETIMTYWKNHQLPEKEPETCIVICYLKEMKLVVDGKVDADAWKASNKEKWDDEKHVAAADEIVDKCSAEVPPTENECEWGLALTKCALKHGKEAGIPPPDMEHPKRR

>AlinOBP12

MTTKLRSIGLVFIVSISYAFAYQELLKETIKKCQNGRDVTDDEVEEFTKPLVPKNEEERCLVACVFKEYKVIIDGHFDPVNALNVAKVVYKDYPDKVERIKDVLDHCGEDIPTHNDNECDLAGDIMKCEVKYLNSVPKMTSLEFLAGSMAATAEP

>AlinOBP13

MNISTRMISLTMAYLAAALVSGHRALDGILPQANQDECREESNFRGELNDDVGRNVTQELKCFAACSLMKLGIMNEKDGTVNMTRLDELIASHTPGKDAADVFKTTVVEPCMKEVKKSTDYCEYSYQLIACGMSKVP

>AlinOBP14

MKPPGPPGSGTPPTAEERAARKIAHECADECLYKSSNLLTSAGELDKDAIKALVTKLYTGDWATAATTAIDKCLASAKGEVEATSKCKSGSFQLSRCFMRSMFLGCPASSWTESTECAAAKARLTKCPNAMAPMPHKK

>AlinOBP15

MFAITAALSLLLLNVAIAHPGHFDEDPECRPPHHHRLEEKDCCKIPNLFSKSKDEMHELVHKCFEEAGIKKHGHHDHHGPPPPPGLGLPPPPPPPPKNDSKFDCVEQCFLKNLELINEEGEVKVDELKALIAEKFTGDWASVGSSAIEKCLEKSKTEENDSTKCKAGSKRILICLARESFLSCPASEWTESDVCTAAKDRLEKCPHAPPPMNH

>AlinOBP16

MKFFASAALVLLVAAAVKANEKKANEKVTEIFNKCKETWPVTDEEIEQVKQKNSIPESKNVKCILACMLKEAKVLKDGEYNKDNAELMADVLYKDEPEHAEKSKQIIELCSAELGTKTDGDDCEYAYKMSVCAAKHAKELGVKTPEF

>AlinOBP17

MQAAFVLLGAALLVAVVSGAPPSVKEIVQNVSKKCAAETKASPEQAKIILTQNIPKNDVERCYLQCVYSGVGVIKDGKFSQEGGNKLVAMRFHDAKEKELAKQLINTCAKEIKAKDGEKCSLGKGIRQCFVAHGKEVNFFPHA

>AlinOBP19

MNTLLLCAVIAVSACFAYDFSDPEFNIIIDDELFDIAEGKDTLLRNRRDIDDDDERMAFEQDEADDPSNGPPDHLSEMNDENSHHKGHCKRHHKSCCGKTPLPLSLIQHGKNETKRSTGSECYEEIDAKMGNKTSLENDMDPYNCEKVKRMKKKQYCMHECKAKKMGVANEQGVLDFPKVKDLLLSRVNETWQKEVLGQAVDTCATSKFDQTWKDDQDEYKCNPQALQFKHCVWKQVELKCPEEYQNTGRHCKKLRTKLTSETNKETATKETTI

>AlinOBP20

MYTFNTFFVLTVASYVIAAPPADVPAECLVPKGKEEEVSKCCKLDAISTKEVAAAKECMKLVKDKDAESKGLLPKPEGFDCFDDCVMTKMGFMGADKKIDAAKVNAGAKTSYTGDWAEPGAKMVAKCLAQVSAMKEKEECASGADIYETCIFRESYMNCPEKSWTNSDACKANKERLTKCPKALPYLEKLDLS

>AlinOBP21

MNPTVAIISIFFVAYTQAHTKELHATEVYKLKVRDECNKDIKATPEQSEIVSKFKEVPKDETEKCLLECIYIKTGGIDADGKYSVEGFNKLIDMKYKGDENTNAKKINTDCAKKAVSKEGEKCSLGGSIRECFAAAAKENDFFTI

>AlinOBP22

MATNAKAVLFLALCGIVYVSAYQEVLRATISDCKGGKEVSQEELDEFIKPLIPQTREEKCLMACVFTAYNVIVEGHFDPKLAYGVAKNILHENPEKLKHIKETLDYCGHEIPTKMDDECELASEVMACRNKYNKDHGYDQDP

>AlinOBP23

MNTFAALVLVASVVALSQGNPTTGAPSTTLVSDQPTGSTASGVSKSPEEIKQKIKEQVGALTEACKSQSKITGEQAKIVATQAIPKTEAEKCFLECIYTGLQLTKDGKFNEPAARALAQKRFGNAPDDLQKANSMITACVKEVVVKDTNEKCALGRLIRECFVKNGAKINFFPKP

>AlinOBP24

MPTFSTLSTFLFALALTYGQIVEDPECRPPHPPGREDSCCILPELFIGGIQDVIRKCHDEAGLKKPSGPHGGGSPPSAEEMAAHWRAHECADECVLKSQNLLTTDGELNKDAIKAQVVKSFTGDWAKLASDIADKCLASAKGEVTSTATCKSGAGQFIYCFRRNLFLQCPSSSWTETTDCAAAKARITKCPNAKIPMGHHRH

>AlinOBP25

MDVMILPVFLIVFIAATASPAVLSAECPQRFPKEMRPLSSCCKVEMNPNSSYNSTAGEAIIDKCFGGFNSSATRPTGPPSGYDCEMECLMIEFGFMGKDKTINKDKIVTSIQDEYSADFQEAANKAIEICMGRKYQTSCPSGIDGMMECFAVQMMLNCPAKHWSGGEDCKETKQLIEKCGEVLSIFADYDTE

>AlinOBP26

MKSLWKCCKTVQSSKATHPTSEQMVEIKSCYSNWNHSVETNAAPEGFDCVEECVYSKLGFMGTDKTINKEKLLQFQKEETHEDFHEAITKSMDMCMGKTFTTKCPSGIDAVIKCEAIQIYLNCPAKHWDNGDDCQETKKLMEKCADVTSMYN

>AlinOBP27

MTQWKTASFFVAMAALVVIAFAGLPFQNEMAVMQCKTKFDVTSEDIQLLKDRKLPASHSGKCMMACILKKMKVMTKRGQFDLRNVQKWLRNKYQGDQANLAKGTYVAEACANILPTLGIQDECEMAAEIMSCVRNKSKLIKKTADGQLPSSTGI

>AlinOBP28

MSSDMANILLLLVIGAGIAVNEGDASMEALMECKKDYKVSREQIMSGDSSEEVKCFAECLMKKTGGMDEGGNFNTEKIKEEGRKHAKTDDQKRANDAAVDKCISETEAANPTGKCEKGFEFFKCVRGEMKSPM

>AlinOBP29

MYQLTCPVFVIVMYWVNQGSADVGLLDYSAQCVTEAGVTHDEADKIQQGNLPSNQQGKCYVACVLKSLGLVDRRGKISAENTNRLIDMYSNEPGDAKDKTKQAVNTCATEANRAWTWSQCEVAYRMMSCILRTRGSLQQTRSITITLPQSITINPPPLSFTLFSVG

>AlinOBP30

MQLKNTSDSLVGHLFSIALPNTLLNPSQLLPELAITSRQSPDIVSIACFLHAIGSNFLFASKTPSEANRQSWMHSNSLESVPLVSFESQVVIVLLVKGNT

>AsutOBP1

MNSLIPVLLVVCAAATRADEQTNAMVAKAFNKCREEFPISDDEIGGVREKTTIPESHNAKCLMACMLREGKMLRDGKYEKENALIMADVLNKDDPASADKAKQLVETCAGKVGTDAGGDECEFAYKMAVCAAEEAKKLGVRPPDF

>AsutOBP2

MSLKIQFFVFAAICAACVCAYQEQLKQTIRDCQDGKEVTDDELEEFTKPLIPKNREEKCIMACVMRTYNIISNGHYDPKIAFGILKGILKDHPEKLNKIKEVMDHCGEDVPSHMDDECDLAGEIMQCEVKYQKAMGMA

>AsutOBP3

MATNAKAVLFLALCGIVYVSAYQEVLKATISDCKGGKEVSQEELDEFIKPLIPQTREEKCLMACVFTAYNVIVEGHFDPKLAYGVAKNILHENPEKLKHIKETLDYCGHEIPTKMDDECELASEVMACRNKYNKDHGYDQDP

>AsutOBP4

MRIFVIFTAALTCVMAGELPEEMKEMAQGLHDSCVEETGVDNGLIAPCAKGNFADDAKLRCYFKCVFGNLGVISDEGELDAEAFGSILPDSMQELLPTIKSCGGTTGSDPCDLAMNFNKCLQKADPVNFMVI

>AsutOBP5

MGHIPMSSDMTNILLLLVIGAGIAVNEGDASMEALMECKKDFKVSREQIMSGDSSEEVKCFAECLMKKTGGMDEGGNFNTEKIKEEGRKHAKTDDQKRAHDAAVDKCISETEAANPTGKCEKGFEFFKCVRGEMKSLM

>AsutOBP6

MGFKFVKYRSYFFVLVIHIILCIQIKAKELTDEQKEQIFAEIKNCMESTKLTDEEFESIMAKKELPTSKEGKCFTKCLMEKMEYLEEGGKINVIAVQAGLEENMEKESEITKAKEIIQQCADTVPPEDSCEYAYGISQCMYTKMKEAGISGGP

>AsutOBP7

MNRPLLLLTAVLTVGSGQQEDCKTAPAGWPRRPPQCCDLPFPLEGMKKEFGSCIRQIGNRQSSAVPTAQAVRDARLCIEECVYKGLGFMDEHKLNKDQLLEQLKKGIADKKDWTKPMEGAVKKCHETITKRETPQEAACQDSAHEFTHCVMRELFLNCPASEWNNNDECNLVKSRMQACPNIPPPPPPPPQGFRGQGPPPQ

>AsutOBP8

MKLALVTAFLSAIVLAEGNINKEYLDKLIAAKEKCVKEFSVDDSIVEDLYVRYNKPPTESGKCMVACYMEERGMMKDGKTITEQVMLDNQEKWIAATHVNMGKEVIDTCDKEVPNEENDKCDLAVDYMMCLVKRGDEAGLPKMDVAQLKH

>AsutOBP9

MMELWKWRLALIIFGLVSCIQQTEGSQRTKQQPKSKTKENVVGATRPRDAKATECVNQVKANEEESASFFRKEIPETEAGKCLLACYLEGKGLIVGGKISSSGAARLAARAYPNNRVKTGNVKHILSHCGTIAGRESNNCEMAYKLADCTTTLSDKFKL

>AsutOBP10

MFFNSVFLLVVCVSSYVTKGQELPPPGDVKNKTVVFKNSFLRSAKYCSSIYETSTLAIMALLMSEKSDDQNGKCFLNCMLQRYRLMSQDGSYNKDKFKPFLEYIPDSKFLQSIRGNLKNCISEKDPDPCEKASKFVKCFYTRARNKGEIGASKEVIPADGF

>AsutOBP11

MKTFVGLIFAVALVEFASAVSKEYHDKAIAAKNTCAKLHNVDDETIMKFWKAHQLPEKEPETCIIICYMKEMKLVVDGKVDADAWKASNKEKWDDEKHVAAADEIVDKCSAEVPPTENECEWGLALTKCALKHGKEAGIPPPDMEHPKRR

>AsutOBP12

MFQAFVYQELLKETIKKCQNGRDVTDDEVEEFTKPLVPKNEEERCLVACVFKEYKVIIDGHFDPVNALNVAKVVYKDYPDKVERIKDVLDHCGEDIPSHNDNECDLAGDIMKCEVKYLNSIPKMTSLEFLAGSMAATAEP

>AsutOBP13

MGDVSNTYGSYDSYGASYGGYIRGSTPSGSGRGYKQSYEGDIGSGTSYARGAGYGGAASYGTGYYNGNYGPDRTIYNPDSRTRGSYGGFMRGGGSSSNDGIFGATDFGSYAKQIDTTSYDSSESYDRVESYGGIPSGTPRGIPYNGYHNNANIRWPDSNSQGNARKNGSSLEDVEPCTILCIFRQMKMTNGDSYLEQQSVAAVLMRRARDPQLKDFIGRTVQMCFERFGLANKGRCESAKLFALCMEEAGKMNCEDWDVNKRFALKNPKPGPVLTMPQPLPPPPPRG

>AsutOBP23

MFAITAALSLLLLNVAIAHPGHFDEDPECRPPHHHRLEEKDCCKIPNLFSNSRDEMHELVHKCFEEAGIKKHGHHDHHGPPPPPGLGLPPPPPPPPKNDSKFDCVEQCFLKNLELINEEGEVKVDELKALIAEKFTGDWASVGSSTIEKCLEKSKTEENDSSKCKAGSKRILICLARESFLSCPASEWTESDVCTAAKDRLEKCPHAPPPMNN

>AsutOBP31

MGRLTKTQNADLLIHPAGKIRVGVLPELFVGGIQDVIPCHDEARLKKPSGRHGGGGSPPSAEDMAAHLRAHECADECVLKSQNLLTTDGELNKDAIKAQVVKSFTGDWAKLASDTADKCLASAKGEVTATATCKSGAGQFIYCFRRNLFLQCPSSSWTETTDCAAAKARITKCPNAKIPMGHHRH

>AsutOBP32

MDVMILPVFLIVFIAATASPAVLSAECPQRFPKEMRPLSSCCTVEMNPNSSYNYTEGDPIINKCFGDFNSSATRPTGPPSGYDCELECLMIEFGYMGKDKTINKDKIVKSIEDEYSADFQEAGHKAIEICMGRKYHTSCPSGIDGMIECFAVQMMLNCPAKHWSGGEDCKETKQLIDKCGEVLSIFADYDTD

>NlugOBP1

MKSFIVCIAVSYLLVANIKADEATSSSDAESLITSTTLSPASNESDAARSAIKEQLAKLTESCKTSSQANSDDAKIIGTESVPKTEGEKCFLQCVYTGFGIVKNDQFSVEGARLLAQKRFGAFPEELEKANQLIETCSKEAVKKDSKDKCPMGFLIRQCFVKNGQKINFFPKA

>NlugOBP2

MKCQIVLAALALATICEVSYAGLTPDKLKELKPLIDTCIKQSKVEEDTLGKLHNGHEIPSSQSGKCFIACMAEHMKLMKDGKFEPEMTMEFIDKMVQDKDKAAEIKKSLGECIKSVPEGDKCEMAAGLATCMKDHHAELAGMN

>NlugOBP3

MKASAAITLVFLSLAVFHCSEAKLDKAKKEAAIKKCQAETQATDEDVMKVRKEHIVPDSEEGKCFIACGFNSYDMLKDNRINLEGVNAFFEKLYDEQDKRDIAIKAAASCAATETVSGLNECHYAAKYFACMQRHPDFAKMKDDFDI

>NlugOBP4

MERTSVLIVFTFIPFLSSVLGANFLMMQQSMQGTQMPMIQSIASELKFCMDVNAEQNSDGLNDYLPLLFNEELPSTLGQKCFLTCLFNRFGLLKDGFLDTKTAKNLVETFYADKHDEKTMANIAINVCHVAAVPDALNPCEIGFSLKSCFVDSNKKGKELRGKN

>NlugOBP5

MGIYTTNLIFTLLGSAVVSGVFIDGRNEYRLTRQAPPDDECRPPRPGPNEDGVCCDMPPVFRTAHDKFESCLEELSSIFPPPPPPPHGHHGPPPPPPGARGPPPPPPPGGRRGPPPGFGGPPGHEPPIFACAHECLFNKTGMLENGKLNVEALKKKLEDELGENEVWKNLVQSIVDKCMESKDAPSNEMCTSGSHELARCVLRDMFMNCPQEKWKESDDCSNMKMKLEKCPELVPPMAMRLPHPPMP

>NlugOBP6

MSTFHKFVISGMVVLAGALFVTAEDTTIKIKTPSPHKHQQVYCQAPPTAPERLERIIEQCQDDIKTALQEALNVLTDTSPRDLVKKTRSKREVFSGEEKRIAGCLLQCVYRKVKAVDDQGMPTVPGLVRLYSEGVQDRNYYVATVQAVQQCVSASQHFRYYNPQVLKEDGYTCDLAYDMFNCVSDKIEAFCGRTP

>NlugOBP7

MLLEVCRFSVFLIALFATVNGRFTEEEKQLMNQVHSQCISETGTSEDLVTKATTGDFADDDNLKCYVKCIWSTLTVMDDEGNFDVGVLEVMLPADMKDTVMKAMNACTGVGGATPCEKAFAMTKCLYKEAPSDFFLP

>NlugOBP8

MVTSALMQTATAACLLLVTAYAYDFSDPYFNEHLQSAMEEIMEEEMLSIGRVQRDADQGQEVADEYFKCKHRNLKTCCGKINLMKNYGDKGKIYGKQCYEEVVSAFKTNSSSTADDDDSMMDMFSCEKVKMIKLKHICVHECIGKKTKILKEDGTLNPEEIKQYAREYMFNEEWSKELGEKALDKCLSQTYNSVTKMLDEYEIKCNPSSVQFHHCLWKEIELTCPESKVDLKAKCVRLRERLRKQQAAGM

>NlugOBP9

MPPVFRTAHDKFESCLEELSSIFPPPPPPPHGHHGPPPPPPGARGPPPPPPPGGRRGPPPGFGGPPGHEPPIFACAHECLFNKTGMLENGKLNVEALKKKLEDELGENEVWKNLVQSIVDKCMESKDAPSNEMCTSGSHELARCVLRDMFMNCPQEKWKESDDCSNMKMKLEKCPELVPPMAMRLPHPPMP

>NlugOBP10

MLSESMPVIARVERDASSTTPGFPFKTNQRVALKRAKRSTIFPIDGIVERVAIFPVHEKTKRVVRESSEEDDSCKKMKPEHGAKMCCELPSVFRGSPEIFKACREELGLPDHKSPPPAPSAEGNGKPPHHGGPPHHRGKGCIAECLFNKTGLLEGGKLNKEALQKSLDEHLKTDDAWKAVATSTLDKCYDDVQTKDFKPDNEKFTSGSSEFMRCFSRGLFMDCIPSKWTDSDECSKTKETLEKCPMMLPPG

>LstrOBP1

MNVKMSALLKVGLVFLVYLCSYSGAIPALTEAQIEQVGKAMANMCISSSGVQRSLITKAMKGEIEDDRKLKCFFGCIMEAVQVTKNGRMQPEVLKRRANAMLPKTMREMILPTIDSCSHIENEDKCELAYSIVKCHFSVNGKNPFFFNF

>LstrOBP2

MLLEVCRFSVFLLALSATVNGRFSEEEKQLMNQVHAQCVTETGTAEDIVNKATKGDFTEDDNLKCYVKCIWSTLTVMDDEGDFDVGVLEVMLPADMKDIVMKAMSACTGAGAGLSPCEKAFAVTKCLYKEAPADFFLP

>LstrOBP3

MERTRLFIILAFMPFLPSALGANFAMMQQSLQGTNIPMIQSIAGELKFCMDVNSEQNSDGLDDYLPLLFNEELPTTLGQKCFLTCLFNRFGLLKDGFLDTQTAKTLVETFYKDKHDEKTMANIAINVCHVSAVPDVLNPCEIGFSLKSCFVDSNKKGKELRHKN

>LstrOBP4

MKSLIVCVVVSCLLVANTKADEATSSKPSSSPNAADALIASTTLSPASNETDAARAAIKEQLAKLTESCKTSSQANSDEAKIIGTESVPKTEGEKCFLQCVYTGLGIVKNEQFSVEGAKLLAQKRFGSFPEELEKANQLIETCSKEAVKKDSKDKCPMGFLIRQCFVKNGQKINFFPKA

>LstrOBP5

MKCQVLLALFVVAAVCEVCYAGLTPEKLKELKPLIDTCIKQSKAEEETIGKLQNGHEIPSSQTGKCFIACMAEHMKLMKDGKFEPAMTMEFIDKMVQDKDKAAEIKKAVDDCFKSVPDTGDKCEMAAGLATCMKEHHAELAGMN

>LstrOBP6

MLYVLYFVIVTSALSAVITQIMAADSSDMLTVFNKCRDKTSATEDDIKTFRAQQIPSTTTGKCMLACMFNHSGLMKDGKYDSEGALKLVGQVFADNPIKLGKARQLINGCTDEVKKENDKCEIASKIADCTVKMSSQVGLS

>LstrOBP7

MKASAALTLVFLALAVFHCSEAKIDKAKKEAAIKKCQAETSASDEDVKKVRKEHVVPESEEGKCFIACGFNTYDMLKDNRINLEGVNAFFEKLYDEQEKRDIAIKAAASCAATESISGLNECHVAAKYFACLQRHPDFVKMKEDFDV

>LstrOBP8

MITSALTPSAVLAACLLLATAYAYDFSDPYFNEHLQSAMEEIMEEEMLSIGRVQRDADQGQEVADEYFKCKHRNLKTCCGKINLMKNYGDKGKIYGKQCYEEVVSAFKSNSSSTADDDDSMMDMFSCEKVKMIKLKHICVHECIGKKTKILKEDGSLNAEEIKQYAREYMFNEEWSKELGEKALDKCLTQTYNSVTKMLDEYEIKCNPTSVQFHHCLWKEIEMTCPESKVDLKAKCVRLRERLRKQQAAGM

>LstrOBP9

MSRLHKFAISGMAVLAGAMLIAAEDTTIKIKNPSPHKQQQVYCQAPPTAPERLERIIEQCQDDIKTALLQEALNVLTDTSPRDLVKKTRSKREVFSGEEKRIAGCLLQCVYRKVKAVDDQGMPTVPGLVRLYSEGVQDRNYYVATVQAVQQCVSASQHFRYYNPQVLKEDGYTCDLAYDMFNCVSDKIEAFCGRTP

>MvicOBP3

MASMRFTTEQIDYYGKACNASEDDLVVVKSYKVPSSETGKCLMKCMITKLGLLNDDGSYNKTGMEAGLKKYWSEWSTEKIESINNKCYEEALLVSKEVIATCNYSYTVMACLNKQLDLDKST

>NribOBP3

MRFTTEQIDYYGKACNASEDDLVVVKSYKVPSTETGKCLMKCMITKLGLLNDDGSYNKTGMEAGLKKYWSEWSTEKIETINNKCYEEALLVSKEVVATCNYSYTVMACLNKQLDLDKST

>RpadOBP3

MISPTFYISLLFSIGMLISCSFGRFTTEQIDHYGKACNASEDDLVIVKSYKVPTSDTGKCLMKCMISKLGLLNDDGSYNKTGMEAGLKKYWSEWSTDTIENINNKCYEEALLVSKDVVATCNYAYVVMACLNKQLKLDKST

>RpadOBP7

MNMLPATVLLAVIAATVLKDSDAYLSEAAIKKTQQMLKNVCSKKHSVGEDVFTDIKKGIFPENNNNIKCYFACNFKTMQMINPKGILDKKMFKDKMTMLAPPNVLEILLPAIEQCIGTDKDTEICQSSYNFIKCAYRVDPKSLEFLPL

>AglyOBP2

MKVSAATAVLVALVATVQSSDPCNISTCYKSGTTKPPTTVTPTRLPVQSSSTPTSHQQTTYAKDHVHSSTATKSGVNTTATTTSGASVNGTERTTVVKSSSGVAGNVTTPKPTMTDGHLALKQKLNTIAVKCKDELHAPQEIMALVSNTVVPQNEQQRCYLECVYKNLNLIKNNKFSVDDGKAMAKIRFANQPEEHKKAVTIIETCEKEAIIDPKTTEKCAAGRVIRNCFVKNGEKINFFPKA

>AglyOBP3

MISSTFYTSLMFGIVMLISCSFGRFTTEQIDHYGKACNATEDDLVVVKSYKVPTSDTGKCLMKCMISKLGLLNDDGSYNKTGMEAGLKKYWSEWSTDTIESINNKCYEEALLVSKDIIATCNYAYVVMACLNKQLDLDKST

>AglyOBP4

MRGNYSLVVFLLFGFGLLEIYCQKQETSGKCRAPDKAPLNLEIIINICQEEIKSALLQEALDILNDGTLEQNTPSYSRSKRDADEDLSNEERRVAGCLLQCVYKKVKAVDETGFPVVDGLMKLYNEGVQDRNYYMATLSAVRHCISIAQQLKQQQPSKSFDDGQTCDLAYEMFECVSEKIEENCGVENKSNNLSQRQV

>AglyOBP5

MKMSTNGATMKCVAIAVVLFQMSVIFAEAGHQRRGKELLDTEDSDFFRCKQASRKSCCGPENAMKRFGDKDKVAADECYAQVAEKFATVTATTPKQDLFSGEAVKITKKKQFCLHECIGKKNKLLTEDGSLNKTFIADYAMKSVFKEQWQKQIGQKALDKCLEETYIPWPAEETENKCNPVYVQFQHCLWLEYESNCPDNKIKLTKKCEKTRNRYRMQKSPSNQ

>AglyOBP6

MQKVVFLCIFAIICQTVFTVGFERTWILRQKRVTNDDECRTLIPSSEKKLPTCCQMPNILPGLDNAWEVCFEKFKQFKDKHATKEYKEMAHGNEPPCLFQCVFMQSGLTTSDGKVNEDAVIKKMAEGMDNDEKWKSIWRNTFNKCLNDVKQEDKEQIKMTNTPTGRLMKCFLRDLYMNRPKNVWVESSECSNLKDLVEKCPKMPPPVFKSPPKLI

>AglyOBP7

MVARKRMYMLPATVLLAVVAATILKDSDAYLSEEAIKKTQKMLKNVCSKKHSVEEEVFTDIKKGIFPENNNNIKCYFACNFRTMQMVNQKGILDKKMFKDKMTMLAPPNVLAILLPPIEQCIGNDKDTEICRSSYNFIKCAHRVDPKSLEFLPL

>AglyOBP8

MFAFKVACLCLSVAVVFGENNQQNSNDRSASIFQSCISETKLSGDALKGFRSMSIPKTQAEKCMMGCLMRKVNVINNGKFSVEEATKVAQKYYGTNETMMKKAKDLIDVCAKKAQSTTEECALAGIVTTCIVEEAQKAGLTGGPGSRSKRTVSPKFRHSIV

>AglyOBP9

MIIKKTLLVSGFVLFGCMFSINKAADDADAKDKELMSKLITVAFKCFKDADWGTCGEMITTKYDITQAKYKQCTCHMACAGEDLGLINSNGQPEPAKFLEYVKRINNSVIKSQLQHIYDKCQNVKGTEKCDLAEQFAICAFKESPEMKERVTKLIEMLVKMKPKSK

>AglyOBP10

MEHLRGTNVVFAIVMALLVVQSSTRPQPDELDDIKKTLYNACSEKFPLTEEIKNNVKNSIVIDDQNFKCFLRCCFDEMSLIDEDGIIDGESLAAMAVDKIKPVAEKIVHDCLPAGKQEKQDGCEASFKFFSCGIKLNPLTIELLPLQ

>SfurOBP1

MLLEVCRFSVFLLAFSATVYGRFSEEEKQLMNQVHTQCVTETGTSEDLVNKATNGDFAEDENLKCYVKCIWSTLTVMDDDGNFDVGVLEVMLPADMKDIVMKAMSACIGAGGGSPCEKAFAVTKCLYKEAPADFFLP

>SfurOBP2

MSTLLNFVFVFLVCLCSYSEASPALTEAQIEQVGKAMANMCISSSGVQRSLITKAMTGEIEDDRKLKCFFGCIMEAVQVTKNGKMQPEVLKRRANAMLPKTMREMILPTIDSCSHIENEDKCELAYSIVKCHFSVNGKNPFFFNF

>SfurOBP3

MPPVFRTAHDKFESCLEELSSIFPPPPPPNGHHGPPPPGGPGGHGPPPPPPPGGRRGPPPGFGGPPGHEPPIFACAHECLFNKTGLLENGKLNVEALKKKLEGELGDDEVWKNLLQSIVDKCMESKDPPSNDMCTSGSHELARCVLRDMFMNCPQEKWKESDDCSNMKMKLEKCPELVPPMAMRLSQPPMP

>SfurOBP4

MCCDLPLVYRGTPELFKACREELGFPDHKPPPPPPSSDGHGPHGHPQRGMCVAECLFNRTGLLENGKINKEALKKALDEYLKTDGAWKDVATTTLEICYDAQTRGDFKPDNEKFTSGSSEFLKCFTRNLFMDCIPEKWTDSEECKKMKEKIDKCPKMLPPALFNKRPH

>SfurOBP5

MLYVSYFVIVTAASSAVITQIMAADSNNPDMQTVFNNCREEASATEDDIKTFRAQQIPSTTTGKCMLACMFNHSGLMKEGKYDSEGALKLVGQVFAADPVKLGKAKTLINTCSDEVKNENDKCEIASKIADCTVKMTSQVGLS

>SfurOBP6

MKCQVFLASFVLVAVFELGYAGLTPEKLKEIKPLIDTCIKESKVEEETLGKLHNGHEIPSSQSGKCFIACMAEHMKLMKDGKFEPAMTMEFIDKMVQDKVKADEIKKAVDDCFKSVPDGDKCEMAASLATCMKEHHAELAGMN

>SfurOBP7

MEEEMLSIGRVQRDADQTQEVADEYFKCKHRNLKTCCGKINLMKNYGDKGKIYGKQCYEEVVSAFKTNSSSTADDDDSMMDMFSCEKVKMIKLKHICVHECIGKKTKILKEDGSLNAEEIKQYAREYMFNEEWSKELGERALDKCLTQSYNSVTKMLDEYEIKCNPTSVQFHHCLWKEIEMTCPESKVDLKAKCVRLRERLRKQQAAGM

>SfurOBP8

MERTHVLIIAFAFIPFLSSAMQADFAMMQFPMQGTGTPMIQSIAGELKYCMDVNAEQNSDGLEDYLPLLFNEELPTSLGQKCFLTCLFNRFGLLKDGFLDAQTAKTLVETFYKDKHDEKTMANIAINVCRVSAVPDILNPCEIGFSLKSCFVDSNKKGKELRGKN

>SfurOBP9

MNTFQKFILSGMVVLAGAMLITAEDTTIKIKNQQSPHKQQQVYCQAPPTAPERLERIIEQCQDDIKTALLQEALNVLTDTSPRDLVKKTRSKREVFSGEEKRIAGCLLQCVYRKVKAVNDQGMPTVPGLVRLYSEGVQDRNYYVATVQAVQQCVSASQHFRYYNPQVLKEDGYTCDLAYDMFNCVSDKIEAFCGRTP

>SfurOBP10

MASGLVAAELEKLKNSCLKKSGATEDTARRLVGINVVTENHVESCFLTCIYKGLKIVSSDNKFQPDTVKKIADDHFLGRNLKVTYQIADSCTKEIKADPADKCSIGASFRNCFSKYGKELGFFPHM

>SfurOBP11

MLLEVCRFSVFLLALSATVYGRFSEEEKQLMNQVHTQCVTETGTSEDLVNKATNGDFAEDENLKCYVKCIWSTLTVMDDDGNFDVGVLEVMLPADMKDIVMKAMSACIGAGGGSPCEKAFAVTKCLYKEAPADFFLP

>SfurOBP12

MRGYILVLCVLLFMRGMASGLVAAELEKLKNSCLKKSGATEDTARRLVGINVVTENHVESCFLTCIYKGLKIVSSDNKFQPDTVKKIADDHFLGRNLKVTYQIADSCTKEIKADPADKCSIGASFRNCFSKYGKELGFFPHM

>LeryOBP7

MVARKRMYNMLPTNVLLTIIAATVLNDCDAYLSEAAIKKTQQMLKSVCSKKYTVEEDVFTNIKKGIFPEDNNNIKCYFSCVFKTMQMINQKGSLDKKIFKEKMSMMAPPSVYNILLPAIEQCIGKDNGEELCQASYNFIKCAHHIDPKSLEFLPL

>DplaOBP3

MISSTFYITSVFGIAMLISCGYGRFTTDQIDYYGKACNASEDDLVVVKSYKVPSTETGKCLMKCMITKLGLLNDDGSYNKTGMEAGLKKHWSEWSTEKIENINNKCYEEALLVSKEVVATCNYSYTVMACLNKQLDLDKST

>BbraOBP3

MISSTFYITLLFGIAMIISCSYGRFTTDQIDYYGKACNASEDDLVVVKSYKVPSTETGKCLMKCMITKLGLLNDDGSYNKTGMEIGLKKYWSEWSTEKIEAINNKCYEEALLVSKEVIATCNYSYTVMACLNKQLDLDKST

>AlinCSP1

MLKVLVLLAAVVCCVSAAATYTSKYDNIDLDEILSNTRLYKKYFDCLANKGKCTPDGKELKESLPDALKTNCAKCTKKQQEGTDKVFRHVLKNKPNDYKVLESIYDPPGIYRKKYEAEAEKRGIKLPGSH

>AlinCSP2

MKVAVLVLLCVGAALSAEVYTSKYDNIDVDKILSNDRILTRYIKCLMEEGNCTNEGKELKKTLPDALASGCTKCSEKQKAQTEKVLRHLSKNRPRDWALLKTKYDPKGEYSKKYEKEAKALTA

>AlinCSP3

MISKLSMVLLIGAFADVWAAEQYTDKYDNIDIDEILNNDRMYKNYFHCVMGNGKCTPDGLELKAKIPEALQTECAKCTDKQKKEVEKVLRFIINQKKDDYKLLEEKFDPEGVYRKKYEAQKKLVEEGKPIEY

>AlinCSP4

MRIILSAFLVAMACSLATCEMTEEEFYTKVFEEVDPDFILDNERILTSYLKCFYNEIECNAHAEVVKKSIPDVLATVCGRCSDKQKSIFKYSLNKFIPAHPKDWEKILSIYDPSGEAWPKVKAFIES

>AlinCSP5

MGHLTIVLLAAAFEVLTGSRAYTTHYDYIDVDQVLNNTRLYTKYVECLLGQGKCTPEARELRDKLPEALQTNCARCSERQASESHRVIRFLIQNRQEDFKLLEAKYDPSGLYFKRFEEETKRNVSLS

>AlinCSP6

MFYKLSVVVLMGILAGVWAADKYTDKYDNIDIDEILTNERLYKKYFDCIQGTGKCTPDGIELKEKIPEALKTECAKCNEKQKAGVEKVMRYLITKKPEDFKILEDKFDPEGVYRKKYEAQRKLVEEGKPVEY

>AlinCSP7

MNYKLSVILLIGVLASVWAASTYTDKYDNIDLDEILTNERLYKKYFDCIQGKGKCTPDGTELKEAIPDALKTECAKCNAKQKAGVEKVLRHLLTKKAEDYKILEDKFDPEGVYRKKYEAQKKLADEGKPIVL

>AlinCSP8

MDYKLSVMLLMGVLACAWAADKYTDKYDNIDIDEILNNERLYKKYFDCILGNGKCTPDGTELKETIPDALKTACAKCNDKQKAGVEKVLRHLLTKKAEDYKILEAKFDPEGVYRKKYEAQKKLAEEGKPIAL

>AlinCSP11

MKVFFSGLLLVCMASVSLCADEYTDKYDSVDLDEILNNQRLYQKYIDCVMGKGKCTPDGALLKEKIPEALQNECAKCSAKQKKGAEKVLRFLINEKADDYKALEEKYDPEGTFRSKYEEQKKNLKEGKPLSV

>AlinCSP12

MMIIIVFGISALLVVVEGAPLQYSDTRYDDVELTTILSNDELYIKLFQCLIGRGKCTPDWEILKDALPGALLDNCSECSNKQKFGTKTLLAHLVHERPSDMRLLEGEFDPDGSYRKELEKEEKESNDINRKRSANLEEVEILDKIKRIIK

>AlinCSP13

MKFVAALLVASVAVLAVEAANQYTTKYDNIDLDDILKNQRLYKKYFECLTGNGKCTPDGKELKEHLPDALKTGCSKCSEKQRAGSEKVIKHLLKNKPQDYAVLEKIYDPSGIYKKKYEAEAKKLGINV

>AlinCSP14

MNSAIVLCVVALAGMVLARPDDTYTTKYDNVDLDEILGNDRLLVPYIKCTLDEGKCAPDAKELKEHIREALENGCAKCTDKQKEGTRRVIAHLIKHKNADWQKLKAKYDPEGKYTHKYEKELEEVQH

>AlinCSP15

MKLIVAVALLCVVAESWAASTYTDKWDNINVDEILESQRLLKAYVDCLLDRGRCTPDGKALKETLPDALENECSKCTDKQKSGSDKVIRHLVNKRPEMWKELSAKYDPNNIYQDRYKDKIEAVKGQ

>AlinCSP16

MLPFYVFSLCAVFVACQETYTSKYDNVNVEDALKNDRLYKAYFNCLADRGPCTREGNMLKEALPDGLRNNCSLCTDPQRRGTHQVIRFLFKYRPEDMKLLEEIYDPEGIYKTKYAEERKKLME

>AlucCSP1

MLKVLVLLAAVVCCVSAAATYTSKYDNIDLDEILSNTRLYKKYFDCLANKGKCTPDGKELKESLPDALKTNCAKCTKKQQEGTDKVLRHVLKNKPNDYKVLESIYDPTGIYRKKYEIEAEKRGIKLPGSH

>AlucCSP2

MVGKLSVVLLIGAVGMVLAADKYTDKYDNIDVDEILGNQRLYQKYFDCIQGKGKCTPDGAELKKNIPEALQTDCAKCSEKQKAGVEKVLRHLINEKPEDYKVLEEQFDPEGVYRKKYEHLKKKVEEGKPVEY

>AlucCSP3

MLKVLVLLAAVVCCVSAAATYTTKYDNIDLDEILSNQRLYKKYYDCLANKGKCTPDGKELKEALPDALKTNCSKCSKKQQEGTDKVLRYVLKNKPNDYKVLENIYDPSGNYRKRYEDEASKRGIKLPGSH

>AlucCSP4

MVSKLSIVLLIGALADVWASELYTDKYDNIDVDEILGNQRLYQKYFDCIQGKGKCTPDGAELKKNIPEALQTDCAKCSEKQKAGVEKVLRHLINEKPEDYKVLEEQFDPEGVYRKKYEHLKKKVEEGKPIEY

>AlucCSP5

MVGKLSVVLLIGAVGMVLAAELYTDKYDNIDVDEILGNQRLYQKYFDCIQGKGKCTPDGAELKKNIPEALQTDCAKCSEKQKAGVEKVLRHLINEKPEDYKVLEEQFDPEGVYRKKYEHLKKKVEEGKPV

>AlucCSP6

MVSKLSIVLLIGALADVWASELYTDKYDNIDVDEILGNQRLYQKYFDCIQGKGKCTPDGAELKKNIPEALQTDCAKCSEKQKAGVEKVLRHLINEKPEDYKVLEEQFDPEGVYRKKYEHLKKKVEEGKPIEY

>AlucCSP7

MVSKLSIVLLLGALADVWAAELYTDKYDNIDIDEILNNDRMYKNYFNCVMGNGKCTPDGLELKAKIPEALQTECAKCSDKQKKGAEKVLRFIINQKKDDYKLLEEKFDPEGVYRKKYEAQKKLAEEGKPIEY

>AlucCSP8

MLKVLVLLANAASTYTTKYDNIDLDEILSNQRLYKKYYDCLANKGKCTPDGKELKEALPDALKTNCSKCSKKQQEGTDKVLRYVLKNKPNDYKVLENIYDPSGNYRKRYEDEASKRGIKLPGSH

>AsutCSP1

MLPFYVFSLCAVFVACQETYTSKYDNVNVEDALKNDRLYKAYFNCLADRGPCTREGNMLKEALPDGLRNNCSLCTDPQRRGTHQVIRFLFKYRPEDMKLLEEIYDPEGIYKTKYAEERKKLME

>AsutCSP2

MGHFPPVFSLSPVLLVASLHTMNTSTLLKIAFLLGCVAACLAAETRSSVSDEALEAALKDKRYLTRQLKCALGEGACDPVGRRLKTYAPLVLRGACPKCTPSEVRQIQQVLSHIQRHYPKEWAKILKQYAGQ

>AsutCSP3

MKFVAALLVASVAVLAVEAANQYTTKYDNIDLDDILKNQRLYKKYFECLTGKGKCTPDGKELKEHLPDALKTGCSKCSEKQRAGSEKVIKHLLKNKPQDYAVLEKIYDPSGIYKKKYEAEAKKLGINV

>AsutCSP4

MRIILSAFLVAMACSLATCEMTEEEFYTKVFEEVDPDFILDNERILTSYLKCFYSEIECNAHAEVVKKSIPDVLATVCGRCSDKQKSIFKYSLNKFIPAHPKDWEKILSIYDPSGEAWPKVKAFIES

>AsutCSP5

MDYKFFVVMQIGVISSVCAAGTYTDKYDNVNLDEVLNNERLYRNYFNCLQGKGKCTLDGAILKEVIPSALKTDCALCSVRQKKGAEKVLIFLITKKPDDFKILEDKFDPEGVYRKKYEAQRKLVEEGKPIH

>AsutCSP6

MVCKLFAVVLMGILAGVWAADKYTDKYDNIDIDEILTNERLYKKYFDCIQGIGKCTPDGIELKEKIPEALKTECAKCNEKQKAGVEKVMRYLITKKPEDFKILEDKFDPEGVYRKKYEAQRKLVEEGKPVEY

>AsutCSP7

MVSKLSMVLLIGALADVWASELYTDKYDSIDIDEILNNDRMYKNYFNCVMGNGKCTPDGTELKAKIPEALQTECAKCSDKQKKGVEKVLRFLIKEKKDDYKLLEEKFDPEGVYRKKYEAQKKLVEEGKPIEY

>AsutCSP8

MDYKLSVMLVMGVLACAWAADMYTDQYDNIDIEEILTNERLYKKYFDCIIGNGKCTPDGTELKETIPDALKTACAKCNDKQKAGVEKVLRHLLTKKAEDYKILEAKFDPEGVYRKKYEAQKKLAEEGKPIVL

>NlugCSP1

MFKNVLLVCLLVAVVSAKPKPAEKKQYTTKYDNIDLDEILNNQRLFDNYYKCLLGGKCTPDGQELREALPDALATACSKCTEKQRVGTEKVIKYLIEKKPTEYSELEKKYDPQGNYKRKYQAEAAKRGIKV

>NlugCSP2

MSKLPVTLVLMLAVFSVDCGKLYKDRYTTKFDKIDLDEALNNQRLFESYLKCLMGDKCSPDGYELREALPDALATACAKCSEAQKAGTEKVIRFLIEKRPKEYALLEKKYDPEGIYRDKYKPIAEMKGIKLD

>NlugCSP3

MKFLCVTIFECALIVVAFGMPQDTTYPTTYDDVNVDDILHNDRLFNRYFTCLTKKEGCTPEGKLLAATIPDALATTCAKCSAKQKTAAEKVIKYLYFNKRDKFDELAKIYDPESNYLNKYLVDGFPAKV

>NlugCSP4

MFLIAVWALSPRRLPWGLPWGGLAGVAAQQQAKNTRYTTRFDSIDVEVILKNERIFRRYMDCLLDKGRCTPEARELKRLLPEALKTECLKCSEVQRRQGAKVMAFIIKNKRPSWELLLAKYDPQGIFRAKYMYNENNIEAVLKQLEREQQGIYGTYSSTNSTTSSNSTSIR

>NlugCSP5

MRCLLLVAVVCAALVAVCHAQDSKYTSKYDNIDIDKILKNDRVLSQYIKCLMGEGSCTQEGRELKRLLPDAIQSNCSKCSEKQRSASVKVMRHLRQSRERDWNRLLDKYDPQGDKRKNLKLD

>NlugCSP6

MLWAARFIVLPLLFCVLQVWSAPADEKYTDIDFDSILANRRVLSSYVKCLTDKGPCTPQGKELKKIVPEVIQTSCTKCSPQQKKVVRNVITTMQSKYKDQWDLVVNKYDPKKQRSGELKAFLSGTD

>NlugCSP7

MIQSTQTFISIALLLFIQIAILSMASASSGTTSTTSAPKTAESASAKSSSKDEIPDQTFDRYINNERYMLMQYECLMGNKPCDHVGRKLKAAVPLVVRGLGCPKCSQREEDQMKRIVSHVQRSYPDKWQKLIKKYGN

>NlugCSP8

MSSTMLVFVAVLCFSAVLAKPADKYTTKYDNIDLDEVLSNQRLFDSYFKCLMGGKCTPDGQELRDALPDALATACEKCSEKQKEGTEKVMKFLIEKKPTEFAELEKKYDPQGTYRQKYKAEADKRGYSV

>NlugCSP9

MKSQQLLVSCLFICTWLVVLMAPSANAAPKEKDPERKALYRLEYIDIEKVLDNNRMLTNFIRCFLRQGPCTPEARDFRKLLPKLAKTMCSDCTARQRYIIKKVFKHLMEERPKEWELLMDRFDPQRKYAERLDTFMVDMTTRAPVTSSPMPSSPVTLTSSSVTMSSTTQRVIEILRTSTDMSNESRPAS

>NlugCSP10

MFMLLACSELGSGQQQQNVDNIEMSIYDKMFENMDVNSLLKNHRLVDSYLKCFLNEGSCTHIGHEVKMMIPEVIKSRCGTCGENQMRALKAGLRLFIVLRPDDWQRFLDVYDPDRKEWPHIKAFMDSDD

>NlugCSP11

MKSIILLVFVSMSAMVYRCRADEPSYPTSWDNVNIDEVLGNERLVQNYAKCLLEKGSCSPEGTELKKAIPDALKTGCTKCSDKQKAGAQKVIKWLVQKKPELWKEVVDKYDPSGEYTKKYEKEYQI

>SfurCSP1

MFNLLTLVVCLSTIAVQIQAAPEEAQYTTKYDKINLDEILNNDRLFKSYFGCLMGGKCTPDGQTLRDILPDALETACSKCSDTQKAGTEKVFKFMIEKKPSEFADLEKKYDPNGKYRARYEADAEKFGIKV

>SfurCSP2

MVLADTPTTSPKVETKAVESGKSSSKDEIPDQTFDRYINNERYMLMQYECLMGNKPCDHVGRKLKAAVPLVVRGLGCPKCSQREEDQMKRIVSHVQRSYPDKWQKLIKKYGN

>SfurCSP3

MQLLYALVFGCTLVMVSSDMPQSTYPTKYDDYNPDDILKNDRLFNQYFICLTKKKGCTTAGELLSAIIPDALATSCAKCSAKQKAIGEKVIRFLYFNKPDEFAEMSKIYDPEGKYLEMYIASGGLI

>SfurCSP4

MKCPLLSVSCLWISLLALSSSASAATKEKDPERKALYRLEYIDIEKVLDNNRMLTNFIRCFLRKGPCSPEARDFRKLLPKLAKTMCSDCSPRQRFIIKKVFKHLMEERPKEWELLMDRFDPQRKYAERLDTFMVDMTTPSTTTTTTSTTPSTPMSSTTQRIIEILRTSTEMSNESSP

>SfurCSP5

MSEILVTSLIFMLLAASELGLGQQQQTQKPQQQNVDNIEMSIYDKMFENMDVNSLLKNHRLVDSYLKCFLNEGSCTHIGHEVKMMIPEVIRSRCATCGENQMRALKAGLRLFIVRRPDDWKRFLDVYDPDRTEWPHIKAFMESDD

>SfurCSP6

MKLALFCCLLGLVAAVSAQSEKSEKPEKYTTKYDYINVDEILSNDRLFNSYYKCLMGGKCTPGGPELRTHLPDALQTNCSKCSEKQKEFSDKVIKHLMDNKPEEFSALVKKYDPEGIYKDAFKPKHNQ

>SfurCSP7

MRASKASSLVAVLLIAVWGFTGVQAQQKSKDTRYTTRFDSIDVEVILKNERIFKRYMDCLLDKGRCTPEARELKRLLPEALKTECLKCSEVQRRQGAKVMGFIIKNKRPYWDLLLAKYDPQGIFRAKYNYNENNIEGVLKQLEREQQGLYGTYSNTTNTTNTVNSTSTRK

>SfurCSP8

MLKFTLTLLVLAVVSVNCGKLYKDRYTTKFDKIDLDEALNNQRLFESYLKCLMGDKCSPDGYELREALPDALATACAKCSDAQKAGTEKVIRFLIEKRPKEYALLEKEYDPEGIYRDKYKPIAQEKGIKI

>SfurCSP9

MRCLLLVAVVFAAFIAAARADEANKYTSKYDNIDIDKILKNDRVLSQYIKCLMGEGSCTQEGRELKRLLPDAIQSNCSKCSEKQRQASVKVMRHLRQSKERDWNRLLDKYDPQGDKRKNLKLD

>LstrCSP1

MASVSSATLTAAALLALLALQLTAAQNFNEADIARMLNDSGLVQRQISCILGEAACDNIGNMLKLAIPEVLKRNCRSCNAQQASNARRLISFVQANYPAQWQRIQSRYVG

>LstrCSP2

MRCLLLVAVICAAFIAAAQADEANKYTSKYDNIDIDKILKNDRVLSQYIKCLMGEGSCTQEGRELKRLLPDAIQSNCSKCSEKQRQASVKVMRHLRQSRVRDWNRLLDKYDPQGDKRKNLKLD

>LstrCSP3

MKLALFCCLLGLVIAVSAEKYTTKYDHINVEEILNNERLFNSYYKCLMGGKCTPDGLELRTHLPDALRTNCSKCSEKQKEFSDKVIRYLIDNKPEEFAALTKKYDPEGIYKTTFGPQFKKDNTTTNQ

>LstrCSP4

MLWAAKFIVFPLIFCVLQVWSAPADEKYSDIDFESILANRRVLSSYVKCLTDKGPCTPQGKELKKIVPEVIQTSCTKCSPQQKKVVRNVITTMQSKYKDQWDLVVNKYDPKKQRAGELKAFLAGTD

>LstrCSP5

MLKFKLTLLVMASAFFSVDGGKLYKDRYTTKFDKIDLDEALNNQRLFESYLKCLMGDKCSPDGYELREALPDALATACAKCSEAQKAGTEKVIRFLIEKRPKEYALLEKKYDPEGVYRDKYKPIAEEKGIKI

>LstrCSP6

MQASSLAMLLIAVWVLSPRRPLSGGFAGVHAQQSKNTRYTTRFDSIDVEVILKNERIFKRYMDCLLDKGRCTPEARELKRLLPEALKTECLKCSEVQRRQGAKVMAFIIKNKRPYWDLLLAKYDPQGVFRAKYKYNDQNIEAVLKQLEREQQGLYGTYSNPTNTTTVNSASSRK

>LstrCSP7

MIQRTQGFNSIVVLLLIKLTVLSMVLASTHAPAPTPTPKVETKATEAAKSSSKDEIPDQTFDRYINNERYMLQQYECLMGNKPCDHVGRKLKAAVPLVVRGLGCPKCSPREEEQMKRIVSHVQRSYPDKWQKLIRKYGQ

>LstrCSP8

MKSPCLLSVSCCILVLVASSASAAPKEKDPERKALYRLEYIDIEKVLDNNRMLTNFIRCFLRKGPCSPEARDFRKLLPKLAKTMCSDCSPRQRFIIKKVFKHLMEERPKEWELLMDRFDPQRKYAERLDTFMVDMTTRATPTTTTTTIPTTTTPMSSTTQRIIEILRTSTEMSNESRP

>LstrCSP9

MSEVLVMILIFMLLAGREQRLQQQQQQQQQPQPQQQNVDNIEMSIYDKMFENMDVNSLLKNHRLVDSYLKCFLNEGSCTHIGHEVKMMIPEVIRSKCATCGENQMRALKAGLRLFIALRPDDWKRFLDVYDPDRTEWPHIKAFMEYDD

>LstrCSP10

MYDHINVYNILKNERLFNRYFTCLTKKEGCTPEGKLLAAAILDALETSCANCSNEQRKLAEQVIQYLYFNKRDKFDELAMIYDLEGVFQEYHIAEYLVSGSWMPDFRKLPPV

>LstrCSP11

MKFLYFTVFGCALVMFTSAIPEIIFTSAMPQKTYSTMYDHINVNNILKNDRLFNRYFTCLTKRGGCTPEGKLLAAAILDALETSCANCSNEQRKLARQVIQYL

>LstrCSP12

MFKNLLVVCLLVAAVSAKPKPAEKKNTTKYDNIDLDEILNNQRLFDNYYKCLLGAKCTPDGQELKEALPDALATACSKCTEKQRVGTEKVIRHLIEKKPTEYAELEKKYDPQGTYKRKYQAEAIKRGIKV

>SaveCSP4

MDSRIAVVCVVLAVFAVDQTVGAPQKDALAAGSPTTYTNKYDHIDIDQVLASKRLVNSYVQCLLDKKPCTPEGAELRKILPDALKTQCAKCSATQKNAALKVVDRLQKDYDKEWKQLLDKWDPKREQFQKFQQFLTEEKKKGVVKF

>SaveCSP5

MNCKVLIALCCVAVYAAQANPAGAATATAADDEIKDFPAYMKRFDKLNVEQVLNNDRVLASHLKCFLNEGPCVQQSRDLKRVIPVIANNGCNGCTERQMTTIKKSLNFLRTKKPVEWARLVKIYDPSGTKLNKFLDA

>SaveCSP7

MARSSSTSVTMKVFVMAVCVCAALARPEEAKMENKPTAVKSETLAAPLPTTIVKRATPQVVSIQKDASLPNVSEDVLDKALSDRRFVQRQLKCATGEGPCDPIGRKIKAHAPLVLRGMCVKCSQSEIKQIQRVMSHIQKNYPKEYTKMLKQYQSGF

>SaveCSP1

MNLLAIFCYITMMCDSQFRRLEQPTAIPQVKRIEQPATIATRIGQATIAPRFGQPTVAPRFGQPTIAPRFGQATAAPQTGEAAIGPRIGQTFQNVNDSVSPTTDGRKTTRETSSYPTRYDFIDIEAVMNNDRIIKILFNCVMNQGPCTREGLELKRIVPDAIQTECAKCNERQRKQAGKVLAHLLQYKPEYWNMLVKKFDPNNIYLRKYMADNDDDEKLSLQKLSNNTTK

>AgosCSP1

MNILTIFCYVTVMCDTQVKPAVSAQRLQSVNQNVTPTNDGRKTIRETSSYPTRYDYIDIEAVMNNERIIKILFNCVMSRGPCTREGLELKRIVPDAIQTECAKCNERQRKQAGKVLAHLLQYKPEYWKMLVQKFDPNNVYLRKYMADNDDDEKLSLQKLSNDTTKKKRNI

>AgosCSP2

MAHLNLFVVLIASLIYFTSAAEEKYTTKFDNFDVDKVLNNNRILTSYIKCLLDEGNCTNEGRELKRVLPDALKTDCSKCTDVQKDRSEKVIKFLIKNRSTDFDRLTAKYDPTGEYKKNLEKFEKERASAKPLKA

>AgosCSP4

MDSRIAVVCVVLAAFAVDQTVGAPQKDAVAASGPAYTTKYDHIDVDQVLASKRLVNSYVQCLLDKKPCTPEGAELRKILPDALKTQCAKCNATQKNAALKVVDRLQKDYDAEWKQLLDKWDPKREHFQKFQQFLAEEKKKGFTKF

>AgosCSP5

MHCKVLIALCCVAVYAVQASPAGTATAAAVSADDEIKDFPAYMKRFDKLNVEQVLNNDRVLASHLKCFLNEGPCVQQSRDLKRVIPVIANNGCNGCTERQMTTIKKSLNFLRTKKPTEWARLVKIYDPSGTKLNKFLDA

>AgosCSP6

MIKLILAIAFCVSITMTVVQTAPAKYTTKYDNVNIDEILNNDRLVASYFKCLMETGKCTPEGEEIKRWLPEAIENKCEDCSEKQKLGSEKIIKFLFEKKNDMWKQLEAKYDPQGTYRQRYAEEAKKLNINV

>AgosCSP7

MSRSSSSVTMKVFVIAICVCAALARPEDVKVENKPAVIKSETLAAPLPTNIVKRATDTIQLDSSLPNVSEDVLDKALSDRRFVQRQLKCATGEGPCDPIGRKIKAHAPLVLRGMCVKCSQSEIKQIQRVMSHIQKNYPKEYTKMLKQYQSGF

>AgosCSP8

MNNIIMNNSRGRYGIFSLLAVTIAAIMLVHQPATVRCADGGIITPQQQQQQTMMFTAPTGYYVSTYDHIDVGRLLRNNKVVSGYVKCFVNEGPCTPDGKLVKAYLLPEIIRTVCGKCTPRQKDMARMVLKHIYTYRQADFEKIMQIYDTDGKRNEILAFMNH

>AgosCSP9

MSAFCLNSFILMTMITVIVTHATFTRSTKFDDRTGIDIHLVKRDTDDVNDDENSVESDEGFFYRFTHFFQDSSDKEDDDDDEKKPDFITTFDIFKLLDEEYAMQQFYCVINEDPCDEVGMRLKATIPEEINRNCERCTSTERNNIRRILNYVKKHYPQFWKRVEPIYKKKI

>AgosCSP10

MINTRPRKLVRCIRGVSISVAKGDDAVNAENKDDDSHLVNREEIQRYMSMMEKINIDQMLNNTRLMSNNVKCFLNEGPCTAHLREMKKMVPMLVKDSCSSCTKEQKIMMKKAMDAVKARRPNDYEKLSKFFDPEGKYEKKFLENLNESK

>MperCSP1

MNLLAVFCYITMMCDSQLFKRLEQPAAISQVKRIEQPAMIANRIGQPTVAPRFGQPTIAPRFGLPTIAPQVGQAAITPQVGQAAIASRFGLPTVAPQVGQAAITPQVGQAAIASRFGLPTVAPQVGQAATTPQVGQAAIASRIGQNFQNANNSVSPTTDGRKTTRETSSYPTRYDFIDIEAVMNNERIIKILFNCVMNQGPCTREGLELKRIVPDAIQTECAKCNERQRKQAGKVLAHLLQYKPEYWNMLVKKFDPNNVYLKKYMADNDDDEKVSLQKLTNDTTK

>MperCSP2

MAHLNLFVVLVASLVCFTLAEEKYTTKFDNFDVDKVLNNNRILTSYIKCLLDEGNCTNEGRELRKVLPDALKTDCSKCTEVQKDRSEKVIKFLIKNRSTDFDRLTAKYDPSGEYKKKIEKFDSEKAAAAKH

>MperCSP4

MDSRIAVVCVVLAVFAVDQTVGAPQKDAVAASGPAYTTKYDHIDIDQVLGSKRLVNSYVQCLLDKKPCTPEGAELRKILPDALKTQCVKCNATQKNAALKVVDRLQRDYDKEWKQLLDKWDPKREYFQKFQQFLAEEKKKGVVKF

>MperCSP5

MNCKVLIALCCVAVYAAHASPAGAATAAAASADEEIKDFPAYMKRFDKLNVEQVLNNDRVLASHLKCFLNEGPCVQQSRDLKRVIPVIANNGCNGCTERQMTTIKKSLNFLRTKKPVEWARLVKIYDPSGTKLNKFLDA

>MperCSP6

MNTLLLAVALCIAITMTVVQTAPAKYTTKYDNVNIDDILNNDRLVASYFKCLMETGKCTPEGEEIKRWLPEAIENKCENCSEKQKIGSEKIIKFLIEKKNDMWKQLEQKYDPQGLYKQRYSEEAKKLNLDV

>MperCSP7

MDRSSSSVTMKVFVIAVCVCAALARPEDSKVENKPAAVKSETLAAPLPTTIVKRATPQVVSTQQGASLPNVSEDVLDKALSDRRFVLRQLKCATGEGPCDPIGRKIKAHAPLVLRGMCVKCSQSEIKQIQRVMSHIQKNYPKEYTMMLKQYQSGF

>MperCSP8

MTNNNMNSPRCRPEIFSLLAVAAIATVLVHQPSTVHCADAGVYPPQQQQQEATMFTAPSGYYVSTYDHMDVGRLLRNNKVVAGFVKCFTNEGPCTPEGRLAKAYLLPEIIRTVCGKCTPRQKDMARLVIRHIYTYRRGDFDKIMQIYDTDGKKNEIIDFMNQK

>MperCSP9

MTSFCLNSVILMTITTVIVAHAASTGMTAFNNRSGSDIHMAQRDYNENKADKAEGFFFTITNFFSRRKHDDDKPDFITTFDIIRLLDEKYAMKQFYCVINKEPCDATGLRLKATIPEEINNDCERCTATETSNIRRILNYVKKHYPEFWDRVEPIYRNNMTA

>MperCSP10

MVSKLFVSVFVLMSVVGVSYSVTEGDDDAAKVADKDLHPVNQEELKKFLSMMEKVDIDQILNNNRLMSNNVKCFLNEGPCTGQLREMKKMVPMLVKDSCSSCNKEQKNMMKKAMDAMKARRPNEYEQISKFFDPEGKYEKKFLENLNESK

>ApisCSP1

MNLLAIFCYITMMCDSQFRRLEQMTAMPQVKQPATIATRIGQATIAPRFGQPTIAPRFGQATVAPQVGQAAVTPQIGQAAIGSRIGQSFQSVNGSVTPTTDGRKTTRETASYPTRYDFIDIEAVMNNDRIIKILFNCVMNQGPCTREGLELKRIVPDAIQTECAKCNERQRKQAGKVLAHLLQYKPEYWNMLVKKFDPNNIYLRKYMADNDDDEKLSLQKLTNNTTK

>ApisCSP2

MAHLNLFVVLVASLVCFTLAEEKYTTKFDNFDVEKVLNNDRILTSYIKCLLDQGNCTNEGRELKRVLPDALKTDCSKCTDVQKDRSERVIKFLIKNRSAEFDKLTAKYDPSGEYKKKIEKFDAERAAAAKH

>ApisCSP3

MVHLNLFVVLVASLVCFTLAEEKYTTKFDNFDVEKVLNNDRILTSYIECLLDQGNCTNEGRELKRVLPDALKTDCSKCTDVQKDRSERVIKFLIKNRSAEFDKLTAKYDPSGEYKKKLEKFSA

>ApisCSP4

MDSRIALVCVVLAVFAVDQTVGAPQKDAASGPVYTTKYDNIDIDQILASKRLVNNYVQCLLDKKPCTPEGAELRKILPDALKTQCSKCNPGQKNAALKVVDRLQKDYDKEWKLLLDKWDPKREQFQKFQQFLVEEKKKGVVKF

>ApisCSP5

MNCKILIALCCVAVYAAQANPAGVATATAADEEIKDLPAYMKRFEKLNVEQVLNNDRVLASHLKCFLNEGPCVQQSRDLKRVIPVIANNSCNGCTERQITTIKKSLNFLRTKKPVEWARLVKIYDPSGVKLNKFLDA

>ApisCSP6

MNKLFLAVAFCIVTMMTVVQTAPAKYTTKYDNVNIDDILNNDRLVNSYFKCLMETGKCTPEGEEIKRWLPEAIENKCEDCSEKQKLGSEKIIKFLIEKKNDMWKQLEEKYDSKGLYRQRYSEDAKKLDIHI

>ApisCSP7

MARSSSSVTMKVFVIAVCVCAALARPEEAKMENKPAVVKSETLAAPLPTTIVKRATPYVVSTQQDSSLPNVSEDVLDKALSDRRFVQRQLKCATGEGPCDPIGRKIKAHAPLVMRGMCVKCSQSEIKQIQRVMSHIQKNYPKEYTKMLKQYQSGF

>ApisCSP8

MTNNNMNCPRSRPEIFSLLAVTTIAAVLVHQPAKVYCADGTIYPSQQQQQQTMMFTAPSGYYLSTYDNLDVGHLLRNKKVVSGFVKCFVNEGPCTPDGKLVKAYLLPEIIRTVCGKCTPRQKDMSRAVLRHLYTYRRADFDKIMQIYDTDNKKNEIINFMNQK

>ApisCSP9

MSSFCLNSVILMTVITVVVARVAFAESTTSNDRPGSDIRLVKKDVDYNEDDADDREEGFFFRISHFFGFTSYDDDKPDFITTFDLIRLLDEKYAMKQFYCVINEEPCDAVGLRLKATIPEEINRDCERCTATETSNIRRILNYVKKHYPKFWERVEPIYRNNTTA

>ApisCSP10

MVSKRFISVFMFMAVVGVSFSVPEDDDATKVVNKEVDHHSVIQEEIKKFLSMMEKINIDQILNNDRLMSNNVKCFLNEGSCTAQLREMKKMLPVLIKDSCSSCTKEQRNMIKKAMDAIKARRPNEYERVTKFFDPEKKYEKKLSEKLNES

>AglyCSP1

MNILTIFCYVTVMCDTQVKPAVSAQRLQSVNQNVTPTNDGRKTIRETSSYPTRYDYIDIEAVMNNERIIKILFNCVMSRGPCTREGLELKRIVPDAIQTECAKCNERQRKQAGKVLAHLLQYKPEYWKMLVQKFDPNNVYLRKYMADNDDDEKLSLQKLSNDTTKKKRNI

>AglyCSP2

MAHLNLFVVLIASLIYFTSAAEEKYTTKFDNFDVDKVLNNNRILTSYIKCLLDEGNCTNEGRELKRVLPDALKTDCSKCTDVQKDRSEKVIKFLIKNRSTDFDRLTAKYDPTGEYKKNLEKFETERATAKPLKA

>AglyCSP4

MDSRIAVVCVVLAAFAVDQTVGAPQKDAVAASGPAYTTKYDHIDVDQVLASKRLVNSYVQCLLDKKPCTPEGAELRKILPDALKTQCAKCNTTQKNAALKVVDRLQKDYDAEWKQLLDKWDPKREHFQKFQQFLAEEKKKGFTKF

>AglyCSP5

MHCKVLIALCCVAVYAVQASPAGTATAAAVSADDEIKDFPAYMKRFDKLNVEQVLNNDRVLASHLKCFLNEGPCVQQSRDLKRVIPVIANNGCNGCTERQMTTIKKSLNFLRTKKPTEWARLVKIYDPSGTKLNKFLDA

>AglyCSP6

MIKLILAIAFCVTITMTVVQTAPAKYTTKYDNVNIDEILNNDRLVASYFKCLMETGKCTPEGEEIKRWLPEAVENKCEDCSEKQKLGSEKIIKFLFEKKNDMWKQLEAKYDPQGIYRQRYAEEAKKLNINV

>AglyCSP7

MSRSSSSVTMKVFVIAICVCAALARPEDVKVENKPAVIKSETLAVPLPTNIVKRATDTIQLDSSLPNVSEDVLDKALSDRRFVQRQLKCATGEGPCDPIGRKIKAHAPLVLRGMCVKCSQSEIKQIQRVMSHIQKNYPKEYTKMLKQYQSGF

>AglyCSP8

MNNIIMNNSRGRYEIFSLLAVTIAAIMLVHQPATVRCADDGIITPQQQQQQTMMFTAPTGYYVSTYDHIDVGRLLRNNKVVSGYVKCFVNEGPCTPDGKLVKAYLLPEIIRTVCGKCTPRQKDMARMVLKHIYTYRQADFEKIMQIYDTDGKRNEILAFMNH

>AglyCSP9

MSAFCLNSFILMTMITVIVTHATFIRSIKFDDRTGIDIHLVKRDTDDVKDDENSVESDEGFFYKITHFFQHHDKEDDDDDEEKPDFITTFDILKLLDEEYAMEQFYCVINEDPCDEVGMRLKATIPEEINRNCERCTSTERNNIRRILNYVKKHYPQFWKRVEPIYKKKI

>AglyCSP10

MNSKIFISVFMFITIVSVSISVAERDDAVKAENKDDDSHPINREEIQRYMSMMEKINIDQMLNNTRLMSNNVKCFLNEGPCTAHLREMKKMVPMLVKDSCSSCTKEQKIMMKKAMDAVKARRPNDYEKLSKFFDPEGKYEKKFLENLNESK

**Table S4.**

| **Gene** | **Forward primer (5’-3’)** | **Reverse primer (5’-3’)** |
| --- | --- | --- |
| *OBP2* | AAACTCTACCACACCAAA | TTCGCAAGTCTCTATTATG |
| *OBP3* | ATGATTTCGTCGACGTTTTACATAA | TCAAGTTGACTTGTCGAGATCCA |
| *OBP4* | AACAAGAACCATCAGGAA | ACGCATTCAAACATTTCA |
| *OBP5* | ACAAGTAGCAGAGAAGTT | ACAGTTCGATTCGTATTG |
| *OBP6* | ATTACCTTCGTGTTGTCAA | TCCTGCTGGAGTATTCAT |
| *OBP7* | GATGCTTACTTGAGTGAA | TCTTCGCCTTTATCTATTC |
| *OBP8* | GTCCGCCACTATATTCCA | TTCCTCCACGATACAAGT |
| *OBP9* | TTCAATCAATAAGGCTACTG | TTTGGCATTTGTCGTAAA |
| *OBP10* | ATGGAACATTTACGTAGCACAAAC | TCACAATGGCAACAGTTGAATAGT |
| *CSP1* | CGTCGTCATATCCAACTC | TACCTTCTCGTCATCATCA |
| *CSP2* | TTCACACTGGCAGAAGAA | GGTCAAACGATCAAAGTCA |
| *CSP4* | ATGGATTCACGAATTGCAGTAGTC | TTAAAATTTGACAACACCTTTTTTCTT |
| *CSP5* | ATGAACTGCAAGGTCTTGATCG | TTACGCGTCGAGGAATTTGTT |
| *CSP6* | ATGAACACACTTCTCCTAGCAGTTG | TTAAACATCAAGGTTCAATTTCTTTG |
| *CSP7* | CGAGGACGTACTTGATAA | TTAGAATCCGCTCTGGTA |
| *CSP8* | ATGACGAATAATAATATGAACAGTCCG | CTATTTTTGGTTCATGAAATCGATG |
| *CSP9* | ATGACGTCGTTTTGTCTGAACTCT | TTAAGCTGTCATATTATTTCTATAAATTGG |
| *CSP10* | CCTTCATCCTGTCAACCA | TCTTTTCGTATTTTCCCTCTG |
| *β-actin* | ATTGCGTGTTGCCCCAGA | CTGTTCGAAGTCCAAAGCGAC |

**Table S5.**

| **Gene** | **Forward primers (5’-3’)** | **Reverse primers (5’-3’)** | **PCR efficiency** |
| --- | --- | --- | --- |
| *OBP2* | GTGACCATAATAGAGACTT | ATGCTTTAGGGAAGAAAT | 1.92 |
| *OBP3* | AAAGTGTCTGATGAAATG | TATAGCCTCAATCTTCTC | 1.91 |
| *OBP4* | AACAAGAACCATCAGGAA | ATTGCCATCATTGAGGAT | 1.92 |
| *OBP5* | ACAAGTAGCAGAGAAGTT | TCTTCAGTGAGCAATCTA | 1.94 |
| *OBP6* | CAGCAGGAAGATTGATGAA | GGGCATTTCTGGACATTT | 1.95 |
| *OBP7* | GATTGCGATGCTTACTTG | TTATGTTGTTGTTGTTCTCC | 1.92 |
| *OBP8* | CGCCACTATATTCCAGAG | ATCAGACAACCCATCATAC | 1.93 |
| *OBP9* | GGAAGAACTTGGATTGATAA | TTTGGCATTTGTCGTAAA | 1.94 |
| *OBP10* | ATGGCATTGTTGGTAGTA | CTTTCTTCATTTCTTCCGTAA | 1.93 |
| *CSP1* | TACGACTTCATTGATATTG | CACATTCTGTCTGTATTG | 1.94 |
| *CSP2* | GCCGAGAATTGAGGAAAG | TCAAACGATCAAAGTCAGTAG | 1.90 |
| *CSP4* | ATGCTGCTCTTAAAGTAGT | TTCTTCTCTTCTGCCAAA | 1.93 |
| *CSP5* | AAGACAGATGACCACCAT | TCGAGGAATTTGTTCAACT | 1.92 |
| *CSP6* | CGATGACATTCTGAACAA | TTCTCTATTGCTTCTGGTA | 1.95 |
| *CSP7* | TAGTGTTGAGAGGAATGTG | TTAGAATCCGCTCTGGTA | 1.94 |
| *CSP8* | TGTTGCCCGAGATCATAC | CCATCCGTGTCGTAAATC | 1.95 |
| *CSP9* | CGGGCAGTGATATACATAT | CGAAAGTGGTAATGAAATCC | 1.94 |
| *CSP10* | CCAAATACTGAACAACAA | TACACGATGAACAACTAT | 1.96 |
| *β-actin* | AGGTATTGCCGACAGAAT | GACAGAGAAGCCAAGATG | 1.91 |
| *GAPDH* | TTCTGTTGTTGACTTGAC | CTTCATCTTCAGTGTAACC | 1.92 |

**Table S6.**

|  | MperOBP2 | MperOBP3 | MperOBP4 | MperOBP5 | MperOBP6 | MperOBP7 | MperOBP8 | MperOBP9 | MperOBP10 |
| --- | --- | --- | --- | --- | --- | --- | --- | --- | --- |
| MperOBP2 | 100 |  |  |  |  |  |  |  |  |
| MperOBP3 | 22 | 100 |  |  |  |  |  |  |  |
| MperOBP4 | 17 | 35 | 100 |  |  |  |  |  |  |
| MperOBP5 | 13 | 30 | 26 | 100 |  |  |  |  |  |
| MperOBP6 | 8 | 30 | 27 | 32 | 100 |  |  |  |  |
| MperOBP7 | 22 | 52 | 36 | 27 | 29 | 100 |  |  |  |
| MperOBP8 | 21 | 48 | 31 | 29 | 29 | 46 | 100 |  |  |
| MperOBP9 | 19 | 44 | 32 | 32 | 30 | 49 | 48 | 100 |  |
| MperOBP10 | 22 | 50 | 35 | 27 | 32 | 59 | 46 | 50 | 100 |

**Table S7.**

|  | MperCSP1 | MperCSP2 | MperCSP4 | MperCSP5 | MperCSP6 | MperCSP7 | MperCSP8 | MperCSP9 | MperCSP10 |
| --- | --- | --- | --- | --- | --- | --- | --- | --- | --- |
| MperCSP1 | 100 |  |  |  |  |  |  |  |  |
| MperCSP2 | 16 | 100 |  |  |  |  |  |  |  |
| MperCSP4 | 18 | 67 | 100 |  |  |  |  |  |  |
| MperCSP5 | 15 | 62 | 60 | 100 |  |  |  |  |  |
| MperCSP6 | 16 | 71 | 65 | 62 | 100 |  |  |  |  |
| MperCSP7 | 10 | 49 | 50 | 52 | 49 | 100 |  |  |  |
| MperCSP8 | 13 | 55 | 51 | 54 | 53 | 51 | 100 |  |  |
| MperCSP9 | 10 | 46 | 44 | 51 | 47 | 54 | 50 | 100 |  |
| MperCSP10 | 14 | 59 | 59 | 62 | 59 | 50 | 54 | 47 | 100 |

**Table S8.**

>SaveOBP1

MLNLKVMMFLCLSVTVVYCEIEENRLNNNTAIEICILETNIPKDEFQAMVTMPNNPDVDILTTRAQKCMLGCVMRKNHIINDGYVSTDVLYRYVMNFYGAVPNTKRKLLSRTVSKVIDICTKKDNLPTEECVLADLIMTCVRSEALKRGLQR

>SaveOBP2

MKVSAATAVLVALVATVQSSDPCNISTCYKSGTTKPPMAVTPTRLPVQSSSTPTSHPQTTYAKDHVHGSTTIKSGANATATTASGASVNGTERPIVVKSSAGVIGNSTTPKPTMTEGHVALKQKLNTIAVKCKDELHAPQEIMALVSNTVVPQNEQQRCYLECVYKNLNLIKNNKFSVEDGKAMARIRFANQPEEHKKAVTIIETCEKEAIIDPKITEKCAAGRIIRNCFVKNGEKINFFPKA

>SaveOBP3

MISSTFYITSVFGIALLISCGYGRFTTDQIDYYGKACNASEDDLVVVKSYKVPSTETGKCLMKCMITKLGLLNDDGSYNKTGMEAGLKKYWSEWSTEKIENINNKCYEEALLVSKEVVATCNYSYTVMACLNKQLDLDKST

>SaveOBP4

MRGNYSLMVFLLFAIGLQDIFCQKQEPSGKCRAPDKAPLNLEIIINICQEEIKSALLQEALDILNDGNLEQNTPSHSSRSKREADEDLTNEERRVAGCLLQCVYKKVKAVDETGFPVVDGLMKLYNEGVQDRNYYMATLSAVRHCISIAQQLKQQQPSKSFDDGQTCDLAYEMFECVSEKIEENCGVENKSNNLSQRQV

>SaveOBP5

MSVNSATIKCIAVAVVLLQISIIFADAGHHRRGKELLDTEDSDFFRCKQASRKSCCGPENAMKRFGDKDKVAADECYAQVAEKFATVTATTPKQDLFSAEAVKITKKKQFCLHECIGKKNNLLTEDGSLNKTFIADYAMKSVFKEQWQKQVGQKALDKCLEETYIPWPAEDKENVCNPVYVQFQHCLWLQYESNCPANKIKTTKKCEKTRNRYRMQKSTSN

>SaveOBP6

MQKVVFICIFAIICQTVFTAGYDRTWILRQKRMTNDDECRTLIPSPEKKLPSCCQMPDILPNSNSTWEKCFETFKQFKDKPETKEYKEMAHGKEPPCLFQCIFMQSGLTTSDGKLNEDAITKKMSEGINNDEKWKSTWQNSLNKCFDDVKQEDKKQIPIMNTPAGRLMKCFLRDMYMSCPKNVWVESSECLNMKDLVQKCPEMPPPVFKSPPKLI

>SaveOBP7

MYNMLPKTVLFAIIAATVLKDCDAYLSEAAIKKTQQMLKTVCSKKFSVEEDVFTDIKKGIFPEDNNNIKCYFACNFKTMQLINQKGSIDKKMFKDKMTMMAPPNVLKVLLPVIEQCTGIDKGEELCQSSYNLIKCAHTVDPRSLEYLPL

>SaveOBP8

MFALKVACLCLSVAVVFGENNQQNGPSDRSASIFQSCIAETKLSGDALKGFRSMSIPKTQAEKCMMGCLMRKVNVINKGKFSVEEATKVAQKYYGTNEVMMKKAKDLIDVCAKKAQSTTEECALAGIVTTCIVEEAQKAGLAGGPGSRSRRTVSPKFRRDAM

>SaveOBP9

MIIKKTLLVSVFVLFGCLFSINKAADDADAGDKELMSKLFTVVLKCFKDADWGTCGEMITTKYDITQAKYKQCTCHMACAGEELGMINTSGQPEPAKFLEYVNKINHPSIKSQLQLIYDKCHNVKGSEKCDLAEQFAICAFKESPALKERAATLMEMLVKMKPKSK

>SaveOBP10

MEHLRSTNVVFAIVMALLVVQSSTRPQPDEMEEIKKTLYNACAGKFPITEEIKKNAKNAIISDDPTFKCFLKCCFDEMSLIDEDGILDGDSMKAMAPDHIKPIFEQVIPSCLKNVKQDGCEASFEFISCGMKLNPLTVELLPL

>SaveOBP13

MDACTVHCVFNQLEMLNSNSRPDKYSIVNIMTNQIKDVELKEFIQDSIDECFDTLELDSNNNKCEFSKNFAVCMENKAQRNCDDWDENLSANKINSAGLQDGTNQQDKRKGY

>AgosOBP2

MKVSAATAVLVALVATVQSSDPCNISTCYKSGTTKPPTTVTPTRLPVQSSSTPTSHQQTTYAKDHVHSSTATKSGVNTTATTTSGASVNGTERTTVVKSSSGVAGNVTTPKPTMTDGHVALKQKLNTIAVKCKDELHAPQEIMALVSNTVVPQNEQQRCYLECVYKNLNLIKNNKFSVDDGKAMAKIRFANQPEEHKKAVTIIETCEKEAIIDPKTTEKCAAGRVIRNCFVKNGEKINFFPKA

>AgosOBP3

MISSTFYTSLMFGIAMLISCSFGRFTTEQIDHYGKACNATEDDLVIVKSYKVPTSDTGKCLMKCMISKLGLLNDDGSYNKTGMEAGLKKYWSEWSTDTIESINNKCYEEALLVSKDIIATCNYAYVVMACLNKQLKLDNST

>AgosOBP4

MRGNYSLVVFLLFGFGLLEIYCQKQELSGKCRAPDKAPLNLEIIINICQEEIKSALLQEALDILNDGTLEQNTPSYSRSKRDADEDLSNEERRVAGCLLQCVYKKVKAVDETGFPVVDGLMKLYNEGVQDRNYYMATLSAVRHCISIAQQLKQQQPSKSFDDGQTCDLAYEMFECVSEKIEENCGVENKLNNLSQRQV

>AgosOBP5

MKMSANGATMKCVAVAVVLFQMSVIFAEAGHQRRGKELLDTEDSDFFRCKQASRKSCCGPENAMKRFGDKDKVAADECYAQVAEKFATVTATTPKQDLFSGEAVKITKKKQFCLHECIGKKNKLLTEDGSLNKTFIADYAMKSVFKEQWQKQIGQKALDKCLEETYIPWPAEETENKCNPVYVQFQHCLWLEYESNCPDNKIKLTKKCEKTRNRYRMQKSPSNQ

>AgosOBP6

MQKVVFLCIFAIICQTVFTVGFERTWILRQKRMTNDNECRALFPSPEKKLPTCCQMPNILPGLDNAWEVCFEKFKQFKDKHATKEYKEMVHENEPPCLFQCVFMQSGLTTSDGKVNEDAVIKKMAEGMDNDEKWKSIWRNTFNKCLNDVKQEDKEQIKVMNTPTGRLMKCFLRDLYMNCPKNVWVENSECSNLKDLVEKCPKLPPPVFQSPPKLI

>AgosOBP7

MNMLPATVLLAVVAATILKDSDAYLSEEAIKKTQKMLKNVCSKKHSVEEEVFTDIKKGIFPENNNNIKCYFACNFKTMQMVNQKGILDKKMFKDKMTMLAPPNVLAILLPPIEQCIGNDKDTEICQSSYNFIKCAHRVDPKSLEFLPL

>AgosOBP8

MFAFKVACLCLSVAVVFGENNQQNSNDRSASIFQSCISETKLSGDALKGFRSMSIPKTQAEKCMMGCLMRKVNVINKGKFSVEEATKVAQKYYGTNESMMKKAKDLIDVCAKKAQSTTEECALAGIVTTCIVEEAQKAGLTGGPGSRSKRTVSPKFRHSIV

>AgosOBP9

MIIKKTLLVSGFVLFGCMFSINKAADDADTADKELMSKLITVAFKCFKDADWGTCGEMITTKYDITQAKYKQCTCHMACAGEDLGLINSNGQPEPAKFLEYVKRINNSVIKSQLQHIYDKCQNVKGTEKCDLAEQFAICAFKESPEMKERVTKLIEMLVKMKPKSK

>AgosOBP10

MEHLRGTNVMFAIVMALLVVQSSTRPQPDEPDDIKKTLYNACSEKFPLTEEIKNNVKNSMVIDDQNFKCFLRCCFDEMSLIDEDGIIDGESLAAMAVDKIKPVAEKIVHDCLPAGKQEKQDGCEAAFKFFSCGMKLNPLTIELLPLQ

>MperOBP2

MKVSAATAVLVALVATVQSSDPCNISTCYKSGTTKPPMNVTPTRLPVQSSSTPTSHPQTTYAKDHAHGSTTVKSGANATATTASGASVNGTERPAVAKSSAGVTGNSTTPKPTMTEGHVALKQKLNTIAVKCKDELHAPQEIMALVSNTVVPQNEQQRCYLECVYKNLNLIKNNKFSVDDGKAMARIRFANQPEEHKKAVTIIETCEKEAVIDPKTTEKCAAGRVIRNCFVKNGEKINFFPKA

>MperOBP3

MISSTFYITLLFGIAMLISCGYGRFSTEQIDYYGKACNASEDDLVVVKSYKVPTTETGKCLMKCMITKLGLLNDDGSYNKTGMEAGLKKYWSEWSTEKIEAINNKCYEEALLVSKEVIATCSYTVMACLNKQLDLDKST

>MperOBP4

MRGNYSLTVFLLFVIGLQDIYCQKQEPSGKCRAPDKAPLNLEIIINICQEEIKSALLQEALDILNDGNLEQNTPSYSSRSKREADEDLTNEERRVAGCLLQCVYKKVKAVDETGFPVVDGLMKLYNEGVQDRNYYMATLSAVRHCISIAQQLKQLQPSKSFDDGQTCDLAYEMFECVSEKIEENCGVENKSNNLSQRQV

>MperOBP5

MSANSATIKCIAVAVVLLQISVVFADAGHHRRGKELLDTEDSDFFRCKQASRKSCCGPENAMKRFGDKDKVAADECYAQVAEKFATVAATTPKQDLFSADAVKITKKKQFCLHECIGKKNRLLTEDGSLNKTFIADYAMKSVFKEQWQKQVGQKALDKCLEETYIPWPAEDKENVCNPVYVQFQHCLWLQYESNCPDNKIKITKKCEKTRNRYRMQKSTSN

>MperOBP6

MQKVVFICIFAIIYQTVFTVGYERTWILRQKRMTNDDECRTLLPSSEKKLPSCCQMPNILPGLDSTWEKCYEKFIQFKDKPETKEYKEMSHGKEPPCLFQCIFMESGLTTNDGKLNEDAITKKMTEGINNDEKWKSTWKKSLDKCFDDVKQEDKKQILIMNTPAGRLMKCFLRDIYMNCPENVWVESSECLNVKNLVQKCPEMPPPVFQSAPKLI

>MperOBP7

MNNMIPATVLLAVIAATVLKDCDAYLSEAAIKKTQQMLKTVCSKKHSVEEDVFTDIKKGIFPENNNNIKCYFACNFKTMQMINQKGTLDKKLFKDKMSMMAPPNIYNILLPAIEQCIGIDKGEELCQSSYNFIKCAHRVDPKSLEYLPL

>MperOBP8

MFVLKVACLCLSVAVVFGENNQQNSSDRSATIFQSCIAETKLSGDALKGFRSMSIPKTQAEKCMMGCLMRKVNVINKGKFSVEEATKVAQKYYGTNETMMKKAKDLIDVCAKKAQSTTEECALAGIVTTCIVEEAQKAGLAGGPGSRSRRTVSPKFRRNSM

>MperOBP9

MLIKKTLLVSVFVLFSCLFSINKATDDADTADKELMSKLFTVVFKCFKDADWGTCGEMITTKYDITQAKYKQCTCHMACAGEELGLINSSGQPEPAKFLEYVNRINNPGIKSQLQHIYDKCQNVKGTEKCDLAEQFAICAFKESPALKERATTLMEILMKMKPKSK

>MperOBP10

MEHLRSTNVVFAIVMALLVVQSSTRPQPDELEEIKKTLYNACAGKFPITEEMKKDILNSNMVDDQNFKCFLRCCFDEMSMIDEDGIIDGESLISMATDNLKPVIQQVVQSCVKDIKQDGCEAAFNFISCGLKLNPMTIQLLPL

>ApisOBP1

MLNLKVMMFLCLSVIVVYCESDQVPINSSAAVESCLLETNMTRDEFEDMLTSPNARELTILKSHAHKCMFGCVMRKNHIVNDGVVSKEVLSKYVLNFYGRPDYKRRLIIKDVEHIVDVCAKKVADESETDECELAATLVTCIVLEANKAGLVDDPARQI*

>ApisOBP2

SDPCNISTCYKSGTTKPPMAVTPTHLPVQSSSTQTSHPQTTYAKDHVHGSTTTKSGVNATVTTASGASVNGTEPPAVVKSSAGVTGNSTTPKPTMTEGHVALKQKLNTIAVKCKDELHAPQEIMALVSNTVVPQNEQQRCYLECVYKNLNLIKNNKFSVEDGKAMARIRFANQPEEHKKAVTIIETCEKEAVIDPKTTEKCAAGRVIRNCFVKNGEKINFFPKA

>ApisOBP3

RFTTEQIDYYGKACNASEDDLVVVKSYKVPTTETGKCLMKCMITKLGLLNDDGSYNKTGMEAGLKKYWSEWSTEKIESINNKCYEEALLVSKEVVATCNYSYTVMACLNKQLDLDKST

>ApisOBP4

QKQETSGKCRAPDKAPLNLEIIINTCQEEIKSALLQEALDILNDGNVEQNTPNYSSRSKREAEEDLTNEERRVAGCLLQCVYKKVKAVDETGFPVVDGLMKLYNEGVQDRNYYIATLSAVRHCISIAQQLKQQQPSKSFDDGQTCDLAYEMFECVSEKIEENCGVENKSNN

>ApisOBP5

DAGHHRRGKELLDTEDSDFFRCKQASRKSCCGPENAMKRFGDKDKVAADECYAQVAEKFATVTATTPKQDLFSAEAVKITKKKQFCLHECIGKKNNLLTEDGSLNKTFIADYAMKSVFKEQWQKQVGQKALDKCLEETYIPWPAEDKENVCNPVYVQFQHCLWLQYESNCPANKIKITKKCEKTRNRYRMQKSTSN

>ApisOBP6

PNILPNLDSTWEKCFETFKQFKDKPETKEYKEMAHGKEPPCLFQCIFMQSGLTTSDGKLNEDAITKKMSEGINNDEKWKSIWQNSLNKCFDDVKQEDKKQILIMNTPAGRLMKCFLRDMYMSCPKNVWVESSECLNMKDLVQKCPEMPPPVFKSPPKLI

>ApisOBP7

YLSEAAIKKTQQMLKTVCSKKHSVEEDVFTNIKKGIFPEDNNNIKCYFACNFKTMQLINQKGVIDKKMFKDKMSMMAPPNVYKILLPVIEQCTGKDKGEELCQSSYNVIKCAHSVDPKSLEFLPL

>ApisOBP8

ENNQQNGPSDRSATIFQSCIAETKLSGDALKGFRSMSIPKTQAEKCMMGCLMRKVNVINKGKFSVEEATKVAQKYYGTNEAMMKKAKDLIDVCAKKAQSTTEECALAGIVTTCIVEEAQKAGLSGGPGSRSRRTVSPKFRRDAM

>ApisOBP9

DDADAKDKELMSKLFTVVFKCFKDADWGTCGEMITTKYDITQAKYKQCTCHMACAGEELGMINASGQPEPAKFLEYVNKINNPDIKSQLQLIYDKCQNVKGSEKCDLAEQFAICAFKESPALKERVSTLMEMLVKMKPKSK

>ApisOBP10

STRPQPDEMEEIKRTLYNACAGKFPITEEIKNNAKNSIISDDPTFKCFLKCCFDEMSMIDEDGIIDGDSLKAMAPDHIKPILEQVIPSCTKNVKQDGCEASFEFISCGIKLNPLIVALLPL

>ApisOBP13

CTIHCVFNQLEMLNSNSRPDKYSIVNIMTNQIKDVELKEFIQDSIDECFDTLELDSHNNKCEFSKNFAVCMENKAQRNCDDWDENLSANKINSAGLQDGTNQQDKRKGY

>AlucOBP1

MCSKYFVMLIGLTVYTSAEVINEECKDRNQSSTEYETFYNCCDLESSFNETKSKEKEEAREFCENEFEKANNVSEDEAEPSPSSVRQDCYVDCILKKLGAMSEDYKMDKEKVTKWFMEGTHKDFEEVGKQAMEKCYDKTYSKKHCASRVMGLLWCYSEELVMNCPAKYWDQSEKCTAAKAYMKKCSTNPWRSED

>AlucOBP2

MRSTGSECFEEIDAKLGNKTSLESDMDPYNCEKVKRMKKRHYCMHECKAKKLGVATEEGNLEFPKVKELLLSRVNETWQKDILGQAADTCATSKFDQTWKDDTEEYKCNPQALQFKHCVWKQVEMKCPEEHQNTGRHCKKLRSKISSETSKDIAKETSV

>AlucOBP3

MFSSATLVCLFAVALTQGQLDEDPECRPPHPPGKDDKCCTIPELIVGENMQAMMKQCFEESGMERRPPGPPGSGTPPTPEEIEAHRSAHECVDECFFKAAKFMNSDGEFDLEAMKTAAASVFTGDWAPLGSETIDKCFASAKSQVSASAKCTSGAHRAKKCILRNFIINCPPSAWNDSTDCAALKARLTKCSNAMPPFPHHKH

>AlucOBP4

MEVAACLVLLAALAALTAAVEEGRPLCKAPTTAPRKLEKVINQCQEEIKYALLQEAPSVLGETVGLKTALTRNRSKRETFTGEERRIAGCLLQCVYRKMKALDETGFPTATGLVKIYSEGVEDRNYYLATIQGVQRCLSRELQSRNTNPSIVKAEGYSCDVAYDMFNCVSEQIEQLCGTSP

>AlucOBP5

MNSIIVLCLVASAVTLSQGNPTTPNPSTSHVSSSAGITVSGVSKSPEEIKLKIKEQVATLTGACKTQTKLTGEQAKIVASQAIPKTEAEKCFLECIYQGLQLTKDGKFNEPAARAWAQKRFGNAPEDLQKANTMIDICVKEVVVKDENEKCALGRLIRECFVKNGAKINFFPKP

>AlucOBP6

MYDRFKLFALLALVVSCKSAPPEEPAECKLPESDSAELVKCCKLNVVLDEMADSVGECMKLVKGKPEKGPPVPEGFDCMDTCVFSKLGFAANNKLDAEKLTKKFSELFKGDWSALSDSTLKKCLPMAEGAKGSCASGADVFKFCIVRELYMNCPASSWTKSDLCKANVERLEKCPHSMPFLPGTGIKKN

>AlucOBP7

MNPLILILLVVFAAATRGEEQANALVAKAFNKCFGEFPLGDDEMKEVKDKSTVPSSHNAKCLMACMLKEGRILRGGKYELENAILMADVLNKNDHAATDKAKQLIETCAAQVGTDASADECEFAYKMALCASDEAKKLGVRPPDF

>AlucOBP8

MVLKMKQILVVFVALQVLISTTEAVMTQAQMKQAMKTVRNMCIPKSGVDKEALAKMVEGEFDESDQKLKCYLGCVLGMMQAVKNNKINLTMVKNQISKMLAPEQGQRILAAFEGCATVTGDDN

CDLAFKFAKCIYDTDKELLFQAFIVP

>AlucOBP9

MKSFVGLIFAVALVEFASAITKEYHDRAVAAKDACLKKHPSIKESDVQEFLKKHKLPETDDGKCMIACYMEEMNLMADGKINVEEAKKTNSDKYDGEPDNKELADKLIDHCSSQVSPDGMSKCEYAYQISKCGLEYGMKNGLTPPKMYEEQRR

>AlucOBP10

MTYHVFFRKFDLPRISRRVRQCYYHSVPRSLSGSSRRMLEETSQHHPKRRSRVSEKHKLPETDDGECMIACYMEEKNLMADGKINVKEANQTNSDKYDGEPDNKQLAEKLIDHCSSQVSPDGMSKCEYAYQFSKCGLEYGMKNGLTPPKMYEEQRR

>AlucOBP11

MGSQYERTLVGVRYLPIMKRVKFILVLSLLSRCSSAPTDDMAACMQITNEDSASMATCCDYVIPFSNKTMTTCDKKETSGEMSKEFECVQDCLFSSDNVLGADKKFDPVAWRKHATNTISGDWKGVIANSGSNCEGFKKVLAQSMEKKCPTSESDVSFNCMTLQWYMNCPKSAWTSSESCEASKKKLMSCFGPIFENTS

>AlucOBP12

MTCSHFIALLSVVALSLSSGEINEECKDIENLKTQLENFYGCCDFESMIERVVRTEEEVETDRFCREERKKINSTDGKVPLASEGHDCFMECVLKRMGAMGQDFKFIREKLDDFFLRGYPEEVKQAGKLAFDKCLSKNFSKKYCASGINGLMMCLPEELVMNCPANIWSSHESCPIAKEAIKKCPSYRVMIEQE

>AlucOBP13

MKHSSCVVPVALTIFVVAIVSGFKELDDVLPKPKQDECRKESNFQAELPSDINQNITQELKCFAACSLVKLGLMNEKDGTINMAQLEDLIAKHTGGKDAADMFKHTVVEPCMKEVNKTTDYCEYSFQLVKCGMSKVKPPSTGTEG

>AlucOBP14

MALNAKAVLLLGVCGLVYVSAYQEVLKATLKDCKGGKEITQEEVDEFMKPLIPKNEEERCLMACVFRAYNVIVDGHFDPKLAYGVAKNILHENPEKLKHIKETLDYCGHEIPTKMDNECDLAGEVMSCRNKYNIDHGYDQDP

>AlucOBP15

MMRPTAYYLFASYAALLVCVHFASVSAITPELDKRAKAAVAKCADVPRTDEAKKEDCHAGCFMSAMGYMTNGEINVKNMEEANKQKWDDQEIIKKGIQVDTTCAKQVGDTKGKSECTIGYEFSTCKKELVKKVGLPPPTPLKE

>AlucOBP16

MKRLVFVLFTLCSLQWVSGITDELKQKAQAARLTCKQQVGLSDKEFNDWVKGIALPTTDGGTCCEVCACWMRELGYLTGGRVNLENMKAVNAQKWNNLAYVELGNKIDALCSDRVLQTGRKECEIAVDFRKCKTELIQQFGGPPKPGST

>AlucOBP17

MRILVLFTAALTCVLAGELPEEMREMAQGLHDSCVGETGVDNGLIAPCAKGSFADDPKLKCYFKCVFGNLGVISDDGELDAEAFASILPDNMQALLPTIRGCGSTTGADPCDLAMNFNKCLQKADPVNFMVI

>AlucOBP18

MHAAIVLIGSALLVAYVSGAPSANVKEIVQNVSKKCAAETKASPDQAKIVLSKNIPKDDAERCFLQCVYTGVGVIKDGKFSEEGGKKLVALRFHDAKEKELANKLIATCAKEIKAKDGEKCSLGRAVRECFVNHGKQVNFFPSA

>AlucOBP19

MNSRFGIVFASLALLHITNAGNIKEGYVAKIAEIKDKCLKEHNVDHSVVEDLLKKSIKPEVKAAQCMVACFFEENGMMKDGKIVSEMVKSNNAHQYEDPADVEKANEASDMCDGEVSTDGKDKCLLAADYALCWVKRTEEAGLPQIDFANSS

>AlucOBP20

MYTFKTFFVLTLASYVIAAPPADEPAECKPMKEKEEEISKCCKLEPVTVKEQAAFVDCMKLVKDTDKKGPPKPEGFECLDDCILSKTGSLGSDKKIDPAKINAAAKTTYTGDWAEPGAKMVEKCLAQVAENKDKTVCSTSGADVYTKCIFRESYINCPEKSWTNSDACKANKERVIKCPKTLPYNAEQHKAETR

>AlucOBP21

MKFFVVSAALVLLVAAAVKANEKKANEKVTEIFNKCKETWPVTDEEIEQVKQKQSIPDSKNVKCILACMLKEAKILRDGEYNKDNAELMADVLYKDEPEHAEKSKQIIEMCSSELGTKTEGDDCEYAYKMSVCASKHAKELGVKTPEF

>AlucOBP22

MSLKIHFFVFAAIGAACVCAYQDQLKQTIKDCQGGKEVTDEELEEFTKPLIPKNEEERCIMACVMRTYNIINNGHYDPKIAFGIIKGILKDHPEKLDRIKEVMDHCGEDVPQHMDNECDLAGEIMQCEVKYQKAMGLN

>AlucOBP23

MYVFTVALSFALLNIVFTHPGHFDEDPECRPPHSHRHEEKECCKTPNLFSKNKDEMHELVHKCFEEAGIKKPHHGHHGPPPPGDEPPPPPPPPFHSKNNTKFECVEQCFLKNLELIDEEGDLKIDDFKALVGEKYTGDWASVGSAALEKCLEKTKTEEKESSKCKAGSKHVLLCIARESFINCPASDWTESEVCSDAKERVVKCPDIPPPMNH

>AlucOBP24

MSTKLRSVGMILAIAITHVCAYQEQLKETIKQCQDGREVTDDEVEEFTKPLVPKNQEERCLVACVFKEYKVIIDGHFDPVNALNVAKMVYKDYPEKWKRIKDVIDHCGEDIPTHNDNECDLAGDIMNCEVKYLNSMPKGVSLELLAGSIAATAEP

>AlucOBP25

MFTSTIFAVFLFSVALTQGQMDDDPECRPPPPPNKEGSCCTVPRLLDNADKPEVIKKCHDEAGMKRPSGPPGSGTPPTAEEMAAHKSAHECADECIFKSSNLLKSDGELDQDAIKATTTKMFTGDWSTIASTAVEKCLATAKSEVGASAKCKSGAHQMVKCFARTMFLNCPASSWTESTECAAAKTRLTKCPNAMPPPPHHSRH

>AlucOBP26

MNPTVAIIFTLLVAYVKANTKELSPSEALKQKVKVQCQQEVKATPEQLKIYDNFKDVPKDDVENCLMECMYTKTGGIGADGKYSVEGFKKLVDMKYKGEENTKARKIAADCEAKAAPKEGEKCSMGRAIRECLAAATKENEFFTI

>AlucOBP27

MARKFIKSCYTLVALLVFVGSIHVEAKELTEEQRTQLFEDLKQCKNSTDLSDDEFETIIAKKELPTSEAGKCFTKCLMEKLDIIEDAEGGKKKISVITMQASLEENMEKEDDIAKGKDIIQKCGDTVEPEDSCAYAYNISKCIYDRMKEAGISQ

>AlucOBP28

MIIEIICVLTVGISPHFIEGQELPPPGGVGNKTAVFKESFIRTAKYCSSIHETSTVAVLAILMSEESDDQNGKCFLNCMLQRYQLMSKQGAYNKDKFKPFLDYIPESRFLQSIKGNLKTCITERDPAPCEKAYKFIKCFYTRARNKDEFGKIQRK

>AlucOBP29

MNRPLLLLTAVLAVGSGQQEDCKTAPAGWPRRPPQCCDLPFPLEGMKKEFGSCIRQIGNRQSSAVPTAQAVRDARLCIEECVYKGLGFMEEHNLNKDQILQQLTKGVADKKDWTKPMEDAVKSCHETITKRETPQEGTCKDSAHEFTHCVMRQLFLSCPASEWNNNDECNLVKSRMQACPNIPPPPPPPPQGFRGQGPPPPQ

>AlucOBP30

MNAHIVLCLVASVFALSQGTPTTPTPATSRRVTVAPEDLEQAKSLRKFCTAKTGFTGITTTETSKGKDQARTATTPRPKTQLEKCYLECLYTGLQLTKDGKFNEPGARALANKRYKNAPEELRKVNSIIDFCITEVVVRDIEEMCALGRLIKECFSKYGAKNFPEL

>AlucOBP31

MFTSATFTVFLFAVTLTRGQIDEDPECRPSGPPGKEPECCTIPMKLFGDEVQEAVVKNCFDEAGMKRPSGPHGGGSPPTAEEMAAHISAHECADECVFKSGNFIKSDGGLDEDAIKAVIAKLFTGDWAPIATAAVNKCLASAKSGVSASAKCKSGAYQLSKCFQRELFLGCPASLWTESTDCSAIKARITKCPNAKVPIGHHHKH

>AlucOBP32

MSGRHSLILVLLAAVTSAEVLTDGDCPKTMPKEMKPLYKCCVVEMDSNKTISDDQKAAVDSCVNTSKSDSDANKHDCMIECIFIKLGYMGEDKTINVDYVLKEMNSLLPEDFHEQTSKSLATCMGKKFSSTECPSEIDGVMACFSTMVLMNCPAKHWTDDEECKATRKFFQKCGDSIGYRYD

>AlucOBP33

MHPWKTTCLIGMTAALMVVTAFAGLPFQNEMAVMQCKVKFDVTAEDIQLLKDSKLPSSHSGKCMMACILKKMKVMTKRGQFDLRNVQKWLRNKYQGDQANLAKGNYVAEACANTLPTLGIQDE

CEMAAEIMTCVRTKSKLVKKTLNGELPKEVSP

>AlucOBP34

MEHWKWRLALLIFGMVTCVPQLEGAQKSKQPSKAKTKESQVVAARPKDARAAACVTQIGPDEEEEASFYRKEIPETDKGKCLLACYLESKGVLSGGKFSSSGAAKIAARAYPNNAAKTGNVKHILSHCGTIAARETEQCQLAYRLAECTTTLADKFKL

>AlucOBP35

MINVVFVLLIGTGIVSGGFMEALIECKQQHHVSKEEAMTGESEEVKCFSECVLKKSGMMSDNNEFDEEKIQAEGARMIKNDEQKNREFEGAAKACIEKVNGENPSEKCAKGHALFKCMKEAMPMSKMRG

>AlucOBP36

MKTFVGLIFAVALVEFASAVSKEYHDKAIAAKNTCAKLHNVDDETIMKFWKAHQLPEKEPETCIIICYMKEMKLVVDGKVDADAWKASNKEKWDDEKHVAAADEIVDKCSAEVPPTENECEWGLALTKCALKHGKEAGIPPPDMEHPKRR

>AlucOBP37

MDTHFGLLIASLAILHTANAVINKDYLEKVVTAKDKCLKEFNVDDSVVEDFIVRYNKPQSESGKCMVACYMEERGMMKDGKTITEQVMLDNQEKWIAATHVNMGKEVIDTCDKEVPNEKNDKCDLAVDYMMCLVKRGDEAGLPKMDVAQLKH

>AlucOBP38

MGFKFVKYRSYFFVLVIHIILCIQIKAKELTDEQKEQIFAEIKNCMESTKLTDEEFESIMAKKELPTSKEGKCFTKCLMEKMEYLEEGGKINVIAVQAGLEENMEKESEITKAKEIIQQCADTVPPEDSCEYAYGISQCMYTKMKEAGISGGP

>LlinOBP1

MRILVLFTAALTCVMAGELPEEMREMAQGLHDGCVEETGVDNGLIGPCAKGNFADDQKLKCYFKCVFGNLGVISDEGELDAEAFGSILPDNMQELLPTIRGCAGTTGADPCELAMNFNKCLQKVDPVNFMVI

>LlinOBP2

MVLKMSLLLVVFVASQVLISTTEAYMSQAQMKQAMKTVRNMCIPKSGVAKEALAKMVEGEFDDSDQKLKCYLGCVLGMMQAVKNNKINLTMVRNQISKMLAPEQGQRILTAFEGCATVTGDDN

CDLAFKFAKCIYDTDKELLFQAFIVP

>LlinOBP3

MAVNAKAVLFLALCGLVYVSAYQEVLKATLQDCKGGKEITQEEVDEFVKPLIPKNEEERCLMACVFRAYNVIVDGHFDPKLAYGVAKNILHENPEKLKHIKETLDYCGHEIPTKMDNECDLAGEVMACRNKYNKDHGYDQDP

>LlinOBP4

MSIKIHFFVFAAIGLACVCAYQEQLKQTIKDCQGGKEVTDDELEEFTKPLIPKNEEERCIMACVMRTYNIINNGHYDPKIAFGILKGILKDHPEKLNKIKEVMDHCGEDVPQHMDNECDLAGEIMQCEVKYQKAMGLA

>LlinOBP5

MTTKLRSVGMIVAVTIAYVCAYQEQLKATIQKCQDGREVTDDEVEEFTKPLIPKNEEERCLVACVFKEYKVIIDGHFDPVNALNVAKMVYKEYPEKWERIRDVIDHCGEDIPTHNDNECDLAGDIMKCEVKYLNSMPKITSLELLAGSIAATEEP

>LlinOBP6

MKFVLSAAVVLLVAAAVKANEKKANEKVTEIFNKCKETWPVTDEEIEQVKQKQSIPESKNVKCILACMLKEAKILRDGEYNKENAELMADVLYKDEPEHAXKSKQIIEMCSAELGTKTEGDDCEYAYKMSVCASKHAKELGVKTPEF

>LlinOBP7

VKANEKKANEKVTEIFNKCKETWPVTRRGNXTSENRSRAFPNPKNVKCILACMLKEAKILRDGEYNKENAELMADVLYKDEPEHAEKSKQIIEMCSAELGTKTEGDDCEFAFQGGQLKIMENSFTLRSLLADCLNRRYPGHVGYRYL

>LlinOBP8

MNPLIPVLLVVCAAATRGDEQTNAMVAKAFNKCHGEFPIGDDEMKGVREKSTVPDSHNAKCLMACMLKEGKILRDGKYEKENAIVMADVLNKDDPAAADKAKQLVETCATQVGSDASADECEF

AYKMAVCAAGEAKKLGVRPPDF

>LlinOBP9

MNPLIPVLLVVCAAATRGDEQTNAMVAKAFNKCHGEFPIGDDEMKGVREKSTVPDSHNAKCLMACMLKEGKILRDGKYEKENAIVMADVLNKDDPAAADKAKQLVETCATQVGSDASADECEFAYKMAVCAAAWSSSTRFLKTHPFLLQLCTHRSTWHTFKYEYLPGNTSSRDLTHHINIVCVT

>LlinOBP10

MTPIVAILFALLAAHVKANTKELSPVEVYKHKIHEECIKETKATPEQAKIVFNYKDVPKDDGEKCFMECVYKKSGGIDANGKYSIEGFNKLVDMKYKGEENAGAKMIVKDCSSKVAPKEGEKCSVGRTIRECLSAASKENEFFTI

>LlinOBP11

MHAAFVLIGSALLVAFVSGAPSANVKEIVQNVSKKCVAETKASPEQAKIAVSQHIPKDDVERCYLQCVYTGVGVIKDGKFSEEGGKKLVALRFHDAKEKELANKLIATCAKEIKAKDGEKCSLGRAVRECFVNHGKQVNFFPSA

>LlinOBP12

MVAECPAYSWVCQSTLSYYLHLQQTLQYIDRDPTLAMNQSSCILTLALTIFVMVVVSGFKELDSVLPQAKQEECRKESNFQGELSGDVSQNVTQELKCFAACSLVKLGLMNEKDGTINTTQLDELIAKHTAGKDAADMFKHSVVEPCLKEVNKTADYCEYSFQLVTCGMNKVKPPTTG

>LlinOBP13

MMKIAFVISVLVVLATVSAITPELDKKAKEAVAKCADVPGINEAKKEDCYAACFMTEMGYMTDGKINVENMEEANKQKWDDQQMINKGIEIDKTCAKQVGDTKGKSECAIGYDFGVCKTRLVKANCILRSILQTQLVPSPWWAHLPPLTPLKDSSTFNASFLKGVYREDMEQRNYNKTGLQPPTPLKQ

>LlinOBP14

MKIAFVVSVLVVLATVSAITPELDKKAKEAVAKCADVPEINEAKKEDCYAACFMTEMGYMTDGKINVENMEEANKQKWDDQQMINKGIEIDKTCAKQVGDTKGKSECAIGYDFGVCKTRLVKANCILRSILQXQLVPSPWWAHLPPLTPLKDSSTFNASFLKGVYRGDMEQRNYNKGKNKTGLQPPTPLKQ

>LlinOBP15

MMKIAFVTSVLVVLATVSAITPELDKKAKEAVAKCADVPGINEAKKEDCYAACFMTEMGYMTDGKINVENMEEANRQKWDDQQMINKGIEIDKTCAKQVGDTKGKSECAIGYDFGVCKTRLVKATGLQPPTPLKQ

>LlinOBP16

MKRLVFVLVTLYLLQSASGITDELRKKATEARLKCKQQVGLSDKEYQDWVKGISLPITNGGSCCEVCACWMRELGYMTDGHLNLNNMKNVNTQKWSEKANVEKANQIDTLCTARVVQDGRKECEIALDYRKCKTEMIKQNGGPPKPGST

>LlinOBP17

MKRLVFVLVTLYLLQSASGITDELRKKATETRLKCKQQVGLSDKEYQDWVKGISLPTTNGGSCCEVCACWMRELGYMTDGHLNLNNMKKRQDKYKHS

>LlinOBP18

MRSTGSECFEEVDAKLGNKTSWESDMDPYNCEKVKRMKKRHYCLHECKAKKLGVANEEGVLDFPKVKDLLLSRVNETWQKDILGQAADTCANSKFDQTWKDDTEEYKCNPQAIQFKHCVWKQVEMKCPEEHQNTGRHCKKLRSKISSETSKDSTAKETSV

>LlinOBP19

MKSFVGLIFAVALVEFASAVTKEYHDRAVAAKDKCAKEHNIKESEIQEFVKKHKLPETEDGKCMIACYMEEMKLITDGKVNVDEWKKSNKEKWDEEAHVAMADEIVDKCNEQVSPDGLAKCEYGFKLTECGLKHRLEKGLPAPNMDDVKRR

>LlinOBP20

MKLVKDDKARPPKPEGYECIDDCIMAKNGFLGTDKKIDAAKVNAAAKTSYTGEWAEPGAKMVEKCLAQVSANKEKGECTSGADIFSICMFRESFINCPEKSWTSSETCKANKERLIKCPKSIPFLNKSAK

>LlinOBP21

MLTAYMIVATLSVFFFAVALTQGQMDEDPDCRPPHPPGKEAQCCPLPDFVGVVDNFHDVMHKCSDEAGLRKPSGPPGSGTPPTAEEMAAHMSAHECADECLFKNTKYLQSNGELDKDAIKASVTKIFTGDWAALASSAADKCLASAKSEVGASAKCKSGARQMVKCFTRAMFLNCPASSWTESTECAAAKARITKCPNAMVPMIPPYPQPISANSTILDSLFACITDGEYMEFQSISSGRLG

>LlinOBP22

MPSQLPCPFFLLNIAFAHPGHFDEDPECRQPHHHRHEENDCCKVPSLFSNNKDEMHELVHKCFEEAGIKKHGPHHEHHGPPPLEDGPIPPPPPPPFSPKNDSKLDCVEQCFLKNLDLVDDEGDLKVDDLKALVTEKFSGDWASVGSSAIEKCLEKAKTEENESSKCKAGSKRVLHCHCTQHASYPKMGGSQSARKVTLDNPNPDPHDLNNVI

>LlinOBP23

MYAFTAALSFFLLNIAFAHPGHFDEDPECRQPHHHRHEENDCCKVPSLFSNNKDEMHELVHKCFEEAGIKKHGPHHEHHGPPPLEDGPIPPPPPPPFSPKNDSKFDCVEQCFLKNLDLVDDEGDLKVDDLKALVTEKFSGDWASVGSSAIEKCLEKAKTEENEPSKCKAGSKRVLHCLAREFFMNCPASDWTESEVCLAAKDRVSKCPHSLPPMHH

>LlinOBP24

MADSVGECMKLIKVKPEKGPPVPEGFDCMDTCVFSKLGFIGADNKLDPEKLAKKFSELFKGDWSALSESTLKKCLPMADVGKGVCSSGADVFKFCLIRELYMNCPASSWTKSDLCKANVERLEKCPNSLPFMNGSGLKNKSSR

>LlinOBP25

MFTTATSTIIFLFAVALTRGQMDEDPECRPPHPPGKAGDCCVQPKLFDEGDMPDVIKKCHEEAGVKRPSGPPGSGTPPTAEEMAAHKSAHECAAECIFKNNNFIKSDGELDXDAIKATVTKMFTGDWATLASTTIDKCLASAKSEVDASPKCKSGADQVVRCFGRSLFIGCPASAWTESTECAAEKARLTKCPNAMPPPPHHKH

>LlinOBP26

MNQSSCILTLALTIVAMAVVSGFKELDSVLPQAKQEECRKESNFQGELSGDLSQNVTQELKCFAACSLVKLGLMNEKDGTINTTQLDELIAKHTEGKDAADMFKHSVVEPCLKEVNKTADYCEYSFQLVTCGMNKVKPPTTG

>LlinOBP27

KFYRRFKHHQIAAHLTFCVYVKTGSRDKTETPRSERPLCKAPTSAPRKLEKVINQCQEEIKYALLQEALSVLGETVSLRTALTRNRSKRETFTGEERRIAGCLLQCVYRKMKALDETGFPTATGLVKIYSEGVEDRNYYLATIQGVQQCLSRELQNRNKNPSIVKAEGYSCDVAYDVFNCVSEEIEQLCGTSP

>LlinOBP28

MEVAACLVLLAALAALTSAVDEKRPLCKAPTSAPRKLEKVINQCQEEIKYALLQEALSVLGETVSLRTALTRNRSKRETFTGEERRIAGCLLQCVYRKMKALDETGFPTATGLVKIYSEGVEDRNYYLATIQGVQQCLSRELQNRNKNPSIVKAEGYSCDVAYDVFNCVSEEIEQLCGTSP

>LlinOBP29

MNRPLLLLTAVLAVGSGQQEDCKTAPAGWPKRPPQCCDLPFPLEGMKREFGSCIRQIGNRQSSAVPTAQAVRDARLCIEECVYKGLGFMEEHNLNKDQLLEQLKKGVAGKKDWEKPMEDAVKSCHETITKRETPQEGACQDSAHEFTHCVMRQLFLSCPASEWNNNDECNLVKNRMQACPNIPPPPPPPPQGFRGQGPPQPQ

>LlinOBP30

MNSFTVLCLVASVVALTQGNPTTPNPTSSSHAASVSGGSTVSGVSKSPEEVKQKIKEQVEALTGACKSQTKITGEQAKIVATQAIPKTEAEKCFLECIYTGLQLTKDGKFNEPAARALAQKRFGNAPEDLTKANSMIDTCVKEVVVKDLNEKCALGRLIRECFVKNGAKINFFPKP

>LlinOBP31

MLTAYMIVATLSVFFFAVALTQGQMDEDPDCRPPHPPGKEAQCCPLPDFVGVVDNFHDVMHKCSDEAGLRKPSGPPGSGTPPTAEEMAAHMSAHECADECLFKNTKYLQSNGELDKDAIKASVTKIFTGDWAALASSAADKCLASAKSEVGASAKCKSGARQMVKCFTRAMFLNCPASSWTESTECAAAKARITKCPNAMVPMIPPKH

>LlinOBP32

MEVNAERNVTDEQRXAVRLCSRYTEVESGLAEAGYDCLAECFFIKLGLMGEDKTLNKENILEEVRIQFHEDXVEPARKALETCMEKKYNTKCPSGIDGTMQCFTVQLMLNCPXQNWTDGEECKETRTFMEKCGETLNYYD

>LlinOBP33

LEKVINQCQEEIKYALLQEALSVLGETVSLRTALTRNRSKRETFTGEERRIAGFEFRCDLXDGWKWSQEERGGSSPTWFEINTIPGMNTTSRSWRLSSQISSSSVFCNVYTGK

>AlinOBP1

MNSLIPVLLVVCAAATRADEQTNAMVAKAFNKCREEFPISDDEIGGVREKTTIPESHNAKCLMACMLREGKMLRDGKYEKENALIMADVLNKDDPASADKAKQLVETCAGKVGTDAGGDECEFAYKMAVCAAEEAKKLGVRPPDF

>AlinOBP2

MSLKIQFFVFAAICAACVCAYQEQLKQTIRDCQDGKEVTDDELEEFTKPLIPRNREEKCIMACVMRTYNIISNGHYDPKIAFGILKGILKDHPEKLNKIKEVMDHCGEDVPSHMDDECDLAGEIMQCEVKYQKAMGMA

>AlinOBP3

MDIRFGFIIACLAILSVANAISKEYSARMIAAKEKCQKEFNVTDSVVEDFMKRNIKPESKSGKCMVHCIMEEMGMIDDHKINTEQVKLGNKEKWDDPALVELANQVADTCDQEVFTEGRCKCLVAVEYMMCLATHGDEVGLPHVDFEDSQDS

>AlinOBP4

MRIFVIFTAALTCVMAGELPEEMKEMAQGLHDSCVEETGVDNGLIAPCAKGNFADDAKLRCYFKCVFGNLGVISDEGELDAEAFGSILPDSMQELLPTIKSCGGTTGSDPCDLAMNFNKCLQKADPVNFLVI

>AlinOBP5

MVLKMNLLLVVLVMSQVFFSVTEAAMSQAQMKQAMKTVRNMCIPKSGVDKEALAKMVNGEFDESDQKLKCYLGCVLGMMQAVKNNKINLTMVRNQITKMLAPERGQRILAAFESCATVTGDDNCGLAFRFAKCIYDTDKEAFIVP

>AlinOBP6

MGFKFVKYRSYFFVLVIRIILCIQIKAKELTDEQKEQIFAEIKNCMESTKLTDEEFESIMAKKELPTSIEGKCFTKCLMEKMEYLEEGGKINVIAVQAGMEENMEKESEITKAKEVIQQCADSVPPEDSCEYAYGISQCMYNKMKEAGISGS

>AlinOBP7

MNRPLLLLTAVLTVGSGQQEDCKTAPAGWPRRPPQCCDLPFPLEGMKKEFGSCIRQIGNRQSSAVPTAQAVRDARLCIEECVYKGLGFMDEHKLNKDQLLEQLKKGIADKKDWTKPMEGAVKRCHETITKRETPQEAACQDSAHEFTHCAMRELFLNCPASEWNNNDECNLVKSRMQACPNIPPPPPPPPQGFRGQGPPPQ

>AlinOBP8

MDTHFGLLIASLAILHTANAVINKDYLEKVVTAKDKCLKEFNVDDSVVEDFIVKYNKPQSESGKCMVACFMEERGMMKDGKTITEQVMLDNQEKWIAATHVNMGKEVIDTCDKEVPNEENDKCDLAVDYMMCLVKRGDEAGLPKMDVAQLKH

>AlinOBP9

MMELWKWRLALIIFGLVSCIQQTEGSQRTKQQPKSKTKESVVGATRPRDAKATECVNKVNANEEESASFFRKEIPETEAGKCLLACYLEGKGLIVGGKISSSGAARVAARAYPNNRVKTGNVKHILSHCGTIAGRESNNCEMAYKLADCTTTLSDKFRL

>AlinOBP10

MFFNSVFLLVVCVSSYVTKGQELPPPGDVKNKTVVFKNSFLRSAKYCSSIYETSTLAIMALLMSEKSDDQNGKCFLNCMLQRYRLMSQDGSYNKDKFKPFLEYIPDSKFLQSIRGNLKNCISEKDPDPCEKASKFIKCFYTRARNKGEIGASKEVIPADGF

>AlinOBP11

MKTFVGLIFAVALVEFASAISKEYHDKAIEAKNTCAKLHNVDDETIMTYWKNHQLPEKEPETCIVICYLKEMKLVVDGKVDADAWKASNKEKWDDEKHVAAADEIVDKCSAEVPPTENECEWGLALTKCALKHGKEAGIPPPDMEHPKRR

>AlinOBP12

MTTKLRSIGLVFIVSISYAFAYQELLKETIKKCQNGRDVTDDEVEEFTKPLVPKNEEERCLVACVFKEYKVIIDGHFDPVNALNVAKVVYKDYPDKVERIKDVLDHCGEDIPTHNDNECDLAGDIMKCEVKYLNSVPKMTSLEFLAGSMAATAEP

>AlinOBP13

MNISTRMISLTMAYLAAALVSGHRALDGILPQANQDECREESNFRGELNDDVGRNVTQELKCFAACSLMKLGIMNEKDGTVNMTRLDELIASHTPGKDAADVFKTTVVEPCMKEVKKSTDYCEYSYQLIACGMSKVP

>AlinOBP14

MKPPGPPGSGTPPTAEERAARKIAHECADECLYKSSNLLTSAGELDKDAIKALVTKLYTGDWATAATTAIDKCLASAKGEVEATSKCKSGSFQLSRCFMRSMFLGCPASSWTESTECAAAKARLTKCPNAMAPMPHKK

>AlinOBP15

MFAITAALSLLLLNVAIAHPGHFDEDPECRPPHHHRLEEKDCCKIPNLFSKSKDEMHELVHKCFEEAGIKKHGHHDHHGPPPPPGLGLPPPPPPPPKNDSKFDCVEQCFLKNLELINEEGEVKVDELKALIAEKFTGDWASVGSSAIEKCLEKSKTEENDSTKCKAGSKRILICLARESFLSCPASEWTESDVCTAAKDRLEKCPHAPPPMNH

>AlinOBP16

MKFFASAALVLLVAAAVKANEKKANEKVTEIFNKCKETWPVTDEEIEQVKQKNSIPESKNVKCILACMLKEAKVLKDGEYNKDNAELMADVLYKDEPEHAEKSKQIIELCSAELGTKTDGDDCEYAYKMSVCAAKHAKELGVKTPEF

>AlinOBP17

MQAAFVLLGAALLVAVVSGAPPSVKEIVQNVSKKCAAETKASPEQAKIILTQNIPKNDVERCYLQCVYSGVGVIKDGKFSQEGGNKLVAMRFHDAKEKELAKQLINTCAKEIKAKDGEKCSLGKGIRQCFVAHGKEVNFFPHA

>AlinOBP19

MNTLLLCAVIAVSACFAYDFSDPEFNIIIDDELFDIAEGKDTLLRNRRDIDDDDERMAFEQDEADDPSNGPPDHLSEMNDENSHHKGHCKRHHKSCCGKTPLPLSLIQHGKNETKRSTGSECYEEIDAKMGNKTSLENDMDPYNCEKVKRMKKKQYCMHECKAKKMGVANEQGVLDFPKVKDLLLSRVNETWQKEVLGQAVDTCATSKFDQTWKDDQDEYKCNPQALQFKHCVWKQVELKCPEEYQNTGRHCKKLRTKLTSETNKETATKETTI

>AlinOBP20

MYTFNTFFVLTVASYVIAAPPADVPAECLVPKGKEEEVSKCCKLDAISTKEVAAAKECMKLVKDKDAESKGLLPKPEGFDCFDDCVMTKMGFMGADKKIDAAKVNAGAKTSYTGDWAEPGAKMVAKCLAQVSAMKEKEECASGADIYETCIFRESYMNCPEKSWTNSDACKANKERLTKCPKALPYLEKLDLS

>AlinOBP21

MNPTVAIISIFFVAYTQAHTKELHATEVYKLKVRDECNKDIKATPEQSEIVSKFKEVPKDETEKCLLECIYIKTGGIDADGKYSVEGFNKLIDMKYKGDENTNAKKINTDCAKKAVSKEGEKCSLGGSIRECFAAAAKENDFFTI

>AlinOBP22

MATNAKAVLFLALCGIVYVSAYQEVLRATISDCKGGKEVSQEELDEFIKPLIPQTREEKCLMACVFTAYNVIVEGHFDPKLAYGVAKNILHENPEKLKHIKETLDYCGHEIPTKMDDECELASEVMACRNKYNKDHGYDQDP

>AlinOBP23

MNTFAALVLVASVVALSQGNPTTGAPSTTLVSDQPTGSTASGVSKSPEEIKQKIKEQVGALTEACKSQSKITGEQAKIVATQAIPKTEAEKCFLECIYTGLQLTKDGKFNEPAARALAQKRFGNAPDDLQKANSMITACVKEVVVKDTNEKCALGRLIRECFVKNGAKINFFPKP

>AlinOBP24

MPTFSTLSTFLFALALTYGQIVEDPECRPPHPPGREDSCCILPELFIGGIQDVIRKCHDEAGLKKPSGPHGGGSPPSAEEMAAHWRAHECADECVLKSQNLLTTDGELNKDAIKAQVVKSFTGDWAKLASDIADKCLASAKGEVTSTATCKSGAGQFIYCFRRNLFLQCPSSSWTETTDCAAAKARITKCPNAKIPMGHHRH

>AlinOBP25

MDVMILPVFLIVFIAATASPAVLSAECPQRFPKEMRPLSSCCKVEMNPNSSYNSTAGEAIIDKCFGGFNSSATRPTGPPSGYDCEMECLMIEFGFMGKDKTINKDKIVTSIQDEYSADFQEAANKAIEICMGRKYQTSCPSGIDGMMECFAVQMMLNCPAKHWSGGEDCKETKQLIEKCGEVLSIFADYDTE

>AlinOBP26

MKSLWKCCKTVQSSKATHPTSEQMVEIKSCYSNWNHSVETNAAPEGFDCVEECVYSKLGFMGTDKTINKEKLLQFQKEETHEDFHEAITKSMDMCMGKTFTTKCPSGIDAVIKCEAIQIYLNCPAKHWDNGDDCQETKKLMEKCADVTSMYN

>AlinOBP27

MTQWKTASFFVAMAALVVIAFAGLPFQNEMAVMQCKTKFDVTSEDIQLLKDRKLPASHSGKCMMACILKKMKVMTKRGQFDLRNVQKWLRNKYQGDQANLAKGTYVAEACANILPTLGIQDECEMAAEIMSCVRNKSKLIKKTADGQLPSSTGI

>AlinOBP28

MSSDMANILLLLVIGAGIAVNEGDASMEALMECKKDYKVSREQIMSGDSSEEVKCFAECLMKKTGGMDEGGNFNTEKIKEEGRKHAKTDDQKRANDAAVDKCISETEAANPTGKCEKGFEFFKCVRGEMKSPM

>AlinOBP29

MYQLTCPVFVIVMYWVNQGSADVGLLDYSAQCVTEAGVTHDEADKIQQGNLPSNQQGKCYVACVLKSLGLVDRRGKISAENTNRLIDMYSNEPGDAKDKTKQAVNTCATEANRAWTWSQCEVAYRMMSCILRTRGSLQQTRSITITLPQSITINPPPLSFTLFSVG

>AlinOBP30

MQLKNTSDSLVGHLFSIALPNTLLNPSQLLPELAITSRQSPDIVSIACFLHAIGSNFLFASKTPSEANRQSWMHSNSLESVPLVSFESQVVIVLLVKGNT

>AlinOBP31

MAVPAVLIVFFAVTASSAVLSDECPKTFPKEMQPMWRCCELKTKPDARLFTYEHQKSCRDYFGSTTNSSKTRPTDKCEEECMMMAAGFMGEDKTIYYEKFENQLKNLFVEEFDEAVGKTMKACTGIKPNGRCSSGMDIFLICMEVQMNRNCPANLWRSSDACKKTKELIEKCGEMEGIV

>AlinOBP32

MEERGMMKDGKTITDQVILNNQEKWIAPNHQALGKQVIDTCEKEVSPEKSTKCDIAVDYVMCLVKRG

>AlinOBP33

MNCPDSSWTKSDLCKANVARLEKCPMSMPFMPVLGLKKN

>AlinOBP34

MISIKLVTLISLVACCCSAFPEESAECKISEKDNDELFKCCKLNVVLDEMAESIGECLKLVKEKSEKGPPVPEGYECMESCVFAKLGFIEGNKLDEEKLTKAFAEL

>AlinOBP35

LKPQGIDLNCPIYDTLQCIFSSLIKHATPSQWSTTSECQGYRAFAAACPICPEDCFAAQVPIGSCNACLTLPRSA

>AsutOBP1

MNSLIPVLLVVCAAATRADEQTNAMVAKAFNKCREEFPISDDEIGGVREKTTIPESHNAKCLMACMLREGKMLRDGKYEKENALIMADVLNKDDPASADKAKQLVETCAGKVGTDAGGDECEFAYKMAVCAAEEAKKLGVRPPDF

>AsutOBP2

MSLKIQFFVFAAICAACVCAYQEQLKQTIRDCQDGKEVTDDELEEFTKPLIPKNREEKCIMACVMRTYNIISNGHYDPKIAFGILKGILKDHPEKLNKIKEVMDHCGEDVPSHMDDECDLAGEIMQCEVKYQKAMGMA

>AsutOBP3

MATNAKAVLFLALCGIVYVSAYQEVLKATISDCKGGKEVSQEELDEFIKPLIPQTREEKCLMACVFTAYNVIVEGHFDPKLAYGVAKNILHENPEKLKHIKETLDYCGHEIPTKMDDECELASEVMACRNKYNKDHGYDQDP

>AsutOBP4

MRIFVIFTAALTCVMAGELPEEMKEMAQGLHDSCVEETGVDNGLIAPCAKGNFADDAKLRCYFKCVFGNLGVISDEGELDAEAFGSILPDSMQELLPTIKSCGGTTGSDPCDLAMNFNKCLQKADPVNFMVI

>AsutOBP5

MGHIPMSSDMTNILLLLVIGAGIAVNEGDASMEALMECKKDFKVSREQIMSGDSSEEVKCFAECLMKKTGGMDEGGNFNTEKIKEEGRKHAKTDDQKRAHDAAVDKCISETEAANPTGKCEKGFEFFKCVRGEMKSLM

>AsutOBP6

MGFKFVKYRSYFFVLVIHIILCIQIKAKELTDEQKEQIFAEIKNCMESTKLTDEEFESIMAKKELPTSKEGKCFTKCLMEKMEYLEEGGKINVIAVQAGLEENMEKESEITKAKEIIQQCADTVPPEDSCEYAYGISQCMYTKMKEAGISGGP

>AsutOBP7

MNRPLLLLTAVLTVGSGQQEDCKTAPAGWPRRPPQCCDLPFPLEGMKKEFGSCIRQIGNRQSSAVPTAQAVRDARLCIEECVYKGLGFMDEHKLNKDQLLEQLKKGIADKKDWTKPMEGAVKKCHETITKRETPQEAACQDSAHEFTHCVMRELFLNCPASEWNNNDECNLVKSRMQACPNIPPPPPPPPQGFRGQGPPPQ

>AsutOBP8

MKLALVTAFLSAIVLAEGNINKEYLDKLIAAKEKCVKEFSVDDSIVEDLYVRYNKPPTESGKCMVACYMEERGMMKDGKTITEQVMLDNQEKWIAATHVNMGKEVIDTCDKEVPNEENDKCDLAVDYMMCLVKRGDEAGLPKMDVAQLKH

>AsutOBP9

MMELWKWRLALIIFGLVSCIQQTEGSQRTKQQPKSKTKENVVGATRPRDAKATECVNQVKANEEESASFFRKEIPETEAGKCLLACYLEGKGLIVGGKISSSGAARLAARAYPNNRVKTGNVKHILSHCGTIAGRESNNCEMAYKLADCTTTLSDKFKL

>AsutOBP10

MFFNSVFLLVVCVSSYVTKGQELPPPGDVKNKTVVFKNSFLRSAKYCSSIYETSTLAIMALLMSEKSDDQNGKCFLNCMLQRYRLMSQDGSYNKDKFKPFLEYIPDSKFLQSIRGNLKNCISEKDPDPCEKASKFVKCFYTRARNKGEIGASKEVIPADGF

>AsutOBP11

MKTFVGLIFAVALVEFASAVSKEYHDKAIAAKNTCAKLHNVDDETIMKFWKAHQLPEKEPETCIIICYMKEMKLVVDGKVDADAWKASNKEKWDDEKHVAAADEIVDKCSAEVPPTENECEWGLALTKCALKHGKEAGIPPPDMEHPKRR

>AsutOBP12

MFQAFVYQELLKETIKKCQNGRDVTDDEVEEFTKPLVPKNEEERCLVACVFKEYKVIIDGHFDPVNALNVAKVVYKDYPDKVERIKDVLDHCGEDIPSHNDNECDLAGDIMKCEVKYLNSIPKMTSLEFLAGSMAATAEP

>AsutOBP13

MGDVSNTYGSYDSYGASYGGYIRGSTPSGSGRGYKQSYEGDIGSGTSYARGAGYGGAASYGTGYYNGNYGPDRTIYNPDSRTRGSYGGFMRGGGSSSNDGIFGATDFGSYAKQIDTTSYDSSESYDRVESYGGIPSGTPRGIPYNGYHNNANIRWPDSNSQGNARKNGSSLEDVEPCTILCIFRQMKMTNGDSYLEQQSVAAVLMRRARDPQLKDFIGRTVQMCFERFGLANKGRCESAKLFALCMEEAGKMNCEDWDVNKRFALKNPKPGPVLTMPQPLPPPPPRG

>AsutOBP23

MFAITAALSLLLLNVAIAHPGHFDEDPECRPPHHHRLEEKDCCKIPNLFSNSRDEMHELVHKCFEEAGIKKHGHHDHHGPPPPPGLGLPPPPPPPPKNDSKFDCVEQCFLKNLELINEEGEVKVDELKALIAEKFTGDWASVGSSTIEKCLEKSKTEENDSSKCKAGSKRILICLARESFLSCPASEWTESDVCTAAKDRLEKCPHAPPPMNN

>AsutOBP31

MGRLTKTQNADLLIHPAGKIRVGVLPELFVGGIQDVIPCHDEARLKKPSGRHGGGGSPPSAEDMAAHLRAHECADECVLKSQNLLTTDGELNKDAIKAQVVKSFTGDWAKLASDTADKCLASAKGEVTATATCKSGAGQFIYCFRRNLFLQCPSSSWTETTDCAAAKARITKCPNAKIPMGHHRH

>AsutOBP32

MDVMILPVFLIVFIAATASPAVLSAECPQRFPKEMRPLSSCCTVEMNPNSSYNYTEGDPIINKCFGDFNSSATRPTGPPSGYDCELECLMIEFGYMGKDKTINKDKIVKSIEDEYSADFQEAGHKAIEICMGRKYHTSCPSGIDGMIECFAVQMMLNCPAKHWSGGEDCKETKQLIDKCGEVLSIFADYDTD

>NlugOBP1

MKSFIVCIAVSYLLVANIKADEATSSSDAESLITSTTLSPASNESDAARSAIKEQLAKLTESCKTSSQANSDDAKIIGTESVPKTEGEKCFLQCVYTGFGIVKNDQFSVEGARLLAQKRFGAFPEELEKANQLIETCSKEAVKKDSKDKCPMGFLIRQCFVKNGQKINFFPKA

>NlugOBP2

MKCQIVLAALALATICEVSYAGLTPDKLKELKPLIDTCIKQSKVEEDTLGKLHNGHEIPSSQSGKCFIACMAEHMKLMKDGKFEPEMTMEFIDKMVQDKDKAAEIKKSLGECIKSVPEGDKCEMAAGLATCMKDHHAELAGMN

>NlugOBP3

MKASAAITLVFLSLAVFHCSEAKLDKAKKEAAIKKCQAETQATDEDVMKVRKEHIVPDSEEGKCFIACGFNSYDMLKDNRINLEGVNAFFEKLYDEQDKRDIAIKAAASCAATETVSGLNECHYAAKYFACMQRHPDFAKMKDDFDI

>NlugOBP4

ANFLMMQQSMQGTQMPMIQSIASELKFCMDVNAEQNSDGLNDYLPLLFNEELPSTLGQKCFLTCLFNRFGLLKDGFLDTKTAKNLVETFYADKHDEKTMANIAINVCHVAAVPDALNPCEIGFSLKSCFVDSNKKGKELRGKN

>NlugOBP5

VFIDGRNEYRLTRQAPPDDECRPPRPGPNEDGVCCDMPPVFRTAHDKFESCLEELSSIFPPPPPPPHGHHGPPPPPPGARGPPPPPPPGGRRGPPPGFGGPPGHEPPIFACAHECLFNKTGMLENGKLNVEALKKKLEDELGENEVWKNLVQSIVDKCMESKDAPSNEMCTSGSHELARCVLRDMFMNCPQEKWKESDDCSNMKMKLEKCPELVPPMAMRLPHPPMP

>NlugOBP6

EDTTIKIKTPSPHKHQQVYCQAPPTAPERLERIIEQCQDDIKTALQEALNVLTDTSPRDLVKKTRSKREVFSGEEKRIAGCLLQCVYRKVKAVDDQGMPTVPGLVRLYSEGVQDRNYYVATVQAVQQCVSASQHFRYYNPQVLKEDGYTCDLAYDMFNCVSDKIEAFCGRTP

>NlugOBP7

RFTEEEKQLMNQVHSQCISETGTSEDLVTKATTGDFADDDNLKCYVKCIWSTLTVMDDEGNFDVGVLEVMLPADMKDTVMKAMNACTGVGGATPCEKAFAMTKCLYKEAPSDFFLP

>NlugOBP8

YDFSDPYFNEHLQSAMEEIMEEEMLSIGRVQRDADQGQEVADEYFKCKHRNLKTCCGKINLMKNYGDKGKIYGKQCYEEVVSAFKTNSSSTADDDDSMMDMFSCEKVKMIKLKHICVHECIGKKTKILKEDGTLNPEEIKQYAREYMFNEEWSKELGEKALDKCLSQTYNSVTKMLDEYEIKCNPSSVQFHHCLWKEIELTCPESKVDLKAKCVRLRERLRKQQAAGM

>NlugOBP9

MPPVFRTAHDKFESCLEELSSIFPPPPPPPHGHHGPPPPPPGARGPPPPPPPGGRRGPPPGFGGPPGHEPPIFACAHECLFNKTGMLENGKLNVEALKKKLEDELGENEVWKNLVQSIVDKCMESKDAPSNEMCTSGSHELARCVLRDMFMNCPQEKWKESDDCSNMKMKLEKCPELVPPMAMRLPHPPMP

>NlugOBP10

MLSESMPVIARVERDASSTTPGFPFKTNQRVALKRAKRSTIFPIDGIVERVAIFPVHEKTKRVVRESSEEDDSCKKMKPEHGAKMCCELPSVFRGSPEIFKACREELGLPDHKSPPPAPSAEGNGKPPHHGGPPHHRGKGCIAECLFNKTGLLEGGKLNKEALQKSLDEHLKTDDAWKAVATSTLDKCYDDVQTKDFKPDNEKFTSGSSEFMRCFSRGLFMDCIPSKWTDSDECSKTKETLEKCPMMLPPG

>LstrOBP1

MAADSSDMLTVFNKCRDETSATEDDIKTFRAQQIPSTTTGKCMLACMFNHSGLMKDGKYDSEGALKLVGQVFADNPIKLGKARQLINGCTDEVKNENDECEIASKIADCTVKMSSQVGLS

>LstrOBP2

MANMCISSSGVQRSLITKAMKGEIEDDRKLKCFFGCIMEAVQVTKNGRMQPEVLKRRANAMLPKTMREMILPTVDSCSHIENEDKCELAYSIVKCHFSVNGKNPFFFNF

>LstrOBP3

MAADSSDMLTVFNKCRDETSATEDDIKTFRAQQIPSTTTGKCMLACMFNHSGLMKDGKYDSEGALKLVGRVFADNPIKLGKARQLINGCTDEVKNENDKCEIASKIADCTVKMSTQVGLS

>LstrOBP4

MMNQVHAQCVTETGTAEDIVNKATKGDFTEDDNLKCYVKCIWSTLTVMDDEGDFDVGVLEVMLPADMKDIVMKAMSACTGAGAGLSPCEKAFAVTKCLYKEAPA

>LstrOBP5

MMQQSLQGTNIPMIQSIAGELKFCMDVNSEQNSDGLDDYLPLLFNEELPTTLGQKCFLTCLFNRFGLLKDGFLDTQTAKTLVETFYKDKHDEKTMANIATNVCHVSAVPDVLNPCEIGFSLKSCFVDSNKKGKELRHKN

>LstrOBP6

ADSSDMLTVFNKCRDKTSATEDDIKTFRAQQIPSTTTGKCMLACMFNHSGLMKDGKYDSEGALKLVGQVFADNPIKLGKARQLINGCTDEVKKENDKCEIASKIADCTVKMSSQVGLS

>LstrOBP7

KIDKAKKEAAIKKCQAETSASDEDVKKVRKEHVVPESEEGKCFIACGFNTYDMLKDNRINLEGVNAFFEKLYDEQEKRDIAIKAAASCAATESISGLNECHVAAKYFACLQRHPDFVKMKEDFDV

>LstrOBP8

YDFSDPYFNEHLQSAMEEIMEEEMLSIGRVQRDADQGQEVADEYFKCKHRNLKTCCGKINLMKNYGDKGKIYGKQCYEEVVSAFKSNSSSTADDDDSMMDMFSCEKVKMIKLKHICVHECIGKKTKILKEDGSLNAEEIKQYAREYMFNEEWSKELGEKALDKCLTQTYNSVTKMLDEYEIKCNPTSVQFHHCLWKEIEMTCPESKVDLKAKCVRLRERLRKQQAAGM

>LstrOBP9

EDTTIKIKNPSPHKQQQVYCQAPPTAPERLERIIEQCQDDIKTALLQEALNVLTDTSPRDLVKKTRSKREVFSGEEKRIAGCLLQCVYRKVKAVDDQGMPTVPGLVRLYSEGVQDRNYYVATVQAVQQCVSASQHFRYYNPQVLKEDGYTCDLAYDMFNCVSDKIEAFCGRTP

>PsalOBP2

MTEVHVALKQKLNTIAVKCKDELHAPQEIMALASNTVVPQNEQQRCYLECVYKNLNLIKNDKFSVDDGKTMAKIRFAKQPEEYKKAVTIIETCEKEAVIDPKTTEKCAAGRVIRNCFVKNGEKINFFPKA

>PsalOBP1

MNSSAAVENCLLETNMTRDEFEDMLTSPNARELTILKSHAHKCMFGCVMRKNHIVNDGVVSKEVLSKYVLNFYGRPDYKRRLIIKDVEHIVDVCAKKVADESETDECELAATLVTCIVLEANKAGLVDDPARQI

>PsalOBP4

QKQETSGKCRAPDKAPLNLEIIINTCQEEIKSALLQEALDILNDGNTEQNTQNQSNRSKRETEEDLTNEERRVAGCLLQCVYKKVKAVDETGFPVVDGLMKLYNEGVQDRNYYIATLSAVRHCISIAQQLKQQQPSKTFDDGQTCDLAYEMFECVSEKIEENCGVENKSNN

>PsalOBP9

MAKLFGVALKCFKDADWGACGEMITTKYDITEPKYKQCTCQMACVGEDLGMINTKGEPEPAKFLEYVKRINNQSIKSQLQHIYDKCQNVKGADKCDLSEQFAICAFKESPALKERVSTLMEMLVKMKPKSK

>PsalOBP10

STRPQPDELEEVKKSLYNACSSKFPLTEEIRNQAKNGILTEDPNLKCFLRCCFDEMSLIDEDGIIDGETLVAMSMDRIKLITEQAVHNCLKTTKQDGCEASFQFLSCGIKLNPLI

>AfabOBP8

ENNQQNSNDRSATIFQSCISETKLSGDALKGFRSMSIPKTQAEKCMMGCLMRKVNVINKGKFSVEEATKVAQKYYGTNETMMKKAKDLIDVCAKKAQSTTEECALAGIVTTCIVEEGQKVGLTGGPGGRSRRTVSPKFRRNSM

>AfabOBP2

SDPCNISTCYQSGTTKPPTTVTPTRLPVQSSSTPTSHQQTTYAKDHVHSSTATKSGVNTTATTTSGASVNGTERTTVVKSSSGVAGNLTTPKPTMTEGHVALKQKLNTIAVKCKDELHAPQEIMALVSNTVVPQNEQQRCYLECVYKNLNLNKNNKFSVDDGKAMAKIRFANQPEEHKKAVTIIETCEKEAIIDPKTTEKCAAGRVIRNCFVKNGEKINFFPKA

>AcraOBP2

SDPCNISTCYKSGTTKPPTTVTPTRLPVQSSSTPTSHQQTTYAKDHVHSSTATKSGVNTTATTTSGASVNGTERTTVVKSSSGVAGNATTPKPTMTEGHVALKQKLNTIAVKCKDELHAPQEIMALVSNTVVPQNEQQRCYLECVYKNLNLIKNNKFSVDDGKAMAKIRFANQPEEHKKAVTIIETCEKEAIIDPKTTEKCAAGRVIRNCFVKNGEKINFFPKA

>TsalOBP1

MNSSAAVENCLLETNMTRDEFEDMLTSPNARELTILKSHAHKCMLGCVMRKNHIVNDGVVSKEVLSKYVLNFYGRPDYKRRLIIKDVEHIVDVCAKKVADESETDECELAATLVTCIVLEANKAGLVDDPARQI

>MvicOBP1

MTRDEFEDMLTSPNARELTILKSHAHKCMFGCVMRKNHIVNDGVVSKEVLSKYVLNFYGRPDYKRRLIIKDVEHIVDVCAKKVADESETDECELAATLVTCIVLEANKAGLVDDPARQI

>MvicOBP2

MSVTPTRLPVQSSSTPTSHPQTTYAKDHSHGSTTTKSGANATATTASGASVNGTERPAVVKSSAGVTGNLTTPKPTMTEGHVALKQKLNTIAVKCKDELHAPQEIMALVSNTVVPQNEQQRCYLECVYKNLNLIKNNKFSVEDGKAMAKIRFANQPDEHKKAVTIIETCEKEAVIDPKTTEKCAAGRVIRNCFVKNGEKINFFPKA

>MvicOBP3

MASMRFTTEQIDYYGKACNASEDDLVVVKSYKVPSSETGKCLMKCMITKLGLLNDDGSYNKTGMEAGLKKYWSEWSTEKIESINNKCYEEALLVSKEVIATCNYSYTVMACLNKQLDLDKST

>MvicOBP5

MKRFGDKDKVAADECYAQVAEKFATVTATTPKQDLFSADAVKITKKKQFCLHECIGKKNHLLTEDGSLNKTFIADYAMKSVFKEQWQKPVGLKALEKCLEETYIPWPAEDKENVCNPVYVQFQHCLWLQYESNCPANKIKITKKCEKTRNRYRMQKSTSN

>MvicOBP8

MSIPKTQAEKCMMGCLMRKVNVINKGKFSVEEATKVAQKYYGTNETMMKKAKDLIDVCAKKAQSTTEECALAGIVTTCIVEEAQKAGLSGGPGSRSRRTVSPKFRRNSM

>MvicOBP10

STRPQPDELEEIKKTLYNACAGKFPITEEVKNNAKNSIFLDDQNFKCFLKCCLDEMSLIDDDGIIDGDSLKAMASDKIKPMXEQVVPNCLKNVQQDGLEAAFVFLRXGRG

>MdirOBP1

MTRDEFEDMLTSPNARELTILKSHAHKCMFGCVMRKNHIVNDGVVSKEVLSKYVLNFYGRPDYKRRLIIKDVEHIVDVCAKKVADESETDECELAATLVTCIVLEANKAGLVDDPARQI

>MdirOBP2

MAVTPTRLPVQSSSTPTSHPQTTYAKDHVHGSTTIKSGANATATTASGASVNGTERPTVVKSSAGVIGNSTTPKPTMTEGHVALKQKLNTIAVKCKDELHAPQEIMALVSNTVVPQNEQQRCYLECVYKNLNLIKNNKFSVEDGKAMARIRFANQPEEHKKAVTIIETCEKEAIIDPKTTEKCAAGRVIRNCFVKNGEKINFFPKA

>MdirOBP3

RFTTEQIDYYGKACNASEDDLVVVKSYKVPSTETGKCLMKCMITKLGLLNDDGSYNKTGMEAGLKKYWSEWSTEKIENINNKCYEEALLVSKEVVATCNYSYTVMACLNKQLDLDKST

>MdirOBP4

QKQETSGKCRAPDKAPLNLEIIINICQEEIKSALLQEALDILNDGNLEQNTPASYSSRSKREADEDLTNEERRVAGCLLQCVYKKVKAVDETGFPVVDGLMKLYNEGVQDRNYYMATLSAVRHCISIAQQLKQQQPSKSFDDGQTCDLAYEMFECVSEKIEENCGVENKSNN

>MdirOBP5

DAGHHRRGKELLDTEDSDFFRCKQASRKSCCGPENAMKRFGDKDKVAADECYAQVAEKFATVTATTPKQDLFSAEAVKITKKKQFCLHECIGKKNNLLTEDGSLNKTFIADYAMKSVFKEQWQKQVGQKALDKCLEETYIPWPAKNKENVCNPVYVQFQHCLWLQYESNCPANKIKITKKCEKTRNRYRMQKSTSN

>MdirOBP6

PNILPNSNSTWAKCFETFKQFKDKPETKEYKEMAHGKEPPCLFQCIFVQSGLTTSDGKLNEDAITKKMSEGINNDEKWKSTWQNSLNKCFDDVKQEDKKQILIMNTPAGRLMKCFLRDMYMSCPKSVWVESSECLNMKDLVQKCPEMPPPVFKSPPKLI

>MdirOBP8

ENNQQNSNDRSATIFQSCISETKLSGDALKGFRSMSIPKTQAEKCMMGCLMRKVNVINKGKFSVEEATKVAQKYYGTNGTMMKKAKDLIDVCAKKAQSTTEECALAGIVTTCIVEEAQKAGLSGGPGSRSRRTVSPKFRRNSM

>NribOBP3

RFTTEQIDYYGKACNASEDDLVVVKSYKVPSTETGKCLMKCMITKLGLLNDDGSYNKTGMEAGLKKYWSEWSTEKIETINNKCYEEALLVSKEVVATCSKSHDRKACLNQDPDLDKST

>NribOBP2

MTVTPTHLPVQSSSTPTSHPQTTYAKDHVHGSTTTKSGANATATTASGASVNGTERPAVVKSSAGVTGNFTTPKPTMTEGHVALKQKLNTIAVKCKDELHAPQEIMALVSNTVVPQNEQQRCYLECVYKNLNLIKNNKFSVEDGKAMARIRFANQPEEHKKAVTIIETCEKEAVIDPKTTEKCAAGRVIRNCFVKNGEKINFFPKA

>NribOBP5

DAGHHRRGKELLDTEDSDFFRCKQASRKSCCARKNAMKRFGDKNKVAADECYAQVAEKFATVPATTHKQDLFSAEAVKITKKKQFCLHECIGKKNNLLTEDGSLNKTFIADYAMKSVFKEQWQKEVGQKALDKCLEETYIPWPAEDKENVCNPRYVQIQHCLWLLSRRNIPAHKSKITKKCEKTRNRYRMQKSTSN

>NribOBP7

YLSEAAIKKTQHMLKTVCSKKHSVDEDVFTEIKKGIFPEDNNDIKCYFACNFKTMQLVNQKGYIDKKLFKDKMSIMAPPNVYNILLPVIEQCAGIDKSEELCQSSYNLIKCAHRVNPKSLEFLPL

>NribOBP8

ENNQQNSNDRSATIFQSCISETKLSGDALKGFRSMSIPKTQAEKCMMGCLMRKVNVINKGKFSVEEATKVAQKYYGTNETMMKKAKDLIDVCAKKAQSTTEECALAGIVTTCIVEEGQKAGLTGGPGGRSRRTVSPKFRRNSM

>RpadOBP2

SDPCNISTCYKSGTTKPPTTVTPTRLPVQSSSTPTSHQQTTYAKDHAHSSIAAKSGANVTATTASGATVNGTERPTVVKSSPGVAGNATTPKPTMTVEHVALKQKLNTIAVKCKDELHAPQEIMALVSNTVVPQNEQQRCYLECVYKNLNLIXNNKFSVDDGKAMARIRFANQPEEHEKAVTIIETCEKEAIIDPKTTEKCAAGRVIRNCFVKNGEKINFFPKA

>RpadOBP3

MISPTFYISLLFSIGMLISCSFGRFTTEQIDHYGKACNASEDDLVIVKSYKVPTSDTGKCLMKCMISKLGLLNDDGSYNKTGMEAGLKKYWSEWSTDTIENINNKCYEEALLVSKDVVATCNYAYVVMACLNKQLKLDKST

>RpadOBP5

DAGHHRRGKELLDTEDSDFFRCKQASRKSCCGPDNAMKRFGDKDKVAADECYAQVAEKFATTKATTPKQDLFSSEAVKVTKKKQFCLHECIGKKNKLLTEDGSLNKTFIADYAMKSIFKEQWQKQIGQKALDKCLEETYIPWPAEETENKCNPVYVQFQHCMWFEYESNCPSNKIKLTKKCEKTRNRYRMQKSTSN

>RpadOBP7

MNMLPATVLLAVIAATVLKDSDAYLSEAAIKKTQQMLKNVCSKKHSVGEDVFTDIKKGIFPENNNNIKCYFACNFKTMQMINPKGILDKKMFKDKMTMLAPPNVLEILLPAIEQCIGTDKDTEICQSSYNFIKCAYRVDPKSLEFLPL

>RpadOBP10

STRPQPDEMEEIKKTLYNACSAKFPLTDEIRNNAKNSIVADDQNLKCFLRCCFDEMSMIDEDGIIDGESLVSMTSDKLKIVAKKAVDSCLTADKQDGCEAAFKFISCGIKLNPLIGSATL

>AglyOBP2

MKVSAATAVLVALVATVQSSDPCNISTCYKSGTTKPPTTVTPTRLPVQSSSTPTSHQQTTYAKDHVHSSTATKSGVNTTATTTSGASVNGTERTTVVKSSSGVAGNVTTPKPTMTDGHLALKQKLNTIAVKCKDELHAPQEIMALVSNTVVPQNEQQRCYLECVYKNLNLIKNNKFSVDDGKAMAKIRFANQPEEHKKAVTIIETCEKEAIIDPKTTEKCAAGRVIRNCFVKNGEKINFFPKA

>AglyOBP3

MISSTFYTSLMFGIVMLISCSFGRFTTEQIDHYGKACNATEDDLVVVKSYKVPTSDTGKCLMKCMISKLGLLNDDGSYNKTGMEAGLKKYWSEWSTDTIESINNKCYEEALLVSKDIIATCNYAYVVMACLNKQLDLDKST

>AglyOBP4

MRGNYSLVVFLLFGFGLLEIYCQKQETSGKCRAPDKAPLNLEIIINICQEEIKSALLQEALDILNDGTLEQNTPSYSRSKRDADEDLSNEERRVAGCLLQCVYKKVKAVDETGFPVVDGLMKLYNEGVQDRNYYMATLSAVRHCISIAQQLKQQQPSKSFDDGQTCDLAYEMFECVSEKIEENCGVENKSNN

>AglyOBP5

MKRFGDKDKVAADECYAQVAEKFATVTATTPKQDLFSGEAVKITKKKQFCLHECIGKKNKLLTEDGSLNKTFIADYAMKSVFKEQWQKQIGQKALDKCLEETYIPWPAEETENKCNPVYVQFQHCLWLEYESNCPDNKIKLTKKCEKTRNRYRMQKSTSN

>AglyOBP6

MQKVVFLCIFAIICQTVFTVGFERTWILRQKRVTNDDECRTLIPSSEKKLPTCCQMPNILPGLDNAWEVCFEKFKQFKDKHATKEYKEMAHGNEPPCLFQCVFMQSGLTTSDGKVNEDAVIKKMAEGMDNDEKWKSIWRNTFNKCLNDVKQEDKEQIKMTNTPTGRLMKCFLRDLYMNRPKNVWVESSECSNLKDLVEKCPKMPPPVFKSPPKLI

>AglyOBP7

MVARKRMYMLPATVLLAVVAATILKDSDAYLSEEAIKKTQKMLKNVCSKKHSVEEEVFTDIKKGIFPENNNNIKCYFACNFRTMQMVNQKGILDKKMFKDKMTMLAPPNVLAILLPPIEQCIGNDKDTEICRSSYNFIKCAHRVDPKSLEFLPL

>AglyOBP8

MFAFKVACLCLSVAVVFGENNQQNSNDRSASIFQSCISETKLSGDALKGFRSMSIPKTQAEKCMMGCLMRKVNVINNGKFSVEEATKVAQKYYGTNETMMKKAKDLIDVCAKKAQSTTEECALAGIVTTCIVEEAQKAGLTGGPGSRSKRTVSPKFRHSIV

>AglyOBP9

MIIKKTLLVSGFVLFGCMFSINKAADDADAKDKELMSKLITVAFKCFKDADWGTCGEMITTKYDITQAKYKQCTCHMACAGEDLGLINSNGQPEPAKFLEYVKRINNSVIKSQLQHIYDKCQNVKGTEKCDLAEQFAICAFKESPEMKERVTKLIEMLVKMKPKSK

>AglyOBP10

MEHLRGTNVVFAIVMALLVVQSSTRPQPDELDDIKKTLYNACSEKFPLTEEIKNNVKNSIVIDDQNFKCFLRCCFDEMSLIDEDGIIDGESLAAMAVDKIKPVAEKIVHDCLPAGKQEKQDGCEASFKFFSCGIKLNPLTIELLPLQ

>SfurOBP1

MLLEVCRFSVFLLAFSATVYGRFSEEEKQLMNQVHTQCVTETGTSEDLVNKATNGDFAEDENLKCYVKCIWSTLTVMDDDGNFDVGVLEVMLPADMKDIVMKAMSACIGAGGGSPCEKAFAVTKCLYKEAPADFFLP

>SfurOBP2

MSTLLNFVFVFLVCLCSYSEASPALTEAQIEQVGKAMANMCISSSGVQRSLITKAMTGEIEDDRKLKCFFGCIMEAVQVTKNGKMQPEVLKRRANAMLPKTMREMILPTIDSCSHIENEDKCELAYSIVKCHFSVNGKNPFFFNF

>SfurOBP3

MPPVFRTAHDKFESCLEELSSIFPPPPPPNGHHGPPPPGGPGGHGPPPPPPPGGRRGPPPGFGGPPGHEPPIFACAHECLFNKTGLLENGKLNVEALKKKLEGELGDDEVWKNLLQSIVDKCMESKDPPSNDMCTSGSHELARCVLRDMFMNCPQEKWKESDDCSNMKMKLEKCPELVPPMAMRLSQPPMP

>SfurOBP4

MCCDLPLVYRGTPELFKACREELGFPDHKPPPPPPSSDGHGPHGHPQRGMCVAECLFNRTGLLENGKINKEALKKALDEYLKTDGAWKDVATTTLEICYDAQTRGDFKPDNEKFTSGSSEFLKCFTRNLFMDCIPEKWTDSEECKKMKEKIDKCPKMLPPALFNKRPH

>SfurOBP5

MLYVSYFVIVTAASSAVITQIMAADSNNPDMQTVFNNCREEASATEDDIKTFRAQQIPSTTTGKCMLACMFNHSGLMKEGKYDSEGALKLVGQVFAADPVKLGKAKTLINTCSDEVKNENDKCEIASKIADCTVKMTSQVGLS

>SfurOBP6

MKCQVFLASFVLVAVFELGYAGLTPEKLKEIKPLIDTCIKESKVEEETLGKLHNGHEIPSSQSGKCFIACMAEHMKLMKDGKFEPAMTMEFIDKMVQDKVKADEIKKAVDDCFKSVPDGDKCEMAASLATCMKEHHAELAGMN

>SfurOBP7

MEEEMLSIGRVQRDADQTQEVADEYFKCKHRNLKTCCGKINLMKNYGDKGKIYGKQCYEEVVSAFKTNSSSTADDDDSMMDMFSCEKVKMIKLKHICVHECIGKKTKILKEDGSLNAEEIKQYAREYMFNEEWSKELGERALDKCLTQSYNSVTKMLDEYEIKCNPTSVQFHHCLWKEIEMTCPESKVDLKAKCVRLRERLRKQQAAGM

>SfurOBP8

MERTHVLIIAFAFIPFLSSAMQADFAMMQFPMQGTGTPMIQSIAGELKYCMDVNAEQNSDGLEDYLPLLFNEELPTSLGQKCFLTCLFNRFGLLKDGFLDAQTAKTLVETFYKDKHDEKTMANIAINVCRVSAVPDILNPCEIGFSLKSCFVDSNKKGKELRGKN

>SfurOBP9

MNTFQKFILSGMVVLAGAMLITAEDTTIKIKNQQSPHKQQQVYCQAPPTAPERLERIIEQCQDDIKTALLQEALNVLTDTSPRDLVKKTRSKREVFSGEEKRIAGCLLQCVYRKVKAVNDQGMPTVPGLVRLYSEGVQDRNYYVATVQAVQQCVSASQHFRYYNPQVLKEDGYTCDLAYDMFNCVSDKIEAFCGRTP

>SfurOBP10

MASGLVAAELEKLKNSCLKKSGATEDTARRLVGINVVTENHVESCFLTCIYKGLKIVSSDNKFQPDTVKKIADDHFLGRNLKVTYQIADSCTKEIKADPADKCSIGASFRNCFSKYGKELGFFPHM

>SfurOBP11

MLLEVCRFSVFLLALSATVYGRFSEEEKQLMNQVHTQCVTETGTSEDLVNKATNGDFAEDENLKCYVKCIWSTLTVMDDDGNFDVGVLEVMLPADMKDIVMKAMSACIGAGGGSPCEKAFAVTKCLYKEAPADFFLP

>SfurOBP12

MRGYILVLCVLLFMRGMASGLVAAELEKLKNSCLKKSGATEDTARRLVGINVVTENHVESCFLTCIYKGLKIVSSDNKFQPDTVKKIADDHFLGRNLKVTYQIADSCTKEIKADPADKCSIGASFRNCFSKYGKELGFFPHM

>LeryOBP3

RFTTEQIDYYGKACNASEDDLAVVKSYKVPSTETGKCLMKCMITKLGLLNDDGSYNKTGMEIGLKKYWSEWSTEKIEAINNKCYEEALLVSKEVVATCNYSYTVMACLNKQLDLDKST

>LeryOBP7

MVARKRMYNMLPTNVLLTIIAATVLNDCDAYLSEAAIKKTQQMLKSVCSKKYTVEEDVFTNIKKGIFPEDNNNIKCYFSCVFKTMQMINQKGSLDKKIFKEKMSMMAPPSVYNILLPAIEQCIGKDNGEELCQASYNFIKCAHHIDPKSLEFLPL

>DplaOBP3

MISSTFYITSVFGIAMLISCGYGRFTTDQIDYYGKACNASEDDLVVVKSYKVPSTETGKCLMKCMITKLGLLNDDGSYNKTGMEAGLKKHWSEWSTEKIENINNKCYEEALLVSKEVVATCNYSYTVMACLNKQLDLDKST

>BbraOBP3

MISSTFYITLLFGIAMIISCSYGRFTTDQIDYYGKACNASEDDLVVVKSYKVPSTETGKCLMKCMITKLGLLNDDGSYNKTGMEIGLKKYWSEWSTEKIEAINNKCYEEALLVSKEVIATCNYSYTVMACLNKQLDLDKST

**Table S9.**

>AlinCSP1

MLKVLVLLAAVVCCVSAAATYTSKYDNIDLDEILSNTRLYKKYFDCLANKGKCTPDGKELKESLPDALKTNCAKCTKKQQEGTDKVFRHVLKNKPNDYKVLESIYDPPGIYRKKYEAEAEKRGIKLPGSH*

>AlinCSP2

MKVAVLVLLCVGAALSAEVYTSKYDNIDVDKILSNDRILTRYIKCLMEEGNCTNEGKELKKTLPDALASGCTKCSEKQKAQTEKVLRHLSKNRPRDWALLKTKYDPKGEYSKKYEKEAKALTA

>AlinCSP3

MISKLSMVLLIGAFADVWAAEQYTDKYDNIDIDEILNNDRMYKNYFHCVMGNGKCTPDGLELKAKIPEALQTECAKCTDKQKKEVEKVLRFIINQKKDDYKLLEEKFDPEGVYRKKYEAQKKLVEEGKPIEY*

>AlinCSP4

MRIILSAFLVAMACSLATCEMTEEEFYTKVFEEVDPDFILDNERILTSYLKCFYNEIECNAHAEVVKKSIPDVLATVCGRCSDKQKSIFKYSLNKFIPAHPKDWEKILSIYDPSGEAWPKVKAFIES*

>AlinCSP5

MGHLTIVLLAAAFEVLTGSRAYTTHYDYIDVDQVLNNTRLYTKYVECLLGQGKCTPEARELRDKLPEALQTNCARCSERQASESHRVIRFLIQNRQEDFKLLEAKYDPSGLYFKRFEEETKRNVSLS*

>AlinCSP6

MFYKLSVVVLMGILAGVWAADKYTDKYDNIDIDEILTNERLYKKYFDCIQGTGKCTPDGIELKEKIPEALKTECAKCNEKQKAGVEKVMRYLITKKPEDFKILEDKFDPEGVYRKKYEAQRKLVEEGKPVEY*

>AlinCSP7

MNYKLSVILLIGVLASVWAASTYTDKYDNIDLDEILTNERLYKKYFDCIQGKGKCTPDGTELKEAIPDALKTECAKCNAKQKAGVEKVLRHLLTKKAEDYKILEDKFDPEGVYRKKYEAQKKLADEGKPIVL

>AlinCSP8

MDYKLSVMLLMGVLACAWAADKYTDKYDNIDIDEILNNERLYKKYFDCILGNGKCTPDGTELKETIPDALKTACAKCNDKQKAGVEKVLRHLLTKKAEDYKILEAKFDPEGVYRKKYEAQKKLAEEGKPIAL

>AlinCSP9

LAVVTREMREREFFRQLEVINVDSILINQRLIDKYIKCLLKTGKCDPIMKDLRIALPLILGHLCEARCSEK*

>AlinCSP10

MRSNFINESIPDVLATVCGRCSDKQKSIFKYSLNKFIPAHPKDWEKILSIYDPSGEAWPKVKAFIES*

>AlinCSP11

MKVFFSGLLLVCMASVSLCADEYTDKYDSVDLDEILNNQRLYQKYIDCVMGKGKCTPDGALLKEKIPEALQNECAKCSAKQKKGAEKVLRFLINEKADDYKALEEKYDPEGTFRSKYEEQKKNLKEGKPLSV*

>AlinCSP12

MMIIIVFGISALLVVVEGAPLQYSDTRYDDVELTTILSNDELYIKLFQCLIGRGKCTPDWEILKDALPGALLDNCSECSNKQKFGTKTLLAHLVHERPSDMRLLEGEFDPDGSYRKELEKEEKESNDINRKRSANLEEVEILDKIKRIIK*

>AlinCSP13

MKFVAALLVASVAVLAVEAANQYTTKYDNIDLDDILKNQRLYKKYFECLTGNGKCTPDGKELKEHLPDALKTGCSKCSEKQRAGSEKVIKHLLKNKPQDYAVLEKIYDPSGIYKKKYEAEAKKLGINV*

>AlinCSP14

MNSAIVLCVVALAGMVLARPDDTYTTKYDNVDLDEILGNDRLLVPYIKCTLDEGKCAPDAKELKEHIREALENGCAKCTDKQKEGTRRVIAHLIKHKNADWQKLKAKYDPEGKYTHKYEKELEEVQH*

>AlinCSP15

MKLIVAVALLCVVAESWAASTYTDKWDNINVDEILESQRLLKAYVDCLLDRGRCTPDGKALKETLPDALENECSKCTDKQKSGSDKVIRHLVNKRPEMWKELSAKYDPNNIYQDRYKDKIEAVKGQ*

>AlinCSP16

MLPFYVFSLCAVFVACQETYTSKYDNVNVEDALKNDRLYKAYFNCLADRGPCTREGNMLKEALPDGLRNNCSLCTDPQRRGTHQVIRFLFKYRPEDMKLLEEIYDPEGIYKTKYAEERKKLME

>AlinCSP17

MDYKFLVVMQMGVISSVCAAGPYTDKYDNVNLDEVLNNERLYRNYFNCLQGKGKCTLDGAILKEIIPSALKTDCALCSVRQKKGAEKVLIFLITKKPDDFKILEDKF

>AlinCSP18

MIWILLVAVSMTTSLAEEESIDYYRVFEEIDPDLILDNERILQTYLKCFYGEGPCNTHAQLAKESIPDVLA

>AlinCSP19

MVYKSSVVFLLMGTVAYVWGEKYTEQYDDINLDNILTNERLYRIYFKCILSKGKCTPEGEVLKKAIPDALK

>AlinCSP21

KELKEHIKEALENECGKCTEAQKKGTRRVIGHLINHEADFWNELTAKYDPERKYTTKYEKELKEVKA*

>AlucCSP1

MLKVLVLLAAVVCCVSAAATYTSKYDNIDLDEILSNTRLYKKYFDCLANKGKCTPDGKELKESLPDALKTNCAKCTKKQQEGTDKVLRHVLKNKPNDYKVLESIYDPTGIYRKKYEIEAEKRGIKLPGSH*

>AlucCSP2

MVGKLSVVLLIGAVGMVLAADKYTDKYDNIDVDEILGNQRLYQKYFDCIQGKGKCTPDGAELKKNIPEALQTDCAKCSEKQKAGVEKVLRHLINEKPEDYKVLEEQFDPEGVYRKKYEHLKKKVEEGKPVEY*

>AlucCSP3

MLKVLVLLAAVVCCVSAAATYTTKYDNIDLDEILSNQRLYKKYYDCLANKGKCTPDGKELKEALPDALKTNCSKCSKKQQEGTDKVLRYVLKNKPNDYKVLENIYDPSGNYRKRYEDEASKRGIKLPGSH*

>AlucCSP4

MVSKLSIVLLIGALADVWASELYTDKYDNIDVDEILGNQRLYQKYFDCIQGKGKCTPDGAELKKNIPEALQTDCAKCSEKQKAGVEKVLRHLINEKPEDYKVLEEQFDPEGVYRKKYEHLKKKVEEGKPIEY*

>AlucCSP5

MVGKLSVVLLIGAVGMVLAAELYTDKYDNIDVDEILGNQRLYQKYFDCIQGKGKCTPDGAELKKNIPEALQTDCAKCSEKQKAGVEKVLRHLINEKPEDYKVLEEQFDPEGVYRKKYEHLKKKVEEGKPV*

>AlucCSP6

MVSKLSIVLLIGALADVWASELYTDKYDNIDVDEILGNQRLYQKYFDCIQGKGKCTPDGAELKKNIPEALQTDCAKCSEKQKAGVEKVLRHLINEKPEDYKVLEEQFDPEGVYRKKYEHLKKKVEEGKPIEY

>AlucCSP7

MVSKLSIVLLLGALADVWAAELYTDKYDNIDIDEILNNDRMYKNYFNCVMGNGKCTPDGLELKAKIPEALQTECAKCSDKQKKGAEKVLRFIINQKKDDYKLLEEKFDPEGVYRKKYEAQKKLAEEGKPIEY

>AlucCSP8

MLKVLVLLANAASTYTTKYDNIDLDEILSNQRLYKKYYDCLANKGKCTPDGKELKEALPDALKTNCSKCSKKQQEGTDKVLRYVLKNKPNDYKVLENIYDPSGNYRKRYEDEASKRGIKLPGSH

>AsutCSP1

MLPFYVFSLCAVFVACQETYTSKYDNVNVEDALKNDRLYKAYFNCLADRGPCTREGNMLKEALPDGLRNNCSLCTDPQRRGTHQVIRFLFKYRPEDMKLLEEIYDPEGIYKTKYAEERKKLME

>AsutCSP2

MGHFPPVFSLSPVLLVASLHTMNTSTLLKIAFLLGCVAACLAAETRSSVSDEALEAALKDKRYLTRQLKCALGEGACDPVGRRLKTYAPLVLRGACPKCTPSEVRQIQQVLSHIQRHYPKEWAKILKQYAGQ*

>AsutCSP3

MKFVAALLVASVAVLAVEAANQYTTKYDNIDLDDILKNQRLYKKYFECLTGKGKCTPDGKELKEHLPDALKTGCSKCSEKQRAGSEKVIKHLLKNKPQDYAVLEKIYDPSGIYKKKYEAEAKKLGINV*

>AsutCSP4

MRIILSAFLVAMACSLATCEMTEEEFYTKVFEEVDPDFILDNERILTSYLKCFYSEIECNAHAEVVKKSIPDVLATVCGRCSDKQKSIFKYSLNKFIPAHPKDWEKILSIYDPSGEAWPKVKAFIES*

>AsutCSP5

MDYKFFVVMQIGVISSVCAAGTYTDKYDNVNLDEVLNNERLYRNYFNCLQGKGKCTLDGAILKEVIPSALKTDCALCSVRQKKGAEKVLIFLITKKPDDFKILEDKFDPEGVYRKKYEAQRKLVEEGKPIH*

>AsutCSP6

MVCKLFAVVLMGILAGVWAADKYTDKYDNIDIDEILTNERLYKKYFDCIQGIGKCTPDGIELKEKIPEALKTECAKCNEKQKAGVEKVMRYLITKKPEDFKILEDKFDPEGVYRKKYEAQRKLVEEGKPVEY*

>AsutCSP7

MVSKLSMVLLIGALADVWASELYTDKYDSIDIDEILNNDRMYKNYFNCVMGNGKCTPDGTELKAKIPEALQTECAKCSDKQKKGVEKVLRFLIKEKKDDYKLLEEKFDPEGVYRKKYEAQKKLVEEGKPIEY*

>AsutCSP8

MDYKLSVMLVMGVLACAWAADMYTDQYDNIDIEEILTNERLYKKYFDCIIGNGKCTPDGTELKETIPDALKTACAKCNDKQKAGVEKVLRHLLTKKAEDYKILEAKFDPEGVYRKKYEAQKKLAEEGKPIVL*

>NlugCSP1

MFKNVLLVCLLVAVVSAKPKPAEKKQYTTKYDNIDLDEILNNQRLFDNYYKCLLGGKCTPDGQELREALPDALATACSKCTEKQRVGTEKVIKYLIEKKPTEYSELEKKYDPQGNYKRKYQAEAAKRGIKV*

>NlugCSP2

MSKLPVTLVLMLAVFSVDCGKLYKDRYTTKFDKIDLDEALNNQRLFESYLKCLMGDKCSPDGYELREALPDALATACAKCSEAQKAGTEKVIRFLIEKRPKEYALLEKKYDPEGIYRDKYKPIAEMKGIKLD

>NlugCSP3

MKFLCVTIFECALIVVAFGMPQDTTYPTTYDDVNVDDILHNDRLFNRYFTCLTKKEGCTPEGKLLAATIPDALATTCAKCSAKQKTAAEKVIKYLYFNKRDKFDELAKIYDPESNYLNKYLVDGFPAKV*

>NlugCSP4

MFLIAVWALSPRRLPWGLPWGGLAGVAAQQQAKNTRYTTRFDSIDVEVILKNERIFRRYMDCLLDKGRCTPEARELKRLLPEALKTECLKCSEVQRRQGAKVMAFIIKNKRPSWELLLAKYDPQGIFRAKYMYNENNIEAVLKQLEREQQGIYGTYSSTNSTTSSNSTSIR*

>NlugCSP5

MRCLLLVAVVCAALVAVCHAQDSKYTSKYDNIDIDKILKNDRVLSQYIKCLMGEGSCTQEGRELKRLLPDAIQSNCSKCSEKQRSASVKVMRHLRQSRERDWNRLLDKYDPQGDKRKNLKLD

>NlugCSP6

MLWAARFIVLPLLFCVLQVWSAPADEKYTDIDFDSILANRRVLSSYVKCLTDKGPCTPQGKELKKIVPEVIQTSCTKCSPQQKKVVRNVITTMQSKYKDQWDLVVNKYDPKKQRSGELKAFLSGTD

>NlugCSP7

MASASSGTTSTTSAPKTAESASAKSSSKDEIPDQTFDRYINNERYMLMQYECLMGNKPCDHVGRKLKAAVPLVVRGLGCPKCSQREEDQMKRIVSHVQRSYPDKWQKLIKKYGN

>NlugCSP8

MSSTMLVFVAVLCFSAVLAKPADKYTTKYDNIDLDEVLSNQRLFDSYFKCLMGGKCTPDGQELRDALPDALATACEKCSEKQKEGTEKVMKFLIEKKPTEFAELEKKYDPQGTYRQKYKAEADKRGYSV

>NlugCSP9

MKSQQLLVSCLFICTWLVVLMAPSANAAPKEKDPERKALYRLEYIDIEKVLDNNRMLTNFIRCFLRQGPCTPEARDFRKLLPKLAKTMCSDCTARQRYIIKKVFKHLMEERPKEWELLMDRFDPQRKYAERLDTFMVDMTTRAPVTSSPMPSSPVTLTSSSVTMSSTTQRVIEILRTSTDMSNESRPAS*

>NlugCSP10

MFMLLACSELGSGQQQQNVDNIEMSIYDKMFENMDVNSLLKNHRLVDSYLKCFLNEGSCTHIGHEVKMMIPEVIKSRCGTCGENQMRALKAGLRLFIVLRPDDWQRFLDVYDPDRKEWPHIKAFMDSDD*

>NlugCSP11

MKSIILLVFVSMSAMVYRCRADEPSYPTSWDNVNIDEVLGNERLVQNYAKCLLEKGSCSPEGTELKKAIPDALKTGCTKCSDKQKAGAQKVIKWLVQKKPELWKEVVDKYDPSGEYTKKYEKEYQI*

>SfurCSP1

MFNLLTLVVCLSTIAVQIQAAPEEAQYTTKYDKINLDEILNNDRLFKSYFGCLMGGKCTPDGQTLRDILPDALETACSKCSDTQKAGTEKVFKFMIEKKPSEFADLEKKYDPNGKYRARYEADAEKFGIKV*

>SfurCSP2

MVLADTPTTSPKVETKAVESGKSSSKDEIPDQTFDRYINNERYMLMQYECLMGNKPCDHVGRKLKAAVPLVVRGLGCPKCSQREEDQMKRIVSHVQRSYPDKWQKLIKKYGN*

>SfurCSP3

MQLLYALVFGCTLVMVSSDMPQSTYPTKYDDYNPDDILKNDRLFNQYFICLTKKKGCTTAGELLSAIIPDALATSCAKCSAKQKAIGEKVIRFLYFNKPDEFAEMSKIYDPEGKYLEMYIASGGLI*

>SfurCSP4

MKCPLLSVSCLWISLLALSSSASAATKEKDPERKALYRLEYIDIEKVLDNNRMLTNFIRCFLRKGPCSPEARDFRKLLPKLAKTMCSDCSPRQRFIIKKVFKHLMEERPKEWELLMDRFDPQRKYAERLDTFMVDMTTPSTTTTTTSTTPSTPMSSTTQRIIEILRTSTEMSNESSP*

>SfurCSP5

MSEILVTSLIFMLLAASELGLGQQQQTQKPQQQNVDNIEMSIYDKMFENMDVNSLLKNHRLVDSYLKCFLNEGSCTHIGHEVKMMIPEVIRSRCATCGENQMRALKAGLRLFIVRRPDDWKRFLDVYDPDRTEWPHIKAFMESDD

>SfurCSP6

MKLALFCCLLGLVAAVSAQSEKSEKPEKYTTKYDYINVDEILSNDRLFNSYYKCLMGGKCTPGGPELRTHLPDALQTNCSKCSEKQKEFSDKVIKHLMDNKPEEFSALVKKYDPEGIYKDAFKPKHNQ*

>SfurCSP7

MRASKASSLVAVLLIAVWGFTGVQAQQKSKDTRYTTRFDSIDVEVILKNERIFKRYMDCLLDKGRCTPEARELKRLLPEALKTECLKCSEVQRRQGAKVMGFIIKNKRPYWDLLLAKYDPQGIFRAKYNYNENNIEGVLKQLEREQQGLYGTYSNTTNTTNTVNSTSTRK*

>SfurCSP8

MLKFTLTLLVLAVVSVNCGKLYKDRYTTKFDKIDLDEALNNQRLFESYLKCLMGDKCSPDGYELREALPDALATACAKCSDAQKAGTEKVIRFLIEKRPKEYALLEKEYDPEGIYRDKYKPIAQEKGIKI*

>SfurCSP9

MRCLLLVAVVFAAFIAAARADEANKYTSKYDNIDIDKILKNDRVLSQYIKCLMGEGSCTQEGRELKRLLPDAIQSNCSKCSEKQRQASVKVMRHLRQSKERDWNRLLDKYDPQGDKRKNLKLD

>LstrCSP1

MASVSSATLTAAALLALLALQLTAAQNFNEADIARMLNDSGLVQRQISCILGEAACDNIGNMLKLAIPEVLKRNCRSCNAQQASNARRLISFVQANYPAQWQRIQSRYVG*

>LstrCSP2

MRCLLLVAVICAAFIAAAQADEANKYTSKYDNIDIDKILKNDRVLSQYIKCLMGEGSCTQEGRELKRLLPDAIQSNCSKCSEKQRQASVKVMRHLRQSRVRDWNRLLDKYDPQGDKRKNLKLD

>LstrCSP3

MKLALFCCLLGLVIAVSAEKYTTKYDHINVEEILNNERLFNSYYKCLMGGKCTPDGLELRTHLPDALRTNCSKCSEKQKEFSDKVIRYLIDNKPEEFAALTKKYDPEGIYKTTFGPQFKKDNTTTNQ*

>LstrCSP4

MLWAAKFIVFPLIFCVLQVWSAPADEKYSDIDFESILANRRVLSSYVKCLTDKGPCTPQGKELKKIVPEVIQTSCTKCSPQQKKVVRNVITTMQSKYKDQWDLVVNKYDPKKQRAGELKAFLAGTD*

>LstrCSP5

MLKFKLTLLVMASAFFSVDGGKLYKDRYTTKFDKIDLDEALNNQRLFESYLKCLMGDKCSPDGYELREALPDALATACAKCSEAQKAGTEKVIRFLIEKRPKEYALLEKKYDPEGVYRDKYKPIAEEKGIKI*

>LstrCSP6

MQASSLAMLLIAVWVLSPRRPLSGGFAGVHAQQSKNTRYTTRFDSIDVEVILKNERIFKRYMDCLLDKGRCTPEARELKRLLPEALKTECLKCSEVQRRQGAKVMAFIIKNKRPYWDLLLAKYDPQGVFRAKYKYNDQNIEAVLKQLEREQQGLYGTYSNPTNTTTVNSASSRK*

>LstrCSP7

MIQRTQGFNSIVVLLLIKLTVLSMVLASTHAPAPTPTPKVETKATEAAKSSSKDEIPDQTFDRYINNERYMLQQYECLMGNKPCDHVGRKLKAAVPLVVRGLGCPKCSPREEEQMKRIVSHVQRSYPDKWQKLIRKYGQ*

>LstrCSP8

MKSPCLLSVSCCILVLVASSASAAPKEKDPERKALYRLEYIDIEKVLDNNRMLTNFIRCFLRKGPCSPEARDFRKLLPKLAKTMCSDCSPRQRFIIKKVFKHLMEERPKEWELLMDRFDPQRKYAERLDTFMVDMTTRATPTTTTTTIPTTTTPMSSTTQRIIEILRTSTEMSNESRP*

>LstrCSP9

MSEVLVMILIFMLLAGREQRLQQQQQQQQQPQPQQQNVDNIEMSIYDKMFENMDVNSLLKNHRLVDSYLKCFLNEGSCTHIGHEVKMMIPEVIRSKCATCGENQMRALKAGLRLFIALRPDDWKRFLDVYDPDRTEWPHIKAFMEYDD*

>LstrCSP10

MYDHINVYNILKNERLFNRYFTCLTKKEGCTPEGKLLAAAILDALETSCANCSNEQRKLAEQVIQYLYFNKRDKFDELAMIYDLEGVFQEYHIAEYLVSGSWMPDFRKLPPV*

>LstrCSP11

MKFLYFTVFGCALVMFTSAIPEIIFTSAMPQKTYSTMYDHINVNNILKNDRLFNRYFTCLTKRGGCTPEGKLLAAAILDALETSCANCSNEQRKLARQVIQYL*

>LstrCSP12

MFKNLLVVCLLVAAVSAKPKPAEKKNTTKYDNIDLDEILNNQRLFDNYYKCLLGAKCTPDGQELKEALPDALATACSKCTEKQRVGTEKVIRHLIEKKPTEYAELEKKYDPQGTYKRKYQAEAIKRGIKV*

>AgosCSP1

MNILTIFCYVTVMCDTQVKPAVSAQRLQSVNQNVTPTNDGRKTIRETSSYPTRYDYIDIEAVMNNERIIKILFNCVMSRGPCTREGLELKRIVPDAIQTECAKCNERQRKQAGKVLAHLLQYKPEYWKMLVQKFDPNNVYLRKYMADNDDDEKLSLQKLSNDTTKKKRNI*

>AgosCSP2

MAHLNLFVVLIASLIYFTSAAEEKYTTKFDNFDVDKVLNNNRILTSYIKCLLDEGNCTNEGRELKRVLPDALKTDCSKCTDVQKDRSEKVIKFLIKNRSTDFDRLTAKYDPTGEYKKNLEKFEKERASAKPLKA*

>AgosCSP4

MDSRIAVVCVVLAAFAVDQTVGAPQKDAVAASGPAYTTKYDHIDVDQVLASKRLVNSYVQCLLDKKPCTPEGAELRKILPDALKTQCAKCNATQKNAALKVVDRLQKDYDAEWKQLLDKWDPKREHFQKFQQFLAEEKKKGFTKF*

>AgosCSP5

MHCKVLIALCCVAVYAVQASPAGTATAAAVSADDEIKDFPAYMKRFDKLNVEQVLNNDRVLASHLKCFLNEGPCVQQSRDLKRVIPVIANNGCNGCTERQMTTIKKSLNFLRTKKPTEWARLVKIYDPSGTKLNKFLDA*

>AgosCSP6

MIKLILAIAFCVSITMTVVQTAPAKYTTKYDNVNIDEILNNDRLVASYFKCLMETGKCTPEGEEIKRWLPEAIENKCEDCSEKQKLGSEKIIKFLFEKKNDMWKQLEAKYDPQGTYRQRYAEEAKKLNINV*

>AgosCSP7

MSRSSSSVTMKVFVIAICVCAALARPEDVKVENKPAVIKSETLAAPLPTNIVKRATDTIQLDSSLPNVSEDVLDKALSDRRFVQRQLKCATGEGPCDPIGRKIKAHAPLVLRGMCVKCSQSEIKQIQRVMSHIQKNYPKEYTKMLKQYQSGF*

>AgosCSP8

MNNIIMNNSRGRYGIFSLLAVTIAAIMLVHQPATVRCADGGIITPQQQQQQTMMFTAPTGYYVSTYDHIDVGRLLRNNKVVSGYVKCFVNEGPCTPDGKLVKAYLLPEIIRTVCGKCTPRQKDMARMVLKHIYTYRQADFEKIMQIYDTDGKRNEILAFMNH*

>AgosCSP9

MSAFCLNSFILMTMITVIVTHATFTRSTKFDDRTGIDIHLVKRDTDDVNDDENSVESDEGFFYRFTHFFQDSSDKEDDDDDEKKPDFITTFDIFKLLDEEYAMQQFYCVINEDPCDEVGMRLKATIPEEINRNCERCTSTERNNIRRILNYVKKHYPQFWKRVEPIYKKKI*

>AgosCSP10

MINTRPRKLVRCIRGVSISVAKGDDAVNAENKDDDSHLVNREEIQRYMSMMEKINIDQMLNNTRLMSNNVKCFLNEGPCTAHLREMKKMVPMLVKDSCSSCTKEQKIMMKKAMDAVKARRPNDYEKLSKFFDPEGKYEKKFLENLNESK*

>MperCSP1

MNLLAVFCYITMMCDSQLFKRLEQPAAISQVKRIEQPAMIANRIGQPTVAPRFGQPTIAPRFGLPTIAPQVGQAAITPQVGQAAIASRFGLPTVAPQVGQAAITPQVGQAAIASRFGLPTVAPQVGQAATTPQVGQAAIASRIGQNFQNANNSVSPTTDGRKTTRETSSYPTRYDFIDIEAVMNNERIIKILFNCVMNQGPCTREGLELKRIVPDAIQTECAKCNERQRKQAGKVLAHLLQYKPEYWNMLVKKFDPNNVYLKKYMADNDDDEKVSLQKLTNDTTK*

>MperCSP2

MAHLNLFVVLVASLVCFTLAEEKYTTKFDNFDVDKVLNNNRILTSYIKCLLDEGNCTNEGRELRKVLPDALKTDCSKCTEVQKDRSEKVIKFLIKNRSTDFDRLTAKYDPSGEYKKKIEKFDSEKAAAAKH*

>MperCSP4

MDSRIAVVCVVLAVFAVDQTVGAPQKDAVAASGPAYTTKYDHIDIDQVLGSKRLVNSYVQCLLDKKPCTPEGAELRKILPDALKTQCVKCNATQKNAALKVVDRLQRDYDKEWKQLLDKWDPKREYFQKFQQFLAEEKKKGVVKF*

>MperCSP5

MNCKVLIALCCVAVYAAHASPAGAATAAAASADEEIKDFPAYMKRFDKLNVEQVLNNDRVLASHLKCFLNEGPCVQQSRDLKRVIPVIANNGCNGCTERQMTTIKKSLNFLRTKKPVEWARLVKIYDPSGTKLNKFLDA*

>MperCSP6

MNTLLLAVALCIAITMTVVQTAPAKYTTKYDNVNIDDILNNDRLVASYFKCLMETGKCTPEGEEIKRWLPEAIENKCENCSEKQKIGSEKIIKFLIEKKNDMWKQLEQKYDPQGLYKQRYSEEAKKLNLDV*

>MperCSP7

MDRSSSSVTMKVFVIAVCVCAALARPEDSKVENKPAAVKSETLAAPLPTTIVKRATPQVVSTQQGASLPNVSEDVLDKALSDRRFVLRQLKCATGEGPCDPIGRKIKAHAPLVLRGMCVKCSQSEIKQIQRVMSHIQKNYPKEYTMMLKQYQSGF*

>MperCSP8

MTNNNMNSPRCRPEIFSLLAVAAIATVLVHQPSTVHCADAGVYPPQQQQQEATMFTAPSGYYVSTYDHMDVGRLLRNNKVVAGFVKCFTNEGPCTPEGRLAKAYLLPEIIRTVCGKCTPRQKDMARLVIRHIYTYRRGDFDKIMQIYDTDGKKNEIIDFMNQK*

>MperCSP9

MTSFCLNSVILMTITTVIVAHAASTGMTAFNNRSGSDIHMAQRDYNENKADKAEGFFFTITNFFSRRKHDDDKPDFITTFDIIRLLDEKYAMKQFYCVINKEPCDATGLRLKATIPEEINNDCERCTATETSNIRRILNYVKKHYPEFWDRVEPIYRNNMTA*

>MperCSP10

MVSKLFVSVFVLMSVVGVSYSVTEGDDDAAKVADKDLHPVNQEELKKFLSMMEKVDIDQILNNNRLMSNNVKCFLNEGPCTGQLREMKKMVPMLVKDSCSSCNKEQKNMMKKAMDAMKARRPNEYEQISKFFDPEGKYEKKFLENLNESK*

>ApisCSP1

MNLLAIFCYITMMCDSQFRRLEQMTAMPQVKQPATIATRIGQATIAPRFGQPTIAPRFGQATVAPQVGQAAVTPQIGQAAIGSRIGQSFQSVNGSVTPTTDGRKTTRETASYPTRYDFIDIEAVMNNDRIIKILFNCVMNQGPCTREGLELKRIVPDAIQTECAKCNERQRKQAGKVLAHLLQYKPEYWNMLVKKFDPNNIYLRKYMADNDDDEKLSLQKLTNNTTK*

>ApisCSP2

MAHLNLFVVLVASLVCFTLAEEKYTTKFDNFDVEKVLNNDRILTSYIKCLLDQGNCTNEGRELKRVLPDALKTDCSKCTDVQKDRSERVIKFLIKNRSAEFDKLTAKYDPSGEYKKKIEKFDAERAAAAKH*

>ApisCSP3
MVHLNLFVVLVASLVCFTLAEEKYTTKFDNFDVEKVLNNDRILTSYIECLLDQGNCTNEGRELKRVLPDALKTDCSKCTDVQKDRSERVIKFLIKNRSAEFDKLTAKYDPSGEYKKKLEKFSA*

>ApisCSP4

MDSRIALVCVVLAVFAVDQTVGAPQKDAASGPVYTTKYDNIDIDQILASKRLVNNYVQCLLDKKPCTPEGAELRKILPDALKTQCSKCNPGQKNAALKVVDRLQKDYDKEWKLLLDKWDPKREQFQKFQQFLVEEKKKGVVKF*

>ApisCSP5

MNCKILIALCCVAVYAAQANPAGVATATAADEEIKDLPAYMKRFEKLNVEQVLNNDRVLASHLKCFLNEGPCVQQSRDLKRVIPVIANNSCNGCTERQITTIKKSLNFLRTKKPVEWARLVKIYDPSGVKLNKFLDA*

>ApisCSP6

MNKLFLAVAFCIVTMMTVVQTAPAKYTTKYDNVNIDDILNNDRLVNSYFKCLMETGKCTPEGEEIKRWLPEAIENKCEDCSEKQKLGSEKIIKFLIEKKNDMWKQLEEKYDSKGLYRQRYSEDAKKLDIHI*

>ApisCSP7

MARSSSSVTMKVFVIAVCVCAALARPEEAKMENKPAVVKSETLAAPLPTTIVKRATPYVVSTQQDSSLPNVSEDVLDKALSDRRFVQRQLKCATGEGPCDPIGRKIKAHAPLVMRGMCVKCSQSEIKQIQRVMSHIQKNYPKEYTKMLKQYQSGF*

>ApisCSP8

MTNNNMNCPRSRPEIFSLLTVTAIAAVLVHQPTTVYCADGGTYPQQQLQQQQQQQQQQQQQQQFTAPSGYYVSTYDHIDVGRLLRNQKVVSGYVKCFVNEGPCTPDGKLVKAYLLPEIIRTVCGKCTPRQKEMARMVLRHIYTYRRADFDKIMQIYDTDGKKNEIINFMNQK*

>ApisCSP9

MSSFCLNSVILMTVITVVVARVAFAESTTSNDRPGSDIRLVKKDVDYNEDDADDREEGFFFRISHFFGFTSYDDDKPDFITTFDLIRLLDEKYAMKQFYCVINEEPCDAVGLRLKATIPEEINRDCERCTATETSNIRRILNYVKKHYPKFWERVEPIYRNNTTA*

>ApisCSP10

MVSKRFISVFMFMAVVGVSFSVPEDDDATKVVNKEVDHHSVIQEEIKKFLSMMEKINIDQILNNDRLMSNNVKCFLNEGSCTAQLREMKKMLPVLIKDSCSSCTKEQRNMIKKAMDAIKARRPNEYERVTKFFDPEKKYEKKLSEKLNES*

>SaveCSP2

FVVLVASLVCFTLAEEKYSTKYENFDVDKVLNDDSLLTSYINCLLDEENCTEEGQALKRVLPDALKTNCGKCTDTQKMKIEKILKFLMKNRSTDFDRLTAKYDPSGEYKKKLEKFSA

>SaveCSP4

MDSRIAVVCVVLAVFAVDQTVGAPQKDALAAGSPTTYTNKYDHIDIDQVLASKRLVNSYVQCLLDKKPCTPEGAELRKILPDALKTQCAKCSATQKNAALKVVDRLQKDYDKEWKQLLDKWDPKREQFQKFQQFLTEEKKKGVVKF*

>SaveCSP5

MNCKVLIALCCVAVYAAQANPAGAATATAADDEIKDFPAYMKRFDKLNVEQVLNNDRVLASHLKCFLNEGPCVQQSRDLKRVIPVIANNGCNGCTERQMTTIKKSLNFLRTKKPVEWARLVKIYDPSGTKLNKFLDA*

>SaveCSP7

MARSSSTSVTMKVFVMAVCVCAALARPEEAKMENKPTAVKSETLAAPLPTTIVKRATPQVVSIQKDASLPNVSEDVLDKALSDRRFVQRQLKCATGEGPCDPIGRKIKAHAPLVLRGMCVKCSQSEIKQIQRVMSHIQKNYPKEYTKMLKQYQSGF*

>SaveCSP1

MNLLAIFCYITMMCDSQFRRLEQPTAIPQVKRIEQPATIATRIGQATIAPRFGQPTVAPRFGQPTIAPRFGQATAAPQTGEAAIGPRIGQTFQNVNDSVSPTTDGRKTTRETSSYPTRYDFIDIEAVMNNDRIIKILFNCVMNQGPCTREGLELKRIVPDAIQTECAKCNERQRKQAGKVLAHLLQYKPEYWNMLVKKFDPNNIYLRKYMADNDDDEKLSLQKLSNNTTK*

>AglyCSP1

MNILTIFCYVTVMCDTQVKPAVSAQRLQSVNQNVTPTNDGRKTIRETSSYPTRYDYIDIEAVMNNERIIKILFNCVMSRGPCTREGLELKRIVPDAIQTECAKCNERQRKQAGKVLAHLLQYKPEYWKMLVQKFDPNNVYLRKYMADNDDDEKLSLQKLSNDTTKKKRNI*

>AglyCSP2

MGINIYKLKRIKMAHLNLFVVLIASLIYFTSAAEEKYTTKFDNFDVDKVLNNNRILTSYIKCLLDEGNCTNEGRELKRVLPDALKTDCSKCTDVQKDRSEKVIKFLIKNRSTDFDRLTAKYDPTGEYKKNLEKFETERATAKPLKA*

>AglyCSP4

MDSRIAVVCVVLAAFAVDQTVGAPQKDAVAASGPAYTTKYDHIDVDQVLASKRLVNSYVQCLLDKKPCTPEGAELRKILPDALKTQCAKCNTTQKNAALKVVDRLQKDYDAEWKQLLDKWDPKREHFQKFQQFLAEEKKKGFTKF*

>AglyCSP5

MHCKVLIALCCVAVYAVQASPAGTATAAAVSADDEIKDFPAYMKRFDKLNVEQVLNNDRVLASHLKCFLNEGPCVQQSRDLKRVIPVIANNGCNGCTERQMTTIKKSLNFLRTKKPTEWARLVKIYDPSGTKLNKFLDA*

>AglyCSP6

MIKLILAIAFCVTITMTVVQTAPAKYTTKYDNVNIDEILNNDRLVASYFKCLMETGKCTPEGEEIKRWLPEAVENKCEDCSEKQKLGSEKIIKFLFEKKNDMWKQLEAKYDPQGIYRQRYAEEAKKLNINV*

>AglyCSP7

MYMGNPSPSIDRIWSHYCHHLNTRSMSRSSSSVTMKVFVIAICVCAALARPEDVKVENKPAVIKSETLAVPLPTNIVKRATDTIQLDSSLPNVSEDVLDKALSDRRFVQRQLKCATGEGPCDPIGRKIKDILGADPSSRTASVERNVRQVFTVGNQTDSTCHVPYSEELSQGVHQDAETVPERILITMRRPCTIFLFMTSGFWHNLY*

>AglyCSP8

MNNSRGRYEIFSLLAVTIAAIMLVHQPATVRCADDGIITPQQQQQQTMMFTAPTGYYVSTYDHIDVGRLLRNNKVVSGYVKCFVNEGPCTPDGKLVKAYLLPEIIRTVCGKCTPRQKDMARMVLKHIYTYRQADFEKIMQIYDTDGKRNEILAFMNH*

>AglyCSP9

MSAFCLNSFILMTMITVIVTHATFIRSIKFDDRTGIDIHLVKRDTDDVKDDENSVESDEGFFYKITHFFQHHDKEDDDDDEEKPDFITTFDILKLLDEEYAMEQFYCVINEDPCDEVGMRLKATIPEEINRNCERCTSTERNNIRRILNYVKKHYPQFWKRVEPIYKKKI*

>AglyCSP10

MNSKIFISVFMFITIVSVSISVAERDDAVKAENKDDDSHPINREEIQRYMSMMEKINIDQMLNNTRLMSNNVKCFLNEGPCTAHLREMKKMVPMLVKDSCSSCTKEQKIMMKKAMDAVKARRPNDYEKLSKFFDPEGKYEKKFLENLNESK*
